# Supplementary material for: Characterization of A π–π stacking cocrystal of 4-nitrophthalonitrile directed toward application in photocatalysis
Source: Nat Commun. 2024 Feb 16;15:1455. doi: 10.1038/s41467-024-45686-1 (PMC10873295; doi:10.1038/s41467-024-45686-1)
Supplement: Supplementary file 1 — Supplementary Information [file 41467_2024_45686_MOESM1_ESM.pdf]

Supplementary Information

Characterization of A π-π Stacking Cocrystal of 4-Nitrophthalonitrile Directed Toward Application in Photocatalysis

Ting Xue,[a] Cheng Ma,[b] Le Liu,[a] Chunhui Xiao,*[a] Shao-Fei Ni,*[b] Rong Zeng*[a]

[a] School of Chemistry, Xi'an Jiaotong University, Xi'an 710049, P. R. China
[b] Department of Chemistry and Key Laboratory for Preparation and Application of Ordered Structural Materials of Guangdong, Shantou University, Shantou, 515063, Guangdong, P. R. China
Corresponding author: chunhuixiao@xjtu.edu.cn; sfni@stu.edu.cn; rongzeng@xjtu.edu.cn

Table of Contents

General Information	S2-S3
Optimization of the reaction conditions	S4-S6
Supplementary Table 4. The unsuccessful e-rich aromatics	S7
Preparation of Starting Materials	S8
Characterization of the cocrystal	S9-S11
Alkylation of e-rich aromatic	S12-S27
Mechanistic Studies for arene activation	S28-S37
Oxidation of e-rich aromatics	S38-S41
Hydrocarboxylation of alkenes	S42-S47
Mechanistic studies of formate activation	S48-S49
Mechanism of reductive hydrocarboxylation of alkynes	S50
Synthetic potentials	S51-S52
Spectra	S53-S134
References	S135-S137

Materials: PhH were distilled from Na/benzophenone before use. Benzylidenemalonenitril was purchased from Adamas-beta. Other commercially available chemicals were purchased and used without additional purification unless noted otherwise. Infrared spectra were recorded on a Nicolet iS5 using neat thin film technique. High-resolution mass spectra (HRSM) were obtained on a Waters I-Class VION IMS QTof and are reported as m/z (relative intensity). Accurate masses are reported for the molecular ion $[M+Na]^+$, $[M+H]^+$, $[M-OH]^+$, $[M-H]^-$ or $[M]^+$. ^1H NMR spectra were recorded on a Bruker-400 MHz or Bruker-600 MHz spectrometer, ^{13}C NMR spectra were recorded at 101 MHz or 151 MHz, and ^{19}F NMR spectra were recorded at 376 MHz. Unless otherwise noted, all spectra were acquired in $CDCl_3$. Chemical shifts are reported in parts per million (ppm, δ), downfield from tetramethylsilane (TMS, δ = 0.00 ppm) and are referenced to residual solvent ($CDCl_3$, δ = 7.26 ppm (^1H) and 77.00 ppm (^{13}C)). Coupling constants were reported in Hertz (Hz). Data for NMR spectra were reported as follows: s = singlet, d = doublet, t = triplet, q = quartet, dd = doublet of doublets, m = multiplet, coupling constant (Hz), and integration.

The alkenes including **1ac**,[1] **2b**,[2] **2c**,[3] **2d**,[4] **2f**,[2] **2g**,[2] **2h**,[2] **2i**,[2] **2j**,[5] **2l**,[6] **2m**,[2] **2n**,[7] **2o**,[8] **2p**,[9] **2q**[10], **10b**[11] were known and were prepared according to the known literature.

The LED light (100 W, emitting area: 30 × 30 mm) was assembled using the 390-395 nm chips purchased from GuangHong Chips. The emission spectra of the LED light is shown below (**Supplementary Figure 1**) and wavelength of peak intensity is 390-395 nm. The material of the reaction vessels is regular borosilicate glass. The distance from the light source to the reaction vessel is 5 cm, and the temperature was measured to be about 29 °C. (**Supplementary Figure 2**).

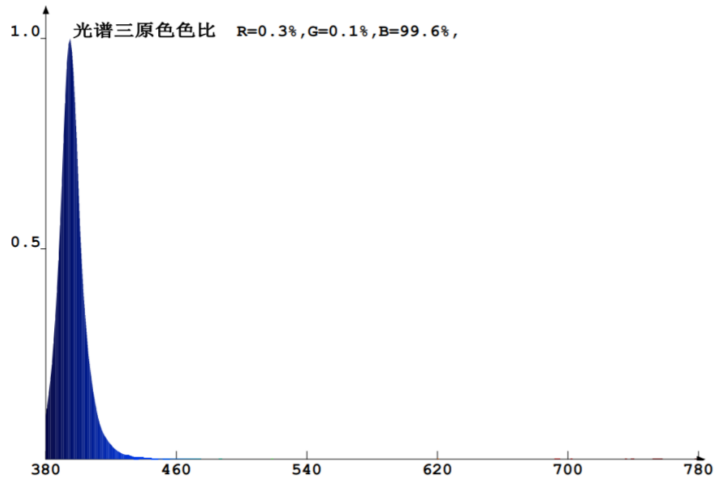

Supplementary Figure 1: **The emission spectrum of the LED light**. Emission peak is around 390-395 nm.

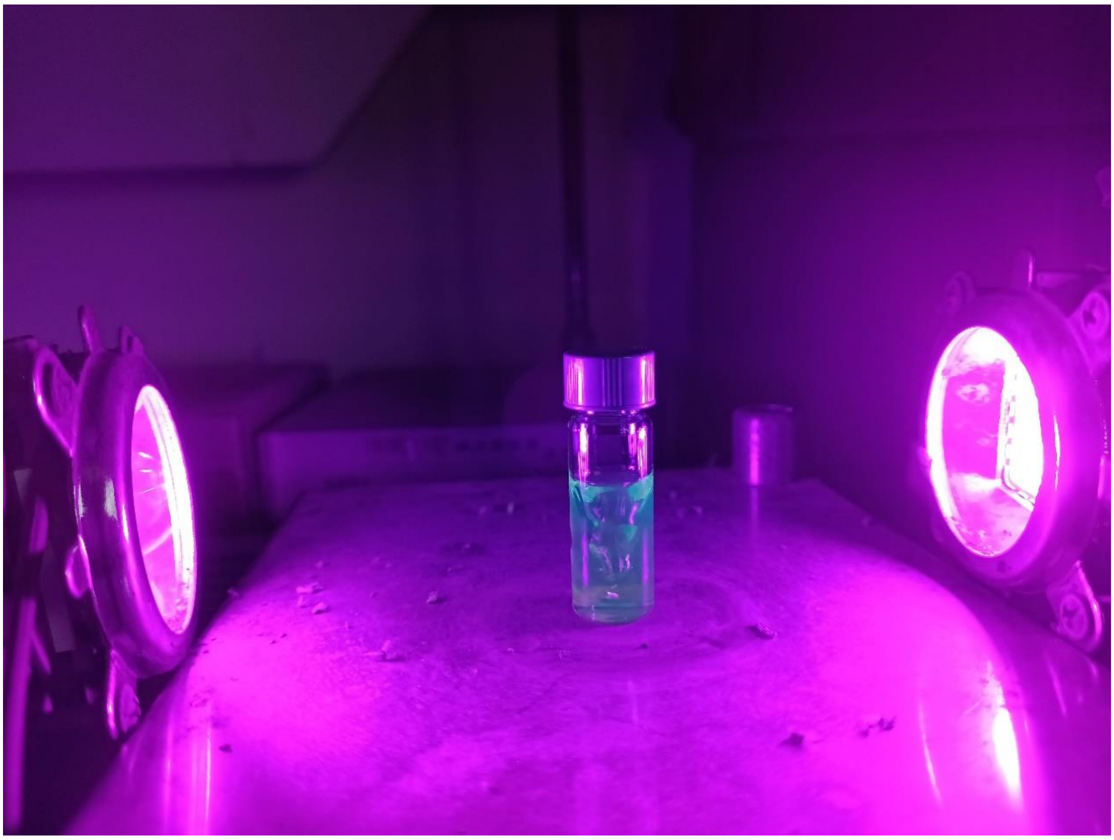

Supplementary Figure 2: **The setting-up reaction**. The material of the reaction vessels is regular borosilicate glass. The distance from the light source to the reaction vessel is 5 cm.

Optimization of reaction conditions

Supplementary Table 1. Optimization of the alkylation reaction[a,b]

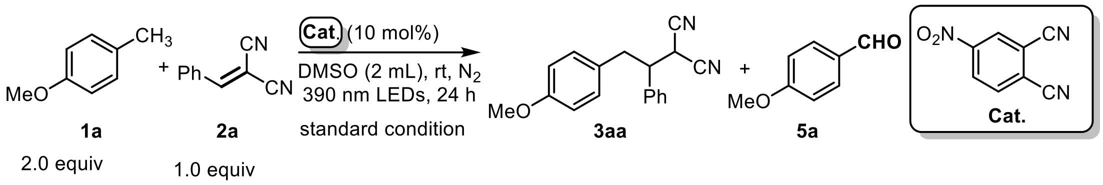

Entry	Variation from the standard conditions	Yield of **3aa** (%)[b]	Yield of **9a** (%)[b]
1	**none**	**75(71)**[c]	**2**
2	No **Cat.**	27	0
3	No LEDs	0	0
4	20 mol% of **Cat.** was used	72	5
5	5 mol% of **Cat.** was used	61	1
6	3.0 equiv of **1a** were used	72	2
7	1.5 equiv of **1a** were used	52	1
8	1.0 equiv of **1a** and 2.0 equiv of **2a**	58	<1%
9	365 nm LEDs were used	23	<1%
10	425 nm LEDs were used	9	<1%
11	460 nm LEDs were used	20	<1%
12	460 nm LEDs were used and no **cat.**	2	0
13	white light LEDs were used	3	<1%
14	DMF instead of DMSO	6	0
15	DMA instead of DMSO	0	0
16	DMP instead of DMSO	0	0
17	Benzene instead of DMSO	25	29
18	DME instead of DMSO	22	1
19	MeOH instead of DMSO	22	1
20	Cl_3CCH_2OH instead of DMSO	17	0
21[d]	BHT was added	14	0

[a] The standard reactions were conducted with **1a** (0.4 mmol), **2a** (0.2 mmol), 4-nitrophthalonitrile (0.02 mmol) in DMSO (2 mL) under nitrogen atmosphere at room temperature with 390 nm LEDs irradiation for 24 h [b] Yields were determined by ^{1}H NMR analysis of the crude product using 1,3,5-trimethoxybenzene as internal standard. [c]The yield in the parenthesis is based on isolation. [d]1.0 equiv Butylated Hydroxytoluene (BHT)

Supplementary Table 2. Optimization of the oxidation reaction[a,b]

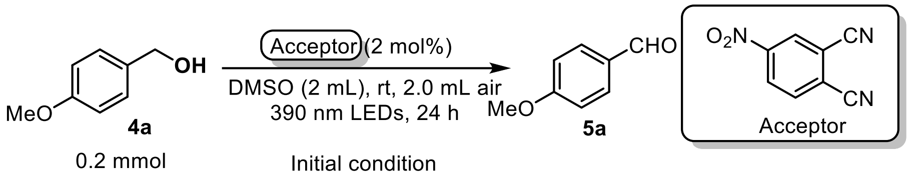

Entry	Variation from the initial conditions	Yield (%)
1	none	48[b]
2	w/o catalyst	7[c]
3	w/o LED and prolonged to 72 hours	0[b]
4	Benzyl alcohol was used instead of **4a**	<5%[c]
5	reaction time prolonged to 72 hours	85[b]
6	oxygen atmosphere	41[c]
7	nitrogen atmosphere	10[c]
8	4-nitrophthalonitrile (10 mol%) was used	73[c]
9	4-nitrophthalonitrile (10 mol%) was used and reaction time was 48 hours	84[c] (81)[d]

[a] The reactions were conducted with **4a** (0.2 mmol) and 4-nitrophthalonitrile (0.0004 mmol) in DMSO (2 mL) under 2.0 mL air atmosphere at room temperature with 390 nm LEDs irradiation for 24 h. [b] Yields were determined by GC analysis of the crude product using decane as internal standard. [c] Yields were determined by ^1H NMR analysis of the crude product using mesitylene as internal standard. [d] The yield in the parenthesis is based on isolation.

Supplementary Table 3. Optimization of the alylation reaction condition[a,b]

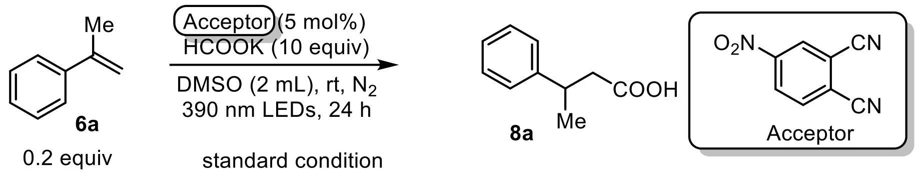

Entry	Variation from the standard conditions	Yield (%)[b]
1	none	82(68)[c]
2	w/o catalyst	0
3	w/o LED	0
4	HCOOH instead of HCOOK	0
5	HCOOCs instead of HCOOK	82
6	HCOOCs·H_2O instead of HCOOK	84
7	HCOOLi instead of HCOOK	46
8	$HCOONH_4$ instead of HCOOK	N.R.
9	H_2O (5.0 equiv.) was added	84
10	H_2O (7.0 equiv.) was added	73
11	DMAc instead of DMSO	0
12	DMF instead of DMSO	0
13[d]	BHT was added	0

[a] The reactions were conducted with **6a** (0.2 mmol), potassium formate (2.0 mmol) and 4-nitrophthalonitrile (0.004 mmol) in DMSO (2 mL) under nitrogen atmosphere at room temperature with 390 nm LED irradiation for 24 h [b] Yields were determined by ^{1}H NMR analysis of the crude product using dibromomethane as internal standard. [c] The yield in the parenthesis is based on isolation. [d] 1.0 equiv Butylated Hydroxytoluene (BHT)

Supplementary Table 4. The unsuccessful e-rich aromatics[a,b]

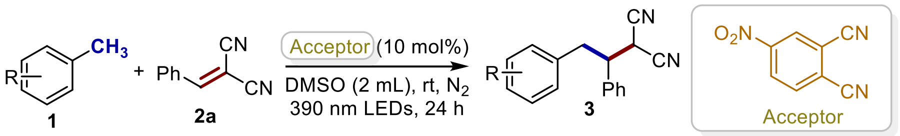

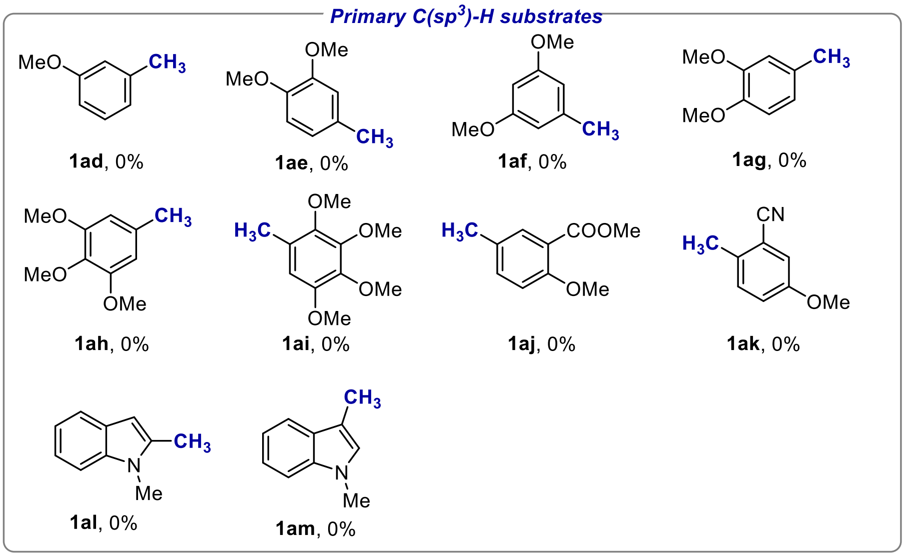

[a] The reactions were conducted with **1** (0.4 mmol), **2a** (0.2 mmol), 4-nitrophthalonitrile (0.2 mmol) in DMSO (2 mL) under nitrogen atmosphere at room temperature with 390 nm LED irradiation for 24 h. [b] All yields were calculated after isolation.

Preparation of Starting Materials

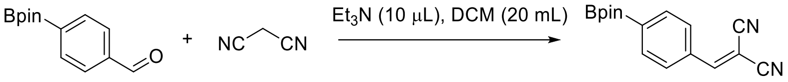

Typical Procedure 1: To a solution of **2e-CHO** (1.3918 g, 6.0 mmol) and $CH_2(CN)_2$ (0.6 mL, *d* = 1.049 g/ml, 9.0 mmol) in dry CH_2Cl_2 (20 mL) was added Et_3N (10 µl, *d* = 0.728 g/mL, 7.28 mg, 0.07 mmol). The resulting mixture was stirring for 24 h at room temperature. After removal of the solvent, the residue was purified by column chromatography on silica gel to afford compound **2e** (0.7797 g, 46%) (eluent: DCM/petroleum ether = 1/1): pale red solid. mp. 110.4-111.4 °C (Hexane/ethyl acetate). ^{1}H NMR (400 MHz, $CDCl_3$) 7.94 (d, *J* = 8.0 Hz, 2 H, Ar-H), 7.86 (d, *J* = 8.4 Hz, 2 H, Ar-H), 7.78 (s, 1 H, CH), 1.36 (s, 12 H, $CH_3 \times 4$). ^{13}C NMR (101 MHz, $CDCl_3$) δ 159.9, 135.6, 132.8, 129.6, 113.6, 112.4, 84.5, 83.6, 24.9. IR ν (neat, cm^{-1}) 2229. HRMS (ESI) m/z: $[M + K]^+$ Calcd for $C_{16}H_{17}BN_2O_2K$ 319.1015; found 319.1028.

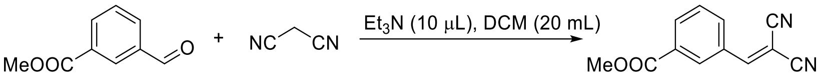

Following Typical Procedure 1, the reaction of **2k-CHO** (0.9842 g, 0.4 mmol), $CH_2(CN)_2$ (0.6 mL, *d* = 1.049 g/ml, 9.0 mmol), and Et_3N (10 µl, *d* = 0.728 g/mL, 7.28 mg, 0.07 mmol) in DCM (20.0 mL) for 12 h afforded **2k** (0.2245 g, 18%) (eluent: DCM/petroleum ether = 2/1): white solid. mp. 120.1-120.8 °C (Hexane/ethyl acetate). ^{1}H NMR (400 MHz, $CDCl_3$) δ 8.46-8.43 (m, 1 H, Ar-H), 8.28 (d, *J* = 7.6 Hz, 1 H, Ar-H), 8.21 (d, *J* = 8.0 Hz, 1 H, Ar-H), 7.84 (s, 1 H, CH), 7.69-7.62 (m, 1 H, Ar-H), 3.97 (s, 3 H, CH_3). ^{13}C NMR (101 MHz, $CDCl_3$) δ 165.4, 158.8, 135.0, 133.4, 132.4, 131.7, 131.1, 129.9, 113.2, 112.1, 84.5, 52.7. IR ν (neat, cm^{-1}) 2227, 1713. HRMS (ESI) m/z: $[M + NH_4]^+$ Calcd for $C_{12}H_{12}N_3O_2$ 230.0924; found 230.0920.

Characterization of the cocrystal of 1,3,5-trimethoxybenzen and 4-nitrophthalonitrile

1. X-ray Crystallography

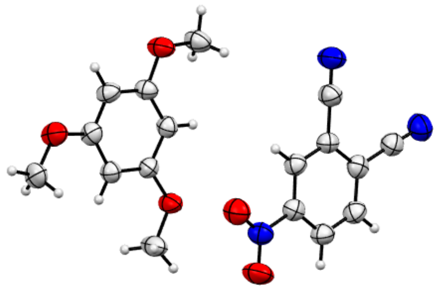

Crystal data for $C_{17}H_{15}N_3O_5$, M = 341.32, triclinic, space group P -1, final R indices [I > 2σ(I)], R_1 = 0.0707, wR_2 = 0.2204; R indices (all data), R_1 = 0.0791, wR_2 = 0.2258; a = 7.6134(5) Å, b = 10.1552(8) Å, c = 10.7929(9) Å, α = 79.319(3)°, β = 83.593(3)°, γ = 83.997(3)°, V = 811.87(11) $Å^3$, T = 300(2) K, Z = 2, reflection collected/unique 14884 / 2833 (Rint = 0.0420), number of observations [>2σ(I)] 2833, parameters: 230. CCDC 2277272 contains supplementary crystallographic data for structure of co-crystal of 1,3,5-trimethoxybenzen and 4-nitrophthalonitrile. These data can be obtained free of charge from The Cambridge Crystallographic Data Centre (CCDC) via www.ccdc.cam.ac.uk/data_request/cif.

2. UV-Vis absorption

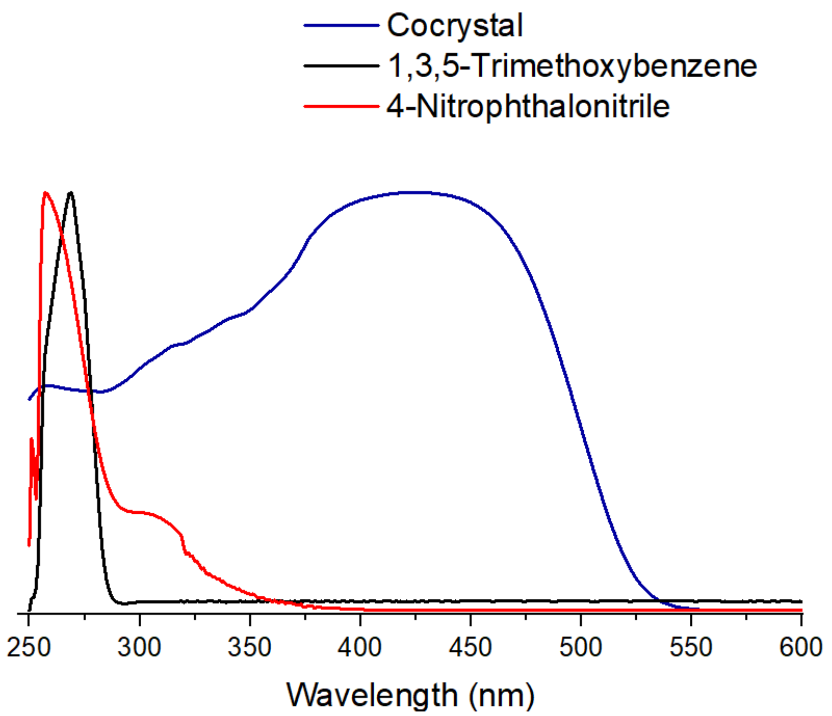

Supplementary Figure 3: **The UV-Vis absorption spectra**. The solution of 4-nitrophthalonitrile (0.1 mM) and 1,3,5-trimethoxybenzene (2.0 mM) in DMSO and the powder of cocrystal were measured using PE Lambda950.

.

3. DFT calculations

All calculations were performed using Gaussian 16, Revision A.03 package with CAM-B3LYP/6-31G(d) level of theory.[12,13] Time-dependent density functional theory (TD-DFT) calculation was carried out at TD-CAM-B3LYP/6-311G(d,p) level for the excited states. Hole-electron, and inter-fragment charge transfer (IFCT) analysis was performed using Multiwfn 3.8(dev) software package.[14,15]

Cartesian coordinates of the crystal structure:

EDA

E = -1196.19001100 a.u.

0 1

O	5.95860000	9.66130000	6.49560000
O	5.14580000	7.67190000	6.32140000
N	5.75900000	8.63530000	5.86850000
N	6.20820000	5.10450000	1.20050000
N	7.97620000	8.33460000	-0.54060000
C	6.27990000	8.53990000	4.49690000
C	6.89960000	9.63960000	3.94680000
H	7.01240000	10.41820000	4.44300000
C	7.34950000	9.56330000	2.64810000
H	7.77100000	10.29640000	2.26080000
C	7.17760000	8.40350000	1.91640000
C	6.55950000	7.29250000	2.50450000
C	6.10640000	7.36000000	3.81060000
H	5.69650000	6.62900000	4.21320000
C	6.36470000	6.07040000	1.76860000
C	7.62520000	8.34790000	0.54690000
O	1.98880000	5.38770000	3.18870000
O	2.75520000	9.67110000	5.11350000
O	3.89940000	9.03750000	0.65890000
C	2.44680000	6.67130000	3.03030000
C	2.94190000	7.14760000	1.82870000
H	2.98400000	6.60270000	1.07600000
C	3.37500000	8.46620000	1.78650000
C	3.28960000	9.28900000	2.88250000
H	3.56640000	10.17510000	2.83080000
C	2.78610000	8.78000000	4.06510000
C	2.36070000	7.47110000	4.16110000
H	2.02700000	7.13380000	4.96150000
C	2.14920000	9.25500000	6.32460000
H	2.05330000	10.01020000	6.91000000

H	1.28420000	8.88310000	6.13820000
H	2.69950000	8.59130000	6.74610000
C	2.14020000	4.51290000	2.06740000
H	1.86060000	3.62790000	2.31180000
H	1.60020000	4.82950000	1.33920000
H	3.06150000	4.49460000	1.79760000
C	4.06210000	8.23100000	-0.49840000
H	3.20180000	7.94580000	-0.81290000
H	4.50150000	8.74010000	-1.18360000
H	4.59460000	7.46260000	-0.27980000

6. EPR spectra

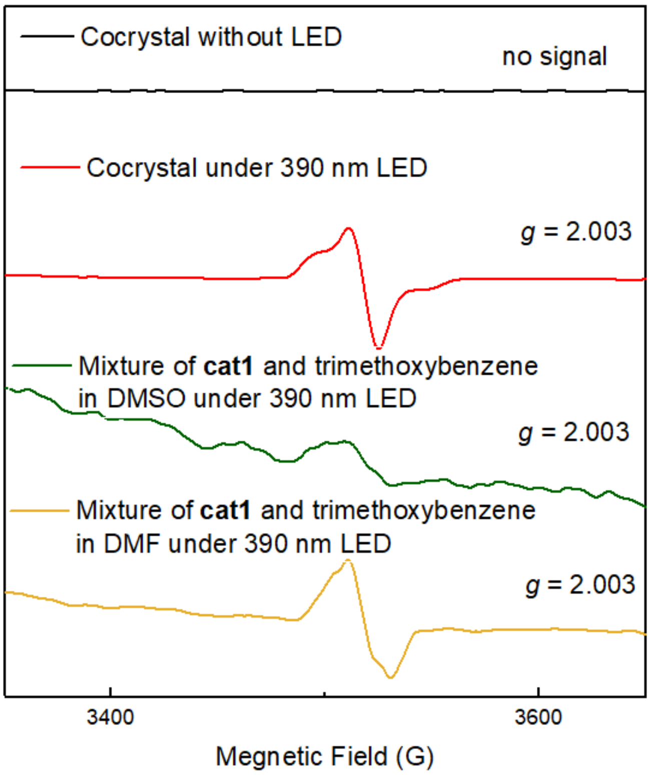

Supplementary Figure 4. EPR spectra. EPR spectra of the cocrystal and solution of 4-nitrophthalonitrile and 1,3,5-trimethoxybenzene in the absence or presence of 390 nm light.

The cocrystal of 4-nitrophthalonitrile and 1,3,5-trimethoxybenzene was used as starting sample, no radical signal was recorded without irradiation. Interestingly, once the sample was irradiated with 390 nm light (ex situ of the magnet), a build-up of a radical signal, assigned as a carbon radical (g = 2.003), was observed in the EPR spectrum. When the saturated solution of 4-nitrophthalonitrile and 1,3,5-trimethoxybenzene in DMSO was examined under irradiation, a similar signal could be obtained, however, in a relatively low resolution, probably due to the low concentration of radical species. Delightedly, a much higher resolution signal could be obtained by switching the solvent to DMF. These results indicated that the electron transfer might indeed proceed between 4-nitrophthalonitrile and 1,3,5-trimethoxybenzene to general the carbon radical species under the irradiation of 390 nm light.

C-H Alkylation of e-rich aromatic

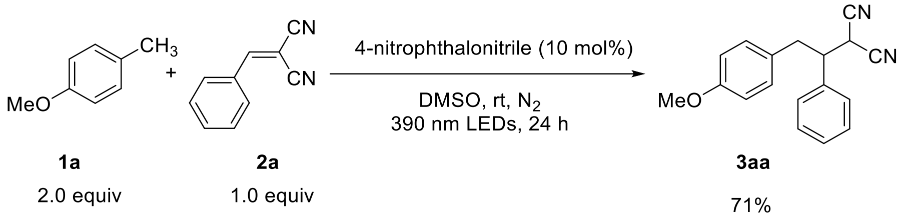

Typical Procedure 2: To a 4 mL vial were added **1a** (48.6 mg, 0.4 mmol), **2a** (30.6 mg, 0.2 mmol), and 4-nitrophthalonitrile (3.5 mg, 0.02 mmol) in DMSO (2.0 mL) in an N_2 glovebox. The vial was sealed and transferred out of glovebox. Under irradiation at 390 nm LEDs, the resulting mixture was stirred for 24 hours at rt. Saturated brine was then added and the aqueous layer was extracted with ethyl acetate (10 mL × 3). Evaporation and flash chromatography on silica gel afforded **3aa** (53.4 mg, 71%) (eluent: petroleum ether/ethyl acetate = 100/1 to 20/1): oil. ^{1}H NMR (400 MHz, $CDCl_3$) δ 7.49-7.36 (m, 5 H, Ar-H), 7.11 (d, *J* = 8.4 Hz, 2 H, Ar-H), 6.86 (d, *J* = 8.8 Hz, 2 H, Ar-H), 3.86 (d, *J* = 5.2 Hz, 1 H, CH), 3.80 (s, 3 H, CH_3), 3.45-3.38 (m, 1 H, CH), 3.26-3.16 (m, 2 H, CH_2). ^{13}C NMR (101 MHz, $CDCl_3$) δ 158.9, 136.4, 129.9, 129.1, 129.0, 128.4, 128.0, 114.4, 112.1, 111.4, 55.3, 48.4, 37.6, 28.3. IR ν (neat, cm^{-1}) 3064, 2254. The ^{1}H NMR spectra are matching with the known literature.[16]

1 gram scale reaction:

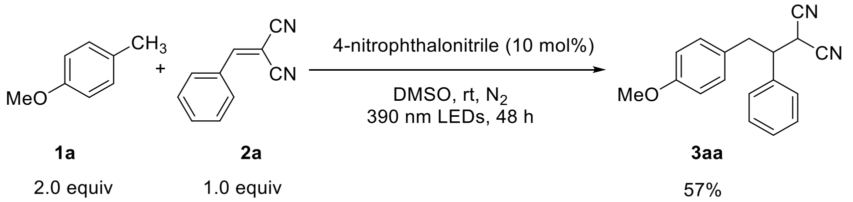

Following Typical Procedure 2, the reaction of **1a** (2.5 mL, *d* = 0.969 g/mL, 2.4225 g, 19.9 mmol), **2a** (1.5405 g, 10.0 mmol), and 4-nitrophthalonitrile (0.1733 g, 1.0 mmol) in DMSO (40.0 mL) afforded **3aa** (1.5653 g, 57%) (eluent: petroleum ether/ethyl acetate = 100/1 to 20/1): oil. ^{1}H NMR (400 MHz, $CDCl_3$) δ 7.46-7.36 (m, 5 H, Ar-H), 7.11 (d, *J* = 8.4 Hz, 2 H, Ar-H), 6.86 (d, *J* = 8.4 Hz, 2 H, Ar-H), 3.86 (d, *J* = 5.2 Hz, 1 H, CH), 3.80 (s, 3 H, CH_3), 3.45-3.37 (m, 1 H, CH), 3.26-3.16 (m, 2 H, CH_2).

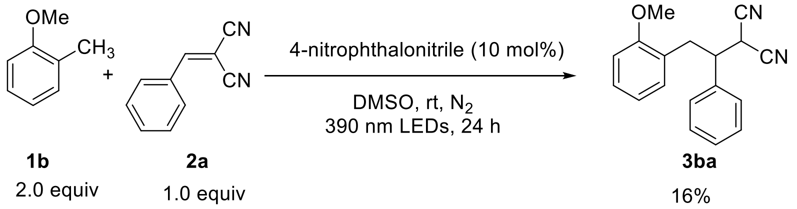

Following Typical Procedure 2, the reaction of **1b** (48.5 mg, 0.4 mmol), **2a** (30.9 mg, 0.2 mmol), and 4-nitrophthalonitrile (3.3 mg, 0.02 mmol) in DMSO (2.0 mL) afforded **3ba** (9.1 mg, 16%) (eluent: petroleum ether/ethyl acetate = 100/1 to 10/1): colorless oil. ^{1}H NMR (400 MHz, $CDCl_3$) δ 7.45-7.34 (m, 5 H, Ar-H), 7.31-7.25 (m, 1 H, Ar-H), 7.13-7.09 (m, 1 H, Ar-H), 6.93-6.86 (m, 2 H, Ar-H), 3.89-3.85 (m, 4 H, CH and CH_3),

3.64-3.56 (m, 1 H, CH), 3.34 (dd, J_1 = 13.6 Hz, J_2 = 9.6 Hz, 1 H, CH_2), 3.20 (dd, J_1 = 14.0 Hz, J_2 = 5.6 Hz, 1 H, CH_2). ^{13}C NMR (101 MHz, $CDCl_3$) δ 157.4, 137.1, 131.0, 129.0, 129.8, 128.0, 125.0, 120.9, 112.4, 111.7, 110.7, 55.2, 46.3, 33.7, 28.5. IR ν (neat, cm^{-1}) 3064, 2254. The ^1H NMR spectra are matching with the known literature.[17]

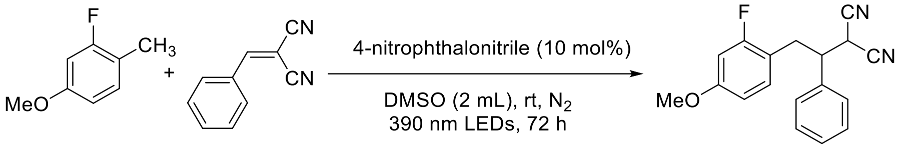

1c **2a** **3ca**

Following Typical Procedure 2, the reaction of **1c** (56.1 mg, 0.4 mmol), **2a** (30.9 mg, 0.2 mmol), and 4-nitrophthalonitrile (3.5 mg, 0.02 mmol) in DMSO (2.0 mL) for 72 h afforded **3ca** (26.4 mg, 45%) (eluent: petroleum ether/ethyl acetate = 100/1 to 20/1): colorless oil. ^1H NMR (400 MHz, $CDCl_3$) δ 7.45-7.34 (m, 5 H, Ar-H), 7.07-6.97 (m, 1 H, Ar-H), 6.59-6.66 (m, 2 H, Ar-H), 3.90 (d, J = 5.2 Hz, 1 H, CH), 3.78 (s, 3 H, CH_3), 3.56-3.49 (m, 1 H, CH), 3.32-3.17 (m, 2 H, CH_2). ^{13}C NMR (101 MHz, $CDCl_3$) δ161.7 (d, ^1J = 240.4 Hz), 160.4 (d, ^3J = 4.0 Hz), 136.3, 131.5 (d, ^3J = 6.1 Hz), 129.1, 129.0, 127.9, 115.3 (d, ^2J = 16.2 Hz), 111.92, 111.40, 110.2 (d, ^4J = 2.0 Hz), 102.1 (d, ^2J = 25.3 Hz), 55.6, 46.7, 31.9, 28.7. ^{19}F NMR (376 MHz, $CDCl_3$) δ -115.4. IR ν (neat, cm^{-1}) 3032, 2255. HRMS (ESI) m/z: $[M + H]^+$ Calcd for $C_{18}H_{16}FN_2O$ 295.1241; found 295.1233.

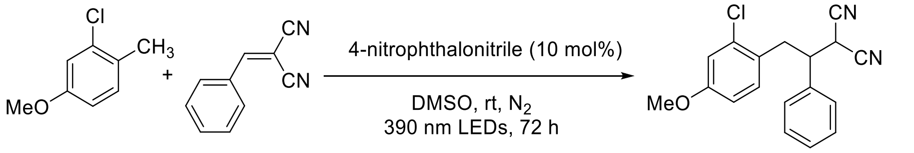

1d **2a** **3da**

2.0 equiv 1.0 equiv 55%

Following Typical Procedure 2, the reaction of **1d** (62.6 mg, 0.4 mmol), **2a** (30.9 mg, 0.2 mmol), and 4-nitrophthalonitrile (3.6 mg, 0.02 mmol) in DMSO (2.0 mL) afforded **3da** (34.2 mg, 55%) (eluent: petroleum ether/ethyl acetate = 100/1 to 20/1): colorless oil. ^1H NMR (400 MHz, $CDCl_3$) δ 7.45-7.35 (m, 5 H, Ar-H), 7.03 (d, J = 8.8 Hz, 1 H, Ar-H), 6.96 (d, J = 2.4 Hz, 1 H, Ar-H), 6.71 (dd, J_1 = 8.4 Hz, J_2 = 2.4 Hz 1 H, Ar-H), 3.90 (d, J = 5.2 Hz, 1 H, CH), 3.78 (s, 3 H, CH_3), 3.56-3.49 (m, 1 H, CH), 3.37 (dd, J_1 = 14.0 Hz, J_2 = 8.6 Hz, 1 H, CH_2), 3.27 (dd, J_1 = 14.4 Hz, J_2 = 7.0 Hz, 1 H, CH_2). ^{13}C NMR (101 MHz, $CDCl_3$) δ 159.6, 136.4, 134.7, 131.8, 129.2, 129.0, 127.9, 126.1, 115.5, 113.2, 111.9, 111.5, 55.5, 46.0, 36.0, 28.6. IR ν (neat, cm^{-1}) 3030, 2255. HRMS (ESI) m/z: $[M + H]^+$ Calcd for $C_{18}H_{16}ClN_2O$ 311.0946; found 311.0961.

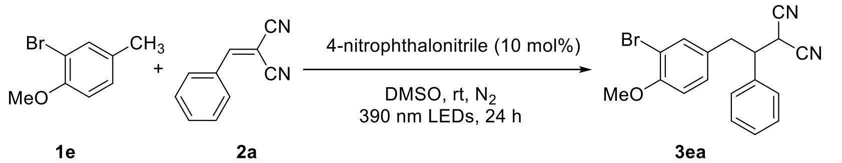

1e **2a** **3ea**

2.0 equiv 1.0 equiv 33%

Following Typical Procedure 2, the reaction of **1e** (81.0 mg, 0.4 mmol), **2a** (31.0 mg,

0.2 mmol), and 4-nitrophthalonitrile (3.7 mg, 0.02 mmol) in DMSO (2.0 mL) afforded **3ea** (23.3 mg, 33%) (eluent: petroleum ether/ethyl acetate = 100/1 to 20/1): colorless oil. ^{1}H NMR (400 MHz, $CDCl_3$) δ 7.46-7.32 (m, 6 H, Ar-H), 7.06 (dd, J_1 = 8.4 Hz, J_2 = 2.0 Hz, 1 H, Ar-H), 6.82 (d, J = 8.4 Hz, 1 H, Ar-H), 3.91-3.84 (m, 4 H, CH and CH_3), 3.46-3.38 (m, 1 H, CH), 3.26-3.12 (m, 2 H, CH_2). ^{13}C NMR (101 MHz, $CDCl_3$) δ 155.3, 136.0, 133.5, 130.0, 129.2, 129.1, 129.0, 127.9, 112.1, 111.9, 111.4, 56.2, 48.3, 37.3, 28.6. IR ν (neat, cm^{-1}) 3030, 2252. HRMS (ESI) m/z: $[M + Na]^+$ Calcd for $C_{18}H_{15}BrN_2ONa$ 377.0260; found 377.0273.

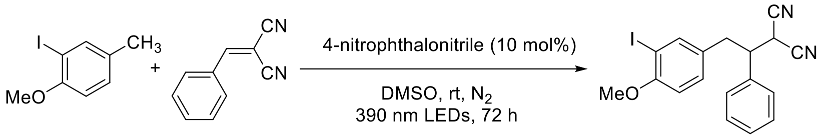

1f 2.0 equiv **2a** 1.0 equiv **3fa** 31%

Following Typical Procedure 2, the reaction of **1f** (99.1 mg, 0.4 mmol), **2a** (30.9 mg, 0.2 mmol), and 4-nitrophthalonitrile (3.6 mg, 0.02 mmol) in DMSO (2.0 mL) for 72 h afforded **3fa** (25.1 mg, 31%) (eluent: petroleum ether/ethyl acetate = 100/1 to 10/1): colorless oil. ^{1}H NMR (400 MHz, $CDCl_3$) δ 7.58 (d, J = 2.0 Hz, 1 H, Ar-H), 7.47-7.33 (m, 5 H, Ar-H), 7.10 (dd, J_1 = 8.4 Hz, J_2 = 2.0 Hz, 1 H, Ar-H), 6.74 (d, J = 8.4 Hz, 1 H, Ar-H), 3.91-3.84 (m, 4 H, CH and CH_3), 3.46-3.38 (m, 1 H, CH), 3.26-3.12 (m, 2 H, CH_2). ^{13}C NMR (101 MHz, $CDCl_3$) δ 157.5, 139.6, 136.1, 130.6, 130.1, 129.2, 129.1, 128.0, 111.9, 111.4, 86.6, 56.38, 48.3, 37.1, 28.5. IR ν (neat, cm^{-1}) 3030, 2254. HRMS (ESI) m/z: $[M + H]^+$ Calcd for $C_{18}H_{16}IN_2O$ 403.0302; found 403.0317.

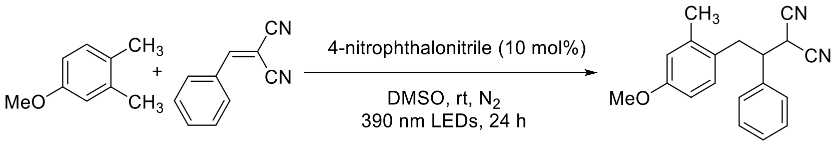

1g 2.0 equiv **2a** 1.0 equiv **3ga** 93%

Following Typical Procedure 2, the reaction of **1g** (54.5 mg, 0.4 mmol), **2a** (30.9 mg, 0.2 mmol), and 4-nitrophthalonitrile (3.6 mg, 0.02 mmol) in DMSO (2.0 mL) afforded **3ga** (54.4 mg, 93%) (eluent: petroleum ether/ethyl acetate = 20/1): colorless oil. ^{1}H NMR (400 MHz, $CDCl_3$) δ 7.51-7.34 (m, 5 H, Ar-H), 7.00 (d, J = 8.4 Hz, 1 H, Ar-H), 6.80-6.66 (m, 1 H, Ar-H), 3.92 (d, J = 5.2 Hz, 1 H, CH), 3.78 (s, 3 H, CH_3), 3.45-3.13 (m, 3 H, CH and CH_2), 2.30 (s, 3 H, CH_3). ^{13}C NMR (101 MHz, $CDCl_3$) δ 158.8, 137.7, 136.7, 130.6, 129.1, 129.0, 127.9, 126.8, 116.7, 112.2, 111.7, 111.5, 55.2, 47.1, 35.3, 28.4, 19.5. IR ν (neat, cm^{-1}) 3030, 2257. HRMS (ESI) m/z: $[M + K]^+$ Calcd for $C_{19}H_{18}N_2O_5K$ 329.1051; found 329.1067.

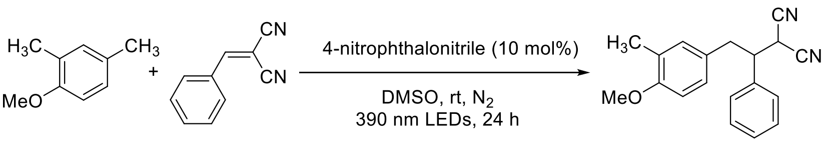

1h 2.0 equiv **2a** 1.0 equiv **3ha** 53%

Following Typical Procedure 2, the reaction of **1h** (54.0 mg, 0.4 mmol), **2a** (30.9 mg, 0.2 mmol), and 4-nitrophthalonitrile (3.3 mg, 0.02 mmol) in DMSO (2.0 mL) afforded

3ha (30.9 mg, 53%) (eluent: petroleum ether/ethyl acetate = 100/1 to 10/1): colorless oil. ^{1}H NMR (400 MHz, $CDCl_3$) δ 7.65-7.37 (m, 5 H, Ar-H), 7.12-6.95 (m, 2 H, Ar-H), 6.93-6.75 (m, 1 H, Ar-H), 4.05-3.78 (m, 4 H, CH and CH_3), 3.49-3.37 (m, 1 H, CH), 3.26-3.12 (m, 2 H, CH_2), 2.20 (s, 3 H, CH_3). ^{13}C NMR (101 MHz, $CDCl_3$) δ 157.2, 136.6, 131.0, 129.1, 129.0, 128.0, 127.5, 127.2, 112.3, 111.5, 110.3, 55.3, 48.5, 37.6, 28.2, 16.2. IR ν (neat, cm^{-1}) 3003, 2254. HRMS (ESI) m/z: $[M + H]^+$ Calcd for $C_{19}H_{19}N_2O$ 291.1492; found 291.1505.

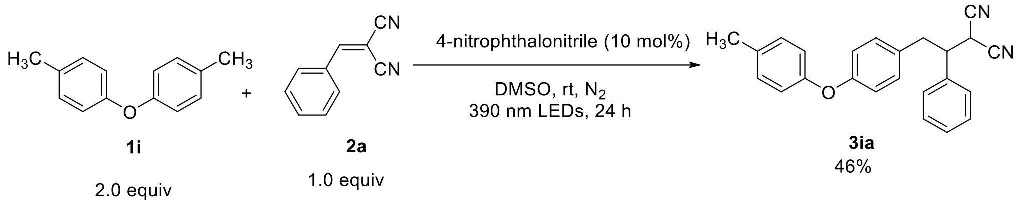

Following Typical Procedure 2, the reaction of **1i** (79.2 mg, 0.4 mmol), **2a** (30.9 mg, 0.2 mmol), and 4-nitrophthalonitrile (3.6 mg, 0.02 mmol) in DMSO (2.0 mL) afforded **3ia** (32.2 mg, 46%) (eluent: petroleum ether/ethyl acetate = 100/1 to 20/1): colorless oil. ^{1}H NMR (400 MHz, $CDCl_3$) δ 7.47-7.37 (m, 5 H, Ar-H), 7.17-7.08 (m, 4 H, Ar-H), 6.95-6.88 (m, 4 H, Ar-H), 3.88 (d, J = 5.2 Hz, 1 H, CH), 3.47-3.34 (m, 1 H, CH), 3.29-3.18 (m, 2 H, CH_2), 2.34 (s, 3 H, CH_3). ^{13}C NMR (151 MHz, $CDCl_3$) δ 157.4, 154.2, 136.4, 133.4, 130.6, 130.3, 130.1, 129.2, 129.1, 128.0, 119.3, 118.6, 112.0, 111.4, 48.4, 37.7, 28.5, 20.7. IR ν (neat, cm^{-1}) 3030, 2255. HRMS (ESI) m/z: $[M + Na]^+$ Calcd for $C_{24}H_{20}N_2ONa$ 375.1468; found 375.1478.

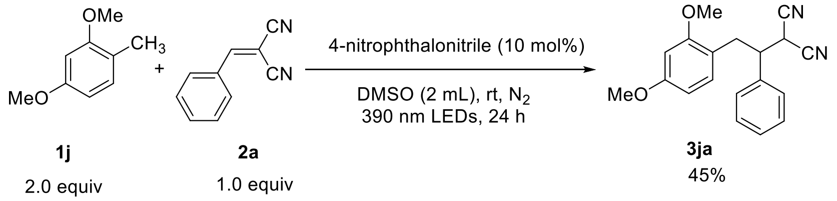

Following Typical Procedure 2, the reaction of **1j** (60.7 mg, 0.4 mmol), **2a** (31.0 mg, 0.2 mmol), and 4-nitrophthalonitrile (3.6 mg, 0.02 mmol) in DMSO (2.0 mL) afforded **3ja** (27.5 mg, 45%) (eluent: petroleum ether/ethyl acetate = 100/1 to 20/1): colorless oil. ^{1}H NMR (400 MHz, $CDCl_3$) δ 7.47-7.37 (m, 5 H, Ar-H), 7.01 (d, J = 8.4 Hz, 1 H, Ar-H), 6.48 (d, J = 2.4 Hz, 1 H, Ar-H), 6.42 (dd, J_1 = 8.4 Hz, J_2 = 2.4 Hz, 1 H, Ar-H), 3.88 (d, J = 4.8 Hz, 1 H, CH), 3.84 (s, 3 H, CH_3), 3.80 (s, 3 H, CH_3), 3.59-3.51 (m, 1 H, CH), 3.24 (dd, J_1 = 14.0 Hz, J_2 = 9.6 Hz, 1 H, CH_2), 3.13 (dd, J_1 = 14.0 Hz, J_2 = 5.6 Hz, 1 H, CH_2). ^{13}C NMR (101 MHz, $CDCl_3$) δ 160.5, 158.4, 137.2, 131.3, 129.0, 128.7, 128.0, 117.3, 112.5, 111.7, 104.3, 98.8, 55.3, 46.4, 33.2, 28.4. IR ν (neat, cm^{-1}) 3005, 2252. HRMS (ESI) m/z: $[M + H]^+$ Calcd for $C_{19}H_{19}N_2O_5$ 307.1441; found 307.1452.

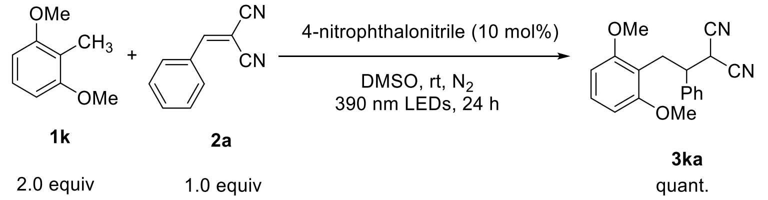

Following Typical Procedure 2, the reaction of **1k** (60.7 mg, 0.4 mmol), **2a** (30.8 mg, 0.2 mmol), and 4-nitrophthalonitrile (3.3 mg, 0.02 mmol) in DMSO (2.0 mL) afforded **3ka** (61.8 mg, quant.) (eluent: petroleum ether/ethyl acetate = 100/1 to 20/1): oil. ^{1}H

NMR (400 MHz, $CDCl_3$) δ 7.50-7.35 (m, 5 H, Ar-H), 7.28-7.21 (m, 1 H, Ar-H), 6.58 (d, *J* = 8.4 Hz, 2 H, Ar-H), 3.89 (d, *J* = 3.6 Hz, 1 H, CH), 3.83 (s, 3 H, CH_3), 3.54-3.43 (m, 2 H, CH_2), 3.19-3.09 (m, 1 H, CH). ^{13}C NMR (101 MHz, $CDCl_3$) δ 158.3, 137.7, 128.7, 128.5, 128.1, 113.3, 113.0, 112.0, 103.5, 55.4, 46.2, 28.5, 26.0. IR ν (neat, cm^{-1}) 3031, 2254. HRMS (ESI) m/z: $[M + H]^+$ Calcd for $C_{19}H_{19}N_2O_5$ 307.1441; found 307.1446.

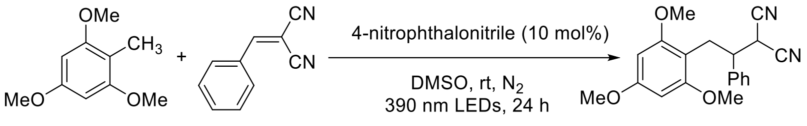

1l 2.0 equiv; **2a** 1.0 equiv; **3la** 94%

Following Typical Procedure 2, the reaction of **1l** (72.6 mg, 0.4 mmol), **2a** (30.9 mg, 0.2 mmol), and 4-nitrophthalonitrile (3.7 mg, 0.02 mmol) in DMSO (2.0 mL) afforded **3la** (63.2 mg, 94%) (eluent: petroleum ether/ethyl acetate = 20/1): oil. ^{1}H NMR (400 MHz, $CDCl_3$) δ 7.53-7.35 (m, 5 H, Ar-H), 6.15 (s, 2 H, Ar-H), 3.95-3.71 (m, 10 H, CH and 3 ×CH_3), 3.45-3.33 (m, 2 H, CH_2), 3.11-3.00 (m, 1 H, CH). ^{13}C NMR (101 MHz, $CDCl_3$) δ 160.6, 159.0, 137.8, 128.7, 128.5, 128.1, 113.1, 112.0, 105.7, 90.3, 55.3, 46.6, 28.4, 25.7. IR ν (neat, cm^{-1}) 3002, 2254. HRMS (ESI) m/z: $[M + H]^+$ Calcd for $C_{20}H_{21}N_2O_3$ 337.1547; found 337.1554.

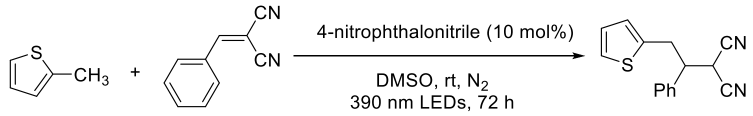

1m 2.0 equiv; **2a** 1.0 equiv; **3ma** 85%

Following Typical Procedure 2, the reaction of **1m** (39.3 mg, 0.4 mmol), **2a** (30.9 mg, 0.2 mmol), and 4-nitrophthalonitrile (3.6 mg, 0.02 mmol) in DMSO (2.0 mL) for 72 h afforded **3ma** (42.8 mg, 85%) (eluent: petroleum ether/ethyl acetate = 100/1 to 20/1): colorless oil. ^{1}H NMR (400 MHz, $CDCl_3$) δ 7.48-7.38 (m, 5 H, Ar-H), 7.22 (d, *J* = 5.2 Hz, 1 H, Ar-H), 6.98-6.94 (m, 1 H, Ar-H), 6.93-6.89 (m, 1 H, Ar-H), 3.98 (d, *J* = 4.8 Hz, 1 H, CH), 3.63-3.41 (m, 3 H, CH and CH_2). ^{13}C NMR (101 MHz, $CDCl_3$) δ 138.5, 135.8, 129.2, 128.0, 127.3, 127.0, 125.3, 111.8, 111.2, 48.4, 32.6, 28.5. IR ν (neat, cm^{-1}) 3033, 2254. HRMS (ESI) m/z: $[M + H]^+$ Calcd for $C_{15}H_{13}N_2S$ 253.0794; found 253.0791.

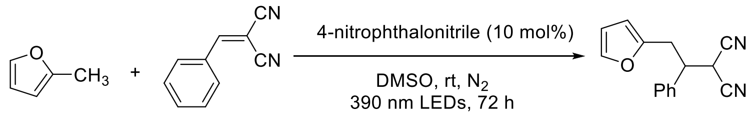

1n 2.0 equiv; **2a** 1.0 equiv; **3na** 44%

Following Typical Procedure 2, the reaction of **1n** (32.9 mg, 0.4 mmol), **2a** (31.0 mg, 0.2 mmol), and 4-nitrophthalonitrile (3.6 mg, 0.02 mmol) in DMSO (2.0 mL) for 72 h afforded **3na** (21.0 mg, 44%) (eluent: petroleum ether/ethyl acetate = 50/1 to 20/1): colorless oil. ^{1}H NMR (400 MHz, $CDCl_3$) δ 7.48-7.32 (m, 5 H, Ar-H), 6.34-6.30 (m, 1 H, Ar-H), 6.17 (d, *J* = 3.2 Hz, 1 H, Ar-H), 3.98 (d, *J* = 4.8 Hz, 1 H, CH), 3.68-3.60 (m, 1 H, CH), 3.36 (dd, J_1 = 15.4 Hz, J_2 = 9.4 Hz, 1 H, CH_2), 3.26 (dd, J_1 = 15.4 Hz, J_2 =

5.8 Hz, 1 H, CH_2). ^{13}C NMR (101 MHz, $CDCl_3$) δ 150.1, 142.5, 136.1, 129.2, 127.8, 111.8, 111.2, 110.6, 108.7, 45.3, 31.0, 28.6. IR ν (neat, cm^{-1}) 2257. HRMS (ESI) m/z: $[M]^+$ Calcd for $C_{15}H_{12}N_2O$ 236.0944; found 236.0936.

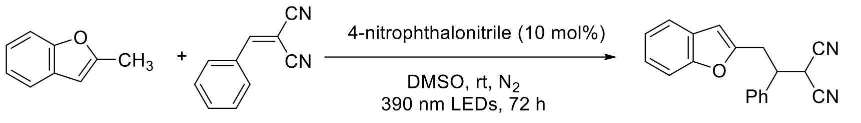

1o 2.0 equiv | **2a** 1.0 equiv | **3oa** 81%

Following Typical Procedure 2, the reaction of **1o** (52.8 mg, 0.4 mmol), **2a** (30.7 mg, 0.2 mmol), and 4-nitrophthalonitrile (3.3 mg, 0.02 mmol) in DMSO (2.0 mL) for 72 h afforded **3oa** (46.1 mg, 81%) (eluent: petroleum ether/ethyl acetate = 100/1 to 10/1): colorless oil. ^{1}H NMR (400 MHz, $CDCl_3$) δ 7.56-7.35 (m, 7 H, Ar-H), 7.33-7.19 (m, 2 H, Ar-H), 6.56 (s, 1 H, Ar-H), 4.04 (d, J = 5.2 Hz, 1 H, CH), 3.86-3.76 (m, 1 H, CH), 3.51 (dd, J_1 = 13.4 Hz, J_2 = 9.2 Hz, 1 H, CH_2), 3.42 (dd, J_1 = 15.2 Hz, J_2 = 6.4 Hz, 1 H, CH_2). ^{13}C NMR (101 MHz, $CDCl_3$) δ 154.9, 152.8, 135.7, 129.3, 128.0, 127.8, 124.4, 123.1, 120.9, 111.6, 111.1, 105.7, 44.7, 31.4, 28.8. IR ν (neat, cm^{-1}) 3034, 2255. HRMS (ESI) m/z: $[M + H]^+$ Calcd for $C_{19}H_{15}N_2O$ 287.1179; found 287.1165.

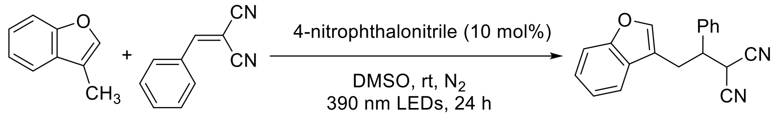

1p 2.0 equiv | **2a** 1.0 equiv | **3pa** 14%

Following Typical Procedure 2, the reaction of **1p** (52.8 mg, 0.4 mmol), **2a** (30.6 mg, 0.2 mmol), and 4-nitrophthalonitrile (3.3 mg, 0.02 mmol) in DMSO (2.0 mL) for 72 h afforded **3pa** (7.9 mg, 14%) (eluent: petroleum ether/ethyl acetate = 100/1 to 20/1): colorless oil. ^{1}H NMR (400 MHz, $CDCl_3$) δ 7.53-7.28 (m, 10 H, Ar-H), 3.98 (d, J = 5.2 Hz, 1 H, CH), 3.68-3.59 (m, 1 H, CH × 2), 3.49-3.529 (m, 2 H, CH_2). ^{13}C NMR (101 MHz, $CDCl_3$) δ ^{13}C NMR (151 MHz, $CDCl_3$) δ 155.4, 142.9, 136.2, 129.4, 129.3, 127.8, 126.9, 125.0, 123.0, 119.0, 115.3, 111.9, 111.8, 111.4, 46.0, 29.0, 26.9. IR ν (neat, cm^{-1}) 3033, 2254. HRMS (ESI) m/z: $[M + H]^+$ Calcd for $C_{19}H_{15}N_2O$ 287.1179; found 287.1168.

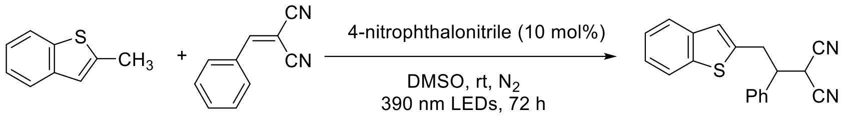

1q 2.0 equiv | **2a** 1.0 equiv | **3qa** 61%

Following Typical Procedure 2, the reaction of **1q** (59.6 mg, 0.4 mmol), **2a** (30.6 mg, 0.2 mmol), and 4-nitrophthalonitrile (3.6 mg, 0.02 mmol) in DMSO (2.0 mL) for 72 h afforded **3qa** (36.4 mg, 61%) (eluent: petroleum ether/ethyl acetate = 100/1 to 20/1): colorless oil. ^{1}H NMR (400 MHz, $CDCl_3$) δ 7.74 (dd, J_1 = 27.4 Hz, J_2 = 7.4 Hz, 2 H, Ar-H), 7.41-7.27 (m, 7 H, Ar-H), 7.15 (s, 1 H, Ar-H), 4.02 (d, J = 4.8 Hz, 1 H, CH), 3.71-3.50 (m, 3 H, CH_2 and CH). ^{13}C NMR (101 MHz, $CDCl_3$) δ 139.6, 139.3, 135.6, 129.4, 128.0, 124.7, 124.6, 123.9, 123.4, 122.3, 111.7, 111.2, 47.7, 33.6, 28.7. IR ν (neat, cm^{-1}) 3032, 2255, 2227. HRMS (ESI) m/z: $[M + H]^+$ Calcd for $C_{19}H_{15}N_2S$

303.0951; found 303.0958.

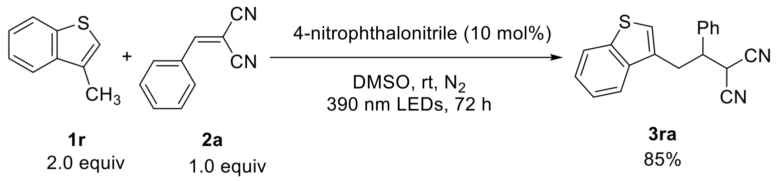

Following Typical Procedure 2, the reaction of **1r** (59.3 mg, 0.4 mmol), **2a** (30.6 mg, 0.2 mmol), and 4-nitrophthalonitrile (3.5 mg, 0.02 mmol) in DMSO (2.0 mL) for 72 h afforded **3ra** (51.2 mg, 85%) (eluent: petroleum ether/ethyl acetate = 100/1 to 10/1): colorless oil. ^{1}H NMR (400 MHz, $CDCl_3$) δ 7.93-7.88 (m, 1 H, Ar-H), 7.78-7.74 (m, 1 H, Ar-H), 7.48-7.38 (m, 7 H, Ar-H), 7.18 (s, 1 H, Ar-H), 3.92 (d, *J* = 4.8 Hz, 1 H, CH), 3.69-3.58 (m, 2 H, CH_2), 3.54-3.45 (m, 1 H, CH). ^{13}C NMR (101 MHz, $CDCl_3$) δ 140.7, 137.8, 136.4, 130.9, 129.3, 129.2, 127.8, 124.8, 124.7, 124.5, 123.3, 121.1, 111.9, 111.5, 45.9, 31.5, 28.9. IR ν (neat, cm^{-1}) 3030, 2253. HRMS (ESI) m/z: $[M + H]^+$ Calcd for $C_{19}H_{15}N_2S$ 303.0951; found 303.0952.

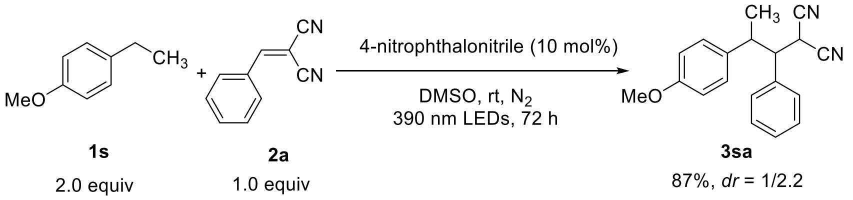

Following Typical Procedure 2, the reaction of **1s** (54.3 mg, 0.4 mmol), **2a** (30.9 mg, 0.2 mmol), and 4-nitrophthalonitrile (3.6 mg, 0.02 mmol) in DMSO (2.0 mL) for 72 h afforded **3sa** (50.9 mg, 87%, *dr* = 1/2.2) (eluent: petroleum ether/ethyl acetate = 100/1 to 20/1): colorless oil. ^{1}H NMR (400 MHz, $CDCl_3$) δ 7.55-7.34 (m, 3.89 H, Ar-H), 7.31-7.26 (m, 2 H, Ar-H), 6.97-6.93 (m, 2 H, Ar-H), 6.87-6.83 (m, 0.6 H, Ar-H), 6.75-6.71 (m, 0.6 H, Ar-H), [4.05 (d, *J* = 8.4 Hz, 0.3 H) and 3.64 (d, *J* = 4.4 Hz, 0.67 H), CH], [3.84 (s, 2.13 H) and 3.75 (s, 0.9 H), OMe] , 3.52-3.05 (m, 2 H of CH × 2), [1.39 (d, *J* = 6.8 Hz, 0.92 H) and 1.12 (d, *J* = 6.8 Hz, 2.1 H), CH_3]. ^{13}C NMR (101 MHz, $CDCl_3$) δ major isomer: 159.2, 135.6, 134.1, 129.2, 128.6, 128.5, 128.1, 114.9, 112.2, 111.5, 55.3, 53.8, 41.2, 28.7, 20.6. Minor isomer: 158.6, 134.7, 132.2, 129.1, 129.0, 128.8, 127.9, 113.7, 112.5, 111.9, 55.1, 52.6, 40.6, 27.4, 20.1. IR ν (neat, cm^{-1}) 3033, 2253. The ^{1}H NMR spectra are matching with the known literature.[7]

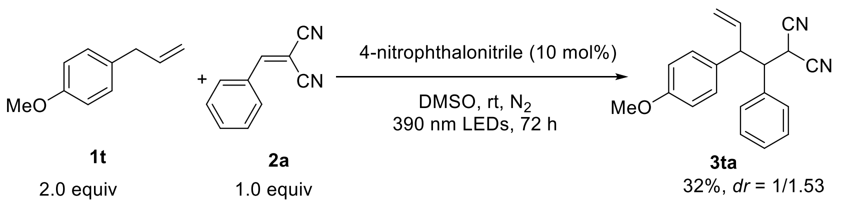

Following Typical Procedure 2, the reaction of **1t** (59.3 mg, 0.4 mmol), **2a** (30.9 mg, 0.2 mmol), and 4-nitrophthalonitrile (3.3 mg, 0.02 mmol) in DMSO (2.0 mL) for 72 h afforded **3ta** (19.5 mg, 32%, *dr* = 1/1.53) (eluent: petroleum ether/ethyl acetate = 20/1 to 10/1): colorless oil. ^{1}H NMR (400 MHz, $CDCl_3$) δ 7.47-7.35 (m, 5 H, Ar-H), 7.34-7.15 (m, 3 H, Ar-H), 6.99-6.80 (m, 2 H, Ar-H and CH), 6.71-6.46 (m, 1 H, CH_2), 6.01-4.83 (m, 1 H of CH_2), [4.07 (d, *J* = 5.6 Hz, 0.56 H), 3.95 (d, *J* = 5.6 Hz, 0.36 H), CH]

[3.82 (s, 1.21 H), 3.79 (s, 1.85 H), CH_3], 3.45-2.81 (m, 2 H of CH). ^{13}C NMR (101 MHz, $CDCl_3$) (a pair of diastereoisomers) δ 159.4, 158.8, 136.5, 136.1, 134.2, 132.5, 129.9, 129.2, 129.1, 129.0, 129.0, 128.9, 128.84, 128.81, 128.7, 128.6, 128.0, 127.9, 127.5, 124.6, 121.6, 118.7, 118.0, 115.0, 114.0, 113.9, 112.0, 111.5, 55.3, 46.7, 46.4, 36.7, 35.8, 30.7, 28.8. IR ν (neat, cm^{-1}) 3030, 2255. HRMS (ESI) m/z: $[M + H]^+$ Calcd for $C_{20}H_{19}N_2O$ 303.1492; found 303.1490.

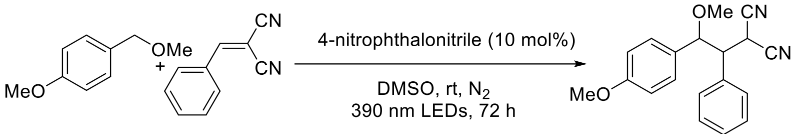

1u 2.0 equiv **2a** 1.0 equiv **3ua** 91%, *dr* = 1/1.8

Following Typical Procedure 2, the reaction of **1u** (60.5 mg, 0.4 mmol), **2a** (30.6 mg, 0.2 mmol), and 4-nitrophthalonitrile (3.3 mg, 0.02 mmol) in DMSO (2.0 mL) for 72 h afforded **3ua** (55.4 mg, 91%, *dr* = 1/1.8) (eluent: petroleum ether/ethyl acetate = 100/1 to 10/1): colorless oil. ^{1}H NMR (400 MHz, $CDCl_3$) δ 7.35-7.17 (m, 5 H, Ar-H), 7.05-6.94 (m, 2 H, Ar-H), 6.81-6.70 (m, 2 H, Ar-H), 4.79-4.72 (m, 1 H, CH), [4.55 (d, J = 10.4 Hz, 0.63 H), 3.27 (d, J = 8.8 Hz, 0.36 H), CH], [3.77 (s, 1.11 H), 3.73 (s, 1.88 H), CH_3], 3.45-3.34 (m, 1 H of CH), [3.27 (s, 1.97 H), 3.24 (s, 1.21 H), CH_3]. ^{13}C NMR (101 MHz, $CDCl_3$) (a pair of diastereoisomers) δ 159.6, 159.5, 134.0, 133.5, 129.2, 128.9, 128.9, 128.8, 128.8, 128.7, 128.6, 128.6, 128.2, 113.9, 113.7, 112.3, 112.2, 112.0, 82.8, 81.9, 57.2, 56.6, 55.2 55.1, 53.3, 53.0, 26.80, 26.6. IR ν (neat, cm^{-1}) 3033, 2254. HRMS (ESI) m/z: $[M + H]^+$ Calcd for $C_{19}H_{18}N_2O_2$ 307.1441; found 307.1443.

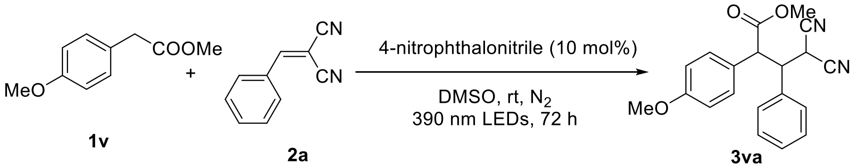

1v 2.0 equiv **2a** 1.0 equiv **3va** 64%

Following Typical Procedure 2, the reaction of **1v** (72.3 mg, 0.4 mmol), **2a** (30.6 mg, 0.2 mmol), and 4-nitrophthalonitrile (3.6 mg, 0.02 mmol) in DMSO (2.0 mL) for 72 h afforded **3va** (42.3 mg, 64%, *dr* = 1/1.0) (eluent: petroleum ether/ethyl acetate = 100/1 to 10/1): oil. ^{1}H NMR (400 MHz, $CDCl_3$) δ 7.57-7.37 (m, 3 H, Ar-H), 7.32-7.16 (m, 3 H, Ar-H), 7.02-6.90 (m, 2 H, Ar-H), 6.72-7.63 (m, 1 H, Ar-H), 4.63 (d, J = 4.8 Hz, 0.5 H of CH), 4.33-4.11 (m, 1 H, CH), 3.95-3.40 (m, 7.5 H, CH × 2 and CH_3 × 2). ^{13}C NMR (151 MHz, $CDCl_3$) (a pair of diastereoisomers) δ 174.0, 172.9, 171.0, 160.3, 159.1, 158.7, 134.5, 133.9, 129.4, 129.3, 129.3, 129.2, 128.91, 128.88, 128.6, 128.3, 127.71, 126.67, 125.9, 115.3, 114.2, 113.9, 111.6, 111.5, 110.9, 55.4, 55.1, 53.8, 53.0, 52.7, 52.4, 52.3, 52.1, 49.2, 48.1, 28.0, 27.7. IR ν (neat, cm^{-1}) 2255, 1731. HRMS (ESI) m/z: $[M]^+$ Calcd for $C_{20}H_{19}N_2O_3$ 334.1312; found 334.1320.

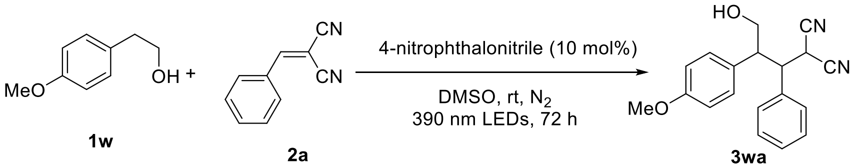

1w 2.0 equiv **2a** 1.0 equiv **3wa** 37%, *dr* = 1/2.74

Following Typical Procedure 2, the reaction of **1w** (60.9 mg, 0.4 mmol), **2a** (30.6 mg, 0.2 mmol), and 4-nitrophthalonitrile (3.6 mg, 0.02 mmol) in DMSO (2.0 mL) for 72 h afforded **3wa** (22.2 mg, 37%, *dr* = 1/2.74) (eluent: petroleum ether/ethyl acetate = 2/1): colorless oil. ^{1}H NMR (400 MHz, $CDCl_3$) δ 7.57-6.74 (m, 9 H, Ar-H), 4.51 (brs, 1H, OH), 4.29-4.14 (m, 1.57 H of CH_2), 3.87-3.72 (m, 4.63 H), 3.63-3.59 (m, 0.53 H), 3.52-3.41 (m, 0.57 H), 2.98-2.89 (m, 0.77 H). ^{13}C NMR (151 MHz, $CDCl_3$) δ 164.0, 159.7, 158.8, 141.6, 134.9, 130.5, 129.8, 129.7, 129.4, 129.1, 128.7, 128.4, 127.9, 127.6, 127.1, 120.7, 115.2, 114.1, 113.5, 112.0, 111.4, 70.4, 64.3, 60.2, 55.4, 55.2, 48.9, 48.8, 46.5, 45.1, 28.6. IR ν (neat, cm^{-1}) 3354, 2184. HRMS (ESI) m/z: [M + H]$^+$ Calcd for $C_{19}H_{19}N_2O_2$ 307.1441; found 307.1431.

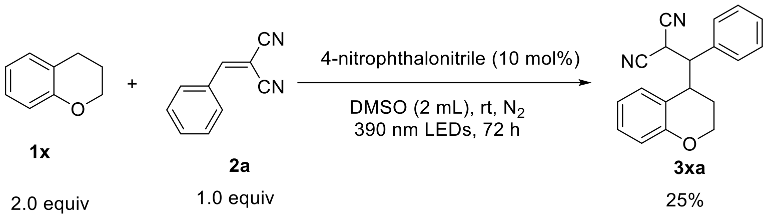

Following Typical Procedure 2, the reaction of **1x** (53.3 mg, 0.4 mmol), **2a** (30.6 mg, 0.2 mmol), and 4-nitrophthalonitrile (3.3 mg, 0.02 mmol) in DMSO (2.0 mL) for 72 h afforded **3xa** (14.0 mg, 25%) (eluent: petroleum ether/ethyl acetate = 100/1 to 10/1): colorless oil. ^{1}H NMR (400 MHz, $CDCl_3$) δ 7.42-7.32 (m, 3 H, Ar-H), 7.14-7.10 (m, 3 H, Ar-H), 6.83-6.77 (m, 1 H, Ar-H), 6.69-6.63 (m, 1 H, Ar-H), 6.56-6.52 (m, 1 H, Ar-H), 4.25-4.13 (m, 2 H, CH and CH_2), 3.90-3.81 (m, 1 H, CH_2), 3.63-3.54 (m, 1 H, CH), 3.52-3.46 (m, 1 H, CH), 2.42-2.27 (m, 1 H, CH_2), 2.21-2.11 (m, 1 H, CH_2). ^{13}C NMR (101 MHz, $CDCl_3$) δ 154.6, 136.3, 129.5, 129.3, 129.2, 129.0, 128.5, 120.2, 120.0, 117.40, 112.0, 111.9, 62.0, 50.6, 36.2, 27.2, 25.6. IR ν (neat, cm^{-1}) 3332, 2253. HRMS (ESI) m/z: [M + H]$^+$ Calcd for $C_{19}H_{16}N_2O$ 289.1335; found 289.1345.

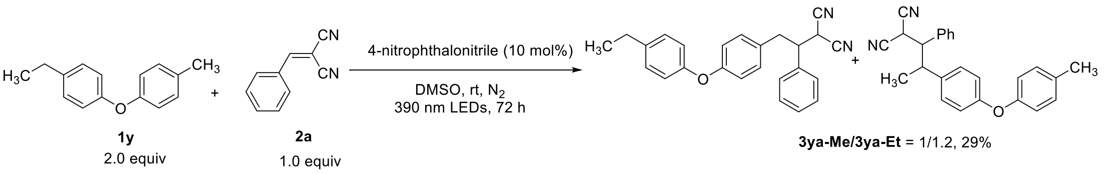

Following Typical Procedure 2, the reaction of **1y** (84.4 mg, 0.4 mmol), **2a** (30.9 mg, 0.2 mmol), and 4-nitrophthalonitrile (3.6 mg, 0.02 mmol) in DMSO (2.0 mL) for 72 h afforded a mixture of **3ya-Me** and **3ya-Et** (21.3 mg, 29%) (eluent: petroleum ether/ethyl acetate = 100/1 to 10/1): colorless oil. ^{1}H NMR (400 MHz, $CDCl_3$) δ **3ya-Me**: 7.54-6.77 (m, 13 H, Ar-H), 3.89 (d, *J* = 5.2 Hz, 1 H, CH), 3.42-3.37 (m, 1 H, CH), 3.34-3.18 (m, 2 H, CH_2), 2.65 (q, *J* = 7.6 Hz, 2 H, CH_2), 1.25 (t, *J* = 7.6 Hz, CH_3). **3ya-Et**: 7.36-6.76 (m, 13 H, Ar-H), 4.08 (d, *J* = 8.0 Hz, 1 H, CH), 3.58-3.44 (m, 2 H, CH × 2), 2.33 (s, 3 H, CH_3), 1.42 (d, *J* = 6.8 Hz, 3 H, CH_3). ^{13}C NMR (101 MHz, $CDCl_3$) δ **3ya-Me**: 157.3, 154.3, 136.3, 133.1, 130.7, 130.2, 130.1, 129.2, 129.1, 128.0, 119.3, 118.7, 112.0, 111.4, 48.4, 37.7, 28.5, 20.1, 15.7. **3ya-Et**: 156.9, 139.7, 134.8, 134.7, 129.2, 129.1, 128.7, 128.62, 128.55, 119.9, 118.09, 112.4, 111.9, 52.6, 40.9, 28.1, 27.5, 20.7. IR ν (neat, cm^{-1}) 3032, 2254. HRMS (ESI) m/z: [M]$^+$ Calcd for $C_{25}H_{22}N_2O$ 366.1727; found 366.1707.

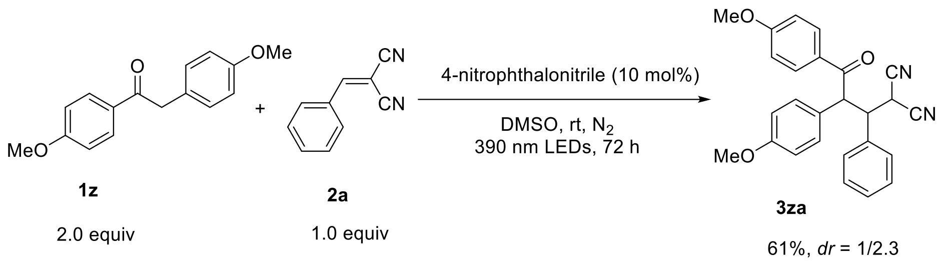

Following Typical Procedure 2, the reaction of **1z** (102.6 mg, 0.4 mmol), **2a** (30.7 mg, 0.2 mmol), and 4-nitrophthalonitrile (3.4 mg, 0.02 mmol) in DMSO (2.0 mL) for 72 h afforded **3za** (61%, *dr* = 1/2.3) (eluent: petroleum ether/ethyl acetate = 20/1) (compound **3z** is unstable and it would decompose back to **1z**, the yield was calculated by adding 1,3,5-trimethoxybenzene as internal standard): yellow oil. ^{1}H NMR (400 MHz, $CDCl_3$) δ 8.01-7.83 (m, 2 H, Ar-H), 7.58-7.43 (m, 3 H, Ar-H), 7.39-7.26 (m, 3.37 H, Ar-H), 7.02-6.85 (m, 2.78 H, Ar-H), 6.84-6.54 (m, 2 H, Ar-H), [5.30 (d, *J* = 11.6 Hz, 0.65 H) and 5.14 (d, *J* = 10.8 Hz, 0.26 H), CH], 4.73 (d, *J* = 4.8 Hz, 0.27 H of CH), 2.23-4.02 (m, 1.22 H, CH × 2), [3.83-3.77 (m, 5.22 H,) and 3.62 (s, 0.91 H), CH_3], 3.68 (d, *J* = 4.0 Hz, 0.71 H of CH). ^{13}C NMR (101 MHz, $CDCl_3$) δ major isomer: 193.9, 163.7, 160.0, 135.4, 130.8, 129.1, 128.9, 128.8, 128.2, 126.4, 115.4, 113.9, 111.9, 111.5, 55.4, 55.3, 53.4, 48.8, 28.2. minor isomer: 196.4, 164.1, 158.8, 134.8, 131.3, 129.63, 129.55 129.1, 128.6, 128.3, 127.0, 114.3, 114.0, 113.9, 112.0, 111.6, 55.4, 55.0, 54.4, 48.1, 27.4. IR ν (neat, cm^{-1}) 2257, 1669. HRMS (ESI) m/z: $[M + H]^+$ Calcd for $C_{26}H_{23}N_2O_3$ 411.1703; found 411.1716.

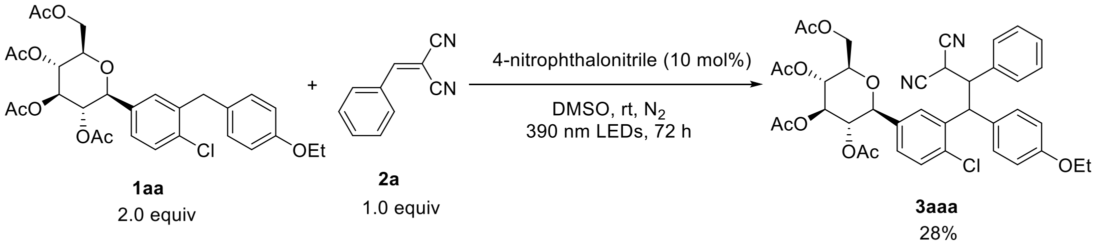

Following Typical Procedure 2, the reaction of **1aa** (230.8 mg, 0.4 mmol), **2a** (30.6 mg, 0.2 mmol), and 4-nitrophthalonitrile (3.4 mg, 0.02 mmol) in DMSO (2.0 mL) for 72 h afforded **3aaa** (40.1 mg, 28%, four diastereomers were observed but hard to determine the accurate ratio) (eluent: petroleum ether/ethyl acetate = 20/1 to 5/1): colorless oil. ^{1}H NMR (400 MHz, $CDCl_3$) see spectrum attached below. ^{13}C NMR (151 MHz, $CDCl_3$) see spectrum attached below. IR ν (neat, cm^{-1}) 2257, 1754. HRMS (ESI) m/z: $[M + Na]^+$ Calcd for $C_{39}H_{39}ClN_2O_{10}Na$ 753.2185; found 753.2177

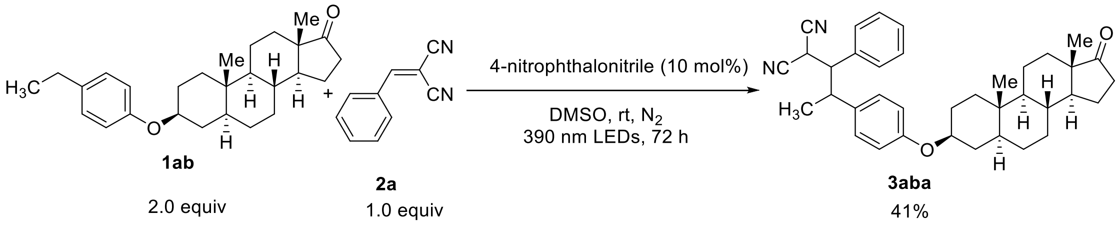

Following Typical Procedure 2, the reaction of **1ab** (158.1 mg, 0.4 mmol), **2a** (30.6 mg, 0.2 mmol), and 4-nitrophthalonitrile (3.6 mg, 0.02 mmol) in DMSO (2.0 mL) for 72 h afforded **3aba** (44.3 mg, 41%, four diastereomers were observed but hard to determine

the accurate ratio) (eluent: petroleum ether/ethyl acetate = 100/1 to 10/1): colorless oil. ^{1}H NMR (400 MHz, $CDCl_3$) δ see spectrum attached below. ^{13}C NMR (101 MHz, $CDCl_3$) δ see spectrum attached below. IR ν (neat, cm^{-1}) 2252, 1738. HRMS (ESI) m/z: $[M + H]^+$ Calcd for $C_{37}H_{45}N_2O_2$ 549.3476; found 549.3464.

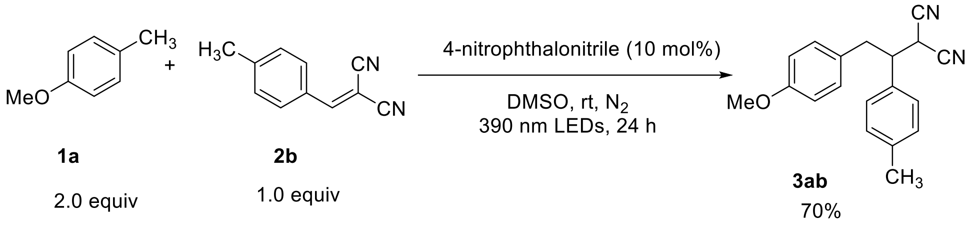

Following Typical Procedure 2, the reaction of **1a** (48.9 mg, 0.4 mmol), **2b** (33.3 mg, 0.2 mmol), and 4-nitrophthalonitrile (3.6 mg, 0.02 mmol) in DMSO (2.0 mL) afforded **3ab** (40.2 mg, 70%) (eluent: petroleum ether/ethyl acetate = 100/1 to 20/1): oil. ^{1}H NMR (400 MHz, $CDCl_3$) δ 7.31-7.20 (m, 4 H, Ar-H), 7.11 (d, *J* = 8.4 Hz, 2 H, Ar-H), 6.86 (d, *J* = 8.4 Hz, 2 H, Ar-H), 3.83 (d, *J* = 4.8 Hz, 1 H, CH), 3.80 (s, 3 H, CH_3), 3.44-3.34 (m, 1 H, CH), 3.22-3.15 (m, 2 H, CH_2), 2.37 (s, 3 H, CH_3). ^{13}C NMR (101 MHz, $CDCl_3$) δ 158.8, 138.8, 133.4, 129.9, 129.8, 128.6, 127.8, 114.4, 112.2, 111.5, 55.2, 48.1, 37.6, 28.5, 21.1. IR ν (neat, cm^{-1}) 3006, 2254. HRMS (ESI) m/z: $[M]^+$ Calcd for $C_{19}H_{18}N_2O$ 290.1414; found 290.1419.

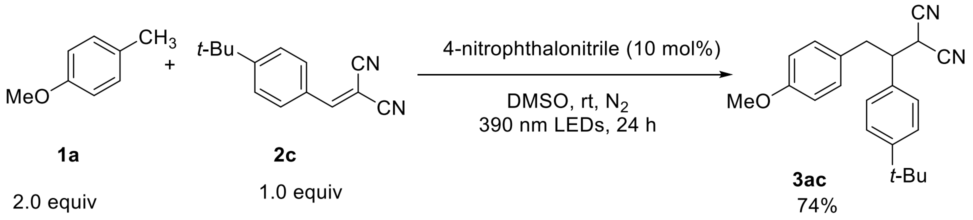

Following Typical Procedure 2, the reaction of **1a** (48.9 mg, 0.4 mmol), **2b** (42.3 mg, 0.2 mmol), and 4-nitrophthalonitrile (3.6 mg, 0.02 mmol) in DMSO (2.0 mL) afforded **3ac** (49.4 mg, 74%) (eluent: petroleum ether/ethyl acetate = 100/1 to 20/1): oil. ^{1}H NMR (400 MHz, $CDCl_3$) δ 7.45 (d, *J* = 8.4 Hz, 2 H, Ar-H), 7.35 (d, *J* = 8.0 Hz, 2 H, Ar-H), 7.14 (d, *J* = 8.4 Hz, 2 H, Ar-H), 6.88 (d, *J* = 8.4 Hz, 2 H, Ar-H), 3.87-3.83 (m, 1 H, CH), 3.81 (s, 3 H, CH_3), 3.46-3.36 (m, 1 H, CH), 3.20 (d, *J* = 8.0 Hz, 2 H, CH_2), 1.35 (s, 9 H, $CH_3 \times 3$). ^{13}C NMR (101 MHz, $CDCl_3$) δ 158.9, 151.9, 133.4, 129.9, 128.7, 127.6, 126.0, 114.5, 112.2, 111.5, 55.2, 48.0, 37.5, 34.6, 31.2, 28.3. IR ν (neat, cm^{-1}) 3031, 2254. HRMS (ESI) m/z: $[M + K]^+$ Calcd for $C_{22}H_{24}N_2OK$ 371.1520; found 371.1502.

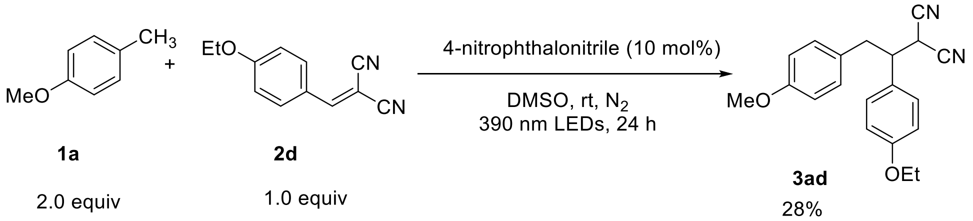

Following Typical Procedure 2, the reaction of **1a** (48.6 mg, 0.4 mmol), **2d** (39.3 mg, 0.2 mmol), and 4-nitrophthalonitrile (3.8 mg, 0.02 mmol) in DMSO (2.0 mL) afforded **3ad** (17.9 mg, 28%) (eluent: petroleum ether/ethyl acetate = 100/1 to 20/1): oil. ^{1}H

NMR (400 MHz, $CDCl_3$) δ 7.31-7.27 (m, 2 H, Ar-H), 7.13-7.07 (m, 2 H, Ar-H), 6.94-6.90 (m, 2 H, Ar-H), 6.88-6.83 (m, 2 H, Ar-H), 4.04 (q, *J* = 7.2 Hz, 2 H, CH_2), 3.83-3.78 (m, 4 H, CH and CH_3), 3.40-3.33 (m, 1 H, CH), 3.17 (d, *J* = 8.0 Hz, 2 H, CH_2), 1.42 (t, *J* = 7.0 Hz, 3 H, CH_3). ^{13}C NMR (101 MHz, $CDCl_3$) δ 159.3, 158.9, 129.9, 129.1, 128.7, 128.2, 114.9, 114.5, 112.3, 111.5, 63.5, 55.3, 47.9, 37.7, 28.6, 14.8. IR ν (neat, cm^{-1}) 2251. HRMS (ESI) m/z: $[M + K]^+$ Calcd for $C_{20}H_{20}N_2O_2K$ 359.1156; found 359.1174.

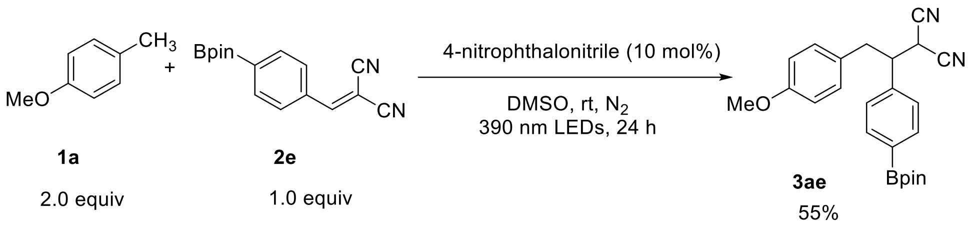

Following Typical Procedure 2, the reaction of **1a** (48.8 mg, 0.4 mmol), **2e** (56.2 mg, 0.2 mmol), and 4-nitrophthalonitrile (3.4 mg, 0.02 mmol) in DMSO (2.0 mL) afforded **3ae** (44.7 mg, 55%) (eluent: petroleum ether/ethyl acetate = 50/1 to 20/1): oil. ^1H NMR (400 MHz, $CDCl_3$) δ 7.85 (d, *J* = 8.0 Hz, 2 H, Ar-H), 7.37 (d, *J* = 8.0 Hz, 2 H, Ar-H), 7.08 (d, *J* = 8.4 Hz, 2 H, Ar-H), 6.84 (d, *J* = 8.8 Hz, 2 H, Ar-H), 3.86 (d, *J* = 5.2 Hz, 1 H, CH), 3.79 (s, 3 H, CH_3), 3.46-3.38 (m, 1 H, CH), 3.26-3.14 (m, 2 H, CH_2), 1.35 (s, 12 H, $CH_3 \times 4$). ^{13}C NMR (101 MHz, $CDCl_3$) δ 158.8, 139.3, 135.5, 129.9, 128.3, 127.3, 114.4, 112.0, 111.4, 84.0, 55.2, 48.5, 37.5, 28.2, 24.8. IR ν (neat, cm^{-1}) 2255, 2231. HRMS (ESI) m/z: $[M + K]^+$ Calcd for $C_{24}H_{27}BN_2O_3K$ 441.1746; found 441.1748.

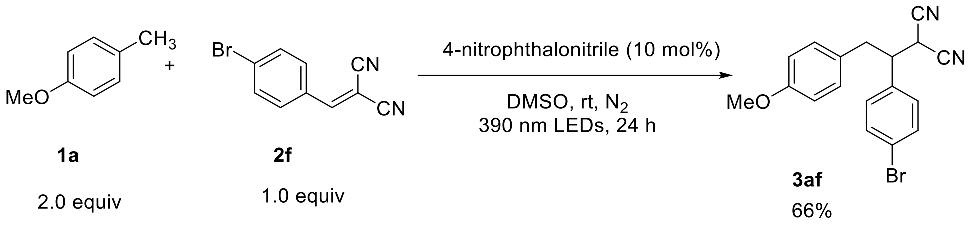

Following Typical Procedure 2, the reaction of **1a** (48.7 mg, 0.4 mmol), **2f** (46.9 mg, 0.2 mmol), and 4-nitrophthalonitrile (3.6 mg, 0.02 mmol) in DMSO (2.0 mL) afforded **3af** (47.4 mg, 66%) (eluent: petroleum ether/ethyl acetate = 100/1 to 20/1): oil. ^1H NMR (400 MHz, $CDCl_3$) δ 7.56 (d, *J* = 8.4 Hz, 2 H, Ar-H), 7.27 (d, *J* = 8.4 Hz, 2 H, Ar-H), 7.09 (d, *J* = 8.8 Hz, 2 H, Ar-H), 6.86 (d, *J* = 8.4 Hz, 2 H, Ar-H), 3.84 (d, *J* = 4.8 Hz, 1 H, CH), 3.80 (s, 3 H, CH_3), 3.43-3.35 (m, 1 H, CH), 3.23-3.12 (m, 2 H, CH_2). ^{13}C NMR (101 MHz, $CDCl_3$) δ 159.0, 135.3, 132.3, 129.9, 129.7, 127.9, 123.1, 114.5, 111.9, 111.2, 55.2, 47.8, 37.4, 28.1. IR ν (neat, cm^{-1}) 2253. HRMS (ESI) m/z: $[M + Na]^+$ Calcd for $C_{18}H_{15}BrN_2ONa$ 377.0260; found 377.0260.

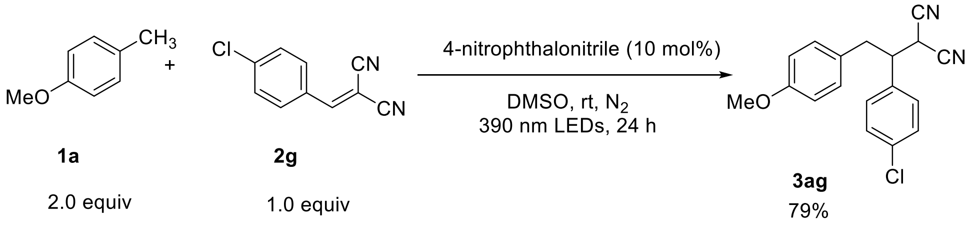

Following Typical Procedure 2, the reaction of **1a** (48.9 mg, 0.4 mmol), **2g** (38.0 mg,

0.2 mmol), and 4-nitrophthalonitrile (3.4 mg, 0.02 mmol) in DMSO (2.0 mL) afforded **3ag** (49.7 mg, 79%) (eluent: petroleum ether/ethyl acetate = 50/1 to 20/1): oil. ^{1}H NMR (400 MHz, $CDCl_3$) δ 7.43-7.38 (m, 2 H, Ar-H), 7.36-7.30 (m, 2 H, Ar-H), 7.12-7.06 (m, 2 H, Ar-H), 6.89-6.83 (m, 2 H, Ar-H), 3.84 (d, *J* = 5.2 Hz, 1 H, CH), 3.80 (s, 3 H, CH_3), 3.44-3.35 (m, 1 H, CH), 3.22-3.11 (m, 2 H, CH_2). ^{13}C NMR (101 MHz, $CDCl_3$) δ 159.1, 135.1, 134.8, 129.9, 129.4, 128.0, 114.6, 111.9, 111.2, 55.3, 47.9, 37.6, 28.2. IR ν (neat, cm^{-1}) 2257. HRMS (ESI) m/z: $[M + H]^+$ Calcd for $C_{18}H_{16}ClN_2O$ 311.0946; found 311.0928.

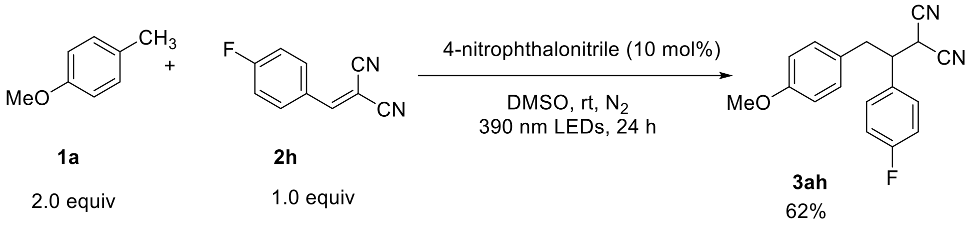

Following Typical Procedure 2, the reaction of **1a** (48.8 mg, 0.4 mmol), **2h** (34.6 mg, 0.2 mmol), and 4-nitrophthalonitrile (3.3 mg, 0.02 mmol) in DMSO (2.0 mL) afforded **3ah** (36.4 mg, 62%) (eluent: petroleum ether/ethyl acetate = 100/1 to 20/1): colorless oil. ^{1}H NMR (400 MHz, $CDCl_3$) δ 7.41-7.34 (m, 2 H, Ar-H), 7.16-7.07 (m, 4 H, Ar-H), 6.89-6.84 (m, 2 H, Ar-H), 3.84 (d, *J* = 5.2 Hz, 1 H, CH), 3.80 (s, 3 H, CH_3), 3.44-3.37 (m, 1 H, CH), 3.23-3.11 (m, 2 H, CH_2). ^{13}C NMR (101 MHz, $CDCl_3$) δ 162.9 (d, ^1J = 249.5 Hz), 159.0, 132.2, 129.9, 129.8 (d, ^3J = 8.1 Hz), 128.1, 116.1 (d, ^2J = 21.2 Hz), 114.5, 112.0, 111.3, 55.2, 47.7, 37.7, 28.4. ^{19}F NMR (376 MHz, $CDCl_3$) δ -112.2. IR ν (neat, cm^{-1}) 3006, 2255. HRMS (ESI) m/z: $[M]^+$ Calcd for $C_{18}H_{15}FN_2O$ 294.1163; found 294.1174.

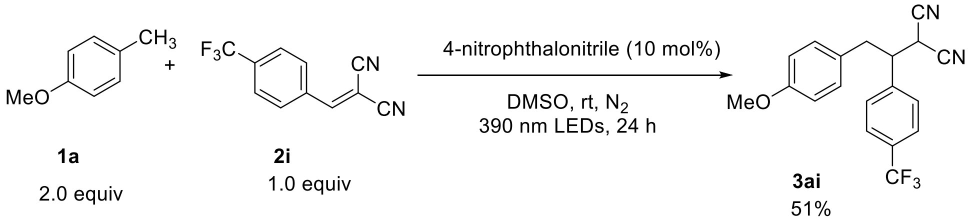

Following Typical Procedure 2, the reaction of **1a** (48.9 mg, 0.4 mmol), **2i** (44.3 mg, 0.2 mmol), and 4-nitrophthalonitrile (3.7 mg, 0.02 mmol) in DMSO (2.0 mL) afforded **3ai** (34.7 mg, 51%) (eluent: petroleum ether/ethyl acetate = 100/1 to 20/1): colorless oil. ^{1}H NMR (400 MHz, $CDCl_3$) δ 7.70 (d, *J* = 8.4 Hz, 2 H, Ar-H), 7.53 (d, *J* = 8.4 Hz, 2 H, Ar-H), 7.11 (d, *J* = 8.8 Hz, 2 H, Ar-H), 6.87 (d, *J* = 8.4 Hz, 2 H, Ar-H), 3.89 (d, *J* = 5.2 Hz, 1 H, CH), 3.80 (s, 3 H, CH_3), 3.56-3.44 (m, 1 H, CH), 3.28-3.15 (m, 2 H, CH_2). ^{13}C NMR (151 MHz, $CDCl_3$) δ 159.2, 140.3, 131.3 (q, ^2J = 33.2 Hz), 129.9, 128.6, 127.7, 126.2 (q, ^3J = 4.5 Hz), 123.7 (q, ^1J = 273.3 Hz), 114.7, 111.8, 111.1, 55.3, 48.2, 37.5, 28.0. ^{19}F NMR (376 MHz, $CDCl_3$) δ -63.0. IR ν (neat, cm^{-1}) 2225. HRMS (ESI) m/z: $[M + H]^+$ Calcd for $C_{19}H_{16}F_3N_2O$ 345.1209; found 345.1212.

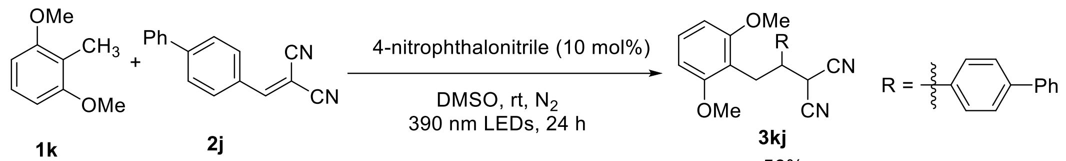

Following Typical Procedure 2, the reaction of **1k** (61.0 mg, 0.4 mmol), **2j** (45.7 mg, 0.2 mmol), and 4-nitrophthalonitrile (3.4 mg, 0.02 mmol) in DMSO (2.0 mL) afforded **3kj** (42.3 mg, 56%) (eluent: petroleum ether/ethyl acetate = 100/1 to 10/1): colorless oil. ^{1}H NMR (400 MHz, $CDCl_3$) δ 7.66-7.59 (m, 4 H, Ar-H), 7.57-7.52 (m, 2 H, Ar-H), 7.49-7.43 (m, 2 H, Ar-H), 7.40-7.34 (m, 1 H, Ar-H), 7.28-7.22 (m, 1 H, Ar-H), 6.59 (d, J = 8.4 Hz, 2 H, Ar-H), 3.91 (d, J = 3.6 Hz, 1 H, CH), 3.84 (s, 6 H, CH_3 × 2), 3.55-3.46 (m, 2 H, CH_2), 3.20-3.11 (m, 1 H, CH). ^{13}C NMR (101 MHz, $CDCl_3$) δ 158.4, 141.5, 140.5, 136.7, 128.8, 128.6, 127.4, 127.1, 113.4, 113.1, 112.0, 103.7, 55.5, 46.1, 28.5, 26.1. IR ν (neat, cm^{-1}) 2225. HRMS (ESI) m/z: $[M + Li]^+$ Calcd for $C_{25}H_{22}N_2O_2Li$ 389.1836; found 389.1843.

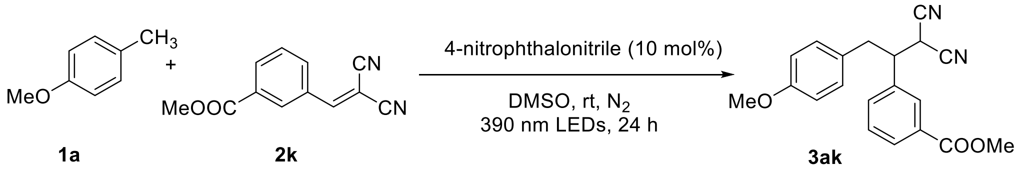

Following Typical Procedure 2, the reaction of **1a** (49.0 mg, 0.4 mmol), **2k** (42.4 mg, 0.2 mmol), and 4-nitrophthalonitrile (3.4 mg, 0.02 mmol) in DMSO (2.0 mL) afforded **3ak** (46.6 mg, 70%) (eluent: petroleum ether/ethyl acetate = 50/1 to 20/1 to 10/1): colorless oil. ^{1}H NMR (400 MHz, $CDCl_3$) δ 8.09-8.03 (m, 2 H, Ar-H), 7.62-7.57 (m, 1 H, Ar-H), 7.54-7.47 (m, 1 H, Ar-H), 7.12-7.05 (m, 2 H, Ar-H), 6.89-6.82 (m, 2 H, Ar-H), 3.94 (s, 3 H, CH_3), 3.89 (d, J = 5.6 Hz, 1 H, CH), 3.79 (s, 3 H, CH_3), 3.53-3.45 (m, 1 H, CH), 3.29-3.15 (m, 2 H, CH_2). ^{13}C NMR (101 MHz, $CDCl_3$) δ 166.4, 159.0, 136.8, 132.4, 131.1, 130.2, 129.9, 129.3, 129.2, 128.0, 114.5, 111.9, 111.2, 55.3, 52.4, 48.3, 37.6, 28.2. IR ν (neat, cm^{-1}) 2254, 1721. HRMS (ESI) m/z: $[M + Na]^+$ Calcd for $C_{20}H_{18}N_2O_3Na$ 357.1210; found 357.1224.

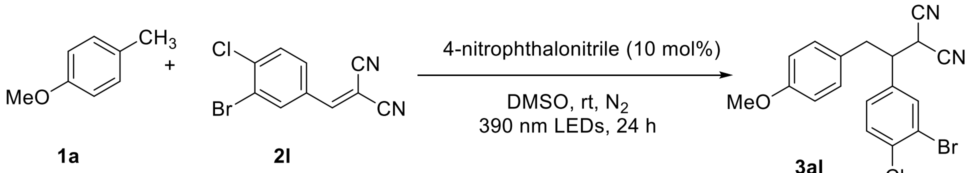

Following Typical Procedure 2, the reaction of **1a** (49.0 mg, 0.4 mmol), **2l** (53.3 mg, 0.2 mmol), and 4-nitrophthalonitrile (3.3 mg, 0.02 mmol) in DMSO (2.0 mL) afforded **3al** (59.0 mg, 76%) (eluent: petroleum ether/ethyl acetate = 100/1 to 20/1): colorless oil. ^{1}H NMR (400 MHz, $CDCl_3$) δ 7.64 (d, J = 2.0 Hz, 1 H, Ar-H), 7.50 (d, J = 8.4 Hz, 1 H, Ar-H), 7.32-7.27 (m, 1 H, Ar-H), 7.09 (d, J = 8.8 Hz, 2 H, Ar-H), 6.87 (d, J = 8.8 Hz, 2 H, Ar-H), 3.85 (d, J = 5.2 Hz, 1 H, CH), 3.80 (s, 3 H, CH_3), 3.41-3.33 (m, 1 H, CH), 3.21-3.11 (m, 2 H, CH_2). ^{13}C NMR (101 MHz, $CDCl_3$) δ 159.1, 136.6, 135.4, 133.3, 130.9, 129.9, 128.0, 127.5, 123.3, 114.7, 111.7, 111.0, 55.3, 47.4, 37.5, 28.0. IR

ν (neat, cm^{-1}) 3003, 2253. HRMS (ESI) m/z: $[M + H]^+$ Calcd for $C_{18}H_{16}BrClN_2O$ 390.0129; found 390.0120.

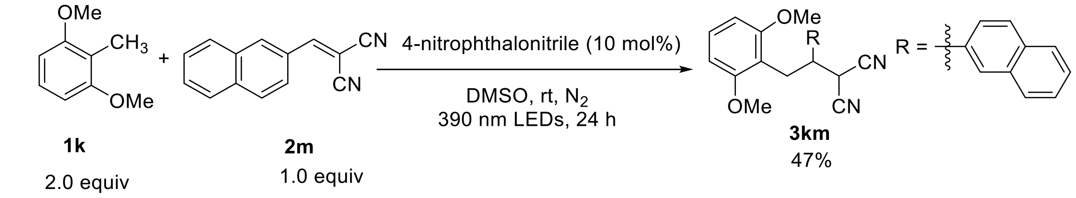

Following Typical Procedure 2, the reaction of **1k** (60.5 mg, 0.4 mmol), **2m** (40.7 mg, 0.2 mmol), and 4-nitrophthalonitrile (3.3 mg, 0.02 mmol) in DMSO (2.0 mL) afforded **3km** (33.7 mg, 47%) (eluent: petroleum ether/ethyl acetate = 100/1 to 20/1): colorless oil. ^1H NMR (400 MHz, $CDCl_3$) δ 7.96-7.84 (m, 4 H, Ar-H), 7.64-7.58 (m, 1 H, Ar-H), 7.56-7.49 (m, 2 H, Ar-H), 7.27-7.21 (m, 1 H, Ar-H), 6.58 (d, J = 8.4 Hz, 2 H, Ar-H), 3.98 (d, J = 3.2 Hz, 1 H, CH), 3.83 (s, 6 H, CH_3 × 2), 3.71-3.52 (m, 2 H, CH_2), 3.29-3.22 (m, 1 H, CH). ^{13}C NMR (101 MHz, $CDCl_3$) δ 158.4, 135.2, 133.2, 133.2, 128.8, 128.5, 128.1, 127.6, 127.5, 126.4, 125.7, 113.4, 113.0, 112.1, 103.6, 55.5, 46.4, 28.6, 26.2. IR ν (neat, cm^{-1}) 3003, 2253. HRMS (ESI) m/z: $[M + H]^+$ Calcd for $C_{23}H_{21}N_2O_2$ 357.1598; found 357.1583.

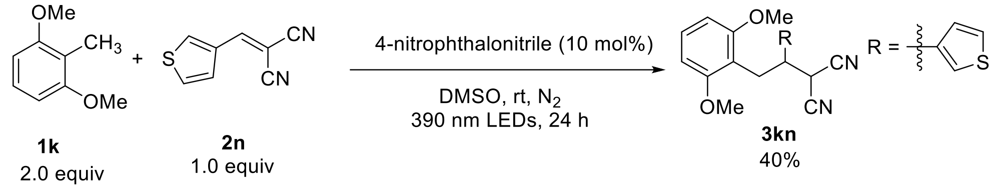

Following Typical Procedure 2, the reaction of **1k** (61.1 mg, 0.4 mmol), **2n** (32.4 mg, 0.2 mmol), and 4-nitrophthalonitrile (3.5 mg, 0.02 mmol) in DMSO (2.0 mL) afforded **3kn** (25.2 mg, 40%) (eluent: petroleum ether/ethyl acetate = 100/1 to 20/1): colorless oil. ^1H NMR (400 MHz, $CDCl_3$) δ 7.45-7.35 (m, 2 H, Ar-H), 7.29-7.21 (m, 2 H, Ar-H), 6.58 (d, J = 8.4 Hz, 2 H, Ar-H), 3.89-3.80 (m, 7 H, CH and CH_3 × 2), 3.69-3.59 (m, 1 H, CH_2), 3.45-3.34 (m, 1 H, CH_2), 3.21-3.11 (m, 1 H, CH). ^{13}C NMR (101 MHz, $CDCl_3$) δ 158.4, 138.4, 128.8, 126.9, 126.2, 123.4, 113.2, 112.0, 103.6, 55.5, 42.1, 28.4, 26.4. IR ν (neat, cm^{-1}) 3004, 2252. HRMS (ESI) m/z: $[M + H]^+$ Calcd for $C_{17}H_{17}N_2O_2S$ 313.1005; found 313.1011.

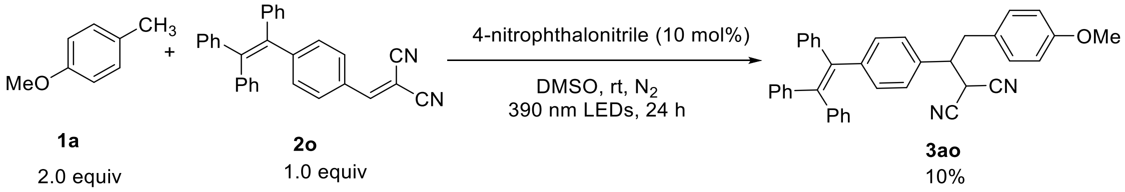

Following Typical Procedure 2, the reaction of **1a** (48.4 mg, 0.4 mmol), **2o** (81.6 mg, 0.2 mmol), and 4-nitrophthalonitrile (3.4 mg, 0.02 mmol) in DMSO (2.0 mL) afforded **3ao** (10.5 mg, 10%) (eluent: petroleum ether/ethyl acetate = 100/1 to 20/1): yellow oil. ^1H NMR (400 MHz, $CDCl_3$) δ 7.15-6.97 (m, 21 H, Ar-H), 6.84 (d, J = 8.8 Hz, 2 H, Ar-H), 3.83-3.78 (m, 4 H, CH and CH_3), 3.35-3.29 (m, 1 H, CH), 3.23-3.07 (m, 2 H, CH_2). ^{13}C NMR (151 MHz, $CDCl_3$) δ 158.9, 144.5, 143.4, 143.3, 143.2, 141.7, 130.0, 134.5, 132.1, 131.9, 131.27, 131.25, 130.0, 128.4, 128.4, 127.74, 127.69, 127.65, 127.3, 126.61, 126.57, 114.3, 112.1, 111.5, 55.3, 48.2, 37.7, 28.3. IR ν (neat, cm^{-1}) 3054, 2255.

HRMS (ESI) m/z: [M]+ Calcd for $C_{38}H_{30}N_2O$ 530.2353; found 530.2333.

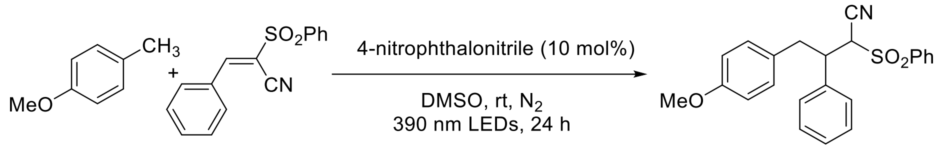

1a 2.0 equiv **2p** 1.0 equiv **3ap** 49%, *dr* = 1/1.1

Following Typical Procedure 2, the reaction of **1a** (50 μL, *d* = 0.969 g/mL, 48.5 mg, 0.4 mmol), **2p** (53.8 mg, 0.2 mmol), and 4-nitrophthalonitrile (3.5 mg, 0.02 mmol) in DMSO (2.0 mL) afforded **3ap** (38.4 mg, 49%, *dr* = 1/1.1) (eluent: petroleum ether/ethyl acetate = 50/1 to 20/1): colorless oil. ^{1}H NMR (400 MHz, $CDCl_3$) δ 8.13-7.99 (m, 1 H, Ar-H), 7.81-7.09 (m, 10 H, Ar-H), 6.97-6.63 (m, 3 H, Ar-H), 4.20-4.09 (m, 1 H, CH), 4.02-3.52 (m, 5 H, CH_2 and CH_3), 3.23-3.07 (m, 1 H, CH). ^{13}C NMR (101 MHz, $CDCl_3$) δ major isomer: 158.8, 137.1, 136.4, 134.5, 129.9, 129.7, 129.3, 128.6, 128.2, 127.9, 114.4, 113.5, 61.0, 55.3, 45.6, 40.5. minor isomer: 158.1, 138.8, 136.8, 135.2, 130.08, 129.7, 129.0, 128.73, 128.66, 128.0, 113.6, 113.2, 62.8, 55.1, 44.3, 37.3. IR ν (neat, cm^{-1}) 3032, 2245. HRMS (ESI) m/z: [M + Na]+ Calcd for $C_{23}H_{21}NO_3SNa$ 414.1134; found 414.1145.

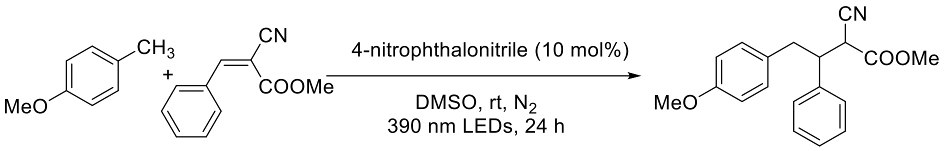

1a 2.0 equiv **2q** 1.0 equiv **3aq** 57%, dr = 1/2

Following Typical Procedure 2, the reaction of **1a** (48.9 mg, 0.4 mmol), **2q** (37.3 mg, 0.2 mmol), and 4-nitrophthalonitrile (3.5 mg, 0.02 mmol) in DMSO (2.0 mL) afforded **3aq** (35.2 mg, 57%, *dr* = 1/2) (eluent: petroleum ether/ethyl acetate = 100/1 to 20/1): colorless oil. ^{1}H NMR (400 MHz, $CDCl_3$) δ 7.89-7.2 3 (m, 5 H, Ar-H), [7.22-7.06 (m, 1.35 H), 7.04-6.99 (m, 0.67 H), Ar-H], [6.89-6.84 (m, 1.32 H), 6.79-6.73 (m, 0.66 H), Ar-H], [3.79 (s, 2 H), 3.75 (s, 1 H), CH_3], 3.75-3.68 (m, 1 H, CH), 3.67-3.59 (m, 1 H, CH), [3.583 (s, 1 H), 3.576 (s, 2 H), CH_3], 3.29-3.06 (m, 2 H, CH_2). ^{13}C NMR (101 MHz, $CDCl_3$) δ major isomer: 166.0, 158.6, 138.4, 130.0, 129.7, 128.7, 127.9, 127.8, 115.2, 114.3, 55.2, 53.2, 47.5, 42.6, 38.6. minor isomer: 165.5, 158.3, 139.0, 130.3, 129.6, 128.8, 128.1, 127.9, 115.8, 113.7, 55.1, 53.3, 47.7, 43.7, 37.6. IR ν (neat, cm^{-1}) 2248, 1746. HRMS (ESI) m/z: [M + Na]+ Calcd for $C_{19}H_{19}NO_3Na$ 332.1257; found 332.1273.

Mechanistic studies for the arene activation

1 The light on/off experiment

The light on/off experiments were conducted by monitoring the product formation using 2-(4-fluorobenzylidene)malononitrile (**2h**) as a substrate. The reaction was proceeded smoothly under irradiation of blue LED and ceased when the light was turned off, indicating that the light plays a crucial role for the transformation.

Supplementary Table 5. The light on/off experiment

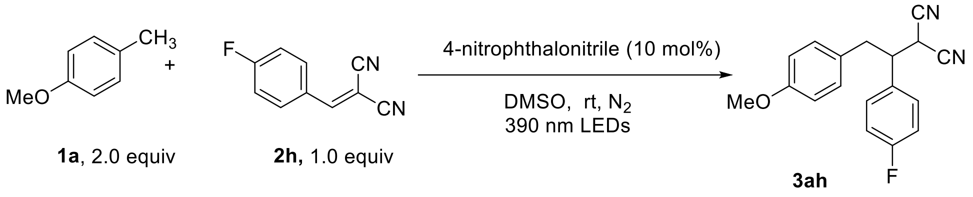

Time scale	Light on/off	Yield[a] / %
0-10 h	On	37
10-15 h	Off	37
15-20 h	On	48
20-25 h	Off	48
25-30 h	On	62
30-35 h	Off	63
35-40 h	On	74
45-50 h	Off	74
35-40 h	On	83
40-45 h	Off	83

[a] All yields were based on crude ^{19}F NMR.

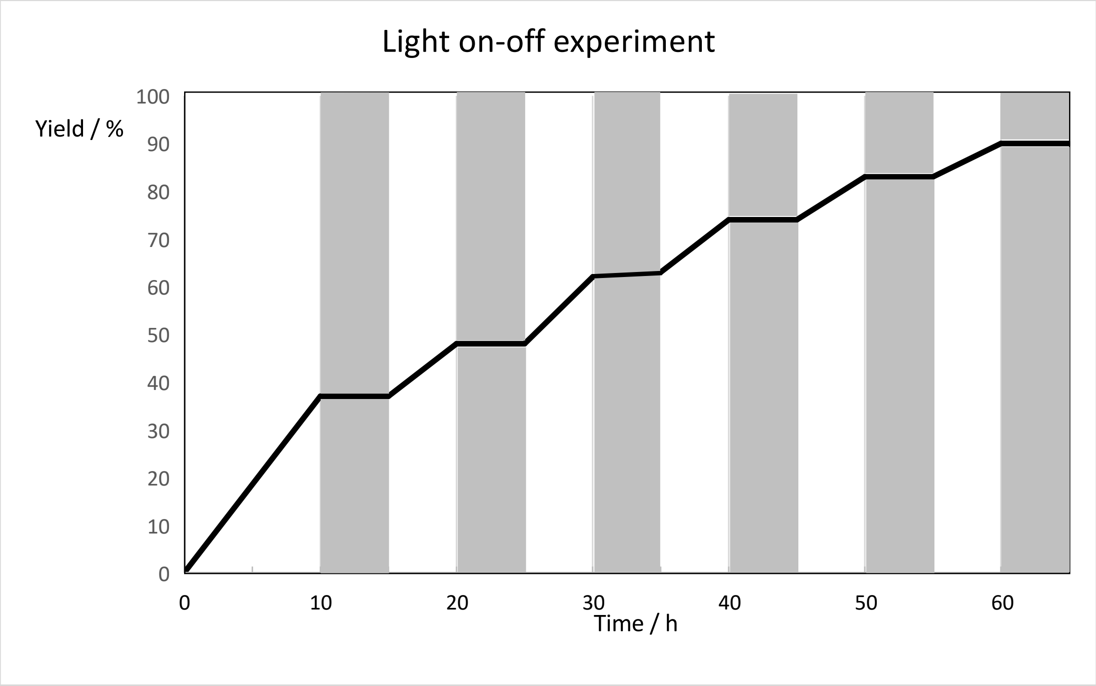

2. The radical-captured experiment

The reaction of **1a** with TEMPO in DMSO under N_2 would produce the radical captured product **1a-TEMPO**, which could be detected by high resolution mass spectroscopy. HRMS (ESI) m/z: $[M + H]^+$ Calcd for $C_{17}H_{28}NO_2$ 278.2115; found 278.2105.

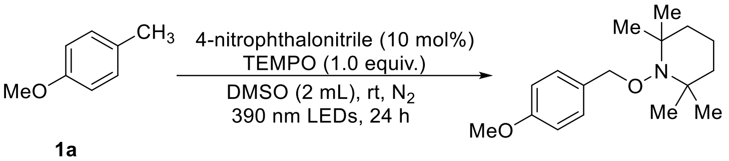

1a

1a-TEMPO

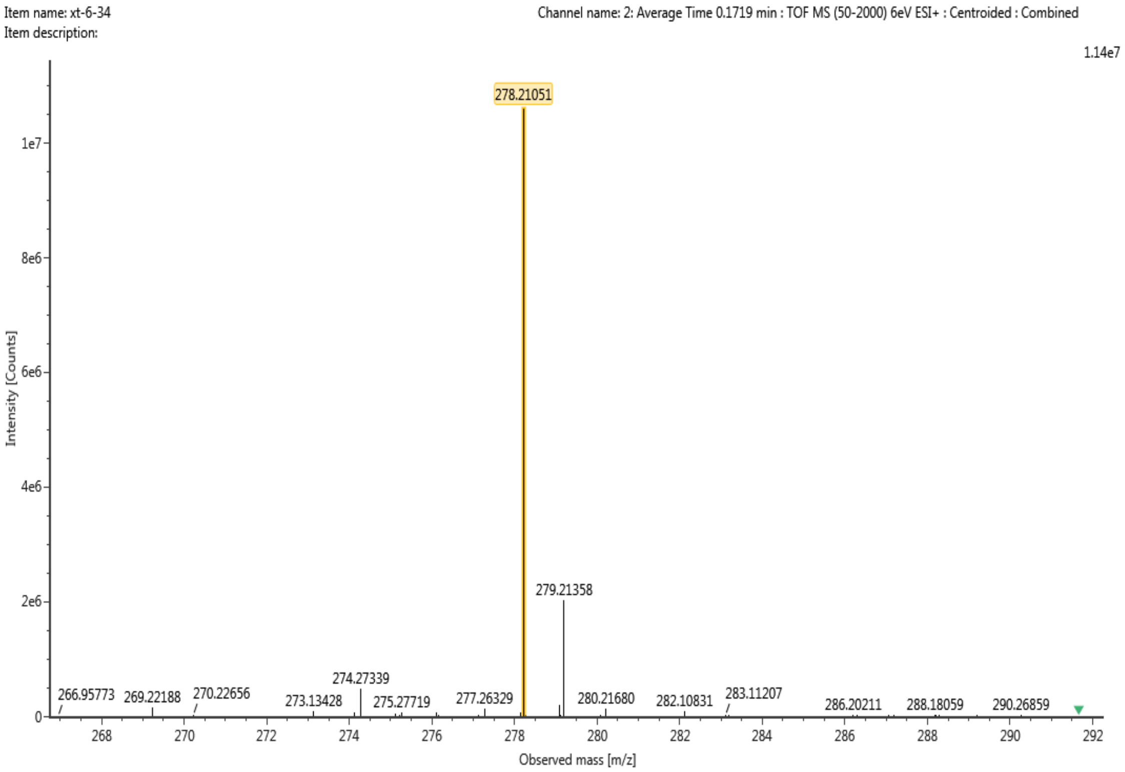

Supplementary Figure 5: **The HRMS spectrum of 1a-TEMPO**. HRMS (ESI) m/z: $[M + H]^+$ Calcd for $C_{17}H_{28}NO_2$ 278.2115; found 278.2105.

The reaction of **1a, 2a** with TEMPO in DMSO under N_2 would produce the radical captured product **1a-TEMPO**, which could be detected by high resolution mass spectroscopy. HRMS (ESI) m/z: $[M + H]^+$ Calcd for $C_{17}H_{28}NO_2$ 278.2115; found 278.2111.

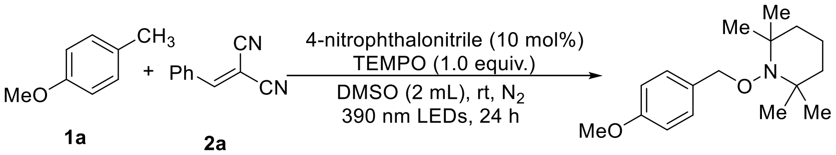

1a 2a

1a-TEMPO

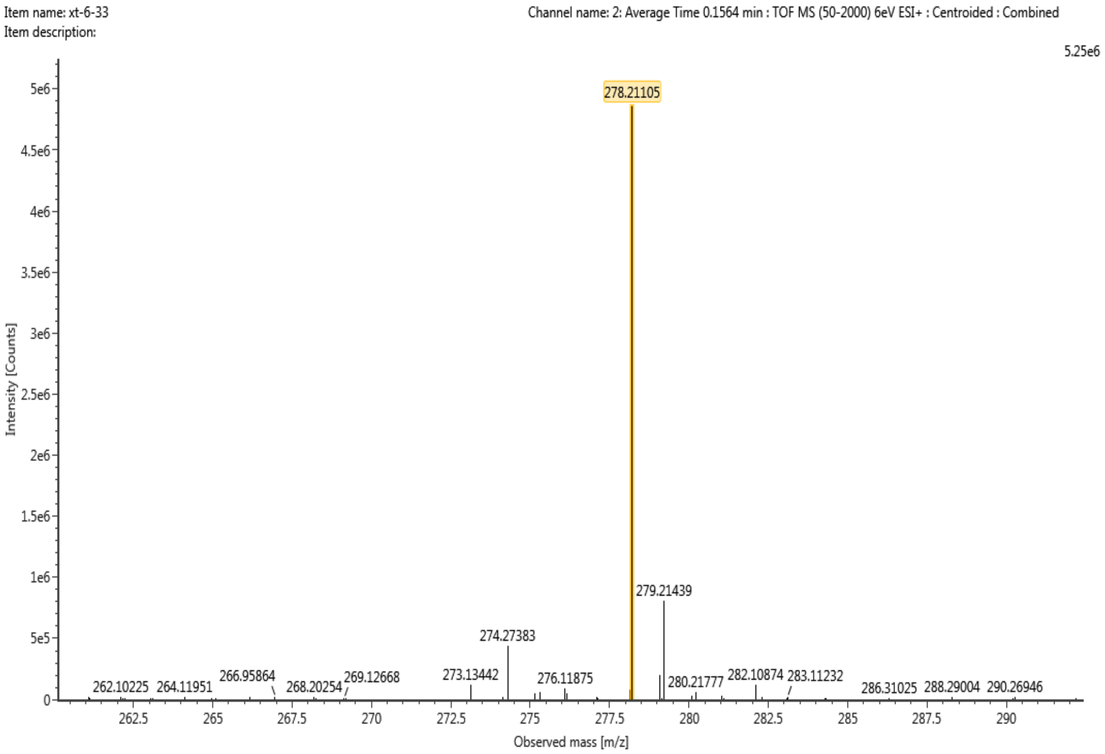

Supplementary Figure 6: **The HRMS spectrum of 1a-TEMPO.** HRMS (ESI) m/z: $[M + H]^+$ Calcd for $C_{17}H_{28}NO_2$ 278.2115; found 278.2111.

3. KIE experiment

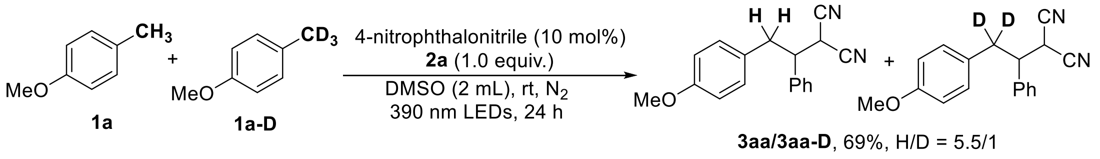

To a 4 mL reaction tube were added **1a** (24.3 mg, 0.2 mmol), **1a-D** (25.1 mg, 0.2 mmol), **2a** (30.9 mg, 0.2 mmol), and 4-nitrophthalonitrile (3.3 mg, 0.02 mmol) in DMSO (2.0 mL) in an N_2 glovebox. The reaction tube was transferred out of glovebox. Under irradiation at 390 nm LEDs, the resulting mixture was stirred for 24 hours at rt. Evaporation and flash chromatography on silica gel afforded **3aa**/**3aa**-D (38.1 mg, 69%) (eluent: petroleum ether/ethyl acetate = 50/1 to 20/1). The KIE was determined to be k_H/k_D = 5.5/1 by the analysis of the ^{1}H NMR (400 MHz, $CDCl_3$) 7.48-7.35 (m, 5 H, Ar-H), 7.11 (d, J = 8.8 Hz, 2 H, Ar-H), 6.86 (d, J = 8.8 Hz, 2 H, Ar-H), 3.86 (d, J = 4.8 Hz, 1 H, CH), 3.80 (s, 3 H, CH_3), 3.46-3.38 (m, 1 H, CH), 3.27-3.15 (m, 1.69 H, CH_2).

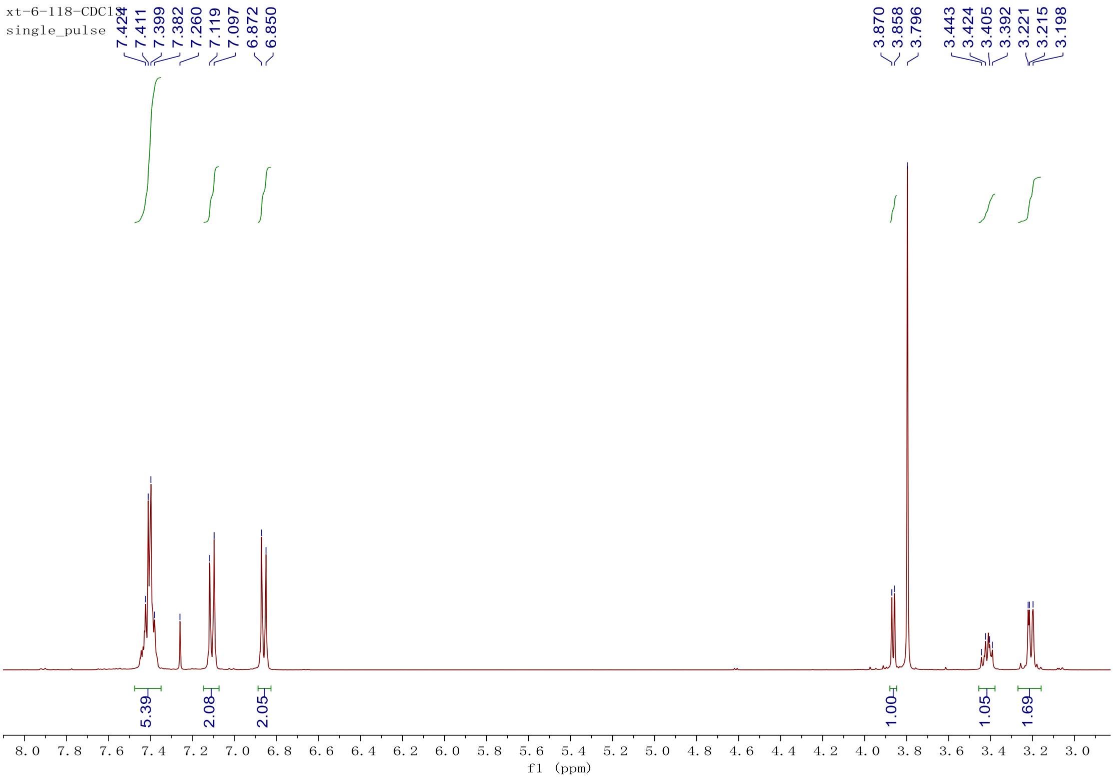

Supplementary Figure 7: The ^{1}H NMR spectrum of 3aa/3aa-D. The KIE was determined to be k_H/k_D = 5.5/1 by the analysis of the ^{1}H NMR (400 MHz, $CDCl_3$).

5. Cyclic Voltammetry Measurements

For all cyclic voltammetry (CV) measurements, a glassy carbon disk electrode (diameter 3 mm) was used as the working electrode. A silver wire coated with AgCl immersed in a 3.5 M aqueous solution of KCl and separated from the analyte by a fritted glass disk was employed as the reference electrode. A Pt wire counter-electrode completed the electrochemical setup. The scan rate was 0.1 v/s.

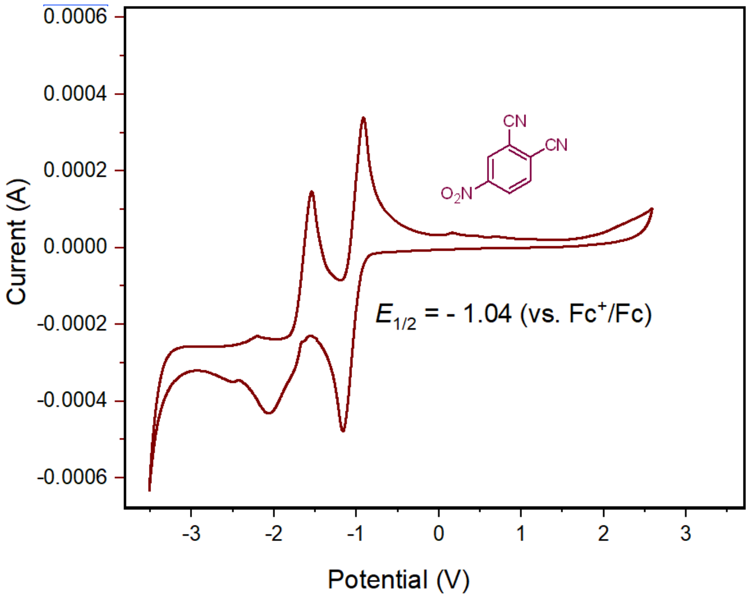

Supplementary Figure 8: Cyclic voltammogram 1. Cyclic voltammogram for 4-

nitrophthalonitrile [0.002 M] in [0.01 M] $TBAPF_6$ in CH_3CN. Measurement started by reduction from 0 to 2.5 V, followed by oxidation from 2.5 to -3.0 V, and finishing at 0 V. Glassy carbon electrode S27 working electrode, Ag/AgCl (KCl 3.5 M) reference electrode, Pt wire auxiliary electrode.

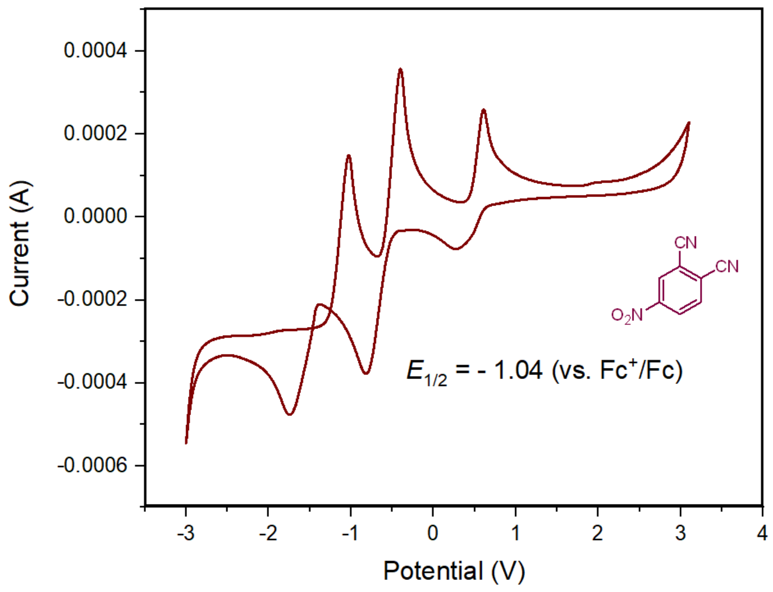

Supplementary Figure 9: Cyclic voltammogram 2. Cyclic voltammogram for 4-nitrophthalonitrile [0.002 M] and ferrocene [0.002 M] in [0.01 M] $TBAPF_6$ in CH_3CN. Measurement started by reduction from 0 to 2.5 V, followed by oxidation from 2.5 to -3.0 V, and finishing at 0 V. Glassy carbon electrode S27 working electrode, Ag/AgCl (KCl 3.5 M) reference electrode, Pt wire auxiliary electrode.

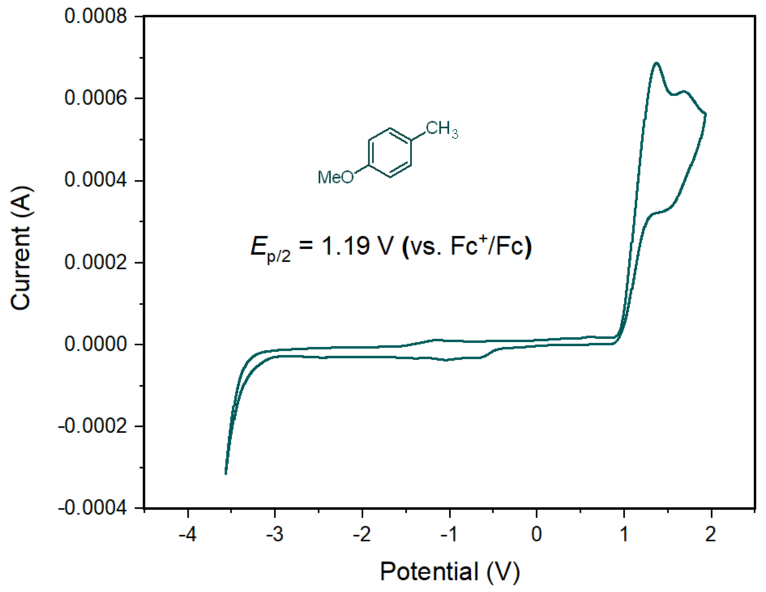

Supplementary Figure 10: Cyclic voltammogram 3. Cyclic voltammogram for 4-methylanisole [0.002 M] in [0.01 M] $TBAPF_6$ in CH_3CN. Measurement started by reduction from 0 to 2.5 V, followed by oxidation from 2.5 to -3.0 V, and finishing at 0

V. Glassy carbon electrode S27 working electrode, Ag/AgCl (KCl 3.5 M) reference electrode, Pt wire auxiliary electrode.

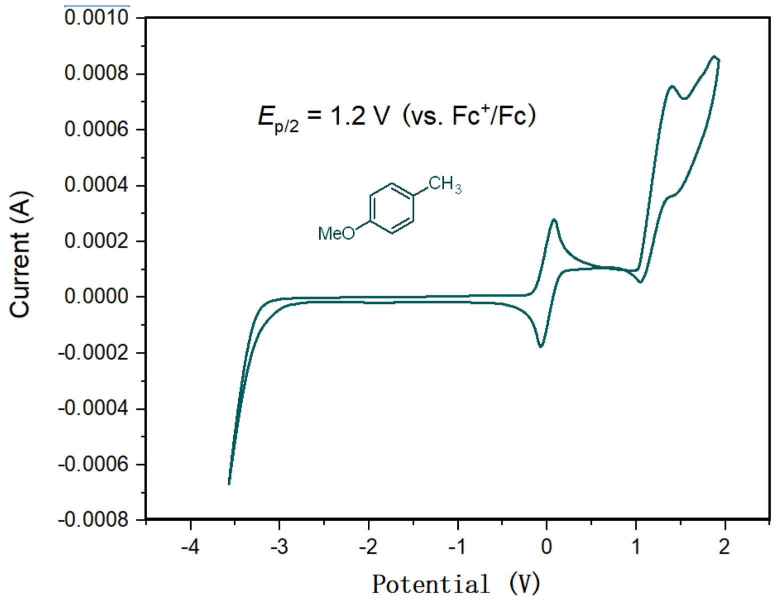

Supplementary Figure 11: Cyclic voltammogram 4. Cyclic voltammogram for 4-methylanisole [0.002 M] and ferrocene [0.002 M] in [0.01 M] $TBAPF_6$ in CH_3CN. Measurement started by reduction from 0 to 2.5 V, followed by oxidation from 2.5 to -3.0 V, and finishing at 0 V. Glassy carbon electrode S27 working electrode, Ag/AgCl (KCl 3.5 M) reference electrode, Pt wire auxiliary electrode.

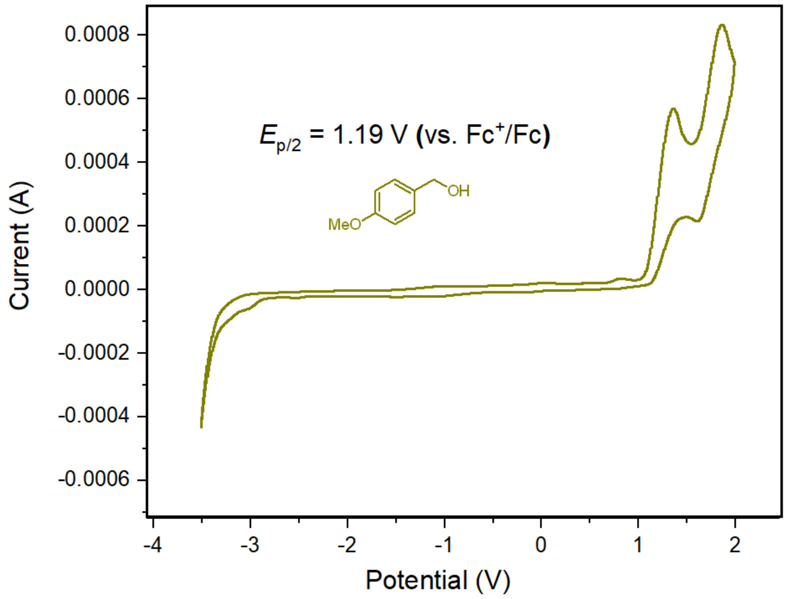

Supplementary Figure 12: Cyclic voltammogram 5. Cyclic voltammogram for 4-methoxybenzyl alcohol [0.002 M] in [0.01 M] $TBAPF_6$ in CH_3CN. Measurement started by reduction from 0 to 2.5 V, followed by oxidation from 2.5 to -3.0 V, and finishing at 0 V. Glassy carbon electrode S27 working electrode, Ag/AgCl (KCl 3.5 M) reference electrode, Pt wire auxiliary electrode.

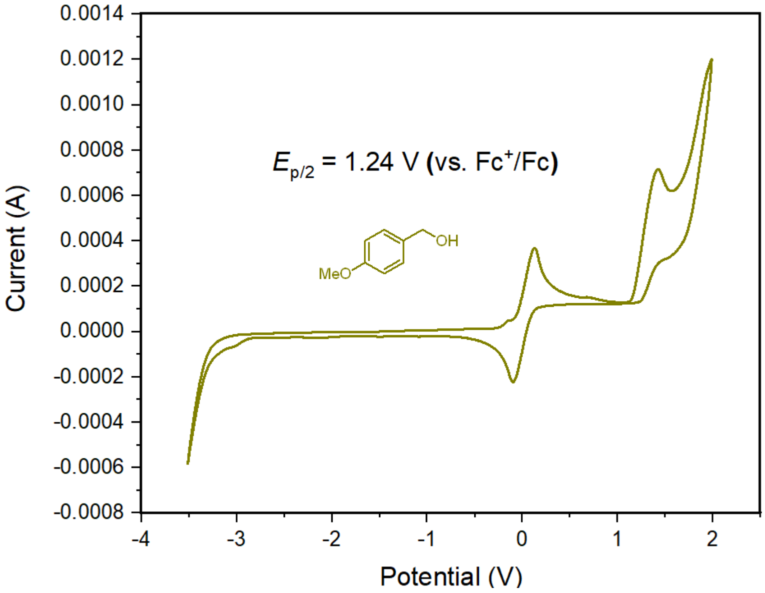

Supplementary Figure 13: Cyclic voltammogram 6. Cyclic voltammogram for 4-methoxybenzyl alcohol [0.002 M] and ferrocene [0.002 M] in [0.01 M] $TBAPF_6$ in CH_3CN. Measurement started by reduction from 0 to 2.5 V, followed by oxidation from 2.5 to -3.0 V, and finishing at 0 V. Glassy carbon electrode S27 working electrode, Ag/AgCl (KCl 3.5 M) reference electrode, Pt wire auxiliary electrode.

6. Determination of quantum yield

(1) Determination of the light intensity at 390 nm:

The photon flux of the spectrophotometer was determined by standard ferrioxalate actinometry (referred to the reported procedure: M. A. Cismesia, et al., *Chem. Sci.* **2015**, *6*, 5426-5434). A 0.006 M solution of ferrioxalate was prepared by dissolving 147.6 mg of potassium ferrioxalate hydrate in 50 mL of 0.05 M H_2SO_4. A buffered solution of phenanthroline was prepared by dissolving 100.0 mg of phenanthroline and 22.1 mg of sodium acetate in 100 mL of 0.5 M H_2SO_4. Both solutions were stored in the dark. To determine the photon flux of the spectrophotometer, 5.0 mL of the ferrioxalate solution was placed in a 10 mL quartz reaction tube (V = 5.0 mL) and irradiated for 20.0 seconds with a commercial 100 W 390 nm laser. After irradiation, the solution was shielded with aluminum foil and 0.88 mL of the phenanthroline solution was added. The solution was then allowed to rest for 1 h to allow the ferrous ions to coordinate to the phenanthroline completely. The absorbance of the solution was measured at 510 nm. A non-irradiated sample was also prepared and the absorbance at 510 nm measured. Conversion was calculated using eq 1.

$$mol\ Fe^{2+} = (V \cdot \Delta A)/(l \cdot \varepsilon) \qquad (1)$$

Where V is the total volume (0.00588 L) of the solution after addition of phenanthroline, ΔA is the difference in absorbance at 510 nm between the irradiated and non-irradiated solutions, l is the path length (1.000 cm), and ε is the molar absorptivity at 510 nm (11,100 L mol^{-1} cm^{-1}). The photon flux can be calculated using eq 2.

$$Photon\ flux = (mol\ Fe^{2+})/(\Phi \cdot t \cdot f) \qquad (2)$$

Where Φ is the quantum yield for the ferrioxalate actinometer (1.19 for a 0.006 M solution at λ = 390 nm), t is the time (20.0 s), and f is the fraction of light absorbed at λ = 390 nm (A = 2.1570, see Supplementary Figure 14). The photon flux was calculated to be 1.85 × 10^{-8} einstein s^{-1}.

$$mol\ Fe^{2+} = (0.00588\ L \cdot 0.8261)/(1.000\ cm \cdot 11100\ L\ mol^{-1} cm^{-1}) = 4.38 \times 10^{-7}\ mol$$

$$Photon\ flux = (4.38 \times 10^{-7}\ mol)/\ (1.19 \cdot 20.0\ s \cdot 0.9930) = 1.85 \times 10^{-8}\ einstein\ s^{-1}$$

(2) Determination of fraction of light absorbed at 390 nm for the ferrioxalate solution:

The absorbance of the above ferrioxalate solution at 390 nm was measured to be 2.1570. The fraction of light absorbed (f) by this solution was calculated using eq 3, where A is the measured absorbance at 390 nm.

$$f = 1 - 10^{-A} \qquad (3)$$

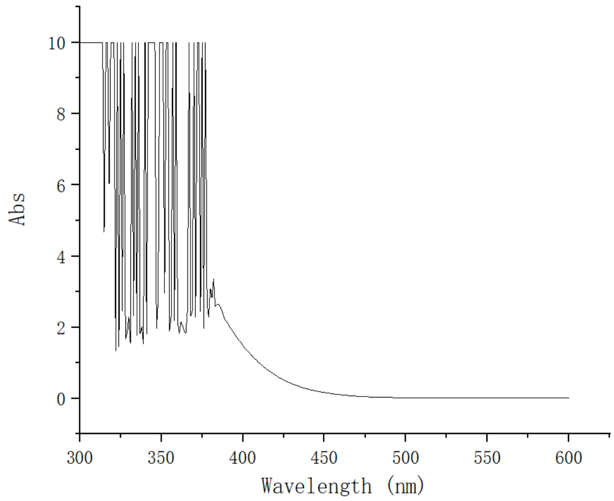

Supplementary Figure 14. Absorbance of the ferrioxalate actinometer solution. Absorbance of the ferrioxalate actinometer solution.

(3) Determination of quantum yield

The reaction sample was prepared following the general procedure, and subsequently the sample was irradiated with a commercial 100 W 390 nm laser. After irradiation, the yield of **3aa** was detected by ^{1}H NMR spectrum of crude product with an internal standard of 1,3,5-trimethoxybenzene. The quantum yield was determined using eq 4.

$$\Phi = mol\ product/(flux \cdot t \cdot f) \qquad (4)$$

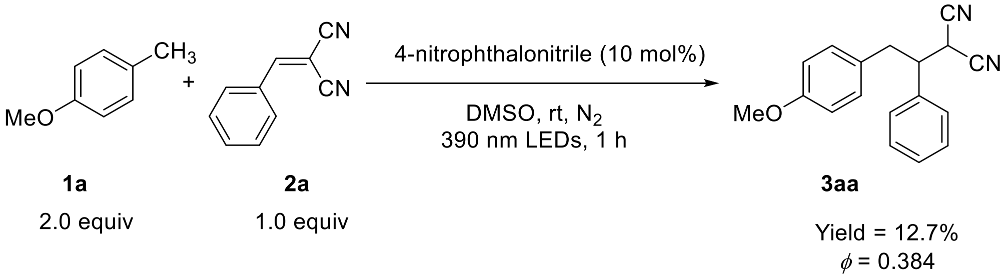

To a 4 mL vial were added **1a** (48.6 mg, 0.4 mmol), **2a** (30.6 mg, 0.2 mmol), and 4-nitrophthalonitrile (3.5 mg, 0.02 mmol) in DMSO (2.0 mL) in an N_2 glovebox. The vial was sealed and transferred out of glovebox. Under irradiation at 390 nm LEDs, the resulting mixture was stirred for 24 hours at rt. Saturated brine was then added and the aqueous layer was extracted with ethyl acetate (10 mL × 3) and evaporation. Yield: 12.7 %, Φ = 0.384.

$$\Phi = (2 \times 10^{-4}\ mol \times 12.7\ \%)/\ (1.85 \times 10^{-8}\ einstein\ s^{-1} \cdot 3600\ s \cdot 0.9930) = 0.384$$

7. UV-Vis absorption spectra

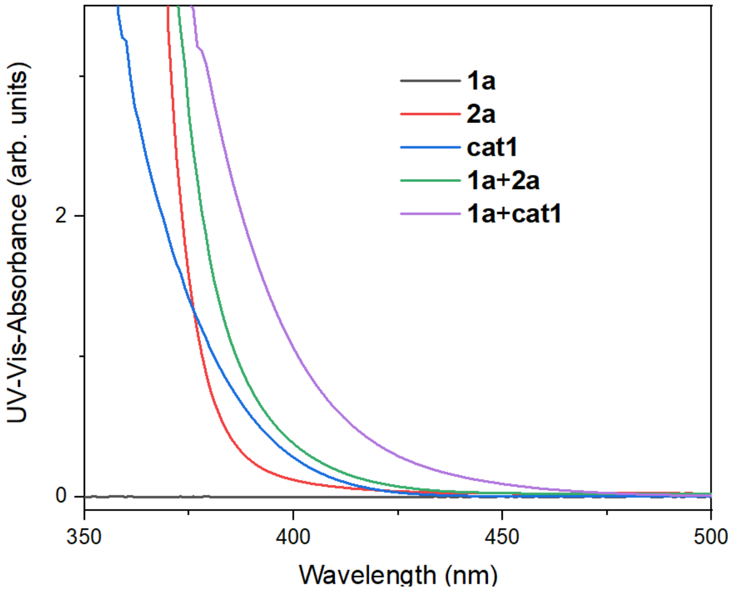

Supplementary Figure 15: **The UV-Vis absorption spectra.** The UV-Vis absorption of the solution of **1a** (0.2 M), **2a** (0.1 M), **cat1** (0.01 M), the mixture of **1a** (0.2 M) and **2a** (0.1 M), and the mixture of **1a** (0.2 M) and **cat1** (0.01 M) in DMSO

The UV-Vis absorption of the mixture of **1a** and **cat1** showcased the increasing absorption at the $\lambda = 350 \sim 500$ nm, indicating the formed EDA complex possessed the new visible-light absorptions to facilitate the photo-excitation. Notably, the mixture of **1a** and **2a** also presented a relative smaller increasing absorption compared with **1a**/**cat1**, which could explain why the reaction in the absence of **cat1** could proceed in lower efficiency.

Oxidation of e-rich aromatics

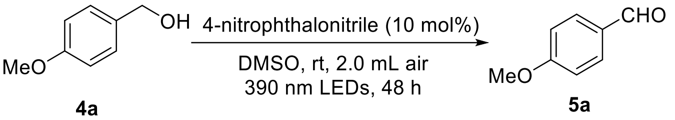

4a **5a** 81%

Typical Procedure 3: To a 4 mL vial were added **4a** (27.8 mg, 0.2 mmol), and 4-nitrophthalonitrile (3.4 mg, 0.02 mmol) in DMSO (2.0 mL) in air. Under irradiation at 390 nm LEDs, the resulting mixture in 2.0 ml air was stirred for 48 hours at rt. Saturated brine was then added and the aqueous layer was extracted with ethyl acetate (10 mL × 3), Evaporation and flash chromatography on silica gel afforded **5a** (22.1 mg, 81%) (eluent: petroleum ether/ethyl acetate = 20/1): colorless oil. ^{1}H NMR (400 MHz, $CDCl_3$) δ 9.89 (s, 1 H, CHO), 7.84 (d, *J* = 8.8 Hz, 2 H, Ar-H), 7.01 (d, *J* = 8.8 Hz, 2 H, Ar-H), 3.89 (s, 3 H, CH_3). ^{13}C NMR (101 MHz, $CDCl_3$) δ 190.8, 164.6, 132.0, 129.9, 114.3, 55.6.

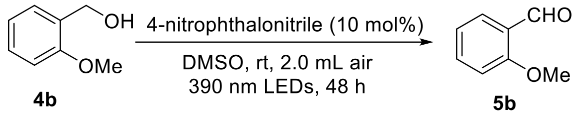

4b **5b** 82%

Following Typical Procedure 3, the reaction of **4b** (27.7 mg, 0.2 mmol), and 4-nitrophthalonitrile (3.3 mg, 0.02 mmol) in DMSO (2.0 mL) afforded **5b** (22.5 mg, 82%) (eluent: petroleum ether/ethyl acetate = 20/1): colorless oil. ^{1}H NMR (400 MHz, $CDCl_3$) 10.47 (s, 1 H, CHO), 7.85-7.79 (m, 1 H, Ar-H), 7.59-7.52 (m, 1 H, Ar-H), 7.06-6.95 (m, 2 H, Ar-H), 3.92 (s, 3 H, CH_3). ^{13}C NMR (101 MHz, $CDCl_3$) δ 189.8, 161.8, 135.9, 128.5, 124.8, 120.6, 111.6, 55.6.

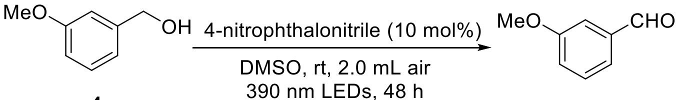

4c **5c** 58%

Following Typical Procedure 3, the reaction of **4c** (27.3 mg, 0.2 mmol), and 4-nitrophthalonitrile (3.8 mg, 0.02 mmol) in DMSO (2.0 mL) afforded **5c** (15.7 mg, 58%) (eluent: petroleum ether/ethyl acetate = 20/1): colorless liquid. ^{1}H NMR (400 MHz, $CDCl_3$) 9.98 (s, 1 H, CHO), 7.49-7.38 (m, 3 H, Ar-H), 7.22-7.14 (m, 1 H, Ar-H), 3.87 (s, 3 H, CH_3). ^{13}C NMR (101 MHz, $CDCl_3$) δ 192.1, 160.1, 137.8, 130.0, 123.6, 121.5, 112.0, 55.5.

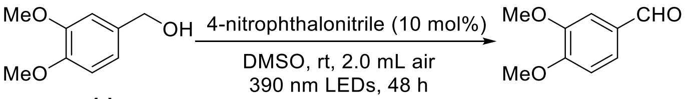

4d **5d** quant.

Following Typical Procedure 3, the reaction of **4d** (33.9 mg, 0.2 mmol), and 4-nitrophthalonitrile (3.6 mg, 0.02 mmol) in DMSO (2.0 mL) afforded **5d** (35.8 mg, quant.) (eluent: petroleum ether/ethyl acetate = 10/1): colorless liquid. ^{1}H NMR (400 MHz, $CDCl_3$) 9.86-9.74 (m, 1 H, CHO), 7.51-7.33 (m, 2 H, Ar-H), 7.01-6.87 (m, 1 H, Ar-H), 4.00-3.83 (m, 6 H, CH_3 × 2). ^{13}C NMR (101 MHz, $CDCl_3$) δ 190.9, 154.49,

149.59, 130.1, 126.8, 110.3, 108.8, 56.2, 56.0.

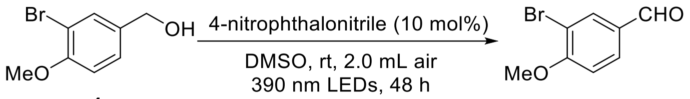

4e **5e** 75%

Following Typical Procedure 3, the reaction of **4e** (43.4 mg, 0.2 mmol), and 4-nitrophthalonitrile (3.6 mg, 0.02 mmol) in DMSO (2.0 mL) afforded **5e** (32.3 mg, 75%) (eluent: petroleum ether/ethyl acetate = 50/1 to 20/1): colorless liquid. ^{1}H NMR (400 MHz, $CDCl_3$) 9.85 (s, 1 H, CHO), 8.09 (s, 1 H, Ar-H), 7.87-7.79 (m, 2 H, Ar-H), 7.01 (d, J = 8.8 Hz, 1 H, Ar-H), 3.99 (s, 3 H, CH_3). ^{13}C NMR (101 MHz, $CDCl_3$) δ 189.6, 160.6, 134.6, 131.2, 130.7, 112.6, 111.5, 56.6.

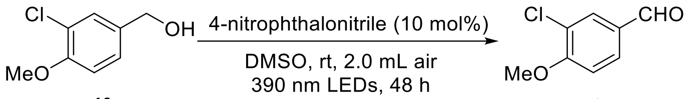

4f **5f** 94%

Following Typical Procedure 3, the reaction of **4f** (34.8 mg, 0.2 mmol), and 4-nitrophthalonitrile (3.6 mg, 0.02 mmol) in DMSO (2.0 mL) afforded **5f** (32.3 mg, 94%) (eluent: petroleum ether/ethyl acetate = 20/1): colorless liquid. ^{1}H NMR (400 MHz, $CDCl_3$) 9.85 (s, 1 H, CHO), 7.90 (d, J = 2.0 Hz, 1 H, Ar-H), 7.81-7.75 (m, 1 H, Ar-H), 7.04 (d, J = 8.8 Hz, 1 H, Ar-H), 3.99 (s, 3 H, CH_3). ^{13}C NMR (101 MHz, $CDCl_3$) δ 189.7, 159.8, 131.2, 130.5, 130.3, 123.7, 111.7, 56.5.

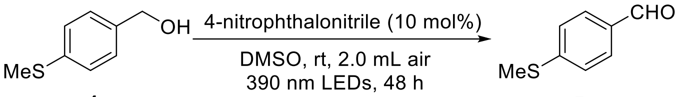

4g **5g** 82%

Following Typical Procedure 3, the reaction of **4g** (31.1 mg, 0.2 mmol), and 4-nitrophthalonitrile (3.6 mg, 0.02 mmol) in DMSO (2.0 mL) afforded **5g** (25.3 mg, 82%) (eluent: petroleum ether/ethyl acetate = 20/1): colorless liquid. ^{1}H NMR (400 MHz, $CDCl_3$) 9.91 (s, 1 H, CHO), 7.76 (d, J = 8.4 Hz, 2 H, Ar-H), 7.31 (d, J = 8.4 Hz, 1 H, Ar-H), 2.53 (s, 3 H, CH_3). ^{13}C NMR (101 MHz, $CDCl_3$) δ 191.2, 147.9, 132.9, 129.9, 125.1, 14.6.

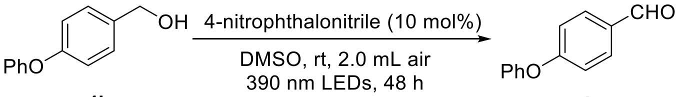

4h **5h** 74%

Following Typical Procedure 3, the reaction of **4h** (40.4 mg, 0.2 mmol), and 4-nitrophthalonitrile (3.6 mg, 0.02 mmol) in DMSO (2.0 mL) afforded **5h** (29.4 mg, 74%) (eluent: petroleum ether/ethyl acetate = 20/1): colorless oil. ^{1}H NMR (400 MHz, $CDCl_3$) 9.92 (s, 1 H, CHO), 7.85 (d, J = 8.8 Hz, 2 H, Ar-H), 7.46-7.37 (m, 2 H, Ar-H), 7.26-7.18 (m, 1 H, Ar-H), 7.13-7.03 (m, 4 H, Ar-H). ^{13}C NMR (101 MHz, $CDCl_3$) δ 190.8, 132.0, 130.1, 125.0, 124.9, 120.4, 117.6.

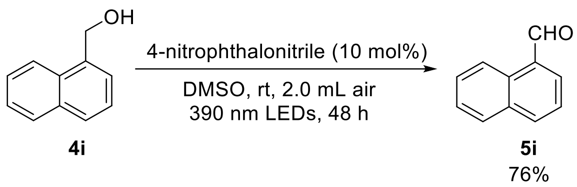

Following Typical Procedure 3, the reaction of **4i** (31.4 mg, 0.2 mmol), and 4-nitrophthalonitrile (3.3 mg, 0.02 mmol) in DMSO (2.0 mL) afforded **5i** (23.7 mg, 76%) (eluent: petroleum ether/ethyl acetate = 20/1): colorless liquid. ^{1}H NMR (400 MHz, $CDCl_3$) 10.40 (s, 1 H, CHO), 9.26 (d, J = 8.8 Hz, 2 H, Ar-H), 8.10 (d, J = 8.4 Hz, 1 H, Ar-H), 8.02-7.97 (m, 1 H, Ar-H), 7.92 (d, J = 8.0 Hz, 1 H, Ar-H), 7.76-7.65 (m, 1 H, Ar-H), 7.65-7.56 (m, 2 H, Ar-H). ^{13}C NMR (101 MHz, $CDCl_3$) δ 193.5, 136.7, 135.3, 133.7, 131.4, 130.5, 129.0, 128.4, 126.9, 124.8.

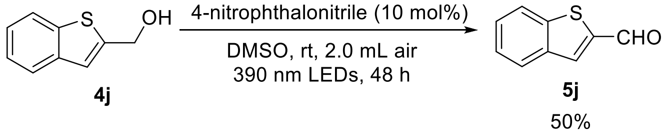

Following Typical Procedure 3, the reaction of **4j** (33.0 mg, 0.2 mmol), and 4-nitrophthalonitrile (3.6 mg, 0.02 mmol) in DMSO (2.0 mL) afforded **5j** (16.3 mg, 50%) (eluent: petroleum ether/ethyl acetate = 20/1): colorless liquid. ^{1}H NMR (400 MHz, $CDCl_3$) 10.11 (s, 1 H, CHO), 8.04 (s, 1 H, Ar-H), 7.98-7.86 (m, 2 H, Ar-H), 7.56-7.41 (m, 2 H, Ar-H). ^{13}C NMR (101 MHz, $CDCl_3$) δ 184.7, 143.3, 142.7, 138.5, 134.5, 128.2, 126.3, 125.2, 123.3.

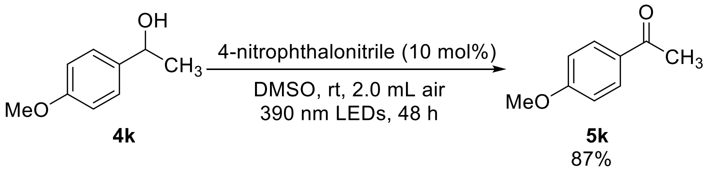

Following Typical Procedure 3, the reaction of **4k** (30.5 mg, 0.2 mmol), and 4-nitrophthalonitrile (3.6 mg, 0.02 mmol) in DMSO (2.0 mL) afforded **5k** (25.9 mg, 87%) (eluent: petroleum ether/ethyl acetate = 10/1): colorless liquid. ^{1}H NMR (400 MHz, $CDCl_3$) 7.93 (d, J = 8.8 Hz, 2 H, Ar-H), 6.92 (d, J = 9.2 Hz, 2 H, Ar-H), 3.86 (s, 3 H, CH_3), 2.55 (s, 3 H, CH_3). ^{13}C NMR (101 MHz, $CDCl_3$) δ 196.8, 163.4, 130.5, 130.3, 113.6, 55.4, 26.3. IR ν (neat, cm^{-1}) 3003, 1676.

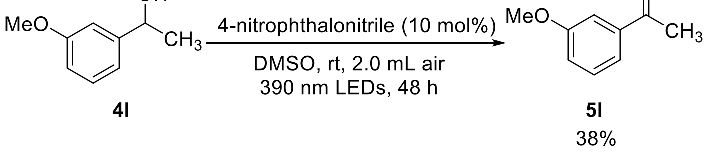

Following Typical Procedure 3, the reaction of **4l** (30.3 mg, 0.2 mmol), and 4-nitrophthalonitrile (3.6 mg, 0.02 mmol) in DMSO (2.0 mL) afforded **5l** (11.4 mg, 38%) (eluent: petroleum ether/ethyl acetate = 20/1): colorless liquid. ^{1}H NMR (400 MHz, $CDCl_3$) 7.52 (d, J = 7.6 Hz, 1 H, Ar-H), 7.48-7.45 (m, 1 H, Ar-H), 7.38-7.32 (m, 1 H, Ar-H), 7.12-7.07 (m, 1 H, Ar-H), 3.84 (s, 3 H, CH_3), 2.58 (s, 3 H, CH_3). ^{13}C NMR (101

MHz, $CDCl_3$) δ 197.9, 159.7, 138.4, 129.5, 121.1, 119.5, 112.2, 55.3, 26.7. IR ν (neat, cm^{-1}) 3002, 1689.

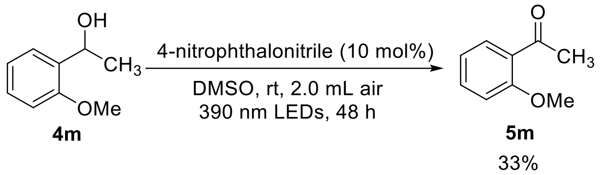

Following Typical Procedure 3, the reaction of **4m** (30.5 mg, 0.2 mmol), and 4-nitrophthalonitrile (3.6 mg, 0.02 mmol) in DMSO (2.0 mL) afforded **5m** (10.0 mg, 33%) (eluent: petroleum ether/ethyl acetate = 20/1): colorless liquid. ^{1}H NMR (400 MHz, $CDCl_3$) 7.72-7.67 (m, 1 H, Ar-H), 7.41-7.35 (m, 1 H, Ar-H), 7.30-7.21 (m, 2 H, Ar-H), 2.59 (s, 3 H, CH_3), 2.53 (s, 3 H, CH_3). ^{13}C NMR (101 MHz, $CDCl_3$) δ 201.7, 138.4, 137.6, 132.0, 131.5, 129.3, 29.6, 21.6. IR ν (neat, cm^{-1}) 1682.

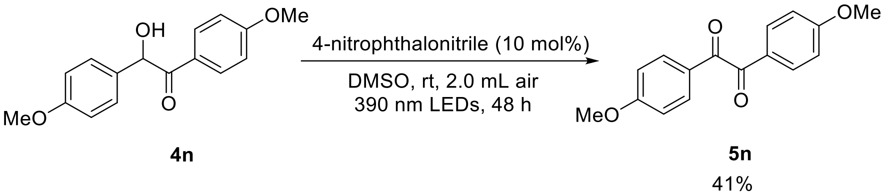

Following Typical Procedure 3, the reaction of **4n** (54.2 mg, 0.2 mmol), and 4-nitrophthalonitrile (3.6 mg, 0.02 mmol) in DMSO (2.0 mL) afforded **5n** (21.9 mg, 41%) (eluent: petroleum ether/ethyl acetate = 50/1): yellow liquid. ^{1}H NMR (400 MHz, $CDCl_3$) 7.94 (d, J = 8.8 Hz, 1 H, Ar-H), 6.97 (d, J = 8.8 Hz, 1 H, Ar-H), 3.88 (s, 3 H, CH_3). ^{13}C NMR (101 MHz, $CDCl_3$) δ 193.5, 164.8, 132.4, 126.3, 114.3, 55.6. IR ν (neat, cm^{-1}) 3026, 1657.

Hydrocarboxylation of alkenes

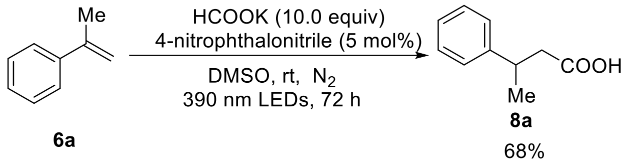

Typical Procedure 4: To a 4 mL vial were added **6a** (23.8 mg, 0.2 mmol), HCOOK (168.2 mg, 2.0 mmol), and 4-nitrophthalonitrile (1.8 mg, 0.01 mmol) in DMSO (2.0 mL) in an N_2 glovebox. The vial was sealed and transferred out of glovebox. Under irradiation at 390 nm LEDs, the resulting mixture was stirred for 72 hours at rt. The mixture was acidified with 2 mL dilute HCl (2 N) and quenched with H_2O and the aqueous layer was extracted with ethyl acetate (10 mL × 3). Evaporation and flash chromatography on silica gel afforded **8a** (22.4 mg, 68%) (eluent: petroleum ether/ethyl acetate = 10/1 to 2/1): oil. ^{1}H NMR (400 MHz, $CDCl_3$) δ 7.34-7.28 (m, 2 H, Ar-H), 7.25-7.18 (m, 3 H, Ar-H), 3.34-3.22 (m, 1 H, CH), 2.73-2.53 (m, 2 H, CH_2), 1.32 (d, J = 6.8 Hz, 3 H, CH_3). ^{13}C NMR (101 MHz, $CDCl_3$) δ 178.3, 145.4, 128.5, 126.7, 126.5, 42.5, 36.1, 21.8. IR ν (neat, cm^{-1}) 3026, 1707. The ^{1}H NMR spectra are matching with the known literature.[18]

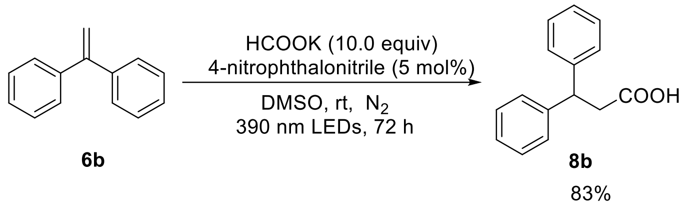

Following Typical Procedure 4, the reaction of **6b** (35.4 mg, 0.2 mmol), HCOOK (168.2 mg, 2.0 mmol), and 4-nitrophthalonitrile (1.7 mg, 0.02 mmol) in DMSO (2.0 mL) afforded **8b** (36.7 mg, 83%) (eluent: petroleum ether/ethyl acetate = 10/1 to 1/1): white solid. ^{1}H NMR (400 MHz, $CDCl_3$) δ 7.35-7.16 (m, 10 H, Ar-H), 4.54 (t, J = 7.8 Hz, 1 H, CH), 3.11 (d, J = 8.0 Hz, 2 H, CH_2). ^{13}C NMR (101 MHz, $CDCl_3$) δ 177.8, 143.2, 128.6, 127.6, 126.6, 46.6, 40.4. IR ν (neat, cm^{-1}) 3025, 1702. The ^{1}H NMR spectra are matching with the known literature.[18]

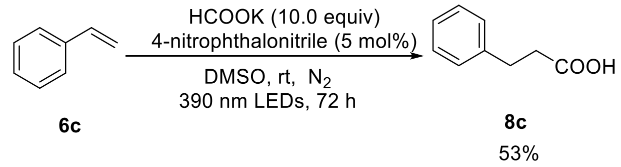

Following Typical Procedure 4, the reaction of **6c** (20.4 mg, 0.2 mmol), HCOOK (168.5 mg, 2.0 mmol), and 4-nitrophthalonitrile (1.8 mg, 0.02 mmol) in DMSO (2.0 mL) afforded **8c** (15.7 mg, 53%) (eluent: petroleum ether/ethyl acetate = 10/1 to 2/1): oil. ^{1}H NMR (400 MHz, $CDCl_3$) δ 7.34-7.28 (m, 2 H, Ar-H), 7.26-7.19 (m, 3 H, Ar-H), 2.97 (t, J = 7.8 Hz, 2 H, CH_2), 2.74-2.65 (m, 2 H, CH_2). ^{13}C NMR (101 MHz, $CDCl_3$) δ 179.2, 140.1, 128.5, 128.2, 126.4, 35.6, 30.5. IR ν (neat, cm^{-1}) 3028, 1708. The ^{1}H NMR spectra are matching with the known literature.[18]

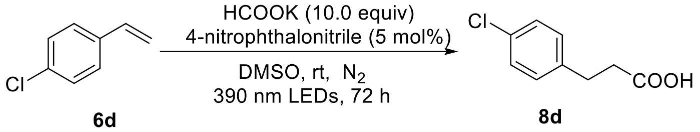

6d **8d** 51%

Following Typical Procedure 4, the reaction of **6d** (27.6 mg, 0.2 mmol), HCOOK (168.0 mg, 2.0 mmol), and 4-nitrophthalonitrile (1.9 mg, 0.02 mmol) in DMSO (2.0 mL) afforded **8d** (18.7 mg, 51%) (eluent: petroleum ether/ethyl acetate = 10/1 to 2/1): white solid. ^{1}H NMR (400 MHz, $CDCl_3$) δ 7.46-7.10 (m, 5 H, Ar-H), 2.93 (t, *J* = 7.6 Hz, 2 H, CH_2), 2.66 (t, *J* = 7.8 Hz, 2 H, CH_2). ^{13}C NMR (101 MHz, $CDCl_3$) δ 177.6, 138.5, 132.2, 129.7, 128.7, 35.2, 29.9. IR ν (neat, cm^{-1}) 3027, 1711. The ^{1}H NMR spectra are matching with the known literature.[18]

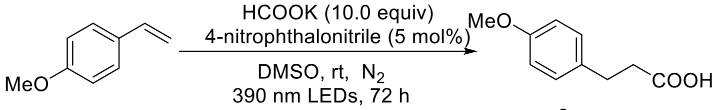

6e **8e** 72%

Following Typical Procedure 4, the reaction of **6e** (26.7 mg, 0.2 mmol), HCOOK (168.2 mg, 2.0 mmol), and 4-nitrophthalonitrile (1.9 mg, 0.02 mmol) in DMSO (2.0 mL) afforded **8e** (25.9 mg, 72%) (eluent: petroleum ether/ethyl acetate = 10/1 to 1/1): oil. ^{1}H NMR (400 MHz, $CDCl_3$) δ 7.13 (d, *J* = 8.4 Hz, 2 H, Ar-H), 6.84 (d, *J* = 8.4 Hz, 2 H, Ar-H), 3.79 (s, 3 H, OCH_3), 2.91 (t, *J* = 7.8 Hz, 2 H, CH_2), 2.65 (t, *J* = 7.8 Hz, 2 H, CH_2). ^{13}C NMR (101 MHz, $CDCl_3$) 178.8, 158.1, 132.2, 129.2, 113.9, 55.2, 35.9, 29.7. IR ν (neat, cm^{-1}) 2957, 1702. The ^{1}H NMR spectra are matching with the known literature.[18]

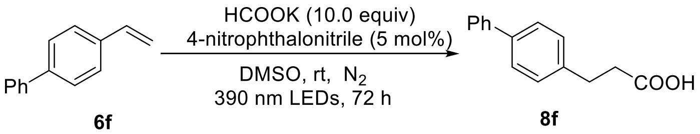

6f **8f** 62%

Following Typical Procedure 4, the reaction of **6f** (36.2 mg, 0.2 mmol), HCOOK (168.5 mg, 2.0 mmol), and 4-nitrophthalonitrile (1.6 mg, 0.02 mmol) in DMSO (2.0 mL) afforded **8f** (28.2 mg, 62%) (eluent: petroleum ether/ethyl acetate = 10/1 to 1/1): white solid. ^{1}H NMR (400 MHz, $CDCl_3$) δ 7.65-7.52 (m, 4 H, Ar-H), 7.50-7.41 (m, 2 H, Ar-H), 7.39-7.28 (m, 3 H, Ar-H), 3.03 (t, *J* = 7.8 Hz, 2 H, CH_2), 2.75 (t, *J* = 7.6 Hz, 2 H, CH_2). ^{13}C NMR (101 MHz, $CDCl_3$) 179.0, 140.8, 139.3, 139.2, 128.72, 128.67, 127.3, 127.1, 127.0, 35.5, 30.2. IR ν (neat, cm^{-1}) 3027, 1694. The ^{1}H NMR spectra are matching with the known literature.[18]

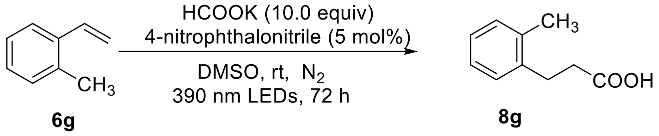

6g **8g** 70%

Following Typical Procedure 4, the reaction of **6g** (23.4 mg, 0.2 mmol), HCOOK (168.2 mg, 2.0 mmol), and 4-nitrophthalonitrile (1.9 mg, 0.02 mmol) in DMSO (2.0 mL) afforded **8g** (22.8 mg, 70%) (eluent: petroleum ether/ethyl acetate = 10/1 to 1/1): white solid. ^{1}H NMR (400 MHz, $CDCl_3$) δ 7.19-7.10 (m, 4 H, Ar-H), 2.96 (t, *J* = 8.0 Hz, 2 H, CH_2), 2.65 (t, *J* = 8.0 Hz, 2 H, CH_2), 2.33 (s, 3 H, CH_3). ^{13}C NMR (101 MHz, $CDCl_3$) 178.8, 138.2, 136.0, 130.3, 128.4, 126.5, 126.2, 34.3, 28.0, 19.2. IR ν (neat, cm^{-1}) 3020, 1708. The ^{1}H NMR spectra are matching with the known literature.[18]

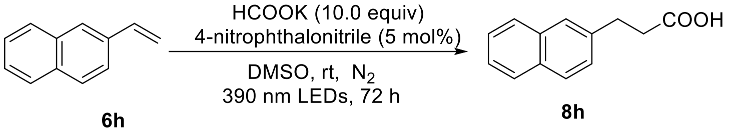

6h **8h** 66%

Following Typical Procedure 4, the reaction of **6h** (30.6 mg, 0.2 mmol), HCOOK (168.2 mg, 2.0 mmol), and 4-nitrophthalonitrile (1.9 mg, 0.02 mmol) in DMSO (2.0 mL) afforded **8h** (26.2 mg, 66%) (eluent: petroleum ether/ethyl acetate = 10/1 to 1/1): white solid. ^{1}H NMR (400 MHz, $CDCl_3$) δ 7.90-7.74 (m, 3 H, Ar-H), 7.70-7.61 (m, 1 H, Ar-H), 7.56-7.41 (m, 2 H, Ar-H), 7.40-7.32 (m, 1 H, Ar-H), 3.13 (t, *J* = 7.8 Hz, 2 H, CH_2), 2.79 (t, *J* = 7.6 Hz, 2 H, CH_2). ^{13}C NMR (101 MHz, $CDCl_3$) 178.5, 137.6, 133.5, 132.2, 128.2, 127.6, 127.5, 126.9, 126.4, 126.1, 125.4, 35.4, 30.7. IR ν (neat, cm^{-1}) 2928, 1711. The ^{1}H NMR spectra are matching with the known literature.[19]

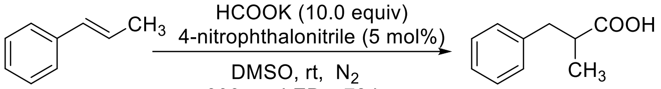

6i **8i** 62%

Following Typical Procedure 4, the reaction of **6i** (23.6 mg, 0.2 mmol), HCOOK (168.2 mg, 2.0 mmol), and 4-nitrophthalonitrile (1.9 mg, 0.02 mmol) in DMSO (2.0 mL) afforded **8i** (20.3 mg, 62%) (eluent: petroleum ether/ethyl acetate = 10/1 to 1/1): oil. ^{1}H NMR (400 MHz, $CDCl_3$) δ 7.35-7.27 (m, 2 H, Ar-H), 7.25-7.17 (m, 3 H, Ar-H), 3.12-3.04 (m, 1 H, CH), 2.84-2.64 (m, 2 H, CH_2), 1.18 (d, *J* = 7.2 Hz, 3 H, CH_3). ^{13}C NMR (101 MHz, $CDCl_3$) 182.3, 139.0, 129.0, 128.4, 126.4, 41.2, 39.3, 16.5. IR ν (neat, cm^{-1}) 2928, 1708. The ^{1}H NMR spectra are matching with the known literature.[18]

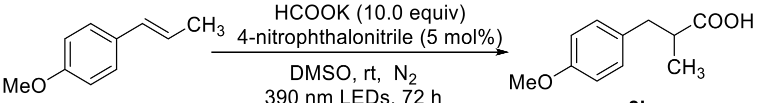

6j **8j** 68%

Following Typical Procedure 4, the reaction of **6j** (29.5 mg, 0.2 mmol), HCOOK (168.5 mg, 2.0 mmol), and 4-nitrophthalonitrile (1.6 mg, 0.02 mmol) in DMSO (2.0 mL) afforded **8j** (26.3 mg, 68%) (eluent: petroleum ether/ethyl acetate = 10/1 to 1/1): oil. ^{1}H NMR (400 MHz, $CDCl_3$) δ 7.10 (d, *J* = 8.4 Hz, 2 H, Ar-H), 6.83 (d, *J* = 8.8 Hz, 2 H, Ar-H), 3.79 (s, 3 H, CH_3), 3.06-2.96 (m, 1 H, CH), 2.78-2.57 (m, 2 H, CH_2), 1.17 (d, *J* = 6.8 Hz, 3 H, CH_2). ^{13}C NMR (101 MHz, $CDCl_3$) 182.3, 139.0, 129.0, 128.4, 126.4, 41.2, 39.3, 16.5. IR ν (neat, cm^{-1}) 2936, 1707. The ^{1}H NMR spectra are matching with the known literature.[20]

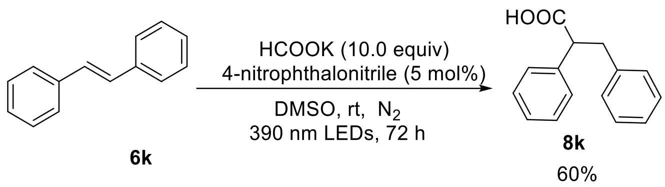

Following Typical Procedure 4, the reaction of **6k** (36.1 mg, 0.2 mmol), HCOOK (167.8 mg, 2.0 mmol), and 4-nitrophthalonitrile (1.7 mg, 0.02 mmol) in DMSO (2.0 mL) afforded **8k** (27.3 mg, 60%) (eluent: petroleum ether/ethyl acetate = 10/1 to 1/1): white solid. ^{1}H NMR (400 MHz, $CDCl_3$) δ 7.35-7.27 (m, 5 H, Ar-H), 7.25-7.09 (m, 5 H, Ar-H), 3.86 (t, J = 7.6 Hz, 1 H, CH), 3.48-3.35 (m, 1 H, CH), 3.09-2.96 (m, 1 H, CH). ^{13}C NMR (101 MHz, $CDCl_3$) 179.6, 138.6, 137.9, 128.9, 128.7, 128.3, 128.1, 127.6, 126.4, 53.4, 39.2. IR ν (neat, cm^{-1}) 2921, 1708. The ^{1}H NMR spectra are matching with the known literature.[20]

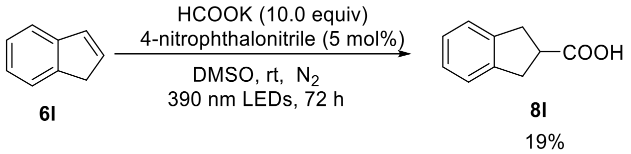

Following Typical Procedure 4, the reaction of **6l** (23.0 mg, 0.2 mmol), HCOOK (168.2 mg, 2.0 mmol), and 4-nitrophthalonitrile (1.9 mg, 0.02 mmol) in DMSO (2.0 mL) afforded **8l** (6.2 mg, 19%) (eluent: petroleum ether/ethyl acetate = 10/1 to 1/1): white solid. ^{1}H NMR (400 MHz, $CDCl_3$) δ 7.25-7.13 (m, 4 H, Ar-H), 3.45-3.19 (m, 5 H, CH_2 × 2 and CH). ^{13}C NMR (101 MHz, $CDCl_3$) 180.8, 141.3, 126.7, 124.3, 43.2, 36.0. IR ν (neat, cm^{-1}) 2924, 1707. The ^{1}H NMR spectra are matching with the known literature.[21]

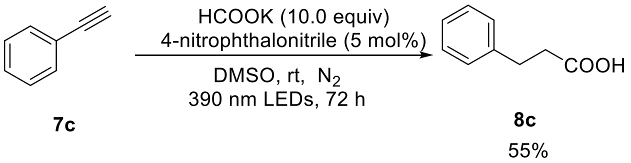

Following Typical Procedure 4, the reaction of **7c** (20.8 mg, 0.2 mmol), HCOOK (168.3 mg, 2.0 mmol), and 4-nitrophthalonitrile (1.8 mg, 0.02 mmol) in DMSO (2.0 mL) afforded **8c** (16.9 mg, 55%) (eluent: petroleum ether/ethyl acetate = 10/1 to 1/1): oil. ^{1}H NMR (400 MHz, $CDCl_3$) δ 7.35-7.29 (m, 2 H, Ar-H), 7.27-7.20 (m, 2 H, Ar-H), 2.98 (t, J = 7.8 Hz, 2 H, CH_2), 2.77-2.64 (m, 2 H, CH_2). 179.4, 140.1, 128.5, 128.2, 126.4, 35.6, 30.5. IR ν (neat, cm^{-1}) 3026, 1707. The ^{1}H NMR spectra are matching with the known literature.[18]

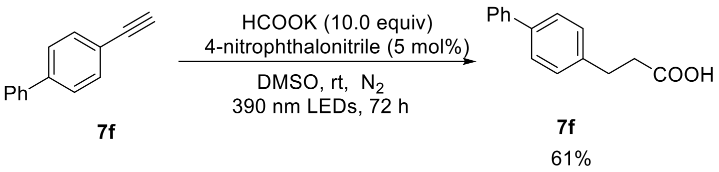

Following Typical Procedure 4, the reaction of **7f** (35.5 mg, 0.2 mmol), HCOOK (168.2 mg, 2.0 mmol), and 4-nitrophthalonitrile (1.8 mg, 0.02 mmol) in DMSO (2.0 mL) afforded **8f** (27.7 mg, 61%) (eluent: petroleum ether/ethyl acetate = 10/1 to 1/1): white

solid. ^{1}H NMR (400 MHz, $CDCl_3$) δ 7.62-7.50 (m, 4 H, Ar-H), 7.48-7.40 (m, 2 H, Ar-H), 7.36-7.28 (m, 3 H, Ar-H), 3.01 (t, J = 7.8 Hz, 2 H, CH_2), 2.73 (t, J = 7.8 Hz, 2 H, CH_2). ^{13}C NMR (101 MHz, $CDCl_3$) 178.6, 140.8, 139.3, 139.2, 128.7, 128.7, 127.3, 127.1, 127.0, 35.4, 30.1. IR ν (neat, cm^{-1}) 3028, 1701. The ^{1}H NMR spectra are matching with the known literature.[18]

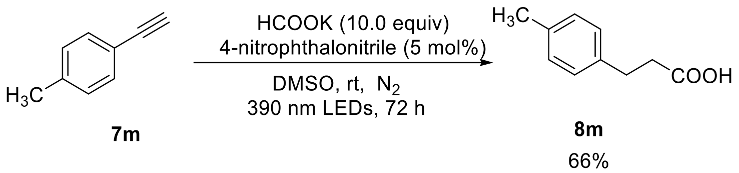

Following Typical Procedure 4, the reaction of **7m** (23.0 mg, 0.2 mmol), HCOOK (168.2 mg, 2.0 mmol), and 4-nitrophthalonitrile (1.8 mg, 0.02 mmol) in DMSO (2.0 mL) afforded **8m** (21.3 mg, 66%) (eluent: petroleum ether/ethyl acetate = 10/1 to 1/1): white solid. ^{1}H NMR (400 MHz, $CDCl_3$) δ 7.18-7.05 (m, 4 H, Ar-H), 2.93 (t, J = 7.8 Hz, 2 H, CH_2), 2.67 (t, J = 7.8 Hz, 2 H, CH_2). ^{13}C NMR (101 MHz, $CDCl_3$) 179.2, 137.0, 135.8, 129.2, 128.1, 35.7, 30.1, 21.0. IR ν (neat, cm^{-1}) 2929, 1717. The ^{1}H NMR spectra are matching with the known literature.[18]

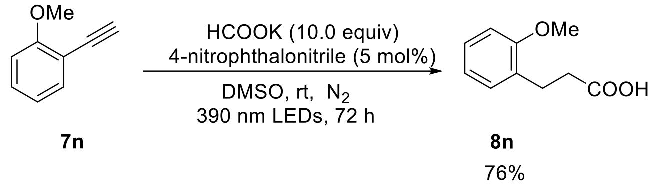

Following Typical Procedure 4, the reaction of **7n** (26.3 mg, 0.2 mmol), HCOOK (167.8 mg, 2.0 mmol), and 4-nitrophthalonitrile (1.8 mg, 0.02 mmol) in DMSO (2.0 mL) afforded **8n** (27.3 mg, 66%) (eluent: petroleum ether/ethyl acetate = 10/1 to 1/1): oil. ^{1}H NMR (400 MHz, $CDCl_3$) δ 7.24-7.14 (m, 2 H, Ar-H), 6.91-6.83 (m, 2 H, Ar-H), 3.82 (s, 3 H, CH_3), 2.95 (t, J = 7.8 Hz, 2 H, CH_2), 2.66 (t, J = 7.8 Hz, 2 H, CH_2). ^{13}C NMR (101 MHz, $CDCl_3$) 179.3, 157.4, 129.9, 128.4, 127.7, 120.4, 110.2, 55.1, 33.9, 25.9. IR ν (neat, cm^{-1}) 2934, 1717. The ^{1}H NMR spectra are matching with the known literature.[22]

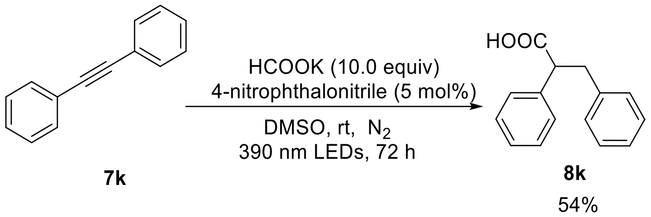

Following Typical Procedure 4, the reaction of **7k** (35.9 mg, 0.2 mmol), HCOOK (168.2 mg, 2.0 mmol), and 4-nitrophthalonitrile (1.6 mg, 0.02 mmol) in DMSO (2.0 mL) afforded **8k** (24.5 mg, 66%) (eluent: petroleum ether/ethyl acetate = 10/1 to 1/1): white solid. mp. 120.1-120.8 ℃. ^{1}H NMR (400 MHz, $CDCl_3$) δ 7.36-7.10 (m, 10 H, Ar-H), 3.94-3.82 (m, 1 H, CH), 3.49-3.37 (m, 1 H, CH), 3.13-3.02 (m, 1 H, CH). ^{13}C NMR (101 MHz, $CDCl_3$) 179.3, 138.6, 137.9, 128.9, 128.7, 128.3, 128.1, 127.6, 126.4,

53.4, 39.2. IR ν (neat, cm^{-1}) 3028, 1704. The ^1H NMR spectra are matching with the known literature.[20]

Mechanistic studies for the formate activation

1. The radical-captured experiment

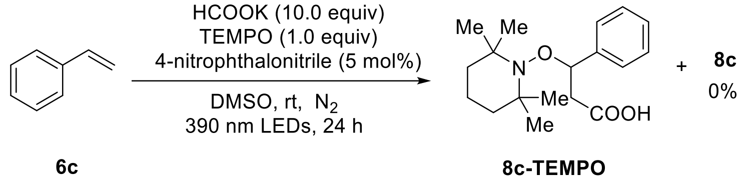

The reaction of **6c**, HCOOK with TEMPO in DMSO under N_2 would produce the radical captured product **8c-TEMPO**, which could be detected by high resolution mass spectroscopy. HRMS (ESI) m/z: $[M]^+$ Calcd for $C_{18}H_{17}NO_3$ 305.1986; found 305.1991.

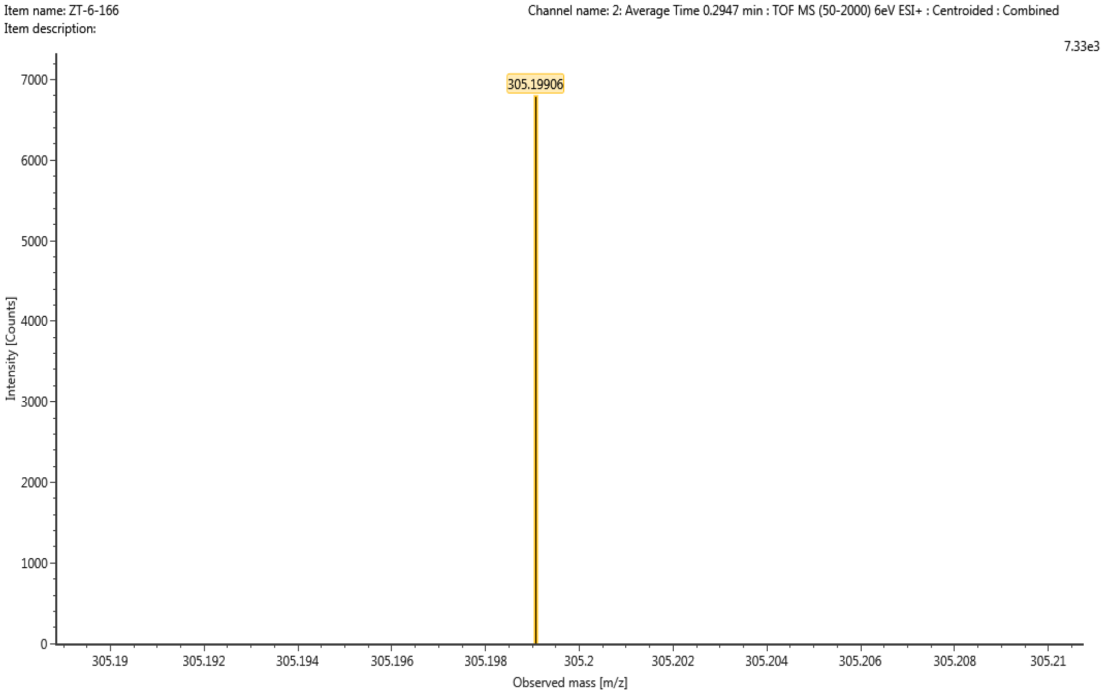

Supplementary Figure 16: **The HRMS spectrum of 8c-TEMPO.** HRMS (ESI) m/z: $[M]^+$ Calcd for $C_{18}H_{17}NO_3$ 305.1986; found 305.1991.

2. The optical absorption spectra of mixture of HCOOK and 4-nitrophthalonitrile.

The UV-Vis absorption of HCOOK and 4-nitrophthalonitrile was showed below. The sum of the concentrations of A and B was 0.01 M in DMSO.

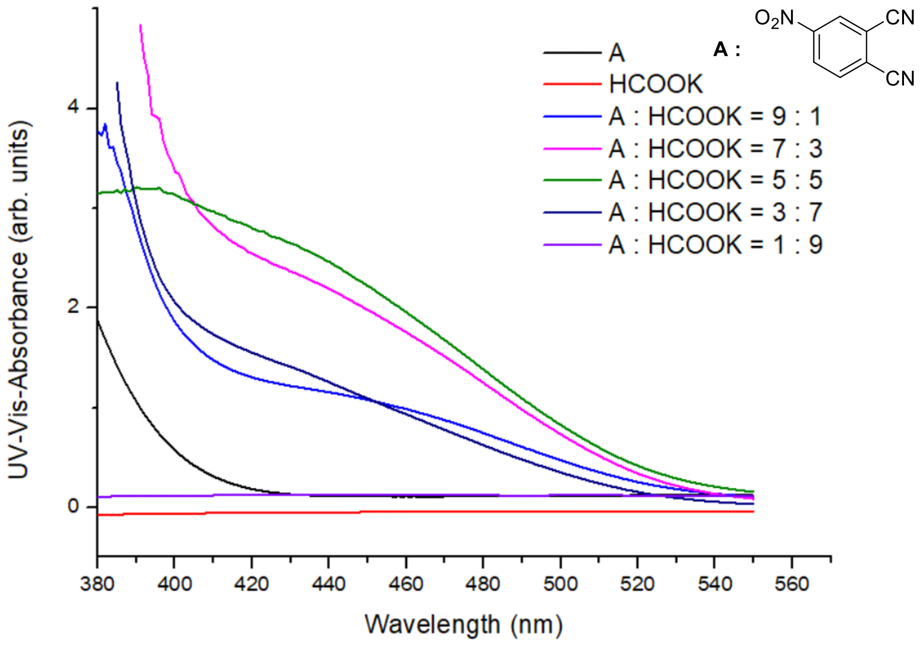

Supplementary Figure 17: The UV-Vis absorption spectra. The UV-Vis absorption of mixture of HCOOK and 4-nitrophthalonitrile in DMSO**.**

Conclusion: UV-Vis spectrum of the mixture of HCOOK and 4-nitrophthalonitrile with a ratio of 1:1 shows a strongest bathochromic shift, which support the formation of ion pair of HCOOK and 4-nitrophthalonitrile in the reaction mixture.

3. ^{1}H NMR shift of 4-nitrophthalonitrile with the addition of HCOOK in DMSO.

The ^{1}H NMR of the mixture HCOOK (0.05 M, 0.1 M and 0. 2 M) and 4-nitrophthalonitrile (0.1 M) in DMSO was showed below.

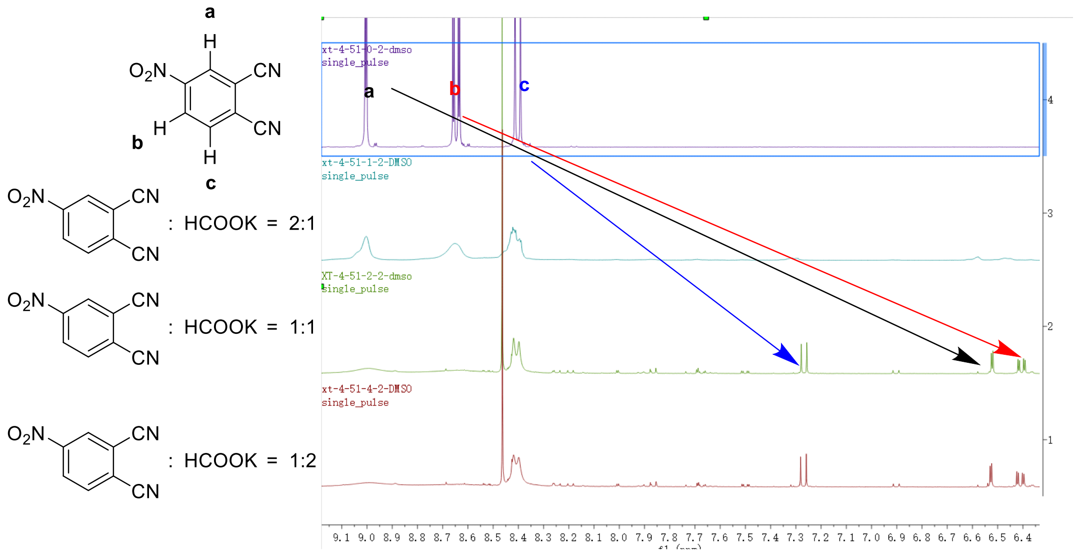

Supplementary Figure 18: Proton NMRs. ^{1}H NMR shift of EDA complex.

Conclusion: A clear upfiled-shift of the ^{1}H NMR signal was observed with the addition

of HCOOK into 4-nitrophthalonitrile. which indicated the interaction of HCOOK and 4-nitrophthalonitrile.

Mechanism of reductive hydrocarboxylation of alkynes

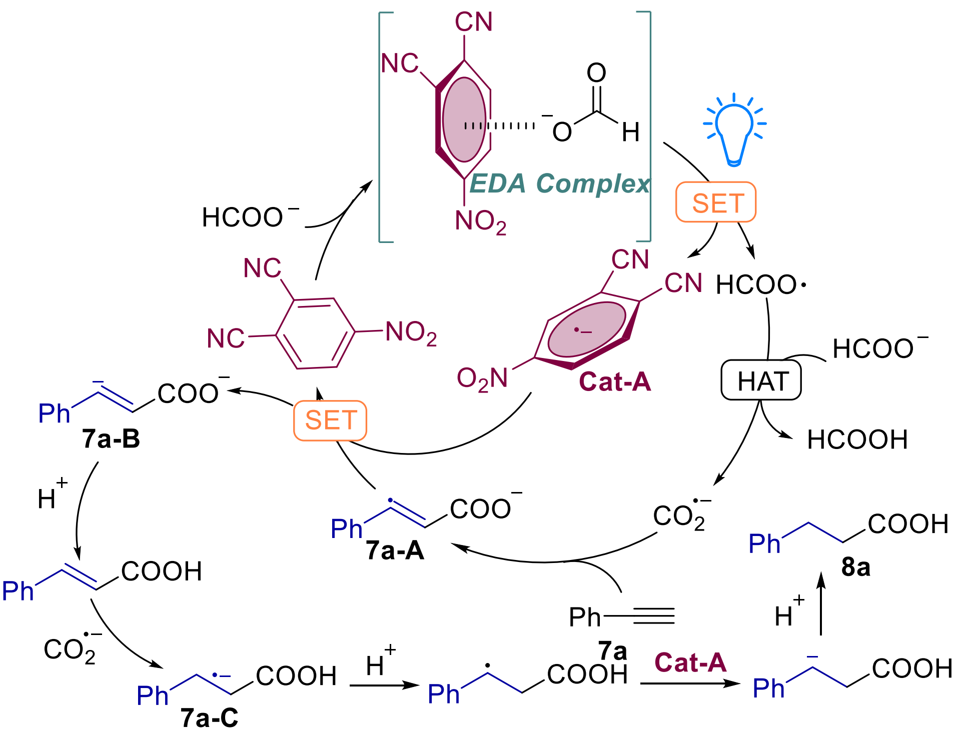

Supplementary Figure 19: Mechanism of reductive hydrocarboxylation of alkynes. The catalytic cycle includes EDA complex formation, photoinduced single electron transfer (SET), deprotonation, radical addition, and protonation.

Synthetic Potentials

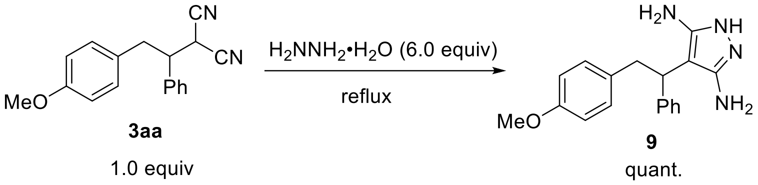

To a 10 mL vial were added **3aa** (110.8 mg, 0.4 mmol), and hydrazine hydrate (120.6 mg, 2.4 mmol) in air. The resulting mixture was heated under reflux for 1.5 hours and cooled to room temperature, then water was added. The insoluble compound was dissolved in ethyl acetate and dried over anhydrous Na_2SO_4. Evaporation afforded compound **9** (122.3 mg, quant.): brown oil. ^{1}H NMR (400 MHz, $CDCl_3$) δ 7.45-7.04 (m, 5 H, Ar-H), 7.00-6.85 (m, 2 H, Ar-H), 6.81-6.66 (m, 2 H, Ar-H), 3.94-3.83 (m, 1 H, CH), 3.77-3.62 (m, 3 H, CH_3), 3.35-3.23 (m, 1 H, CH_2), 3.11-2.91 (m, 1 H, CH_2). ^{13}C NMR (101 MHz, $CDCl_3$) δ 157.9, 148.9, 143.2, 132.7, 130.1, 128.5, 127.6, 126.3, 113.7, 92.8, 55.1, 41.7, 38.3. IR ν (neat, cm^{-1}) 3331. HRMS (ESI) m/z: $[M + Na]^+$ Calcd for $C_{18}H_{20}N_4ONa$ 331.1529; found 331.1518.

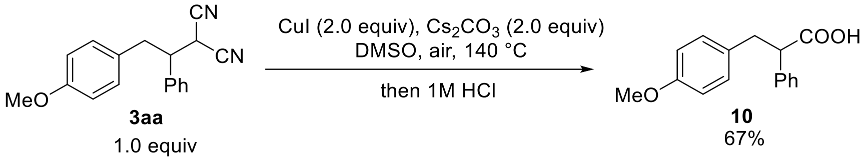

To a 10 mL vial were added **3aa** (110.6 mg, 0.4 mmol), CuI (15.0 mg, 0.08 mmol), and Cs_2CO_3 (260.9 mg, 0.8 mmol) in DMSO (2.0 mL) in air. The resulting mixture was stirred at 140 ℃ in a shot bath (metal pellets) for 24 hours. After cooling to room temperature, HCl (1M) was added until pH reached 2~3, then the solution was extracted with ethyl acetate (10 mL × 3), the combined organic layer was evaporation and flash chromatography on silica gel afforded **10** (68.4 mg, 67%) (eluent: petroleum ether /ethyl acetate = 20/1 to 10/1): colorless oil. ^{1}H NMR (400 MHz, $CDCl_3$) δ 7.35-7.26 (m, 5 H, Ar-H), 7.02 (d, *J* = 8.4 Hz, 2 H, Ar-H), 6.76 (d, *J* = 8.4 Hz, 2 H, Ar-H), 3.89-3.74 (m, 4 H, CH_3 and CH), 3.40-3.29 (m, 1 H, CH_2), 3.02-2.93 (m, 1 H, CH_2). ^{13}C NMR (151 MHz, $CDCl_3$) δ ^{13}C NMR (101 MHz, CHLOROFORM-*D*) δ 158.1, 138.0, 130.7, 129.9, 128.7, 128.1, 127.7, 113.7, 55.2, 53.5, 38.5. IR ν (neat, cm^{-1}) 1697. The ^{1}H NMR spectra are matching with the known literature.[23]

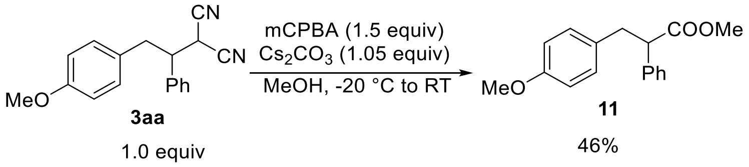

To an oven-dried 10 mL Schleck tube were added **3aa** (110.8 mg, 0.4 mmol), *m*CPBA (103.6 mg, 0.6 mmol), and Cs_2CO_3 (136.6 mg, 0.42 mmol) in MeOH (2.0 mL) in nitrogen. The resulting mixture was stirred at -20 ℃ for 5 hours, the reaction was warmed to room temperature and stirred for another 19 hours. The mixture was filtered through a short column of SiO_2, evaporation and flash chromatography on silica gel afforded **11** (50.1 mg, 46%) (eluent: petroleum ether/ethyl acetate = 50/1): colorless oil.

^{1}H NMR (400 MHz, $CDCl_3$) δ 7.40-7.22 (m, 5 H, Ar-H), 7.06 (d, *J* = 8.4 Hz, 2 H, Ar-H), 6.80 (d, *J* = 8.4 Hz, 2 H, Ar-H), 3.88-3.81 (m, 1 H, CH), 3.78 (s, 3 H, CH_3), 3.62 (s, 3 H, CH_3), 3.47-3.32 (m, 1 H, CH_2), 3.04-2.95 (m, 1 H, CH_2). ^{13}C NMR (101 MHz, $CDCl_3$) δ 173.8, 158.0, 138.6, 131.0, 129.8, 128.6, 127.9, 127.3, 113.6, 55.1, 53.8, 51.9, 38.9. IR ν (neat, cm^{-1}) 3029, 1731. The ^{1}H NMR spectra are matching with the known literature.[24]

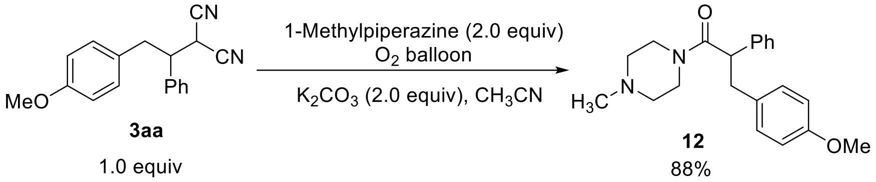

3aa

1.0 equiv

12

88%

To a 10 mL vial were added **3aa** (110.2 mg, 0.4 mmol), K_2CO_3 (110.5 mg, 0.8 mmol), and 1-methylpiperazine (89μL, *d* = 0.903 g/ml, 80.4 mg, 0.8 mmol) in oxygen. The resulting mixture was stirred under oxygen atmosphere for 24 hours, the solution was filtered, the filtrate evaporation and flash chromatography on silica gel afforded **12** (118.8 mg, 88%) (eluent: petroleum ether/ethyl acetate = 1/1 to petroleum ether/ethyl acetate/MeOH = 1/1/1): colorless oil. ^{1}H NMR (400 MHz, $CDCl_3$) δ 7.33-7.11 (m, 5 H, Ar-H), 6.97 (d, *J* = 8.8 Hz, 2 H, Ar-H), 6.73 (d, *J* = 8.4 Hz, 2 H, Ar-H), 3.90 (t, *J* = 7.2 Hz, 1 H, CH), 3.77-3.62 (m, 4 H, CH_2 and CH_3), 3.53-3.44 (m, 1 H, CH_2), 3.44-3.35 (m, 1 H, CH_2), 3.35-3.26 (m, 2 H, CH_2), 2.29-2.20 (m, 1 H, CH_2), 2.19-2.07 (m, 4 H, CH_2 and CH_3), 2.03-1.91 (m, 1 H, CH_2), 1.81-1.68 (m, 1 H, CH_2). ^{13}C NMR (101 MHz, $CDCl_3$) δ 170.8, 157.7, 139.5, 131.7, 129.9, 128.4, 127.7, 126.7, 113.3, 54.9, 54.3, 54.3, 50.8, 45.6, 45.2, 41.7, 39.9. IR ν (neat, cm^{-1}) 3026, 1651. HRMS (ESI) m/z: $[M + H]^+$ Calcd for $C_{21}H_{27}N_2O_2$ 339.2067; found 339.2078.

Spectra

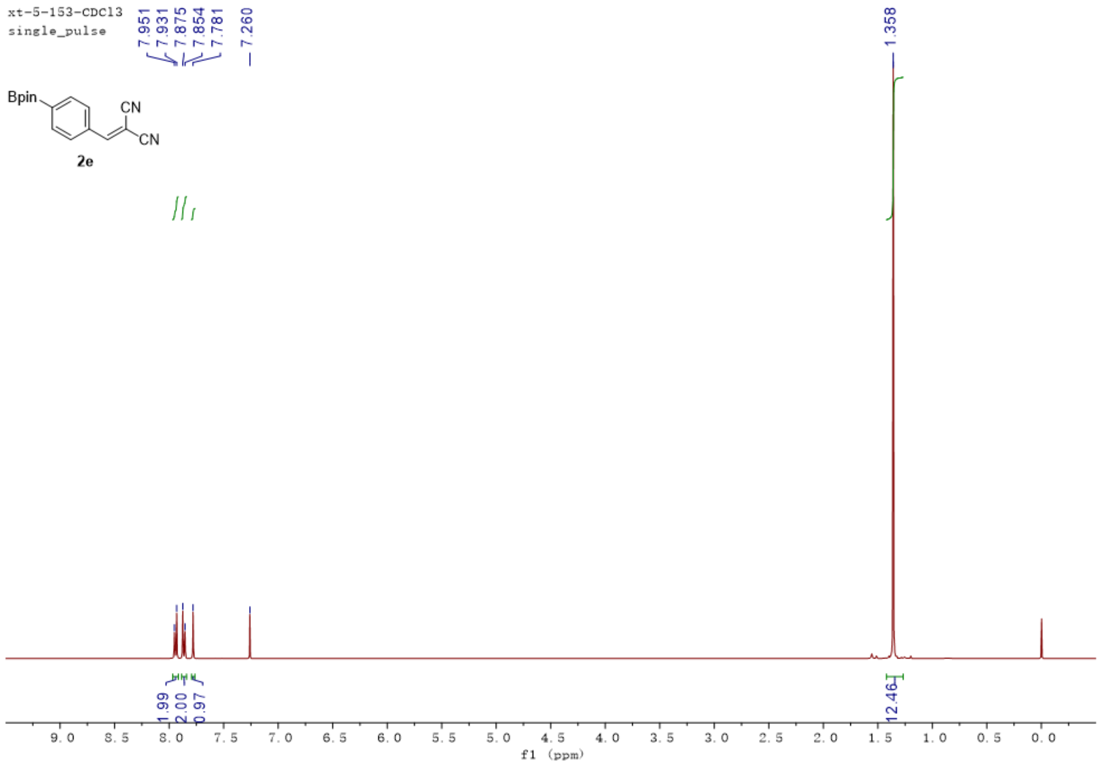

^{1}H NMR (400 MHz, $CDCl_3$) of **2e**

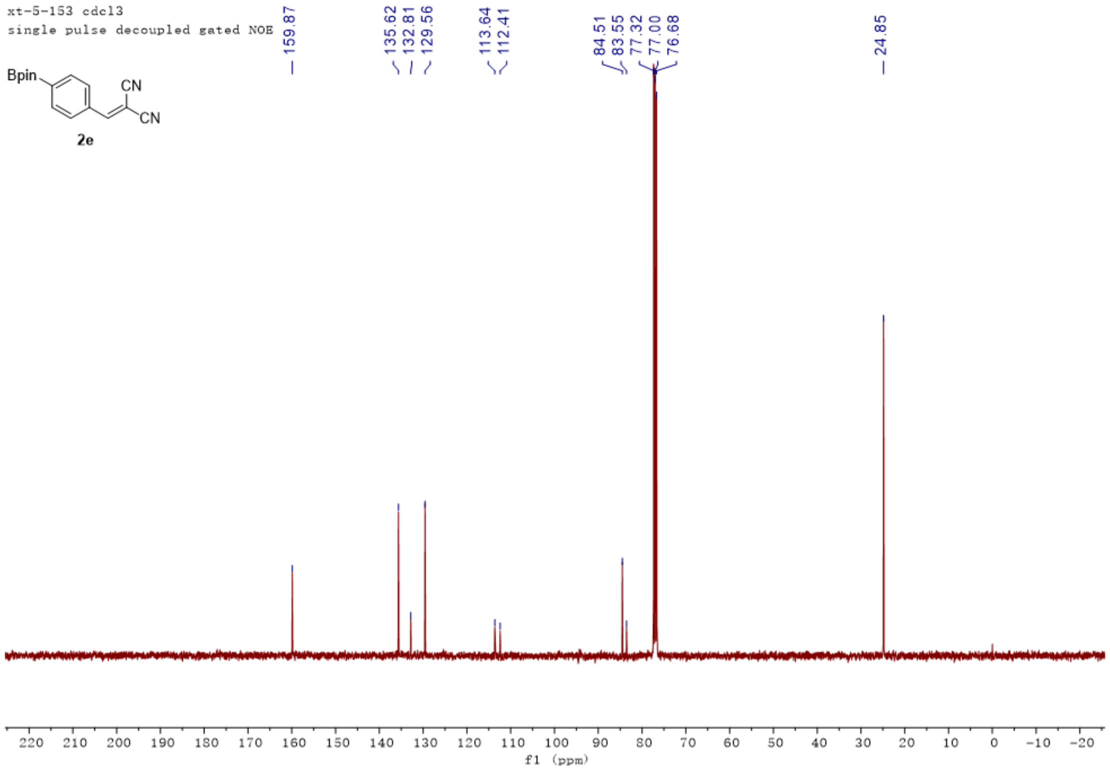

^{13}C NMR (101 MHz, $CDCl_3$) of **2e**

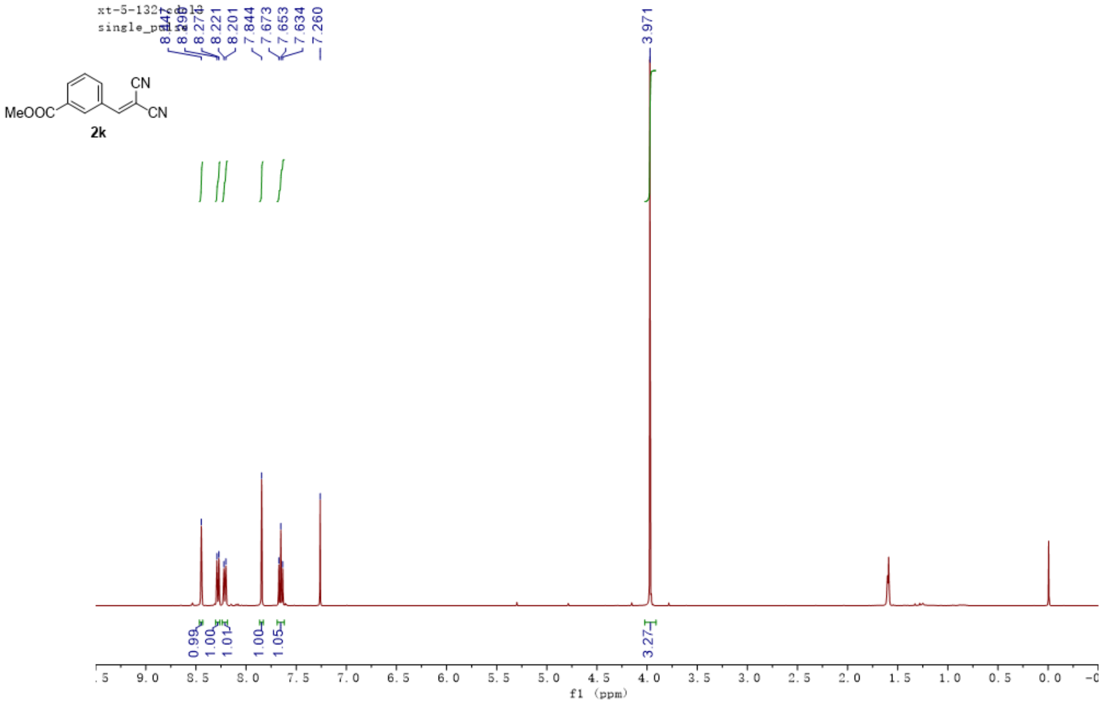

^{1}H NMR (400 MHz, $CDCl_3$) of **2k**

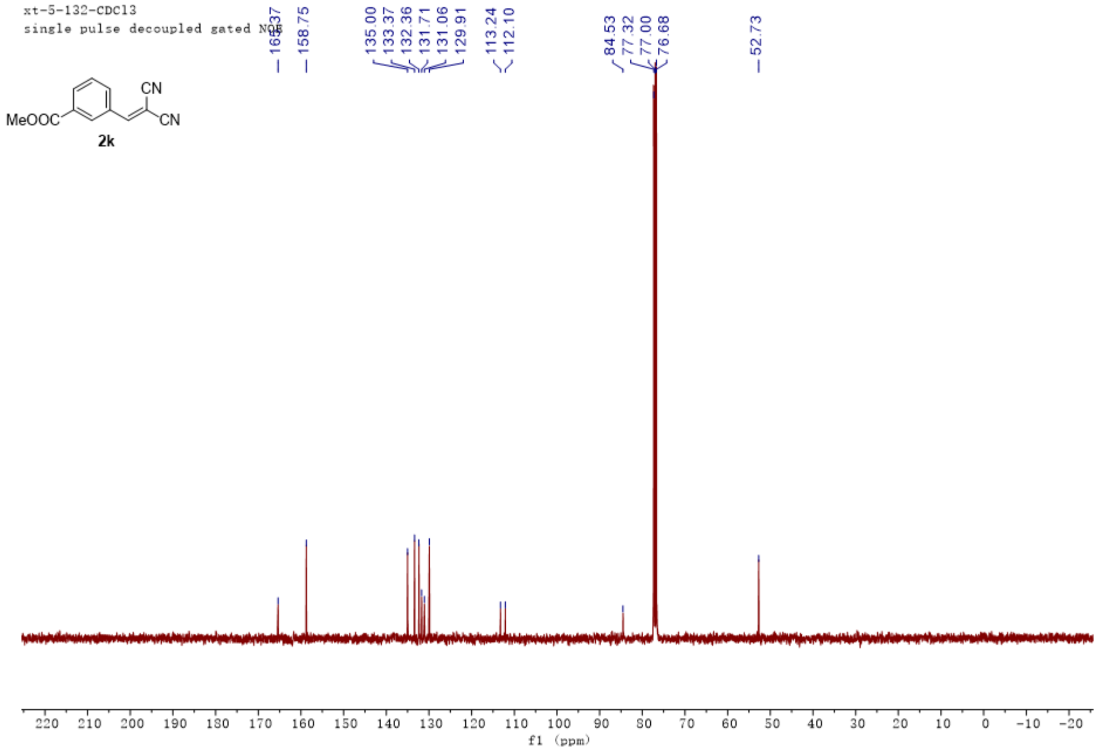

^{13}C NMR (101 MHz, $CDCl_3$) of **2k**

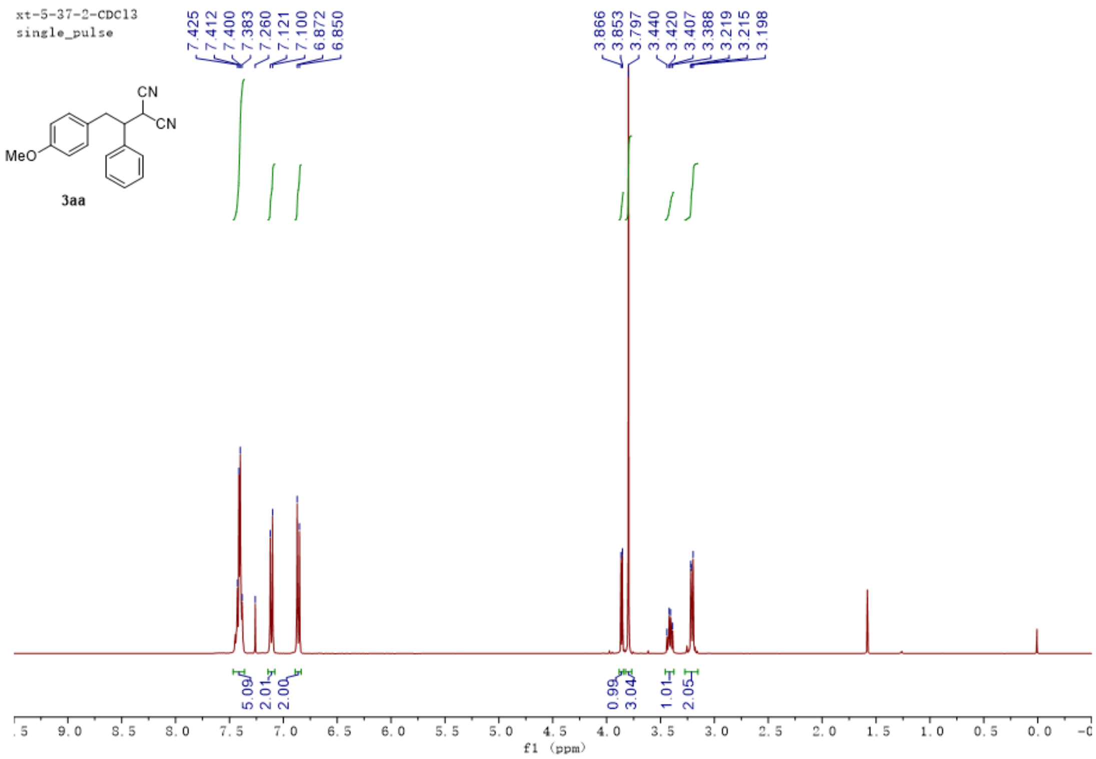

^{1}H NMR (400 MHz, $CDCl_3$) of **3aa**

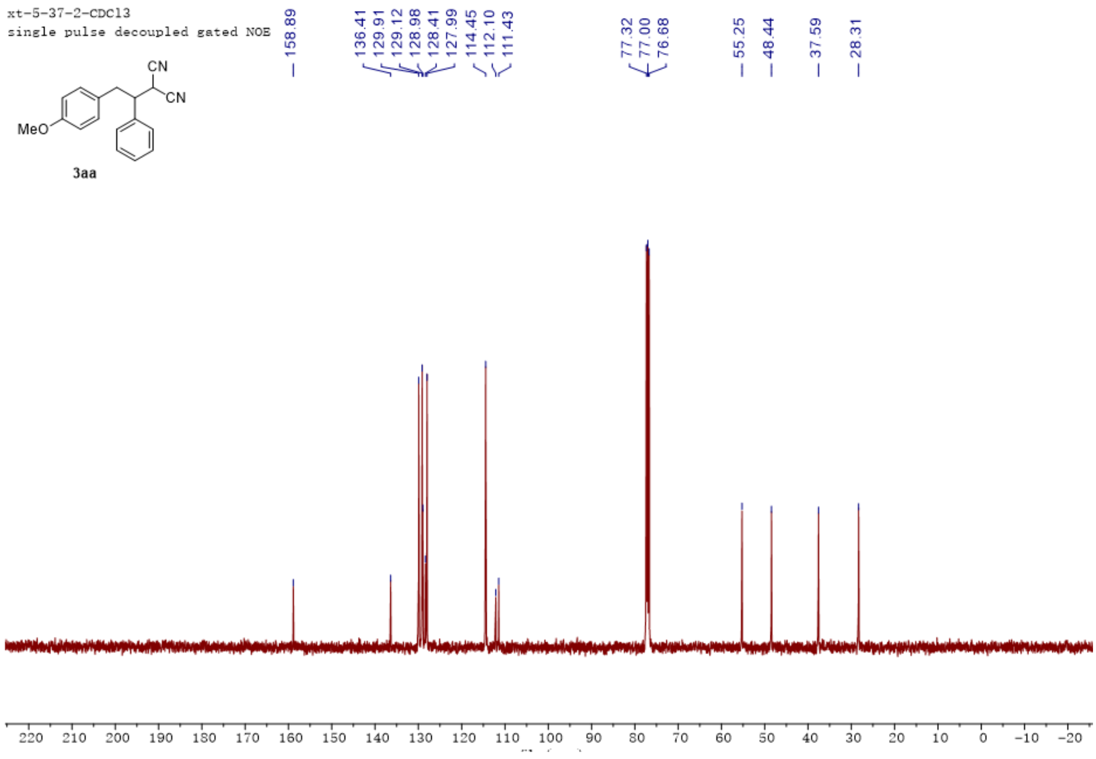

^{13}C NMR (101 MHz, $CDCl_3$) of **3aa**

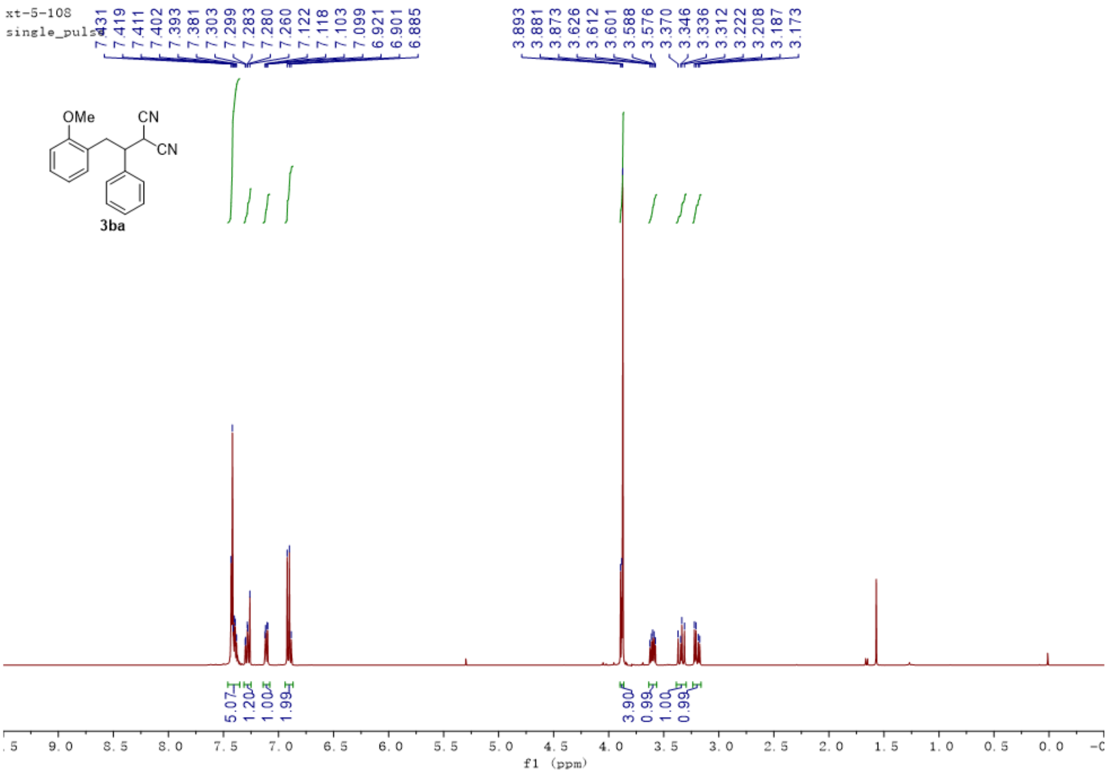

^{1}H NMR (400 MHz, $CDCl_3$) of **3ba**

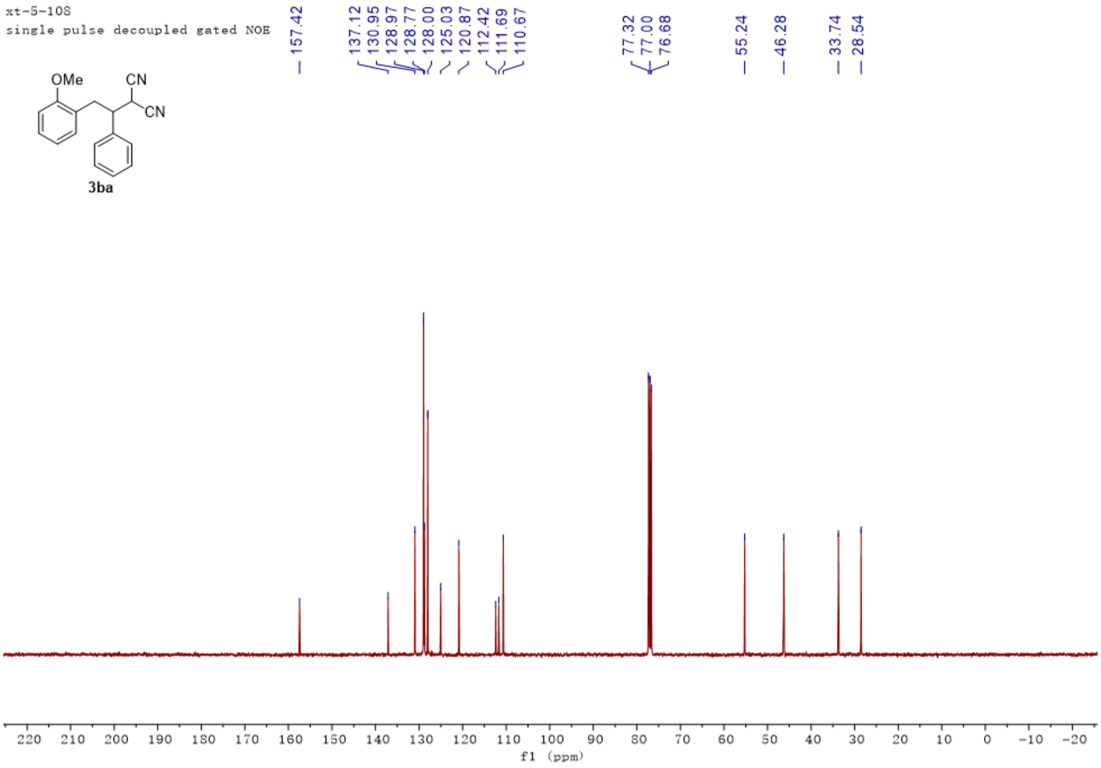

^{13}C NMR (101 MHz, $CDCl_3$) of **3aa**

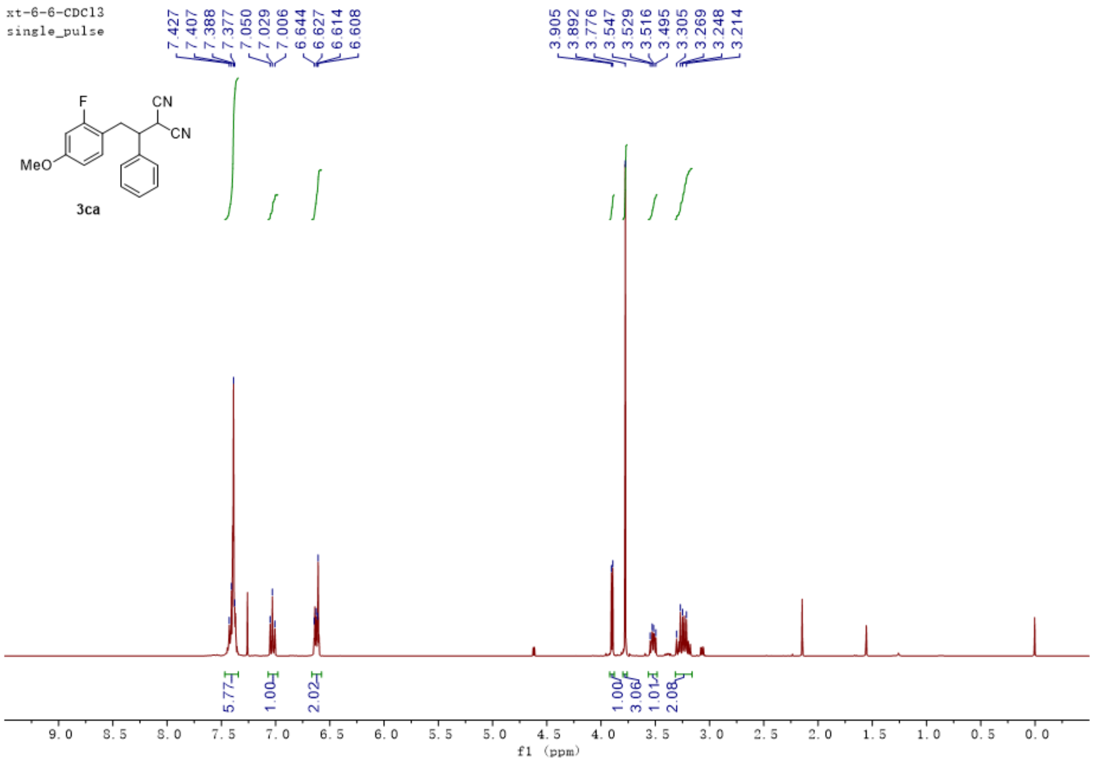

^{1}H NMR (400 MHz, $CDCl_3$) of **3ca**

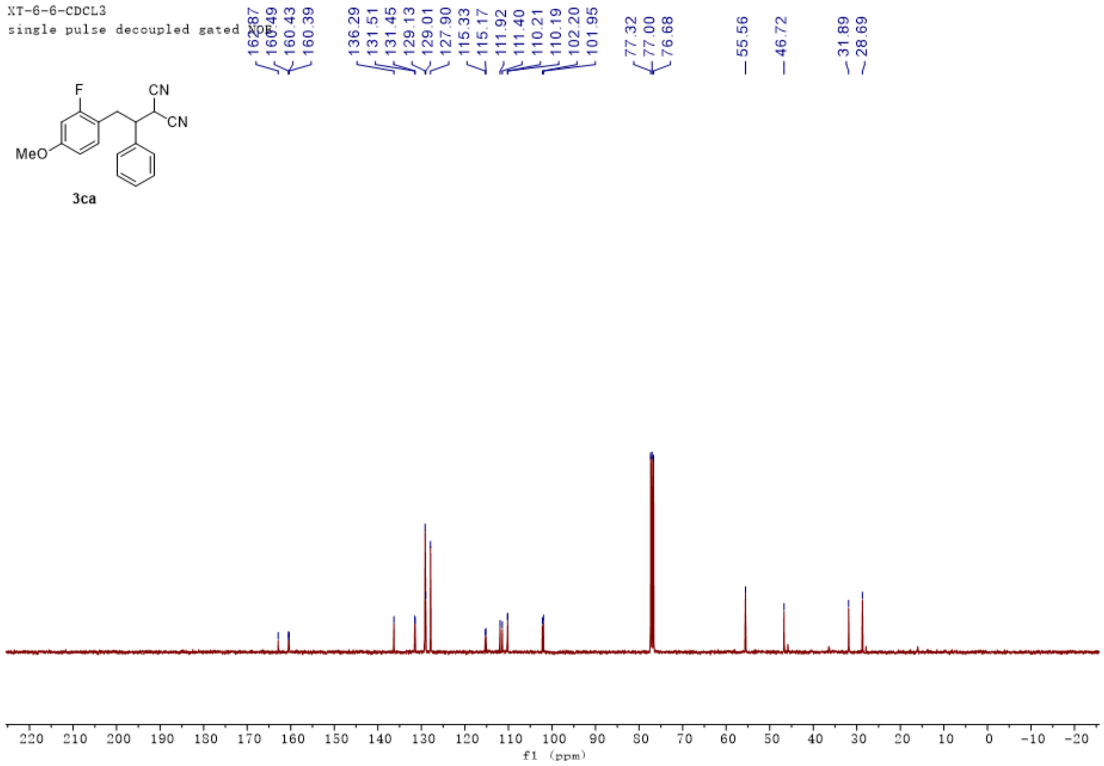

^{13}C NMR (101 MHz, $CDCl_3$) of **3ca**

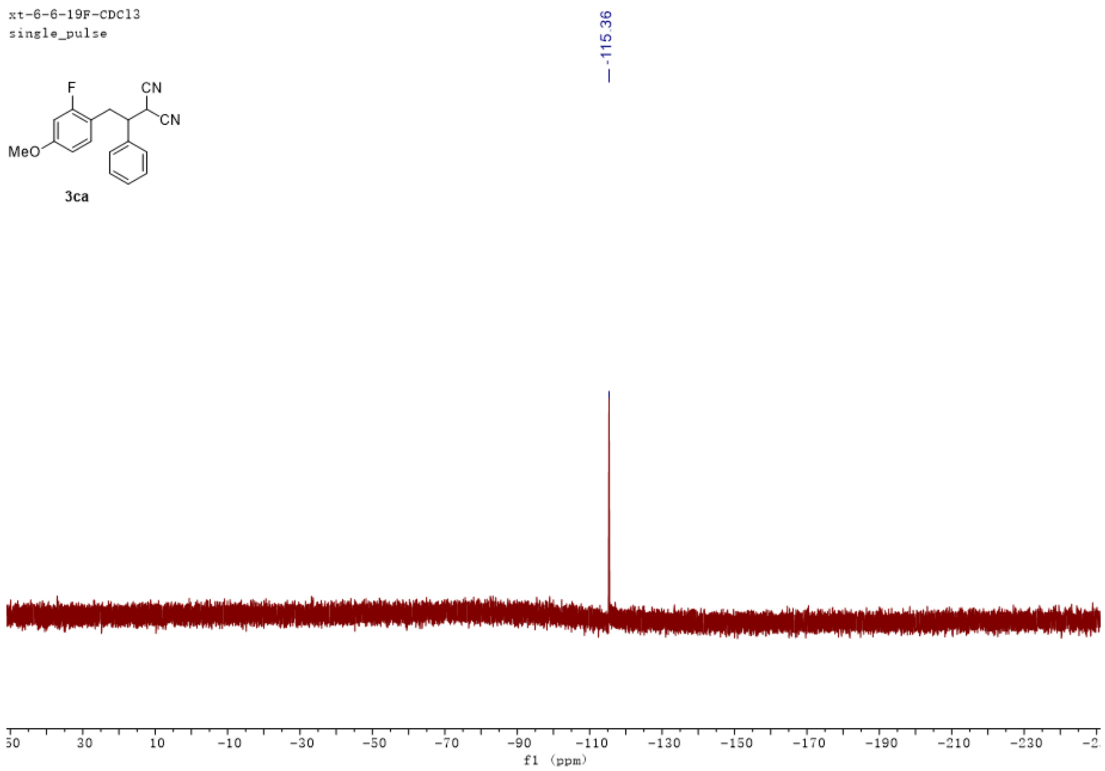

^{19}F NMR (376 MHz, $CDCl_3$) of **3ca**

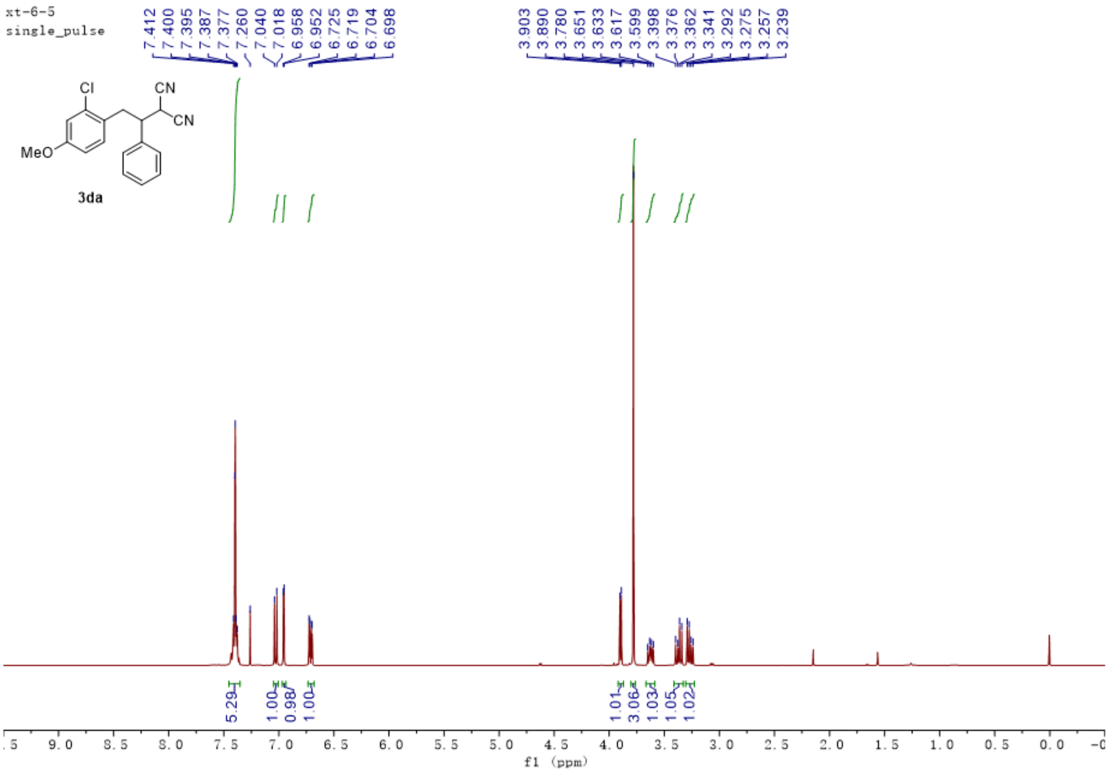

^{1}H NMR (400 MHz, $CDCl_3$) of **3da**

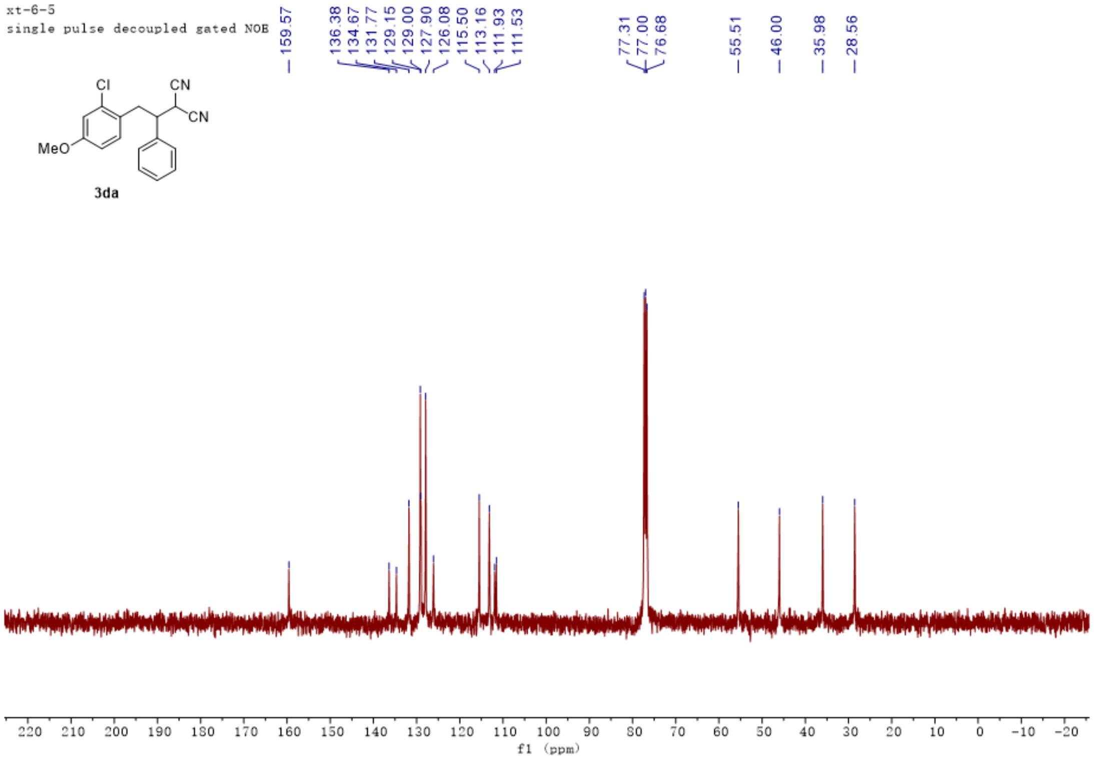

^{13}C NMR (101 MHz, $CDCl_3$) of **3da**

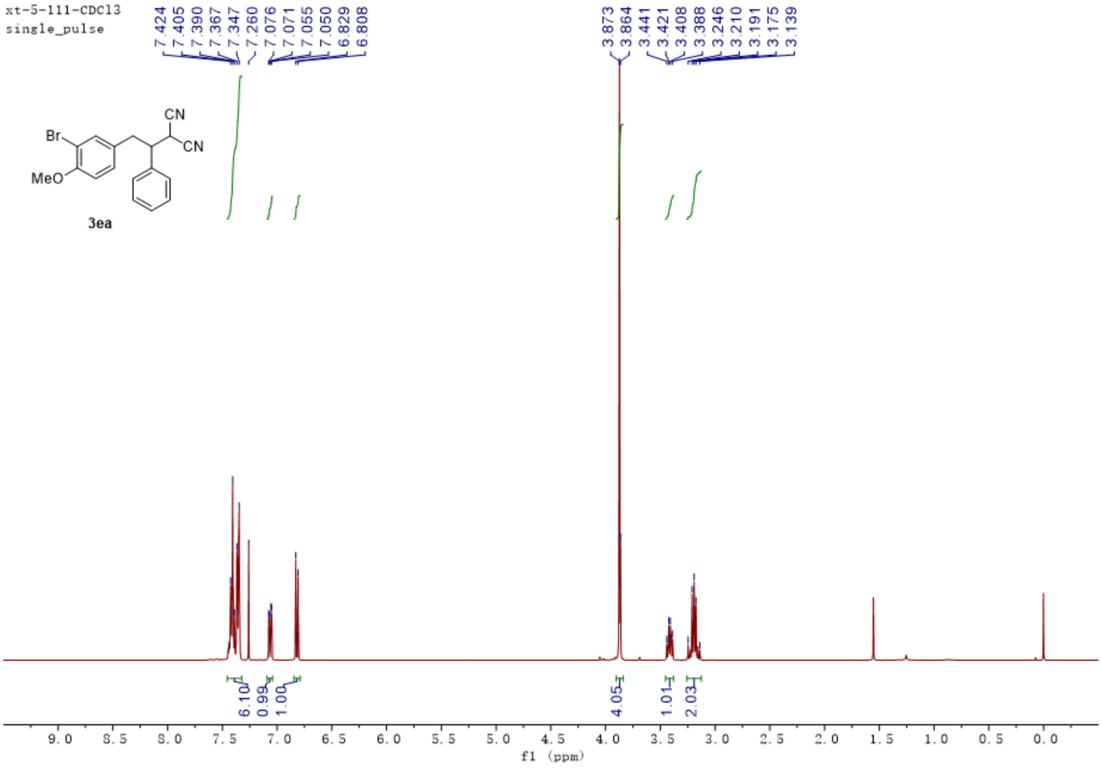

^{1}H NMR (400 MHz, $CDCl_3$) of **3ea**

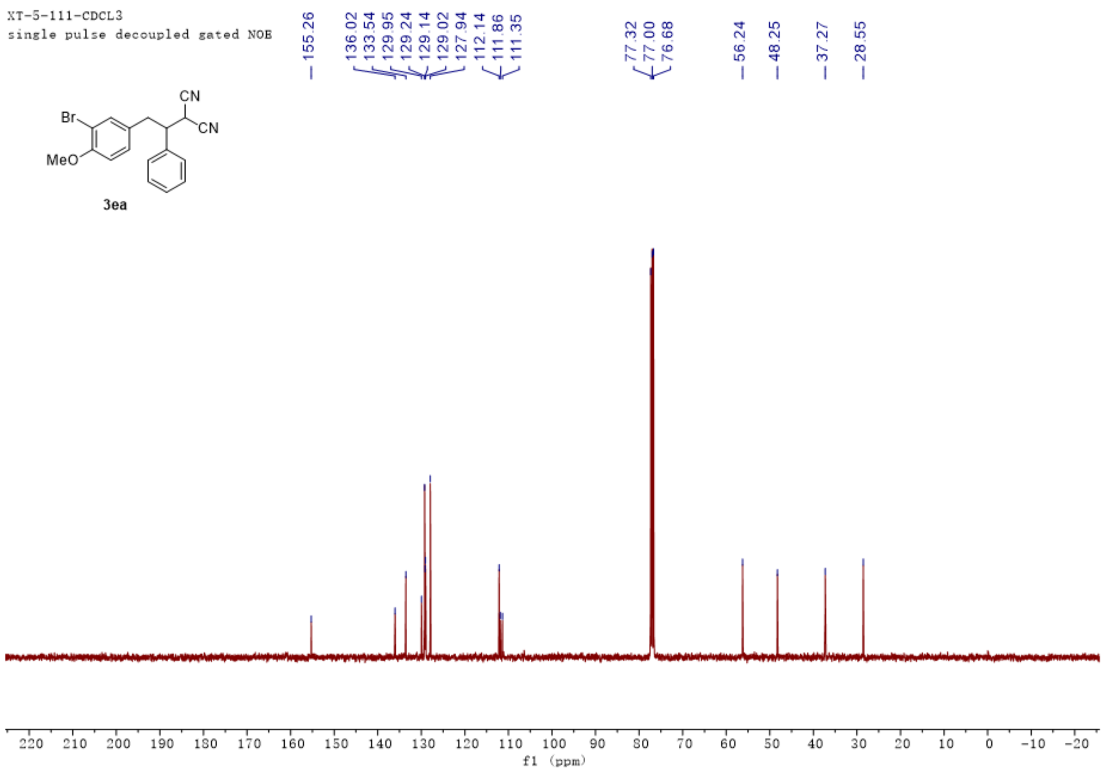

^{13}C NMR (101 MHz, $CDCl_3$) of **3ea**

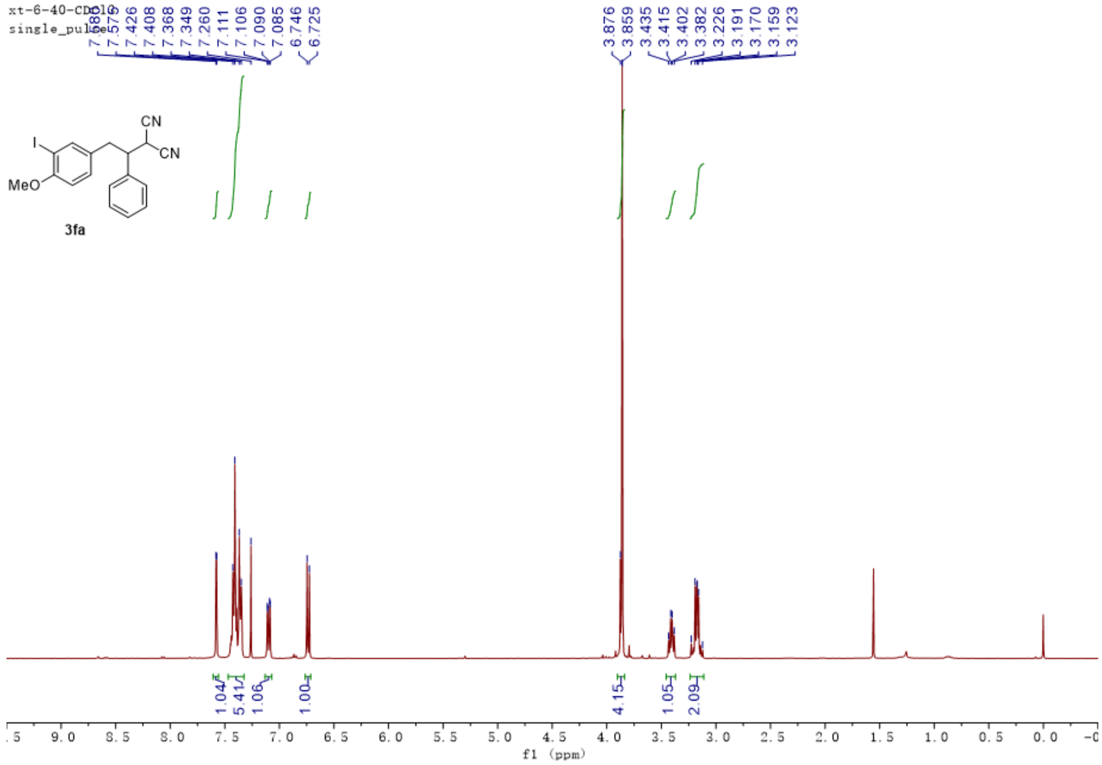

^{1}H NMR (400 MHz, $CDCl_3$) of **3fa**

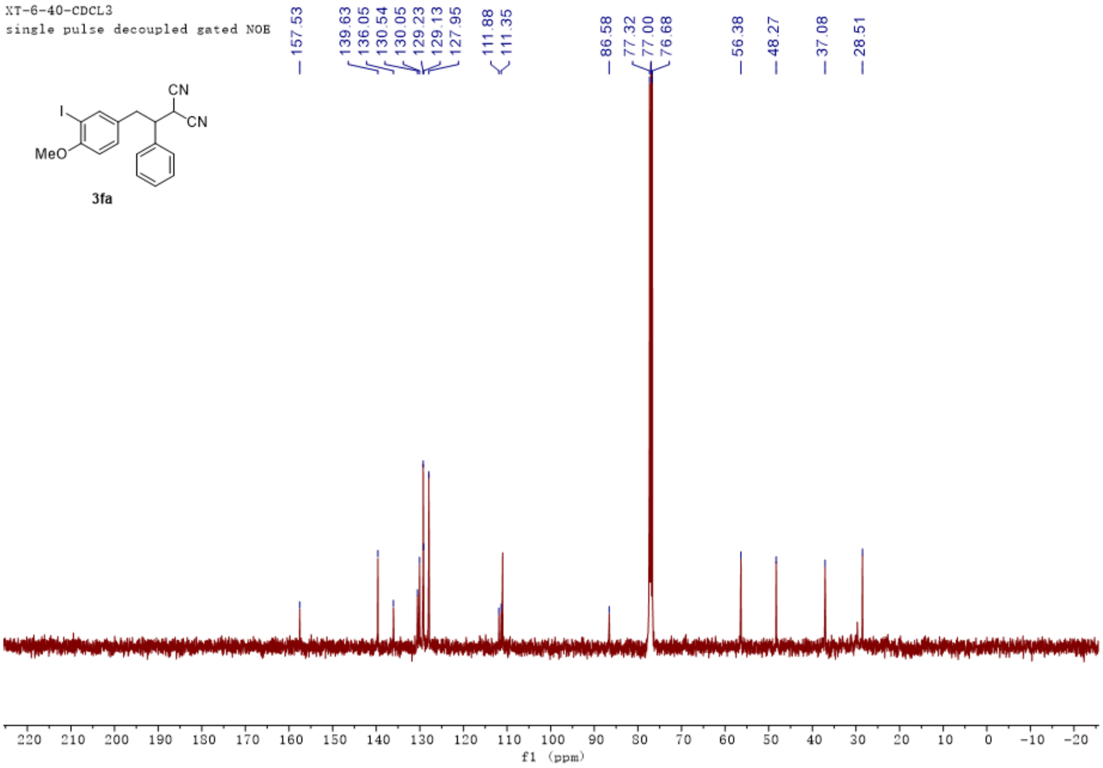

^{13}C NMR (101 MHz, $CDCl_3$) of **3fa**

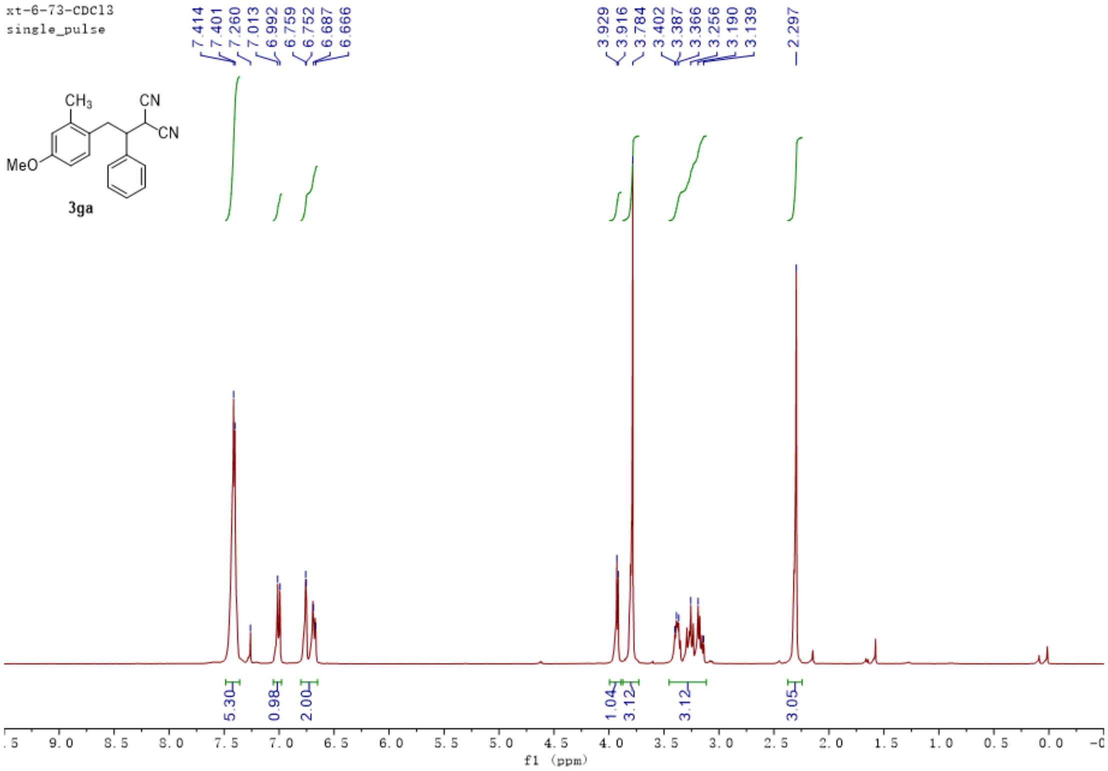

^{1}H NMR (400 MHz, $CDCl_3$) of **3ga**

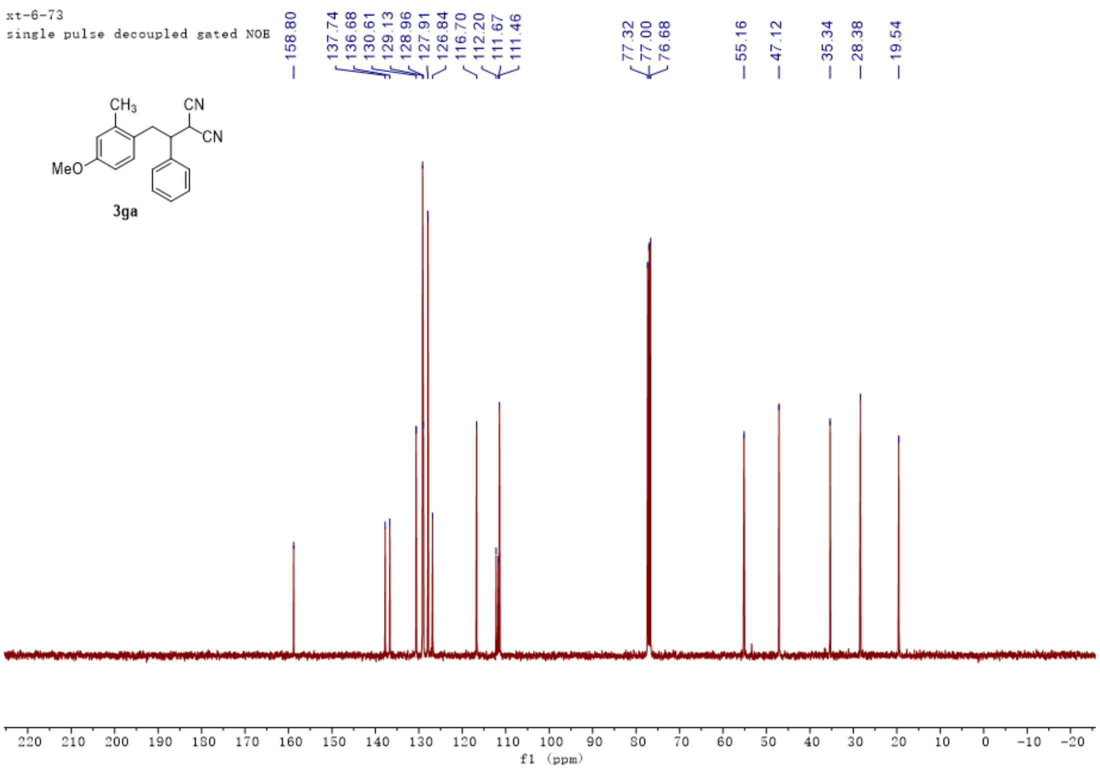

^{13}C NMR (101 MHz, CDCl$_3$) of **3ga**

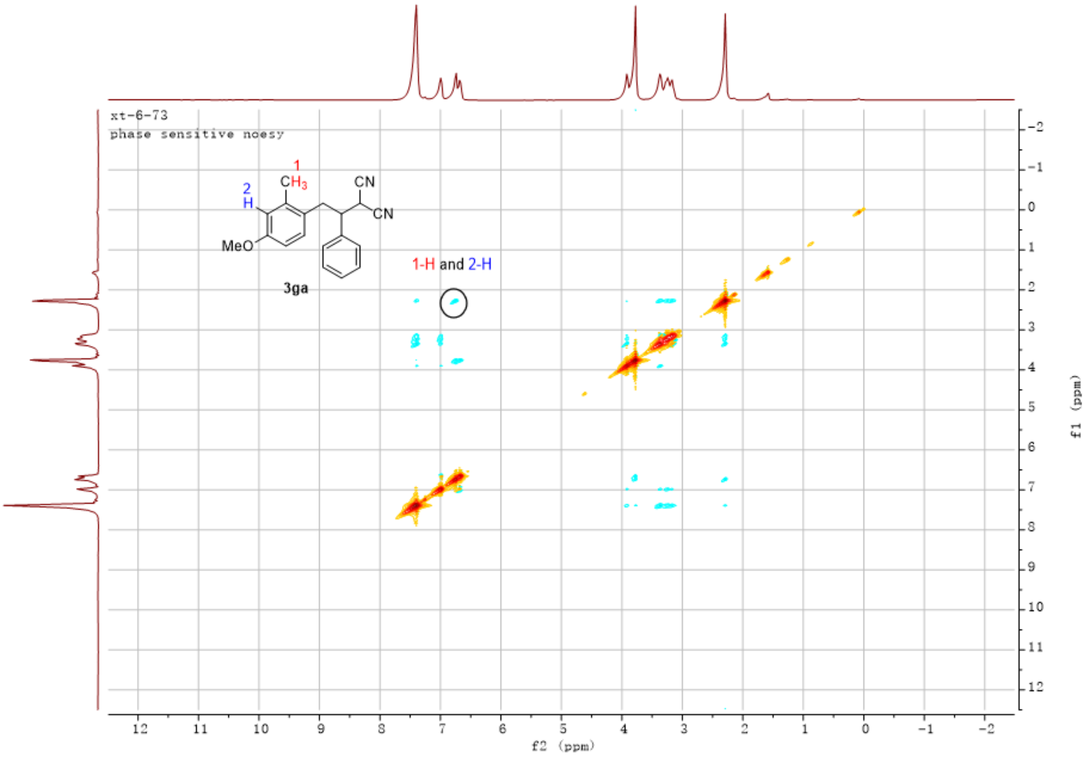

Nuclear Overhauser effect (NOESY) spectroscopy **3ga**

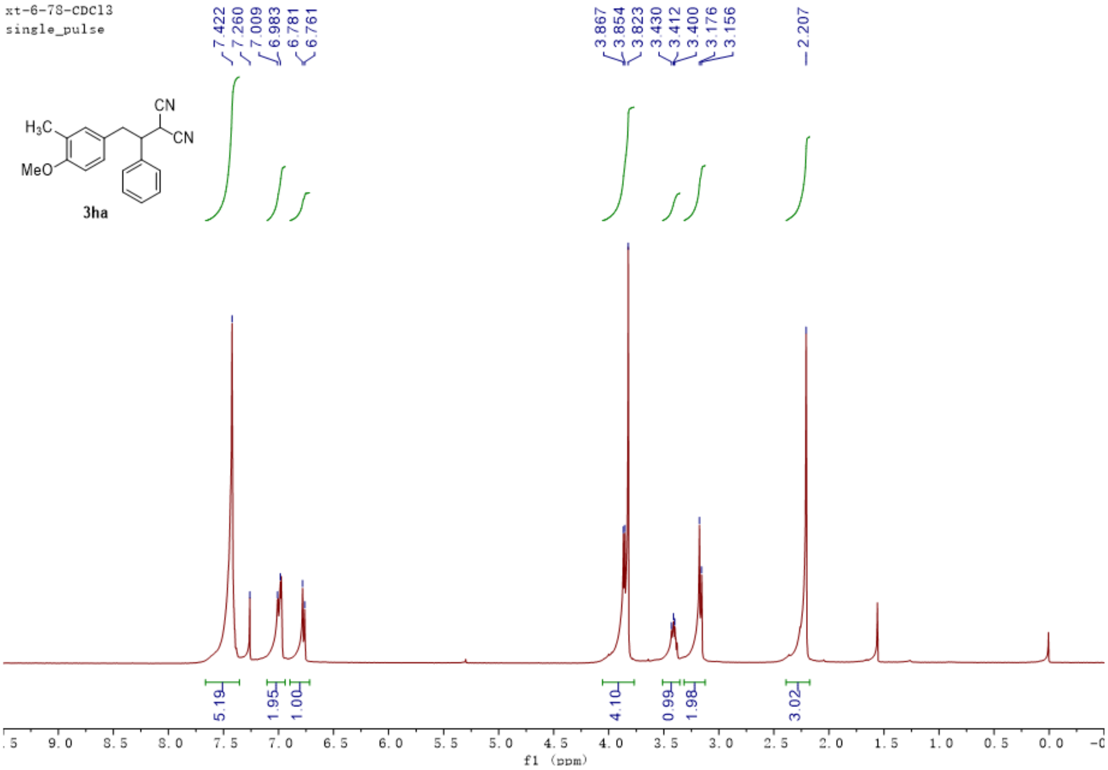

^{1}H NMR (400 MHz, $CDCl_3$) of **3ha**

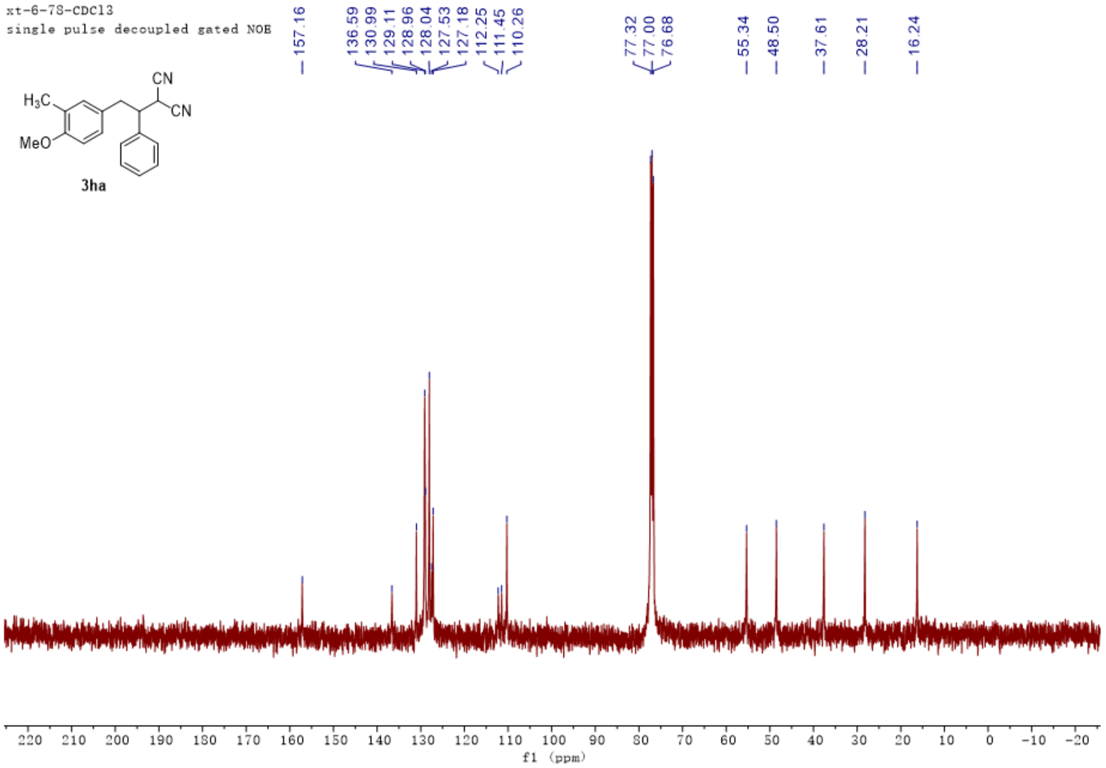

^{13}C NMR (101 MHz, $CDCl_3$) of **3ha**

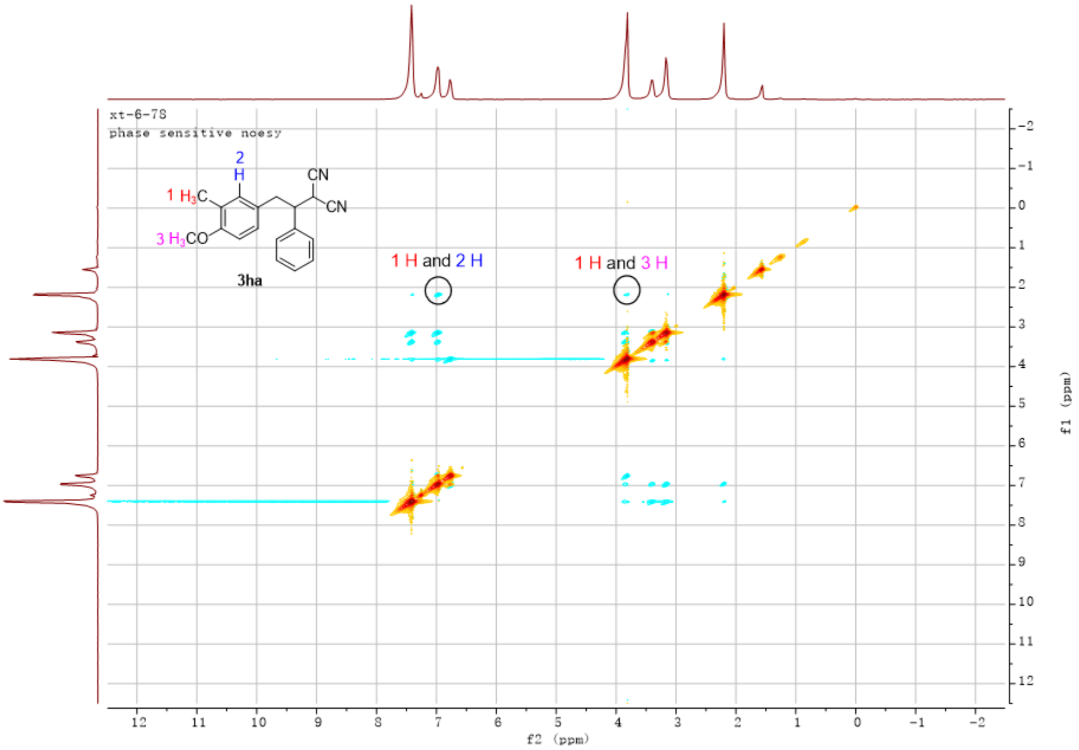

Nuclear Overhauser effect (NOESY) spectroscopy **3ha**

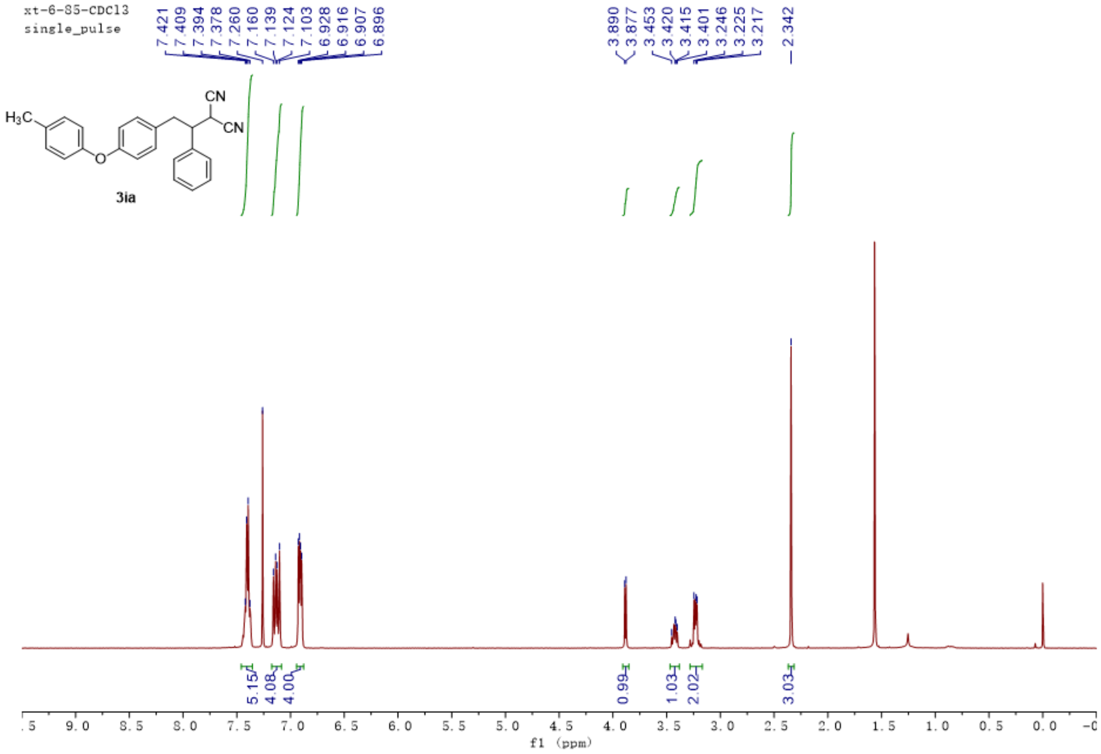

^{1}H NMR (400 MHz, $CDCl_3$) of **3ia**

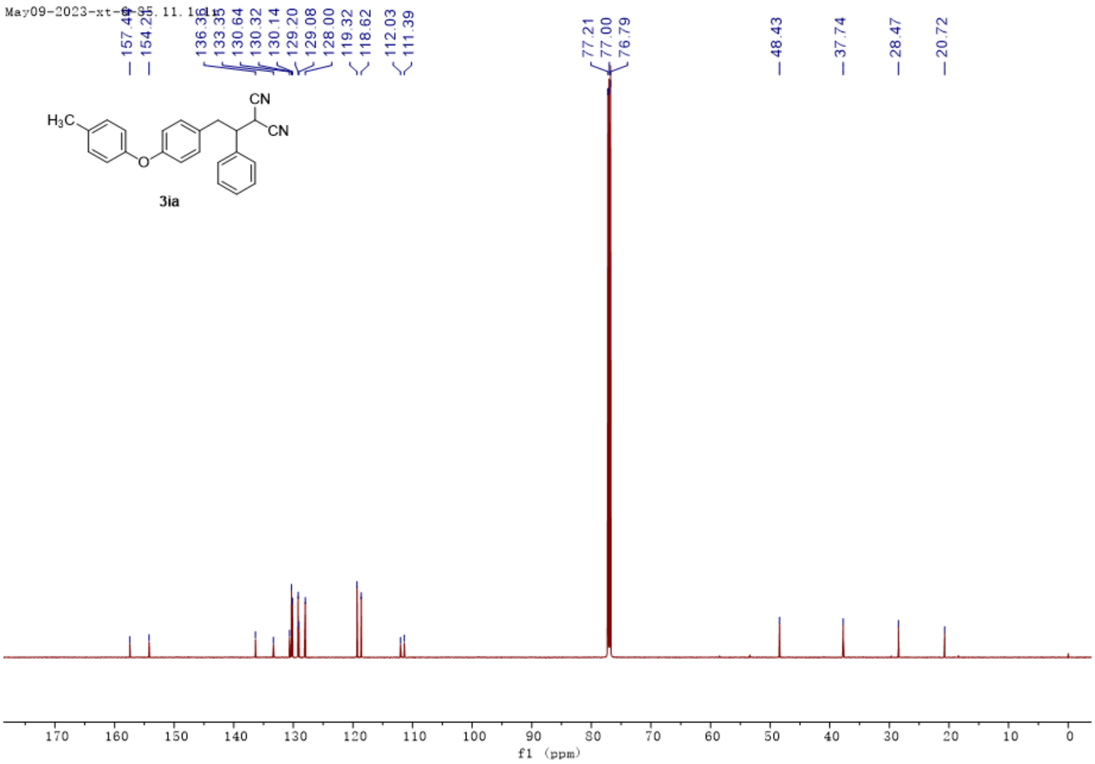

^{13}C NMR (151 MHz, $CDCl_3$) of **3ia**

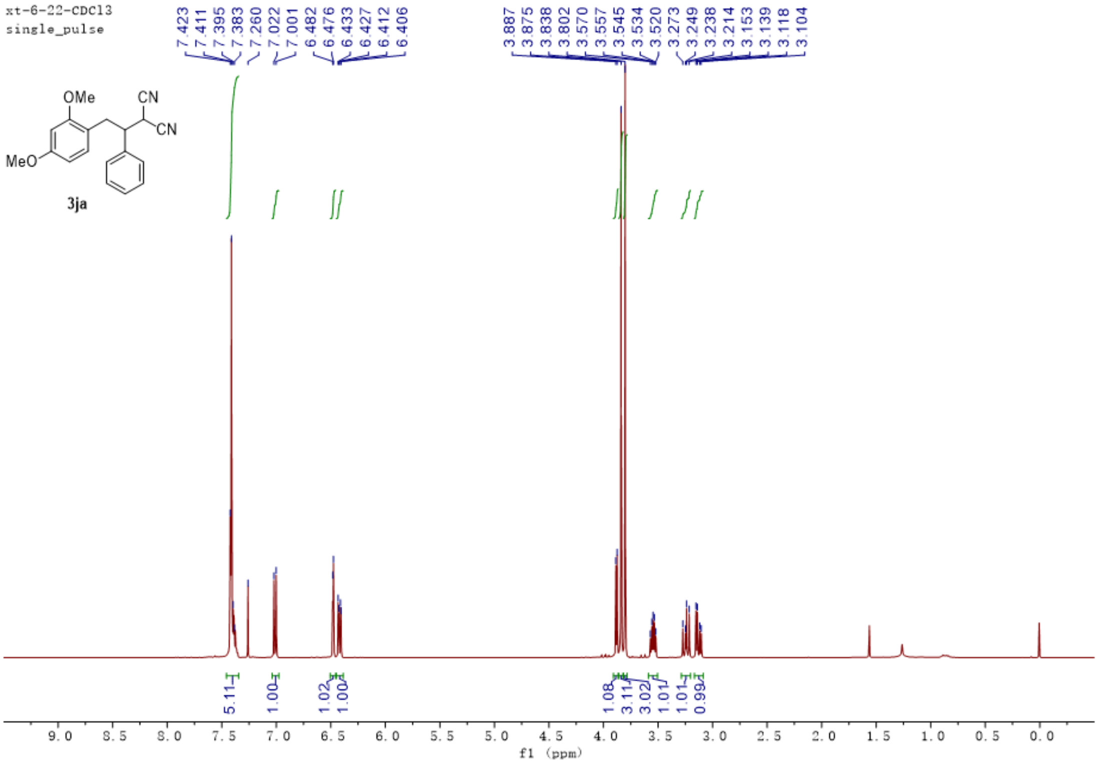

^{1}H NMR (400 MHz, $CDCl_3$) of **3ja**

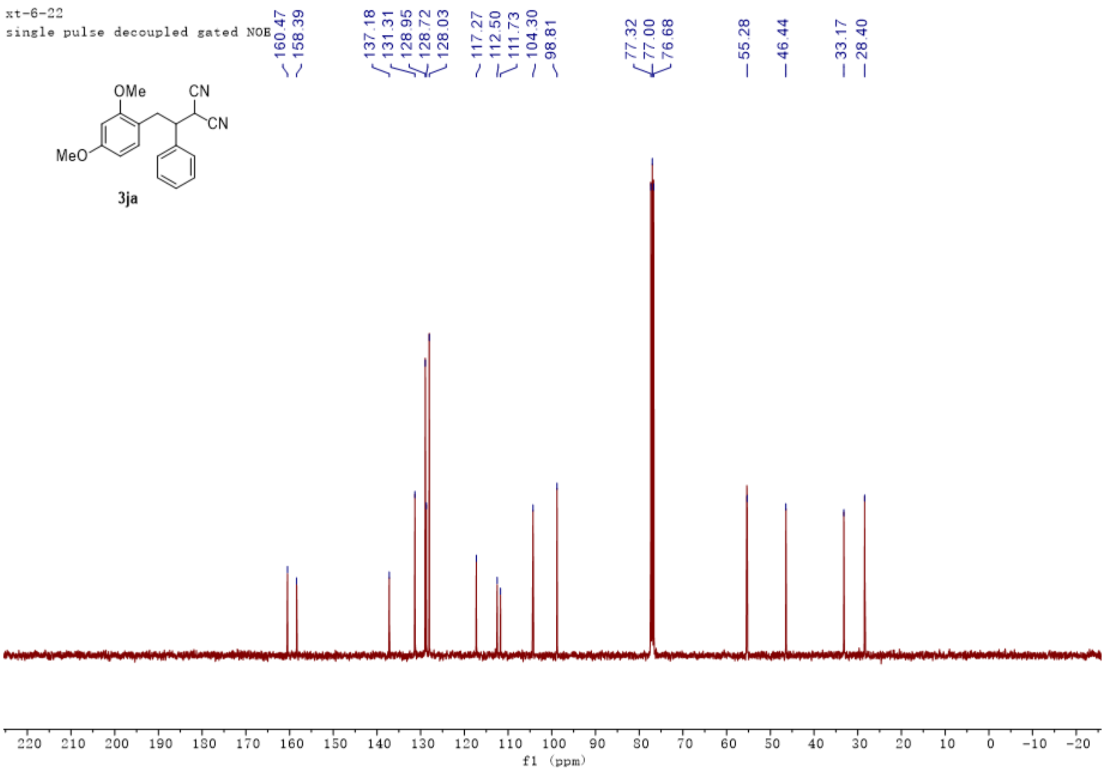

^{13}C NMR (101 MHz, CDCl$_3$) of **3ja**

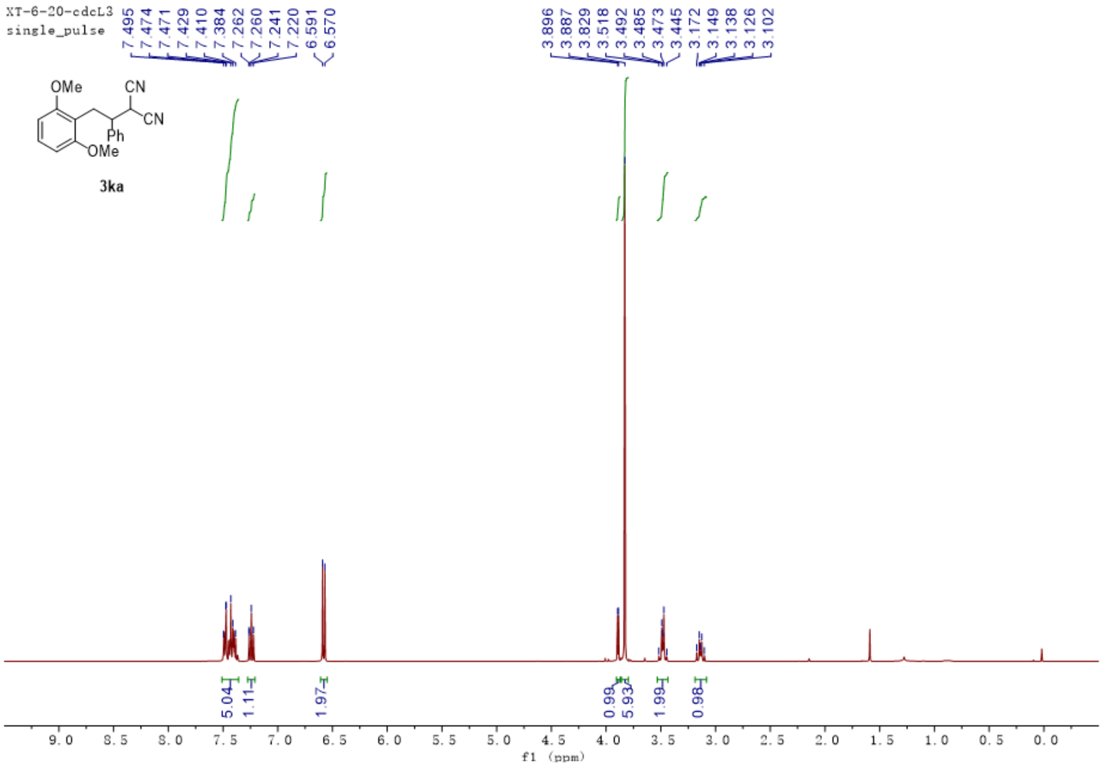

^{1}H NMR (400 MHz, CDCl$_3$) of **3ka**

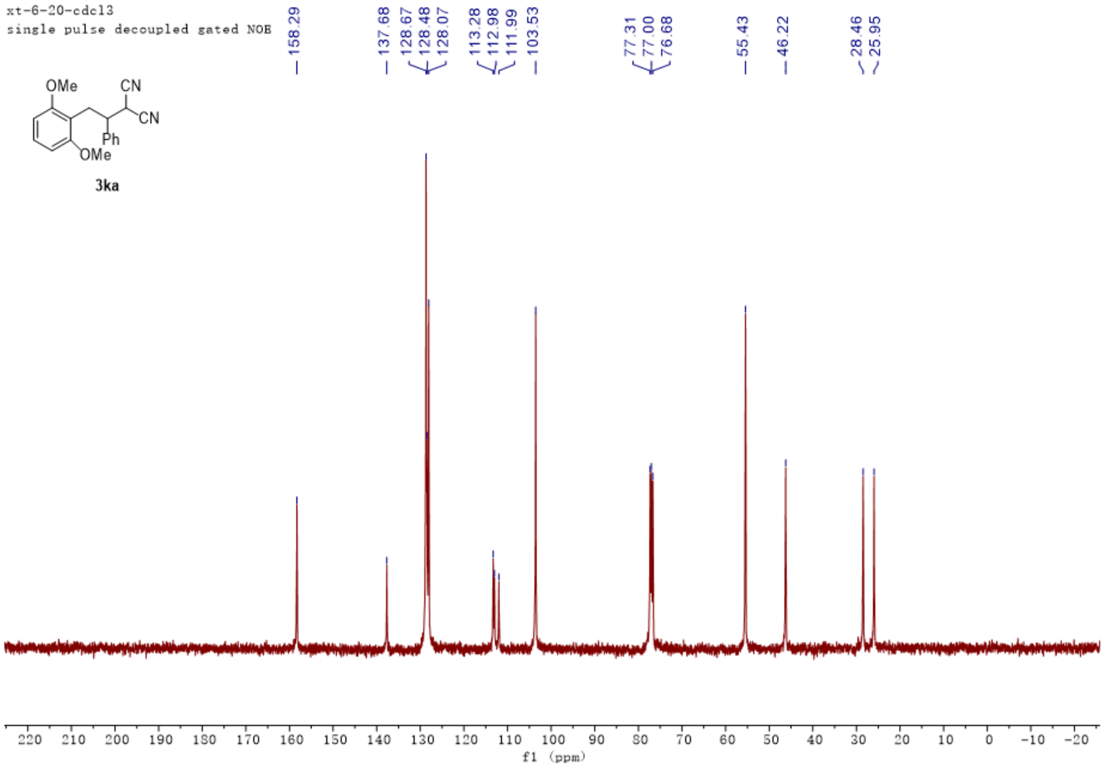

^{13}C NMR (101 MHz, $CDCl_3$) of **3ka**

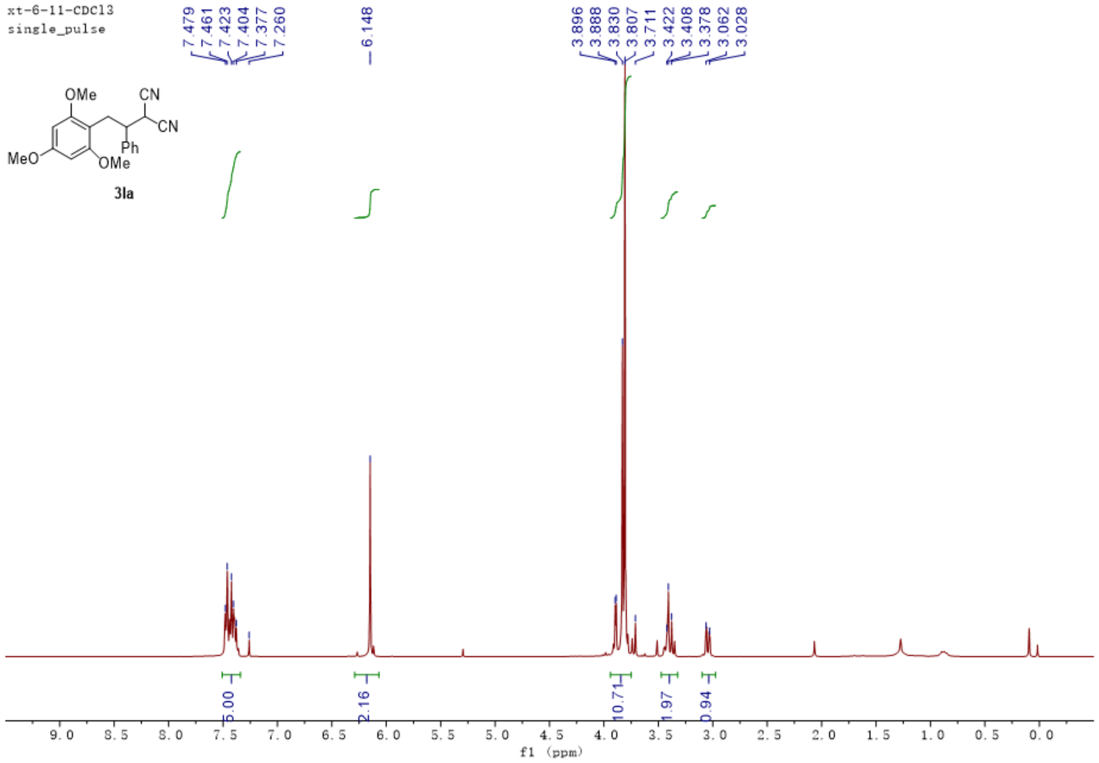

^{1}H NMR (400 MHz, $CDCl_3$) of **3la**

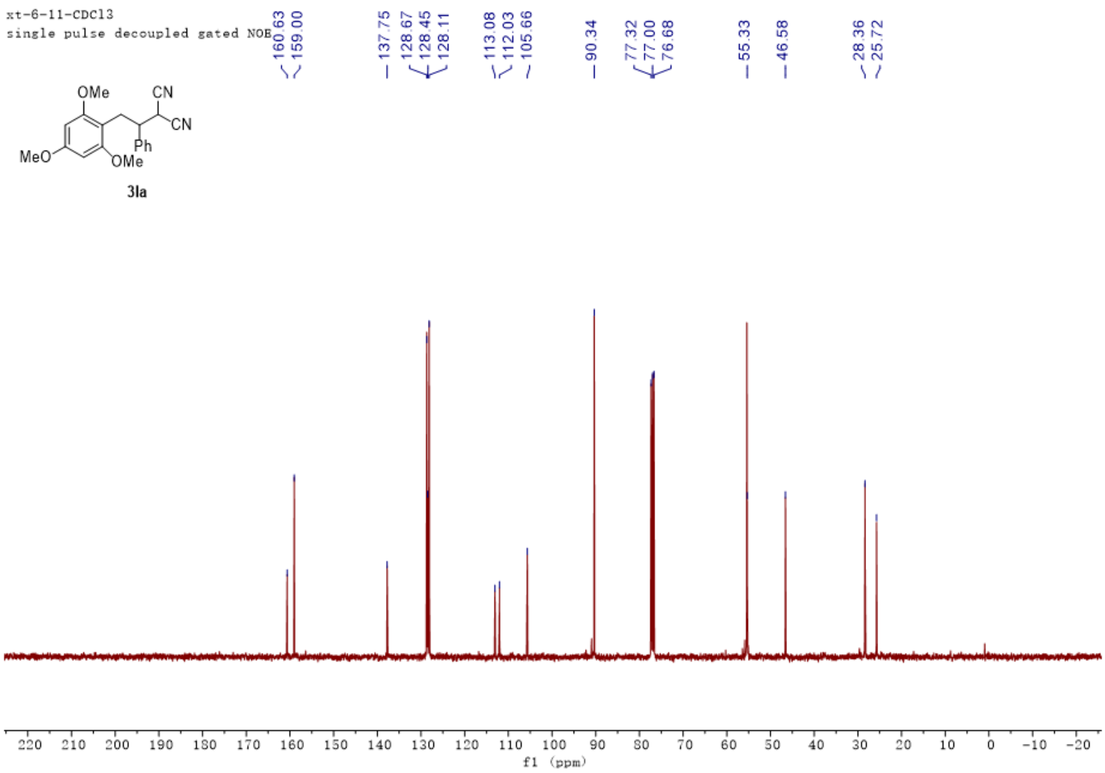

^{13}C NMR (101 MHz, CDCl$_3$) of **3la**

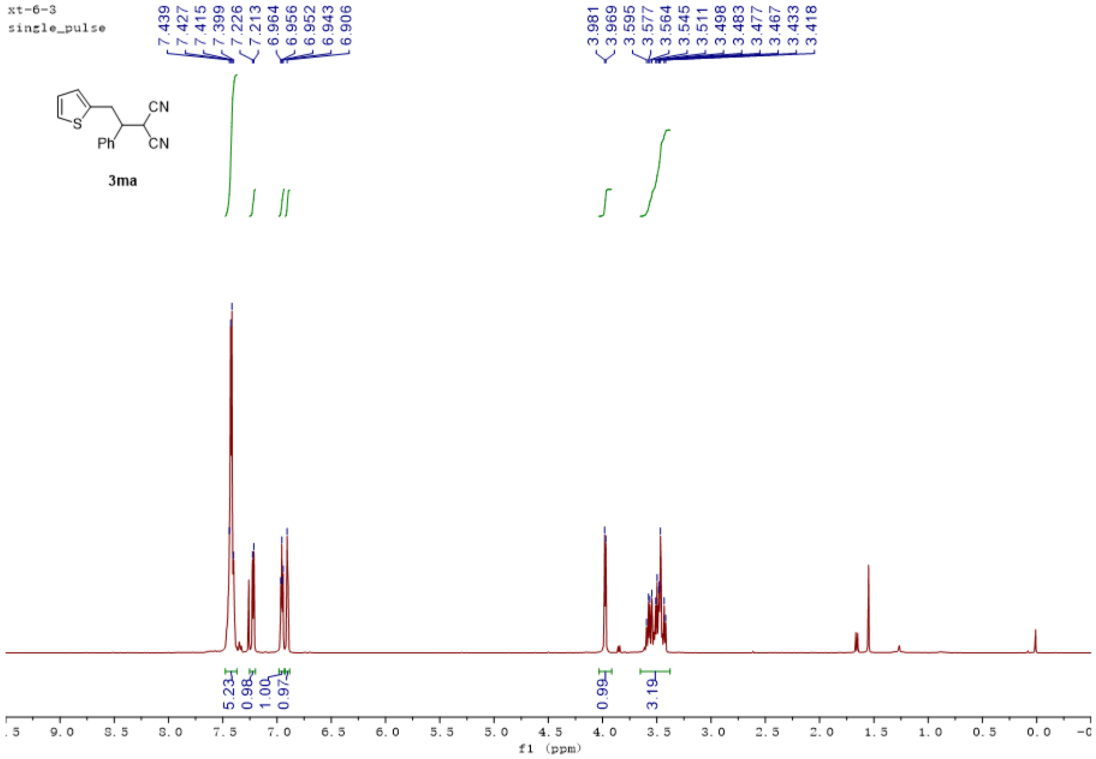

^{1}H NMR (400 MHz, CDCl$_3$) of **3ma**

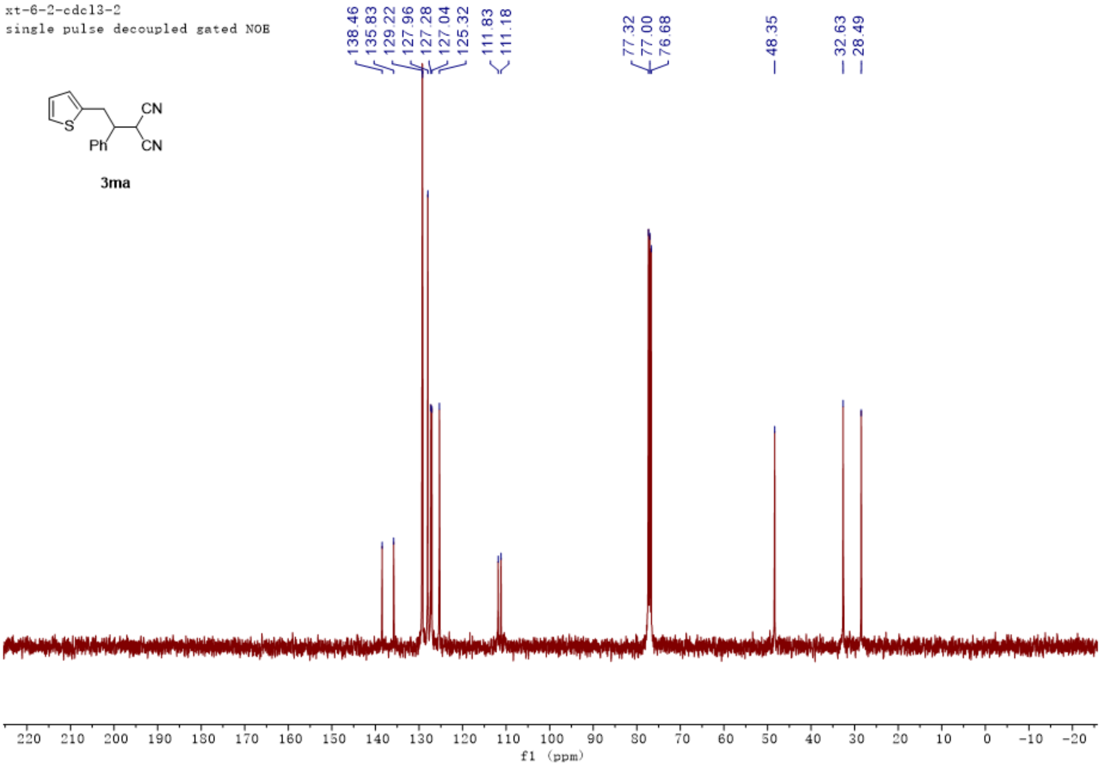

^{13}C NMR (101 MHz, $CDCl_3$) of **3ma**

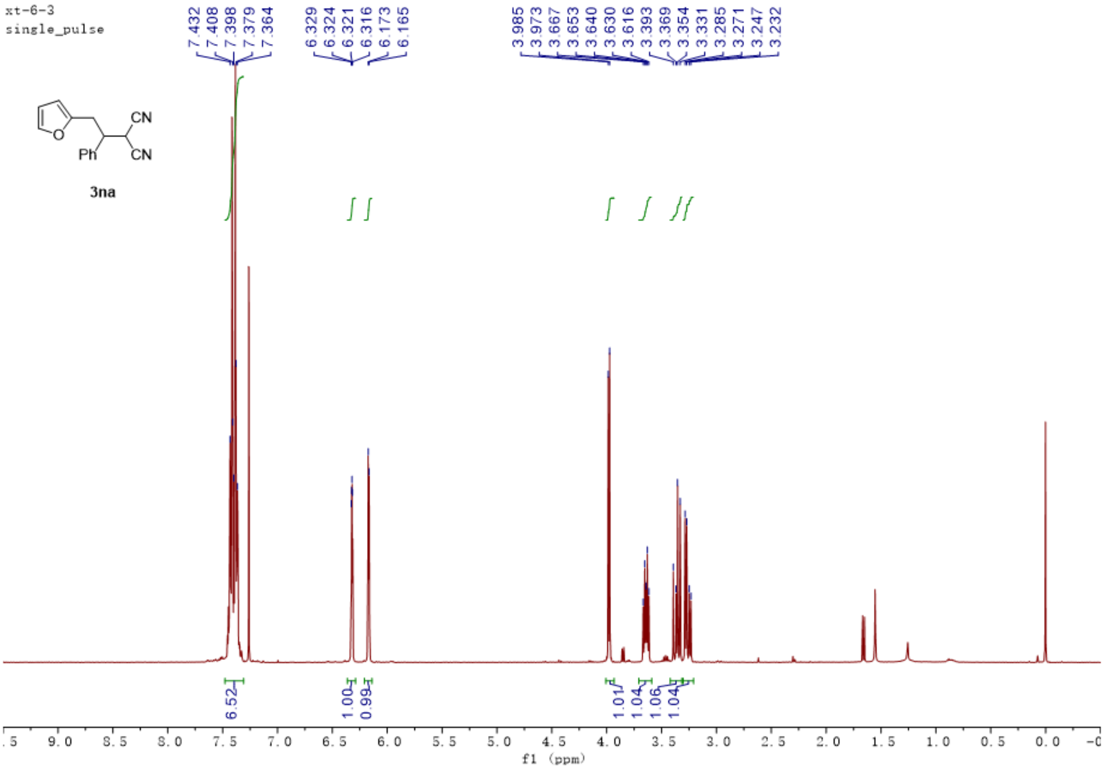

^{1}H NMR (400 MHz, $CDCl_3$) of **3na**

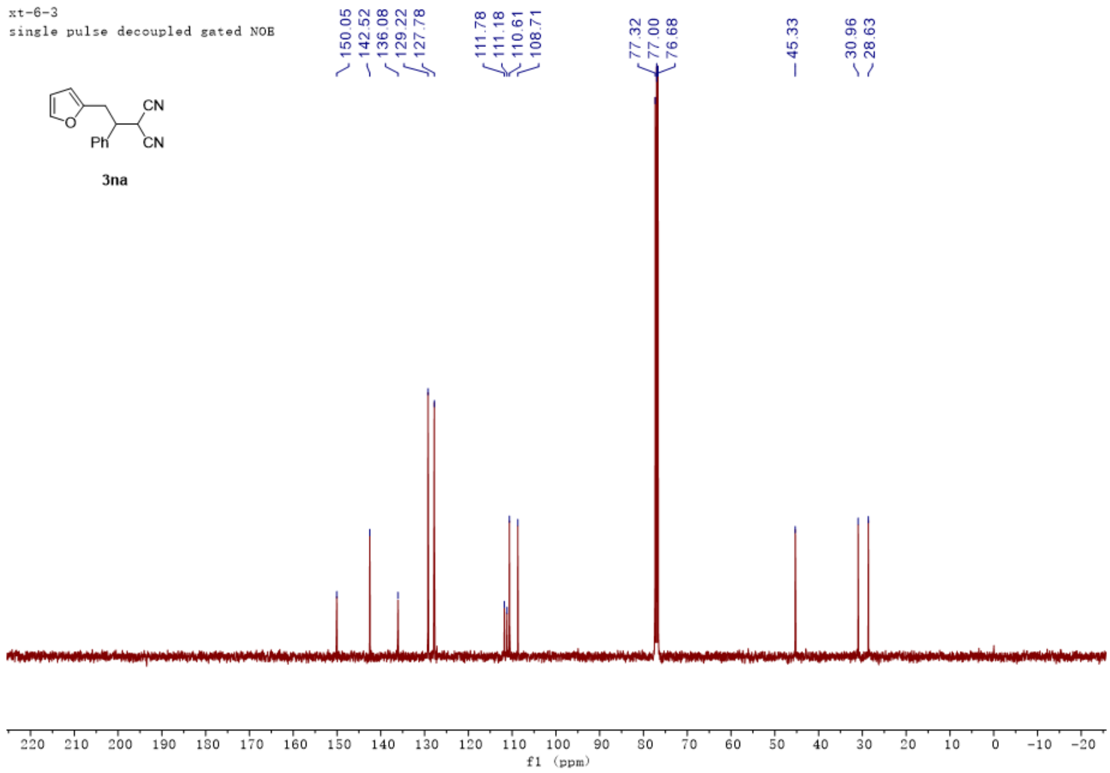

^{13}C NMR (101 MHz, $CDCl_3$) of **3na**

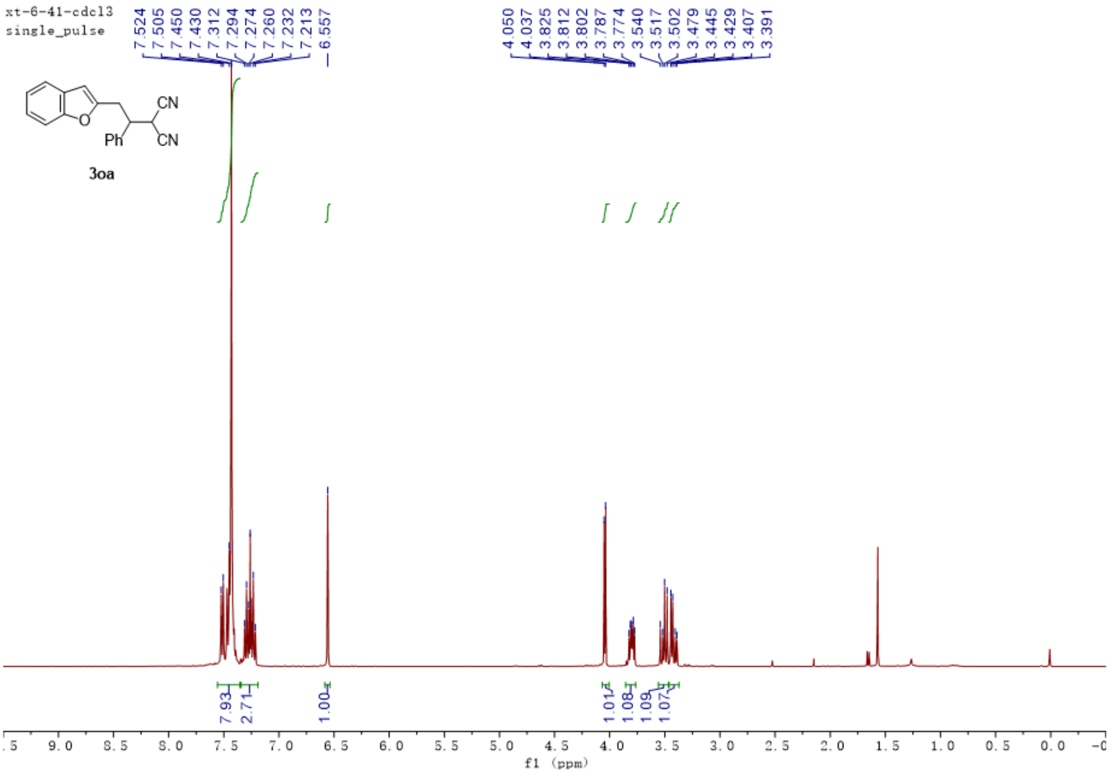

^{1}H NMR (400 MHz, $CDCl_3$) of **3oa**

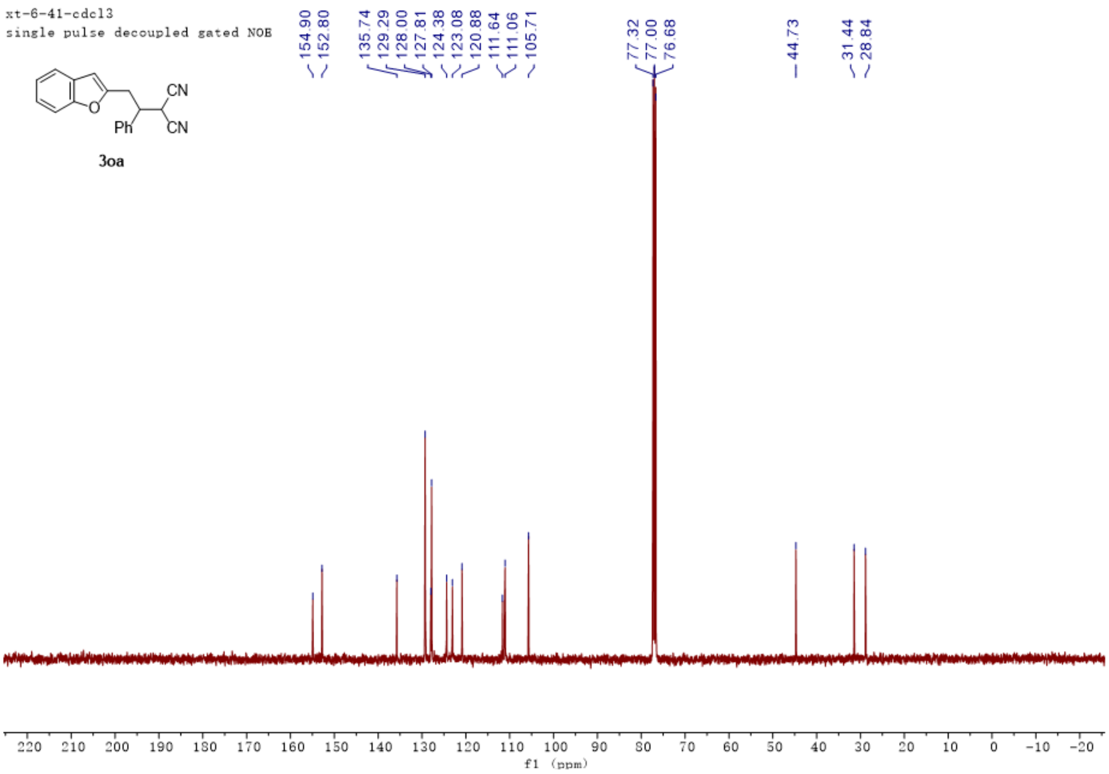

^{13}C NMR (101 MHz, $CDCl_3$) of **3oa**

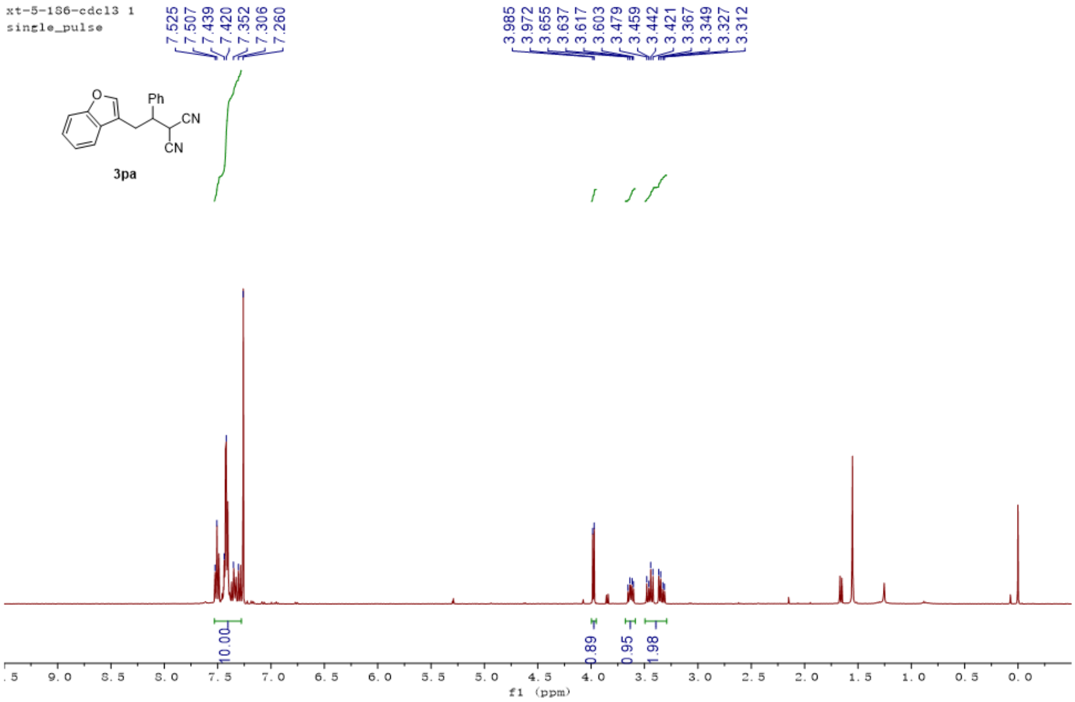

^{1}H NMR (400 MHz, $CDCl_3$) of **3pa**

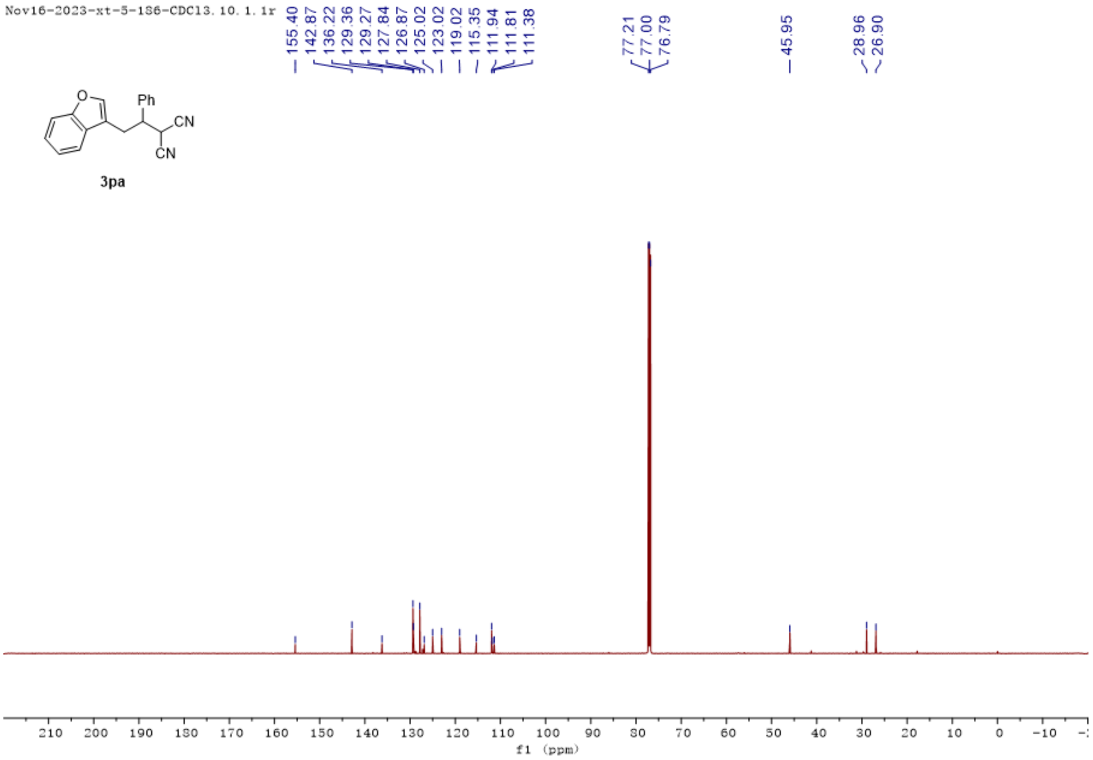

^{13}C NMR (101 MHz, CDCl$_3$) of **3pa**

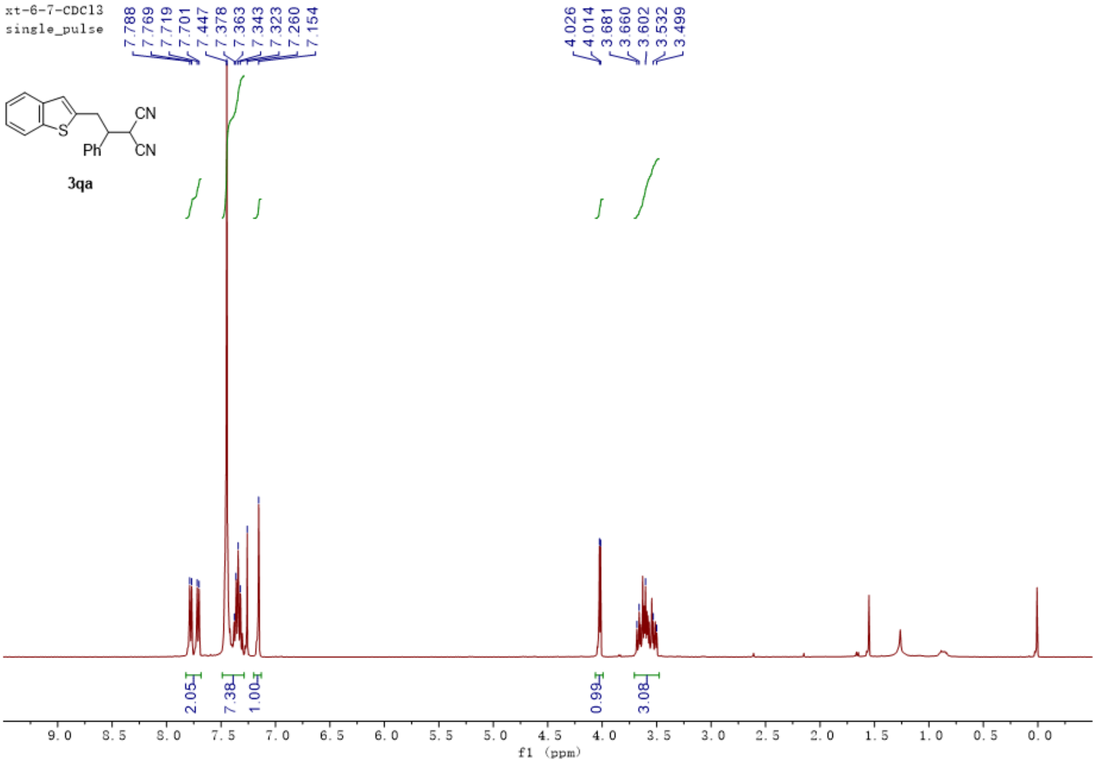

^{1}H NMR (400 MHz, CDCl$_3$) of **3qa**

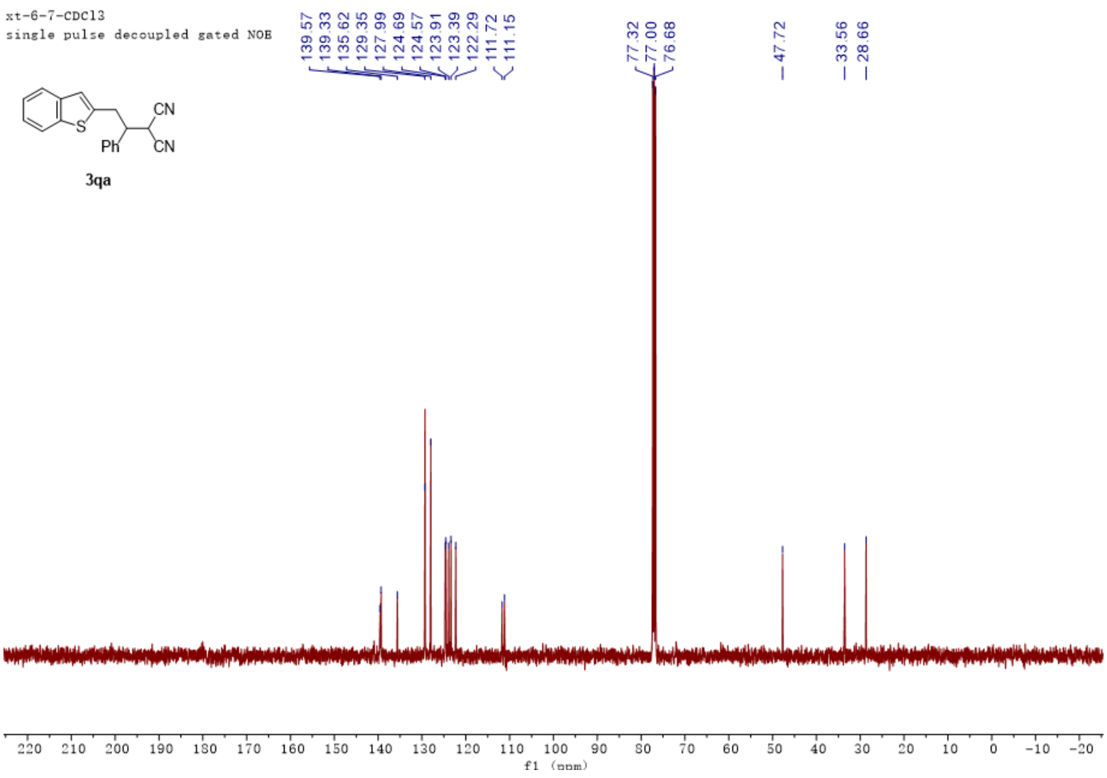

^{13}C NMR (101 MHz, $CDCl_3$) of **3qa**

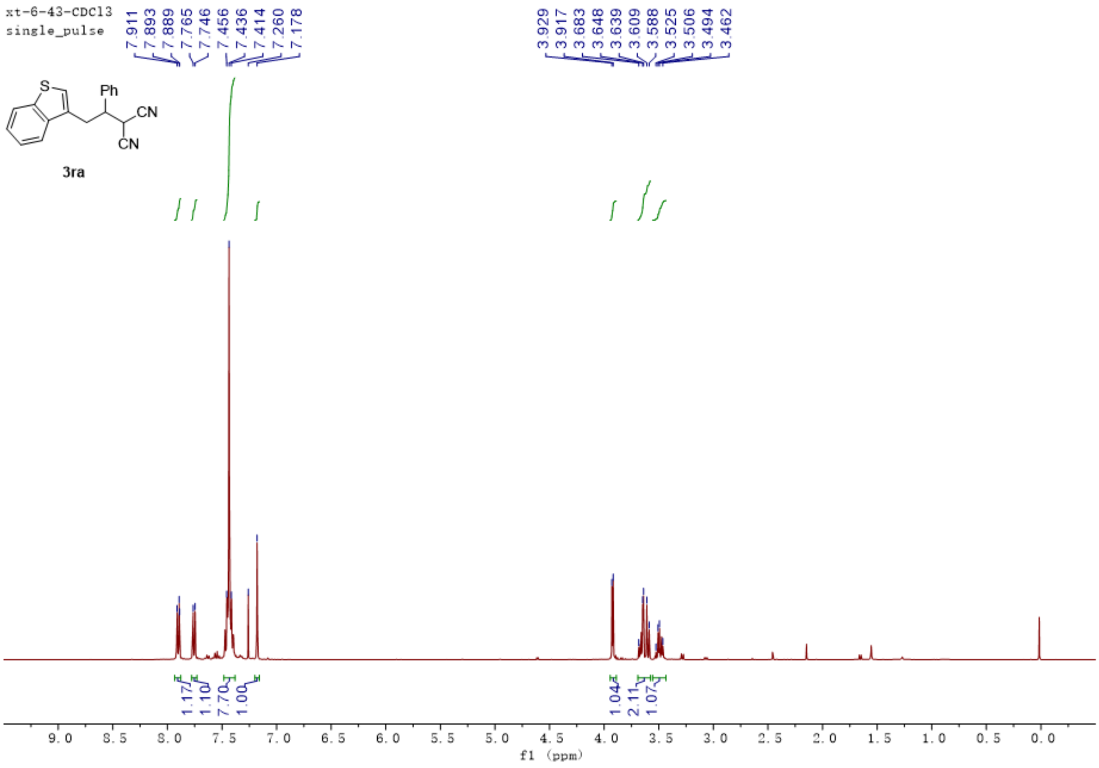

^{1}H NMR (400 MHz, $CDCl_3$) of **3ra**

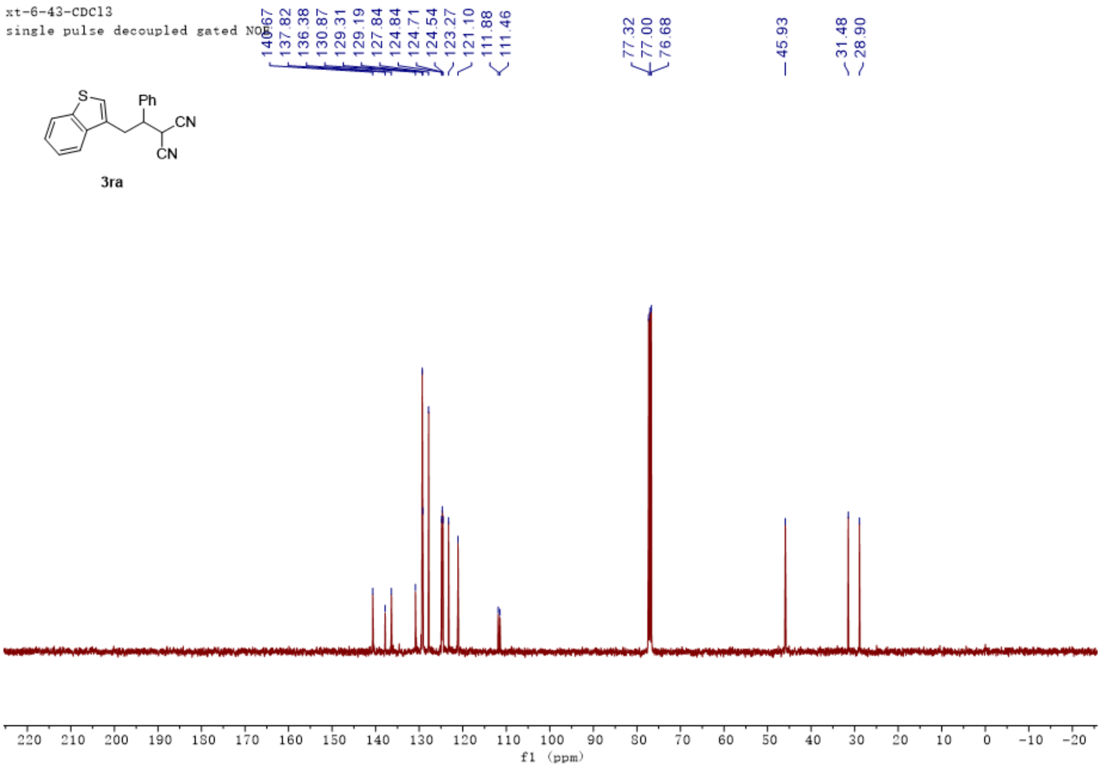

^{13}C NMR (101 MHz, CDCl$_3$) of **3ra**

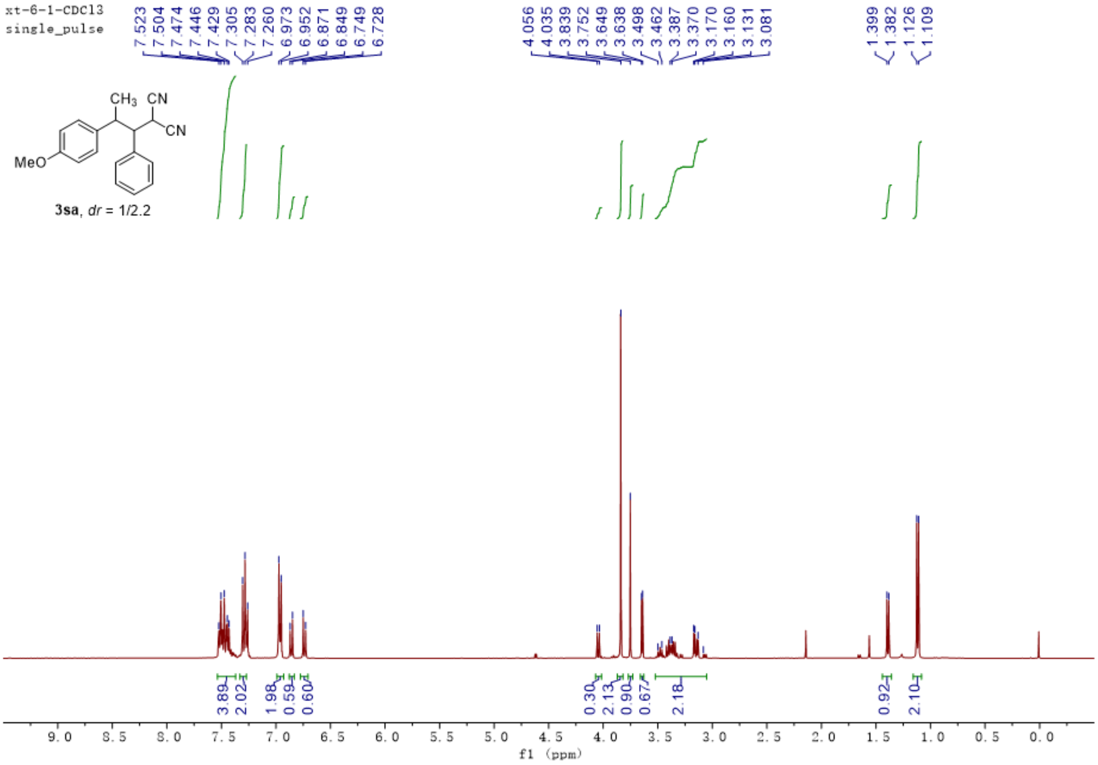

^{1}H NMR (400 MHz, CDCl$_3$) of **3sa**

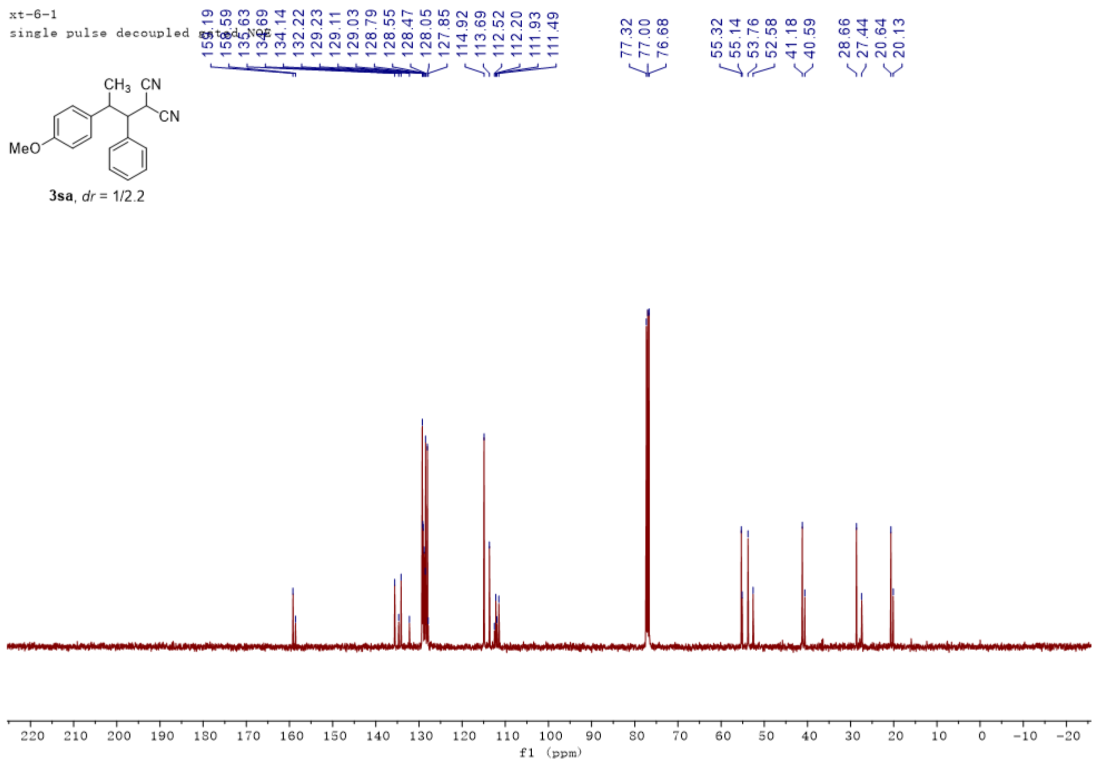

^{13}C NMR (101 MHz, $CDCl_3$) of **3sa**

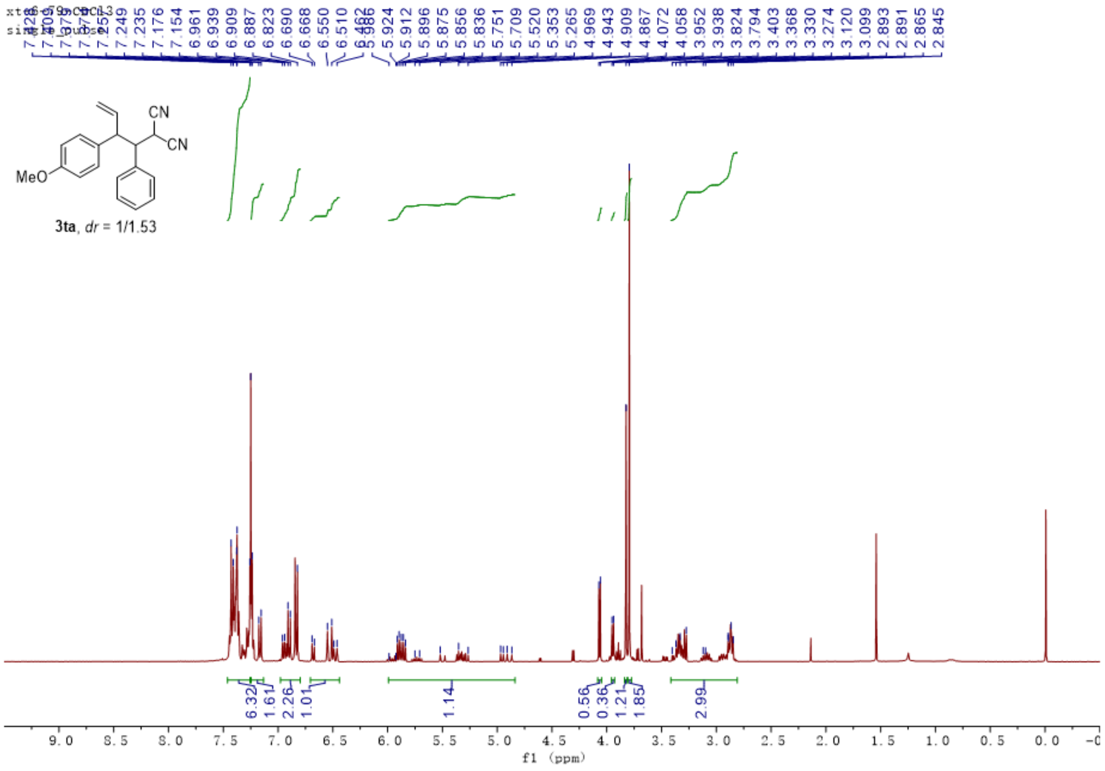

^{1}H NMR (400 MHz, $CDCl_3$) of **3ta**

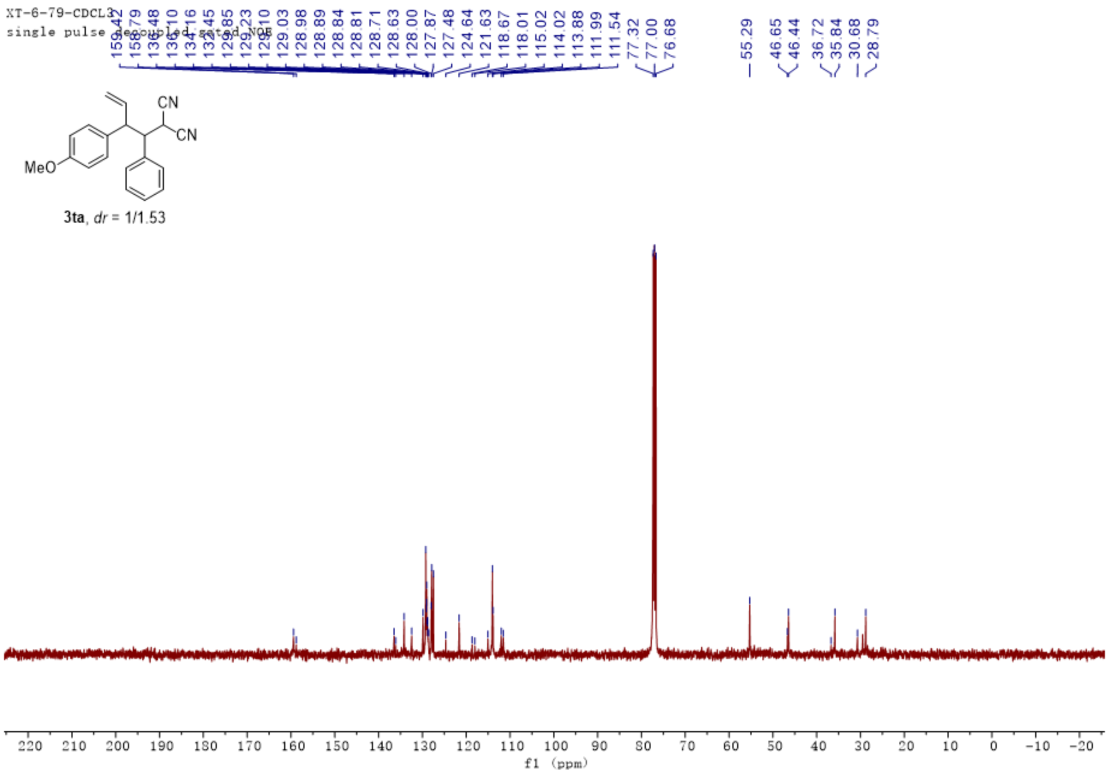

^{13}C NMR (101 MHz, $CDCl_3$) of **3ta**

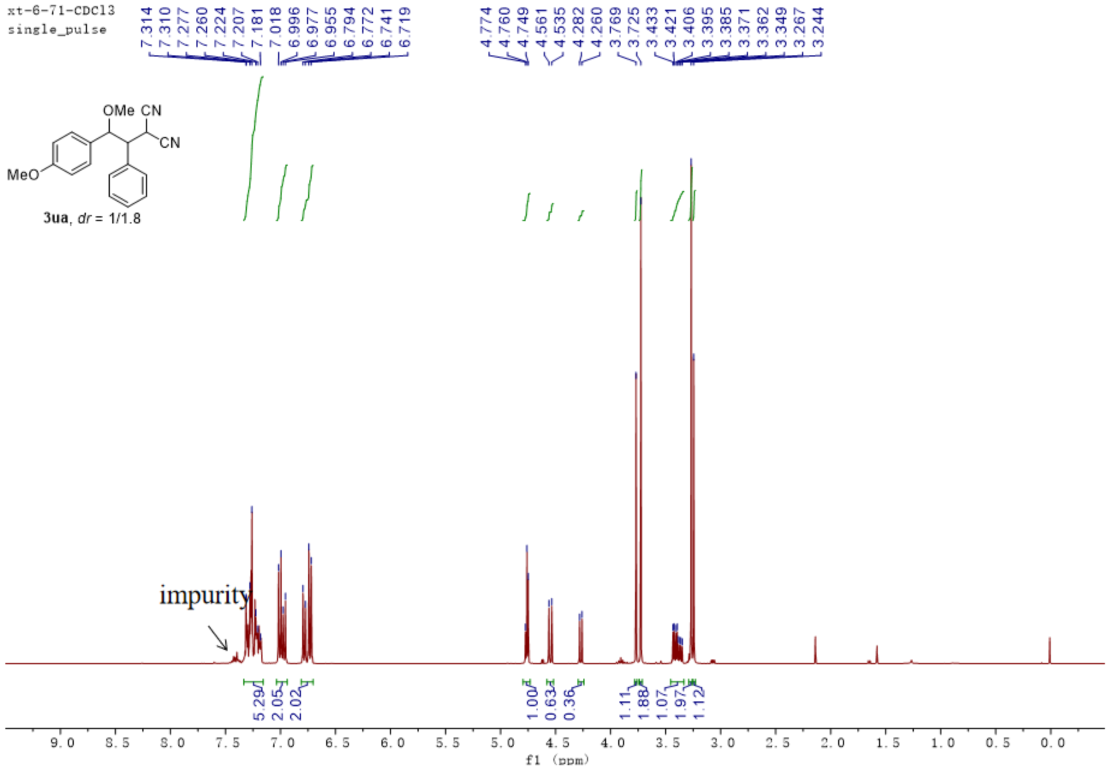

^{1}H NMR (400 MHz, $CDCl_3$) of **3ua**

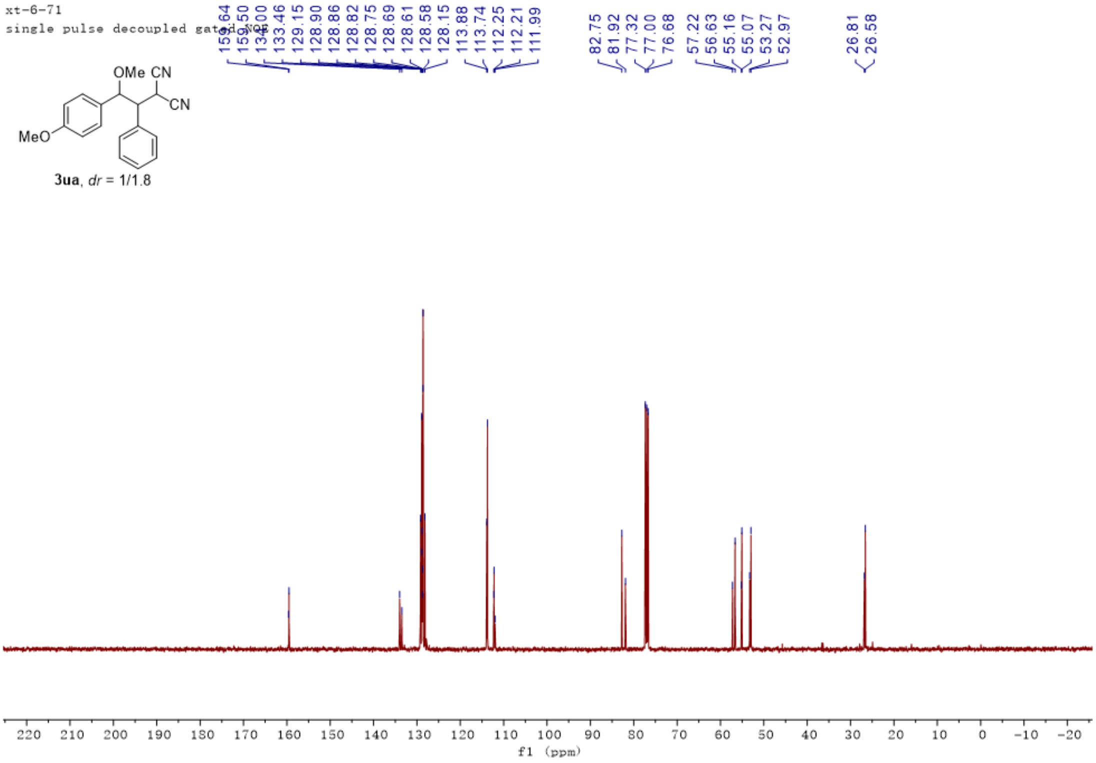

^{13}C NMR (101 MHz, CDCl$_3$) of **3ua**

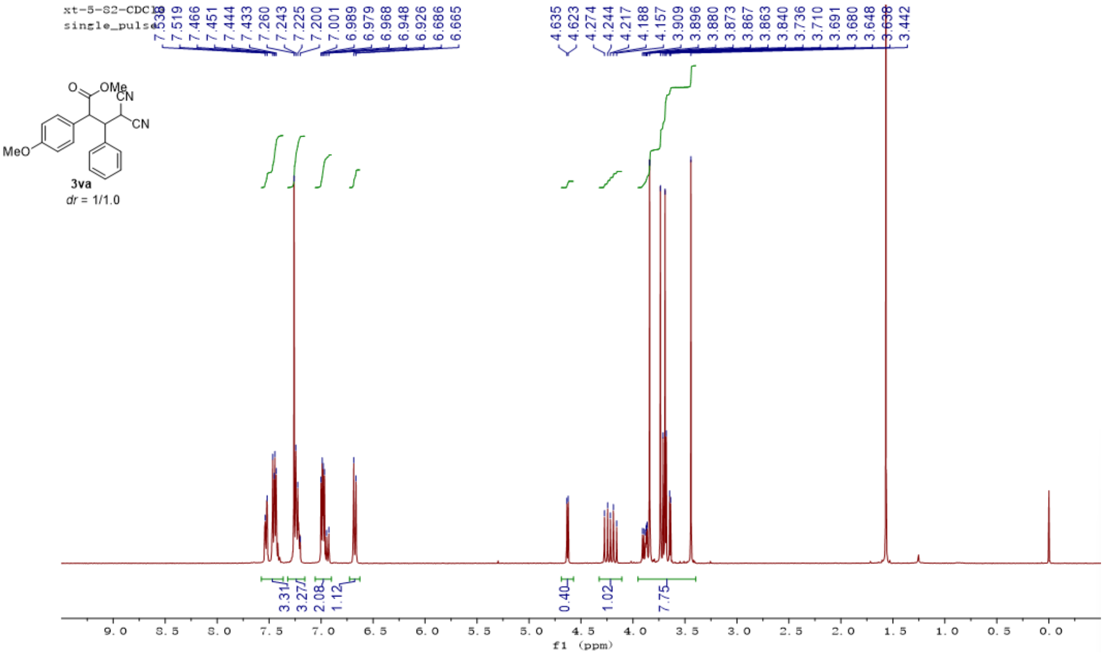

^{1}H NMR (400 MHz, CDCl$_3$) of **3va**

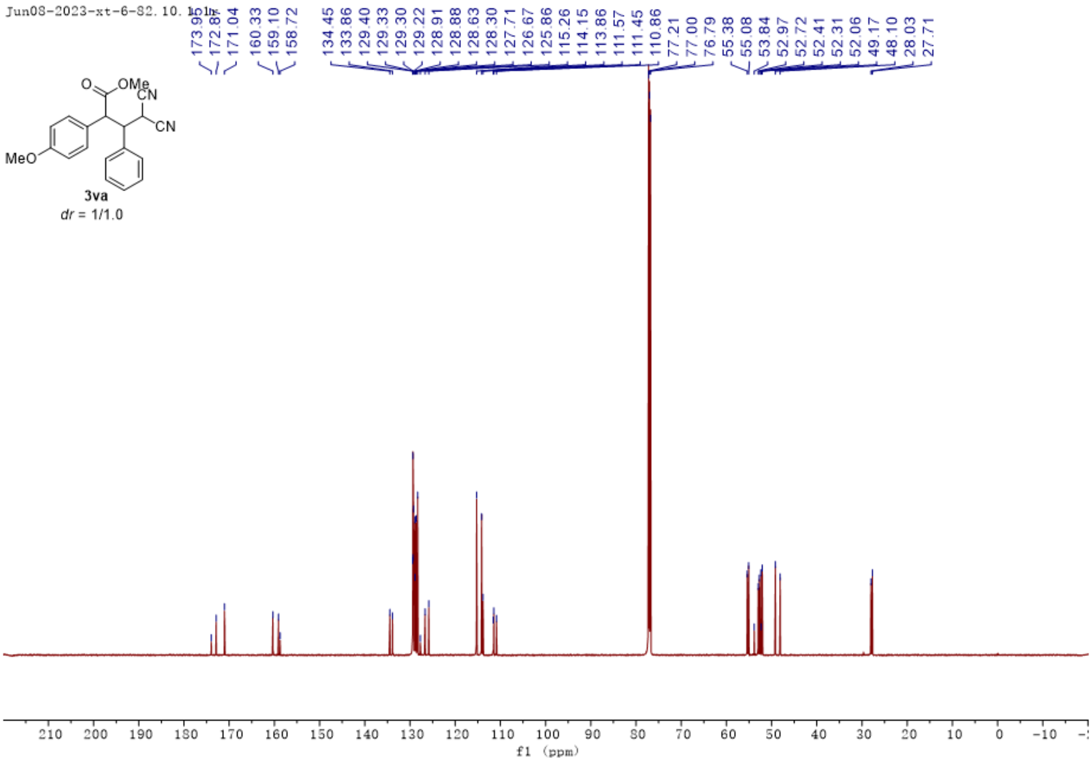

^{13}C NMR (101 MHz, $CDCl_3$) of **3va**

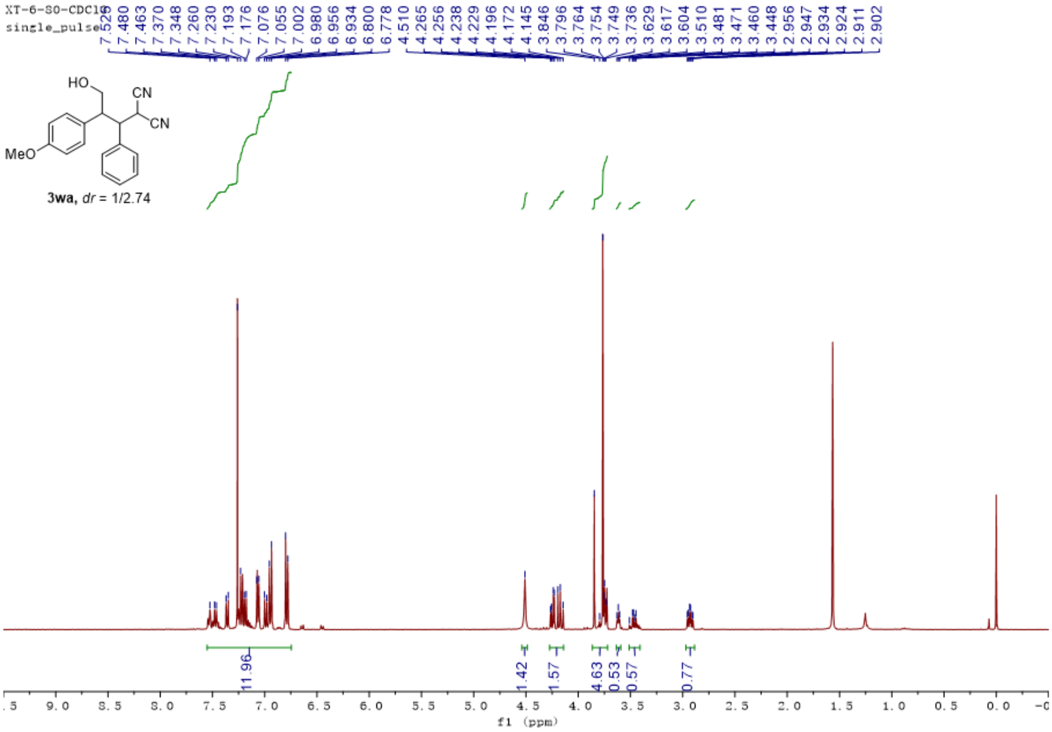

^{1}H NMR (400 MHz, $CDCl_3$) of **3wa**

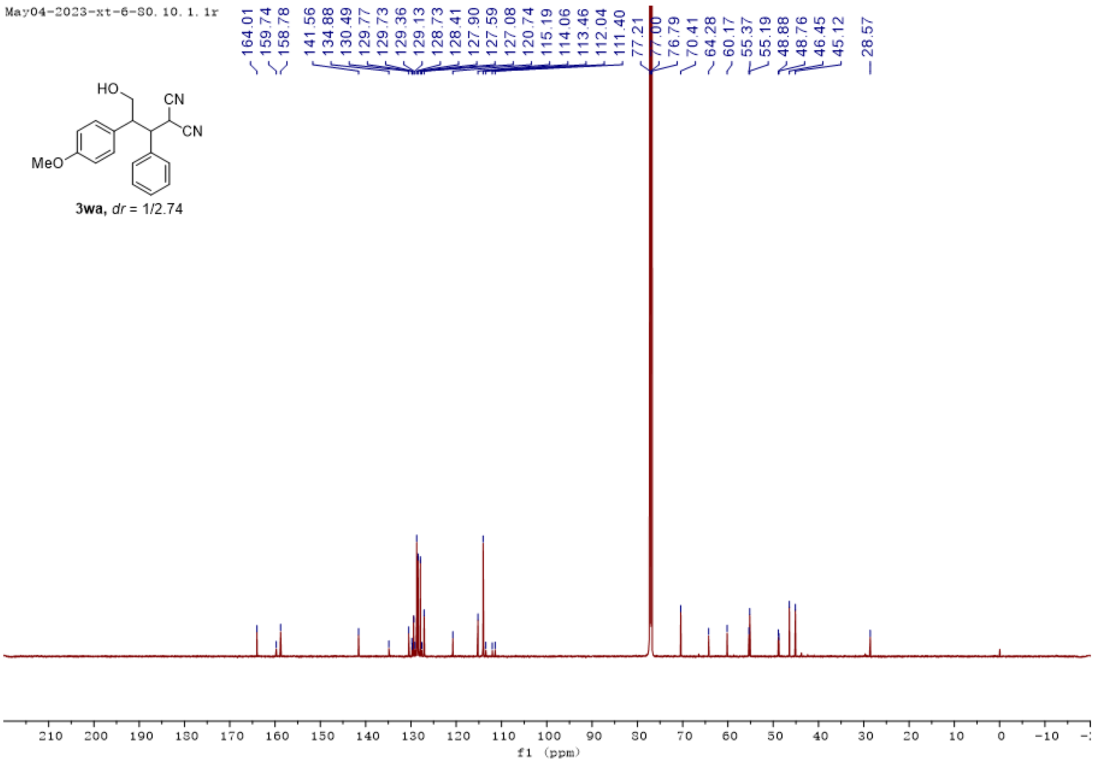

^{13}C NMR (151 MHz, $CDCl_3$) of **3wa**

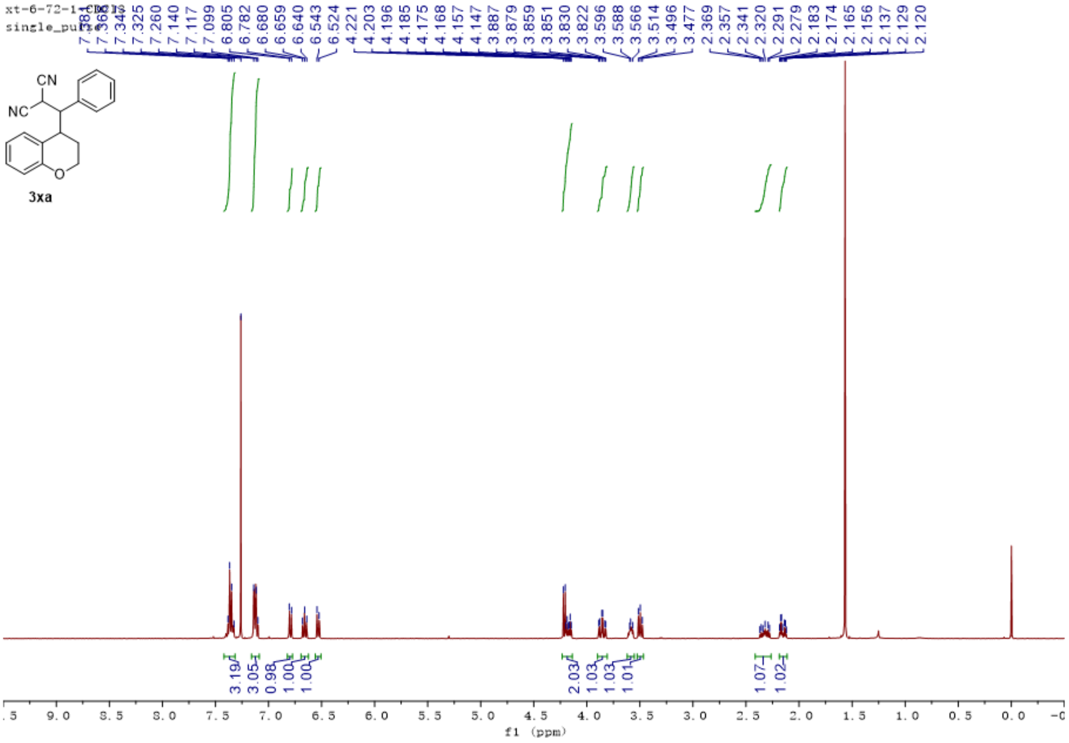

^{1}H NMR (400 MHz, $CDCl_3$) of **3xa**

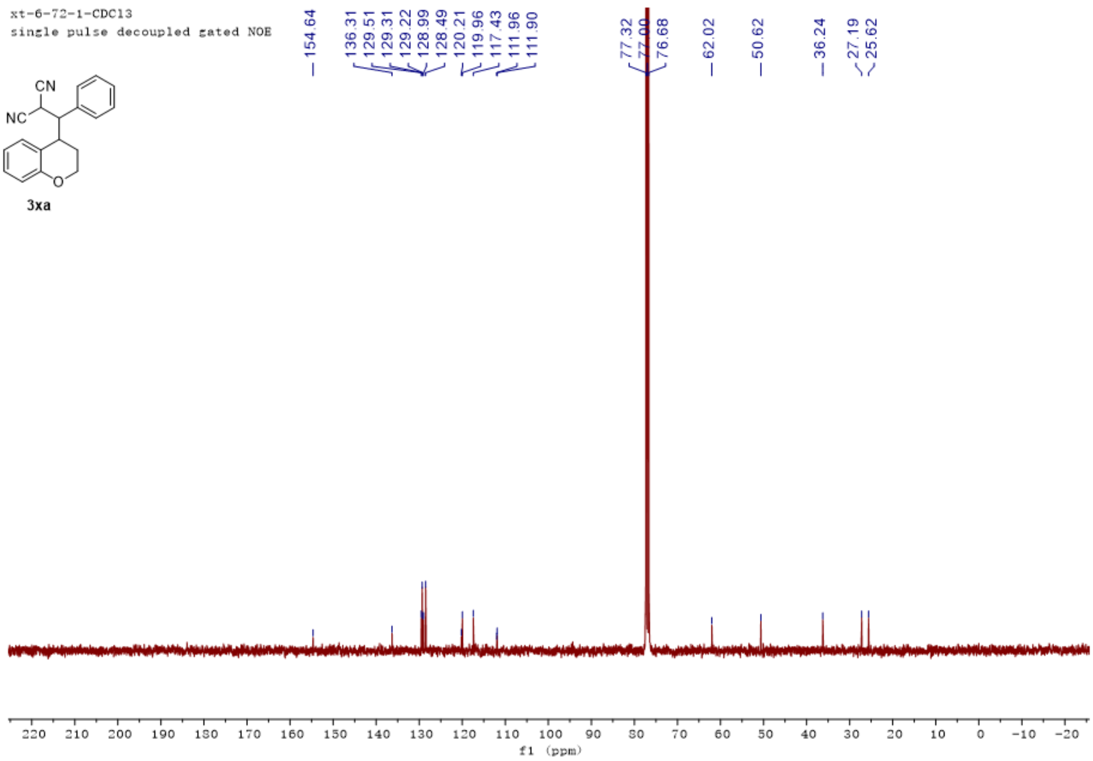

^{13}C NMR (101 MHz, $CDCl_3$) of **3xa**

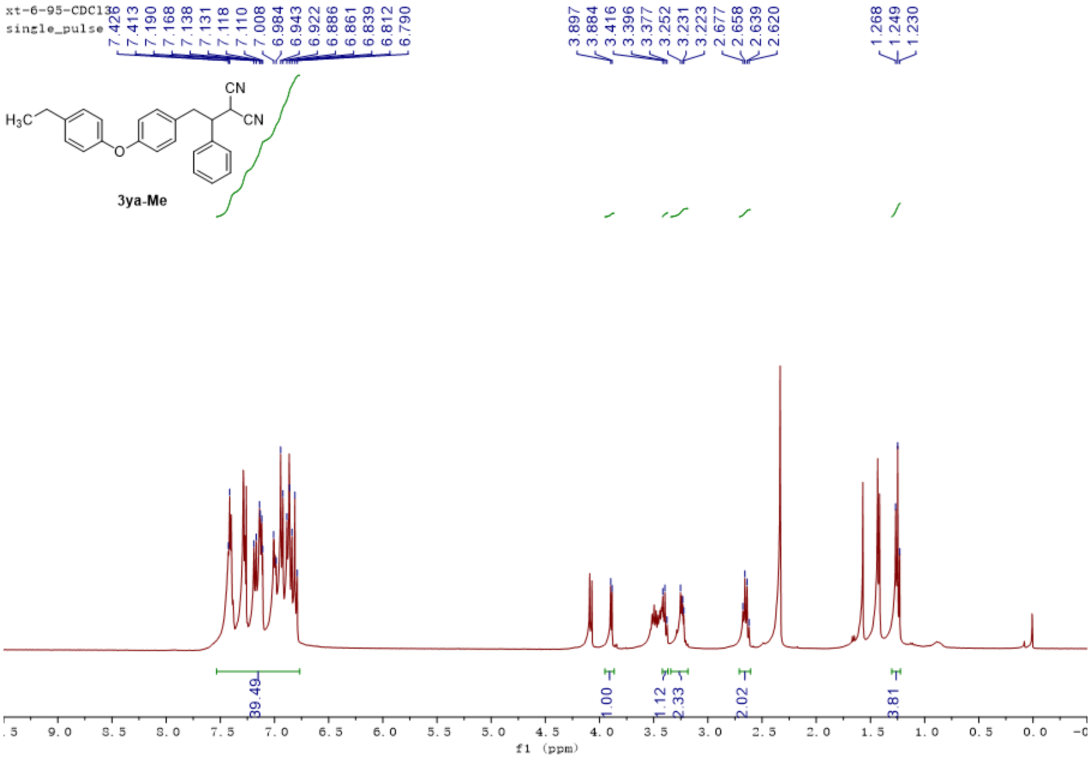

^{1}H NMR (400 MHz, $CDCl_3$) of **3ya-Me**

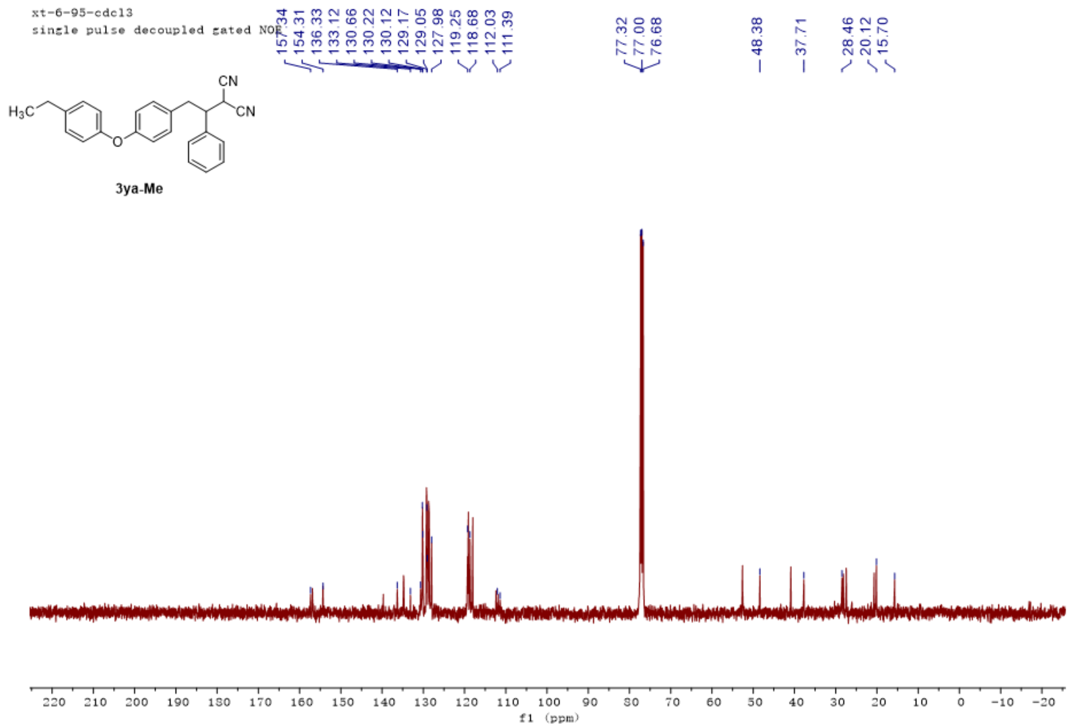

^{13}C NMR (101 MHz, CDCl$_3$) of **3ya-Me**

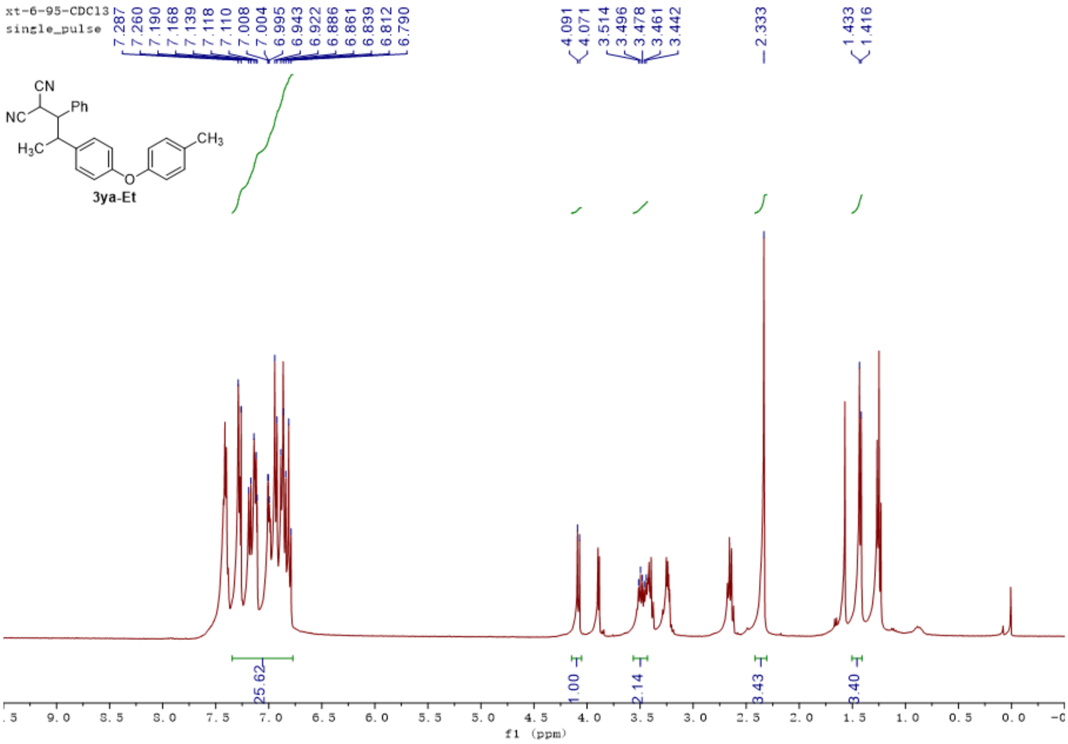

^{1}H NMR (400 MHz, CDCl$_3$) of **3ya-Et**

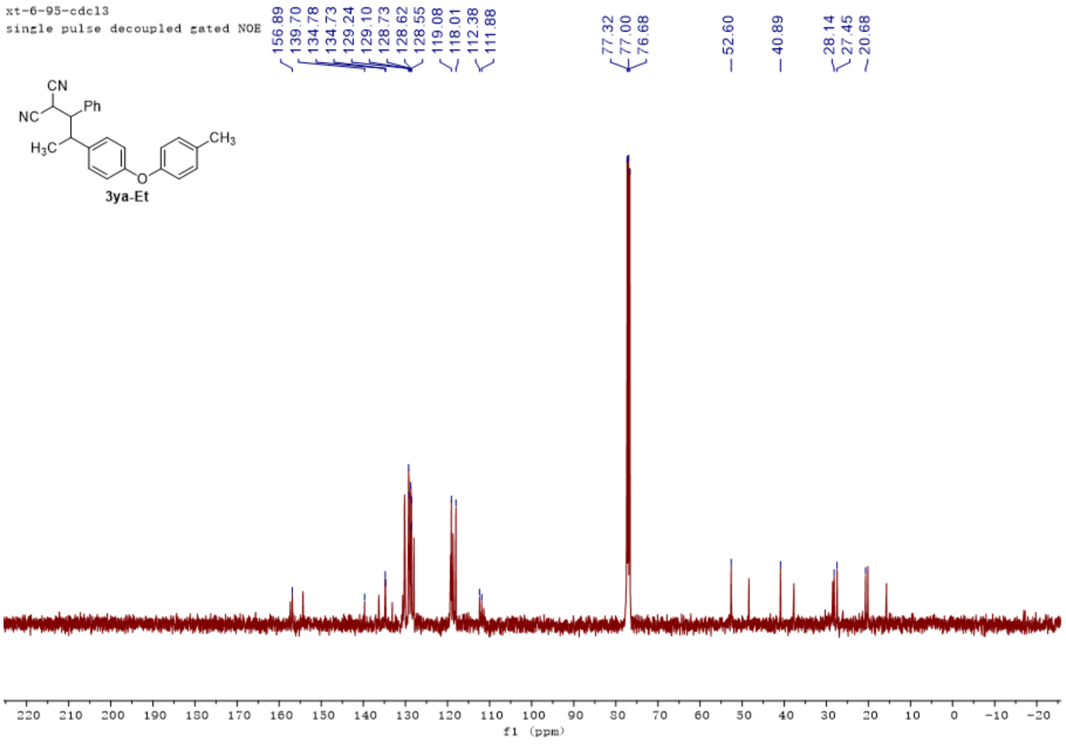

^{13}C NMR (101 MHz, CDCl$_3$) of **3ya-Et**

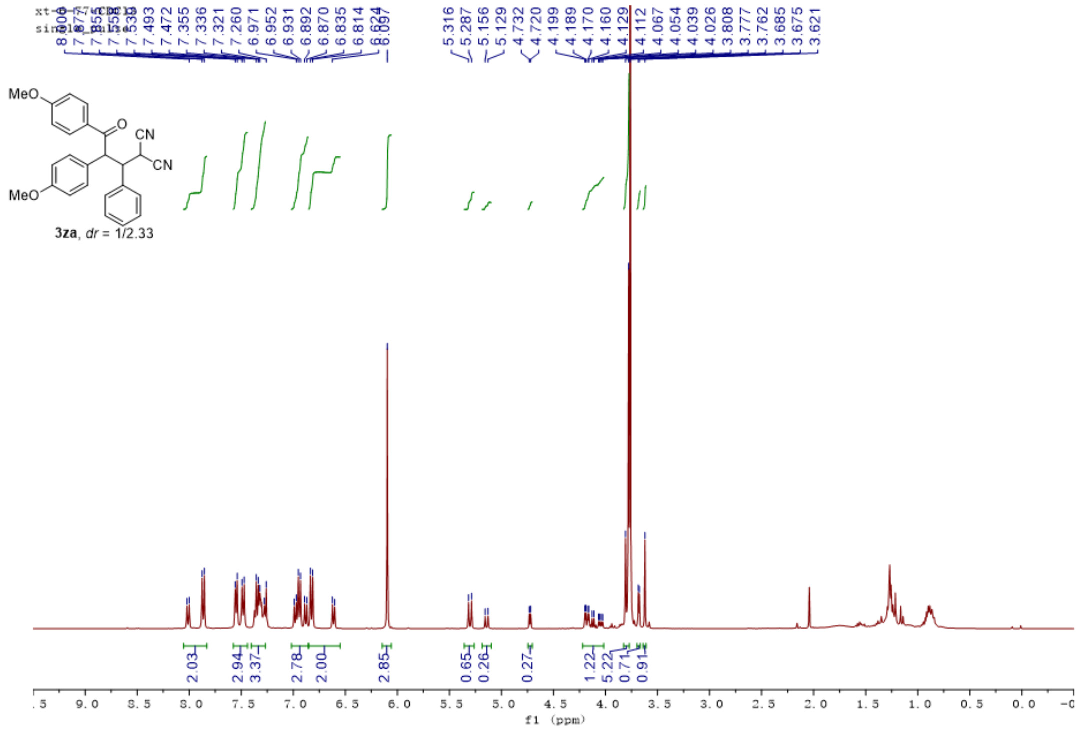

^{1}H NMR (400 MHz, CDCl$_3$) of **3za** with 1,3,5-trimethoxybenzene as internal standard

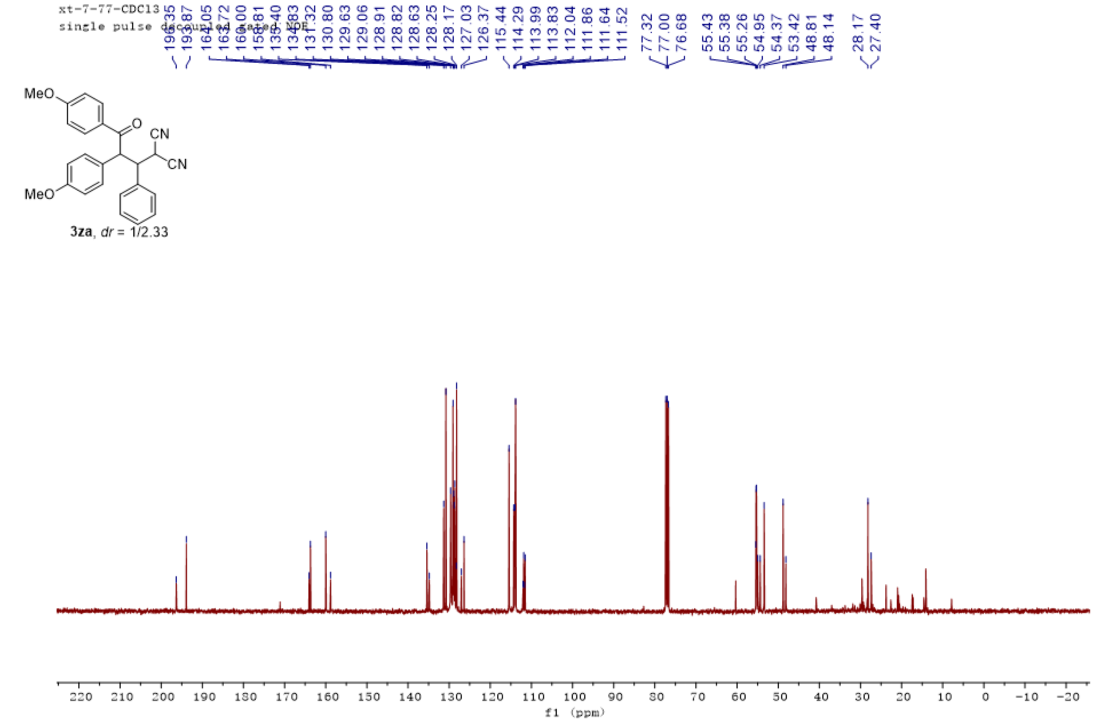

^{13}C NMR (101 MHz, $CDCl_3$) of **3za**

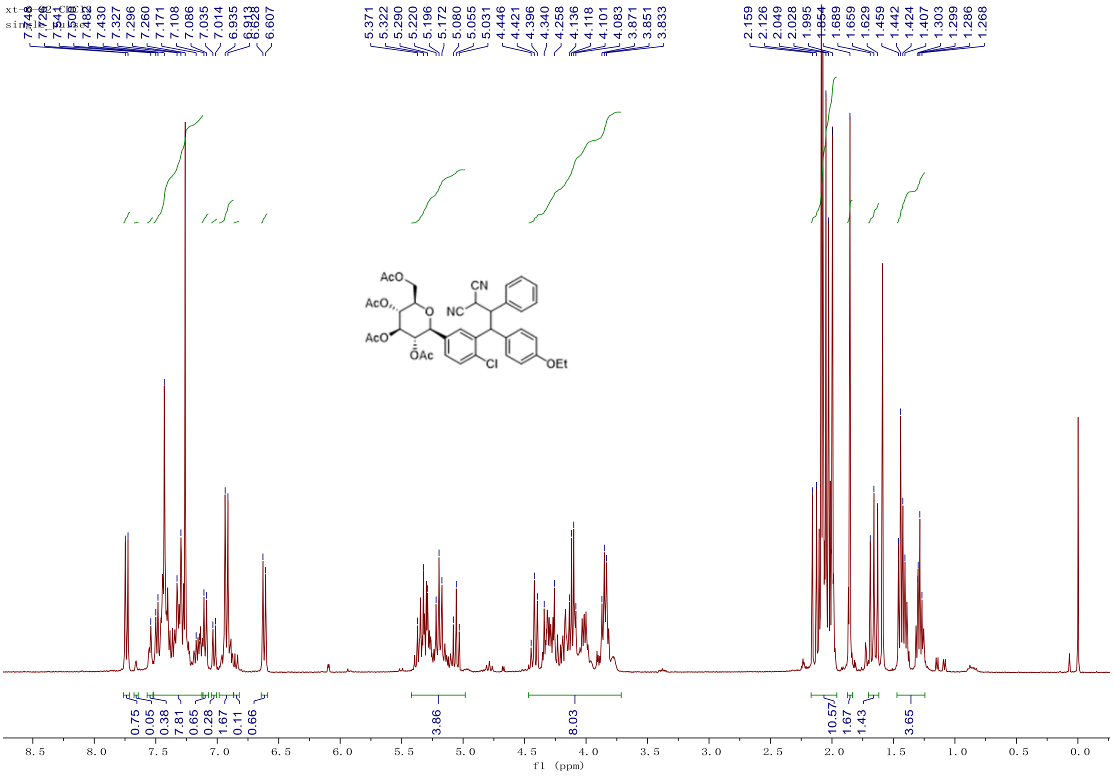

^{1}H NMR (400 MHz, $CDCl_3$) of **3aaa**

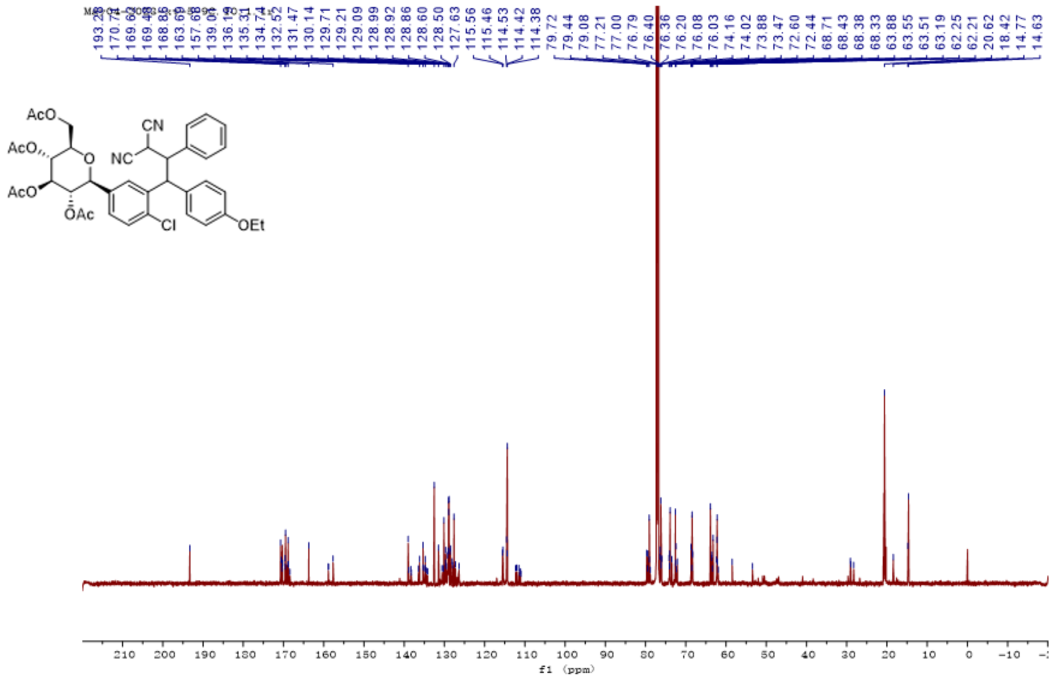

^{13}C NMR (151 MHz, $CDCl_3$) of **3aaa**

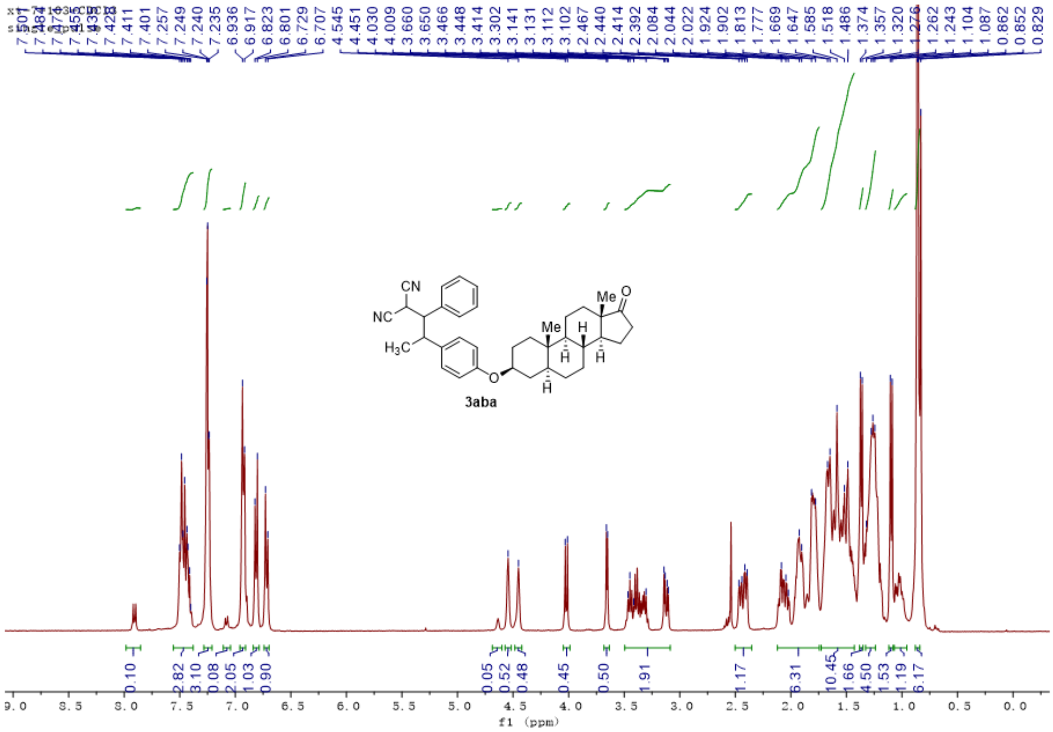

^{1}H NMR (400 MHz, $CDCl_3$) of **3aba**

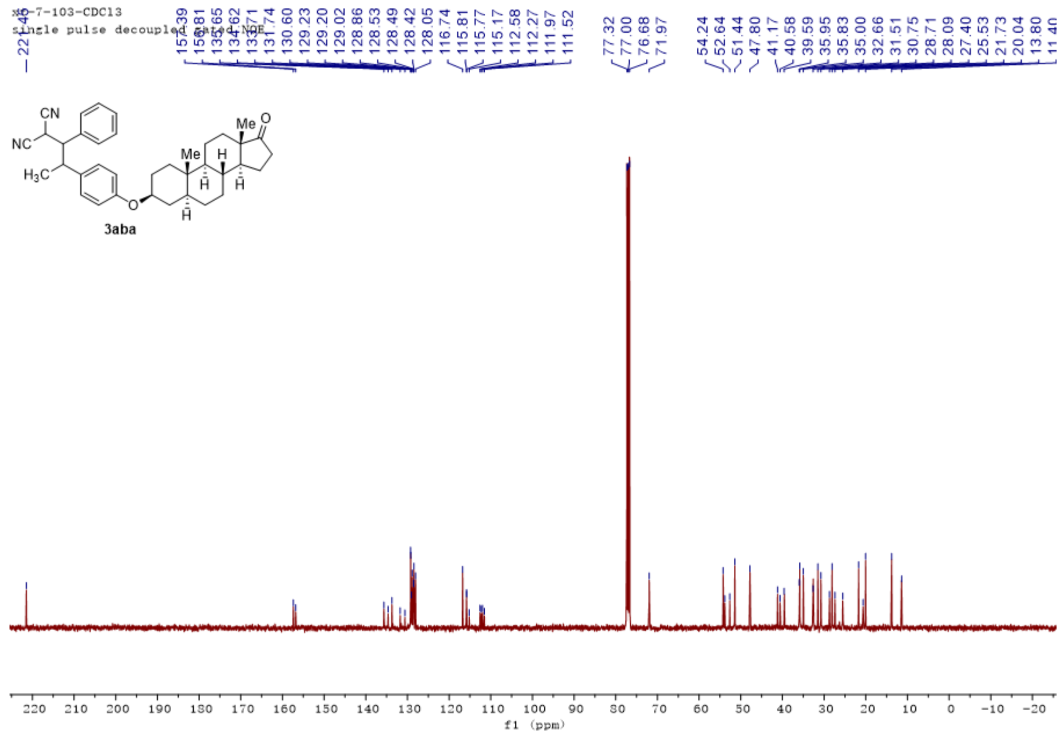

^{13}C NMR (101 MHz, $CDCl_3$) of **3aba**

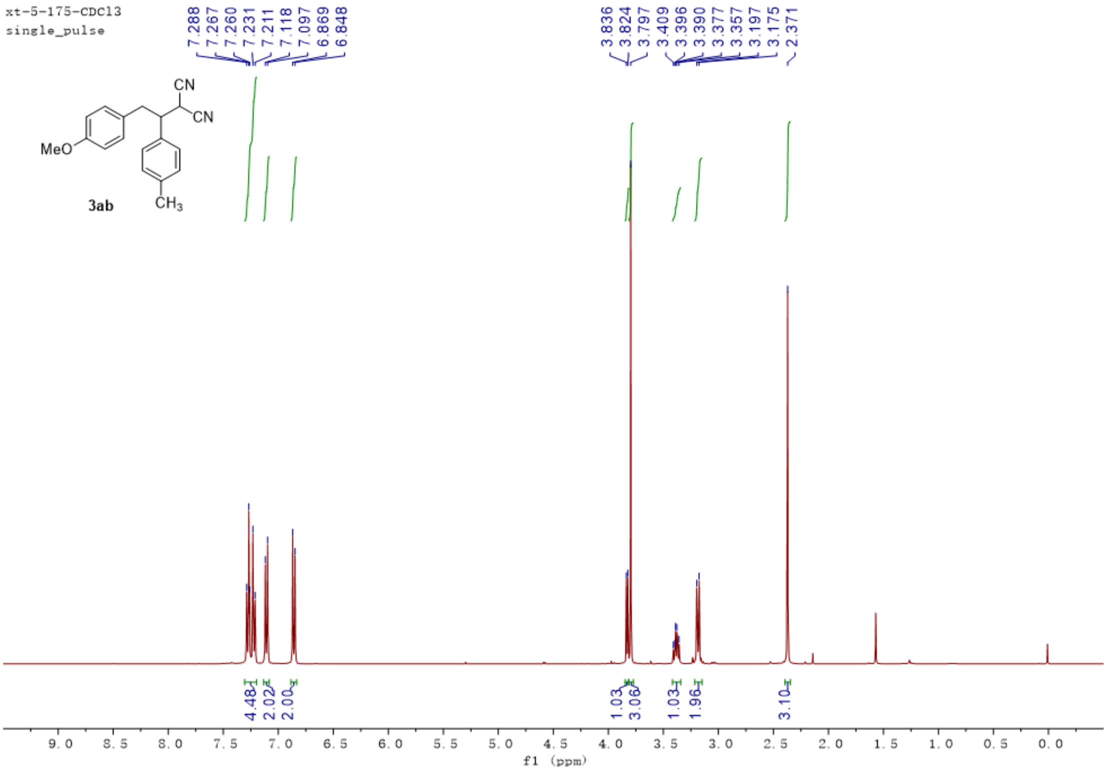

^{1}H NMR (400 MHz, $CDCl_3$) of **3ab**

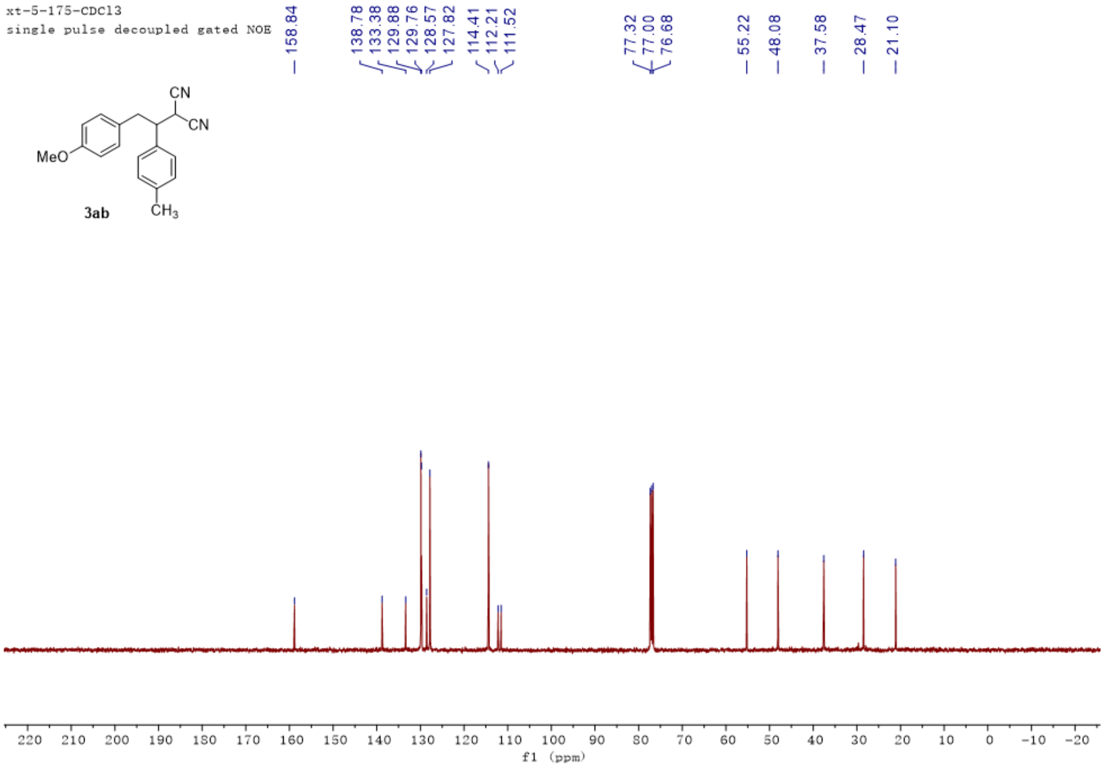

^{13}C NMR (101 MHz, $CDCl_3$) of **3ab**

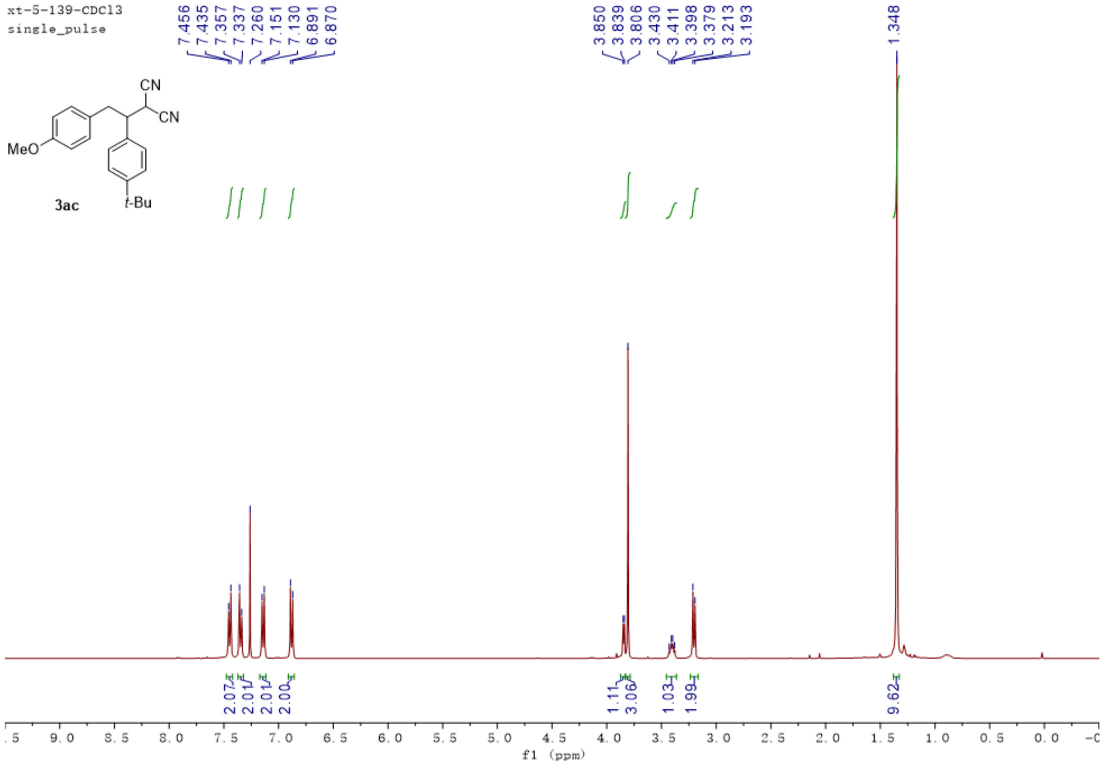

^{1}H NMR (400 MHz, $CDCl_3$) of **3ac**

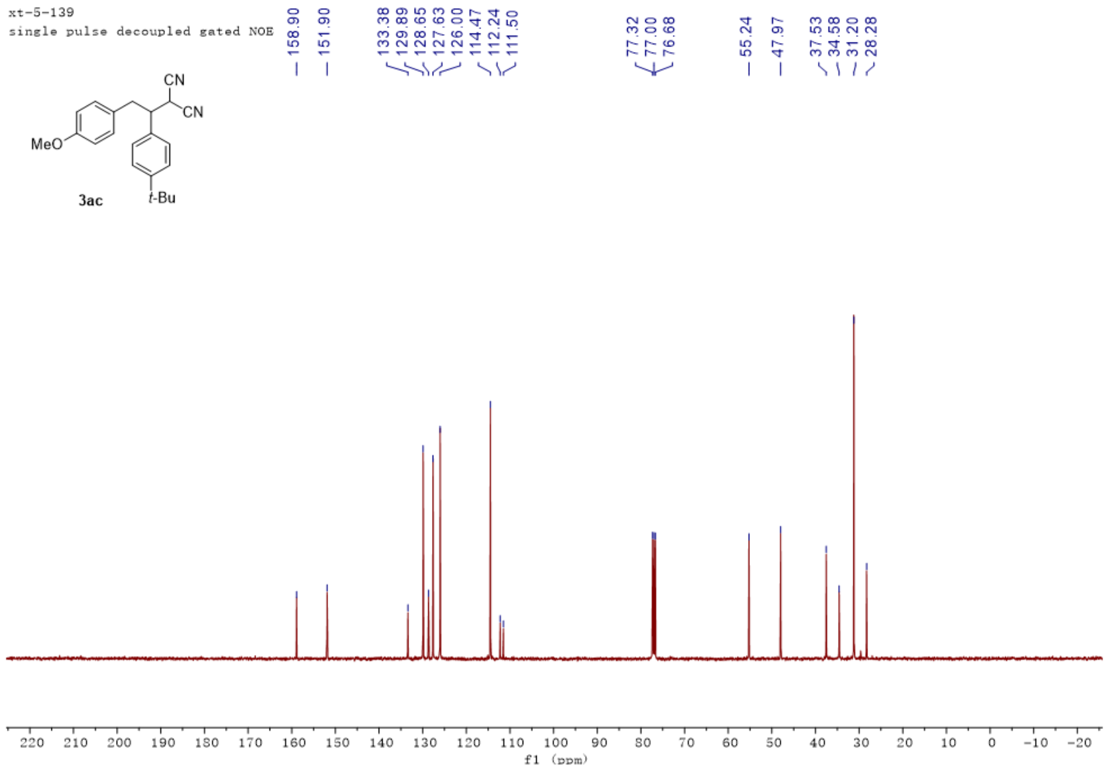

^{13}C NMR (101 MHz, $CDCl_3$) of **3ac**

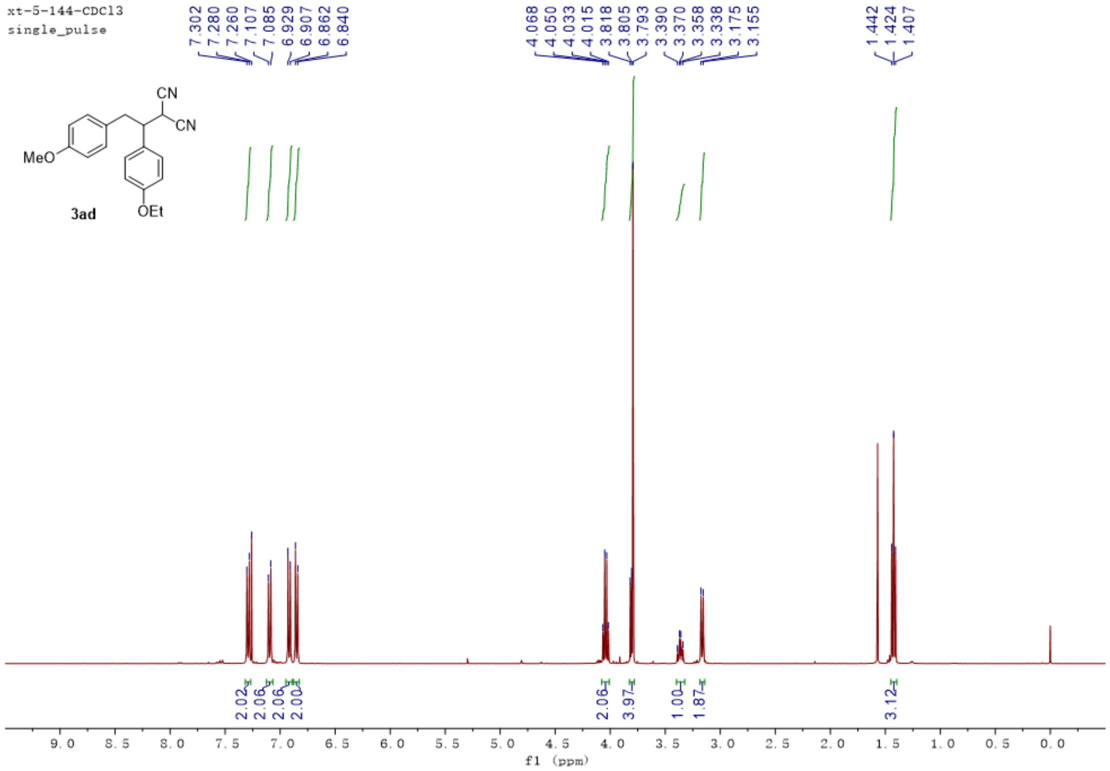

^{1}H NMR (400 MHz, $CDCl_3$) of **3ad**

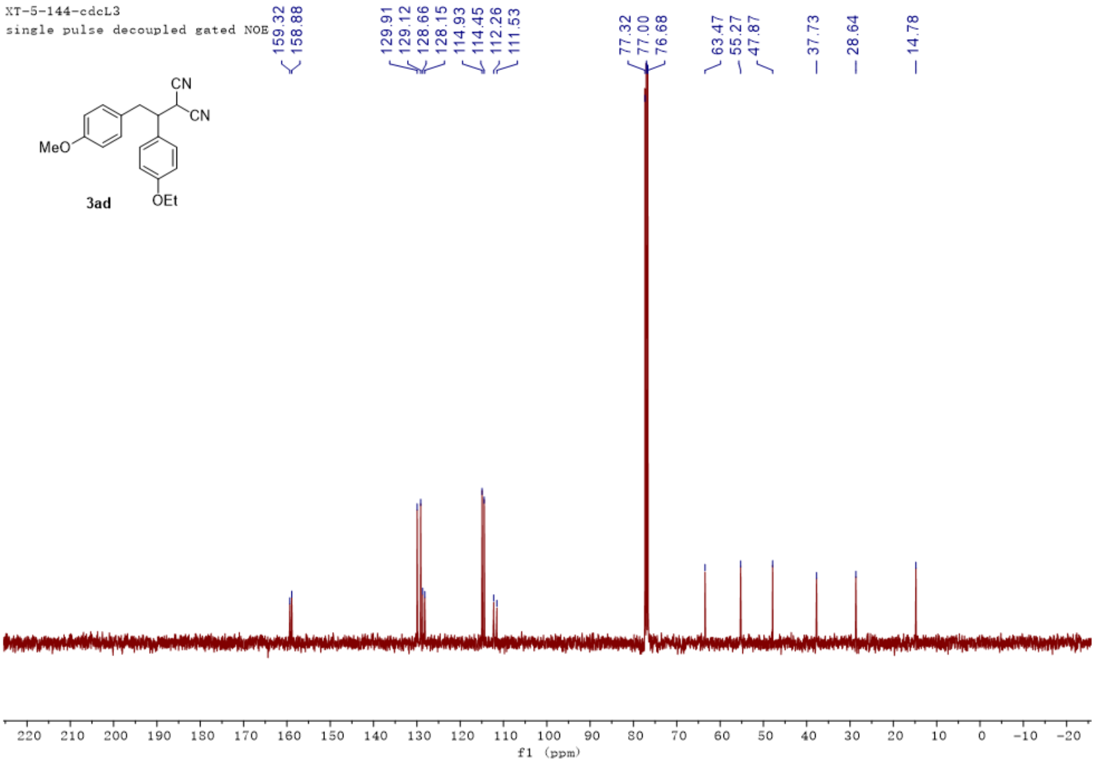

^{13}C NMR (101 MHz, $CDCl_3$) of **3ad**

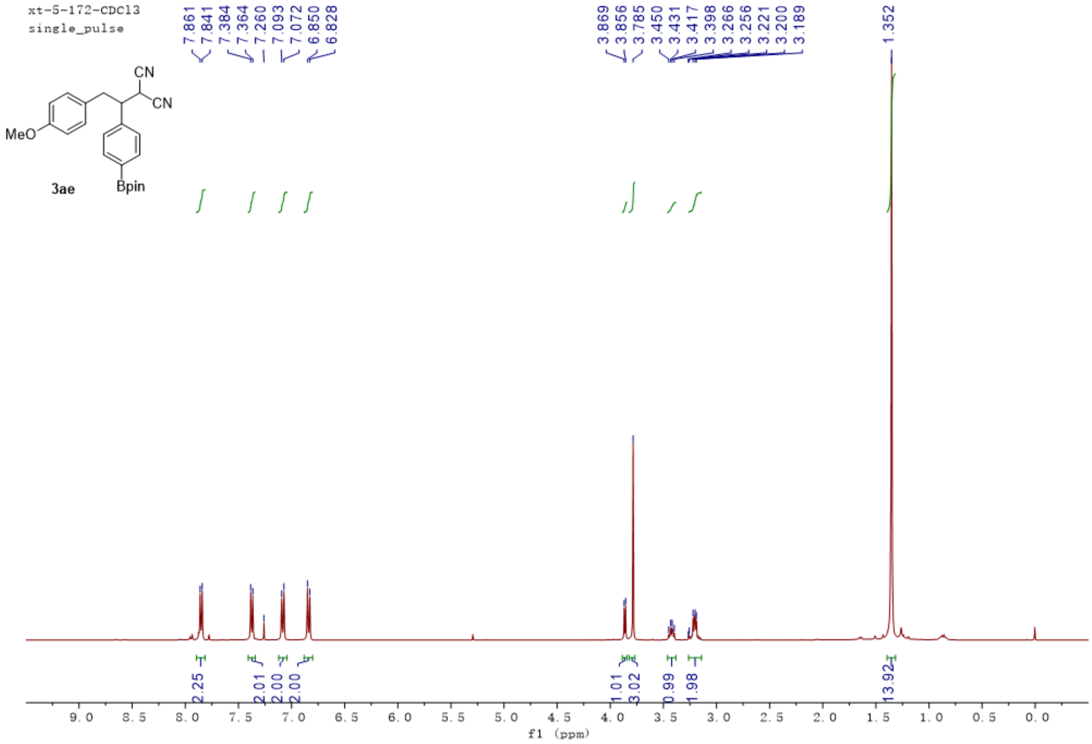

^{1}H NMR (400 MHz, $CDCl_3$) of **3ae**

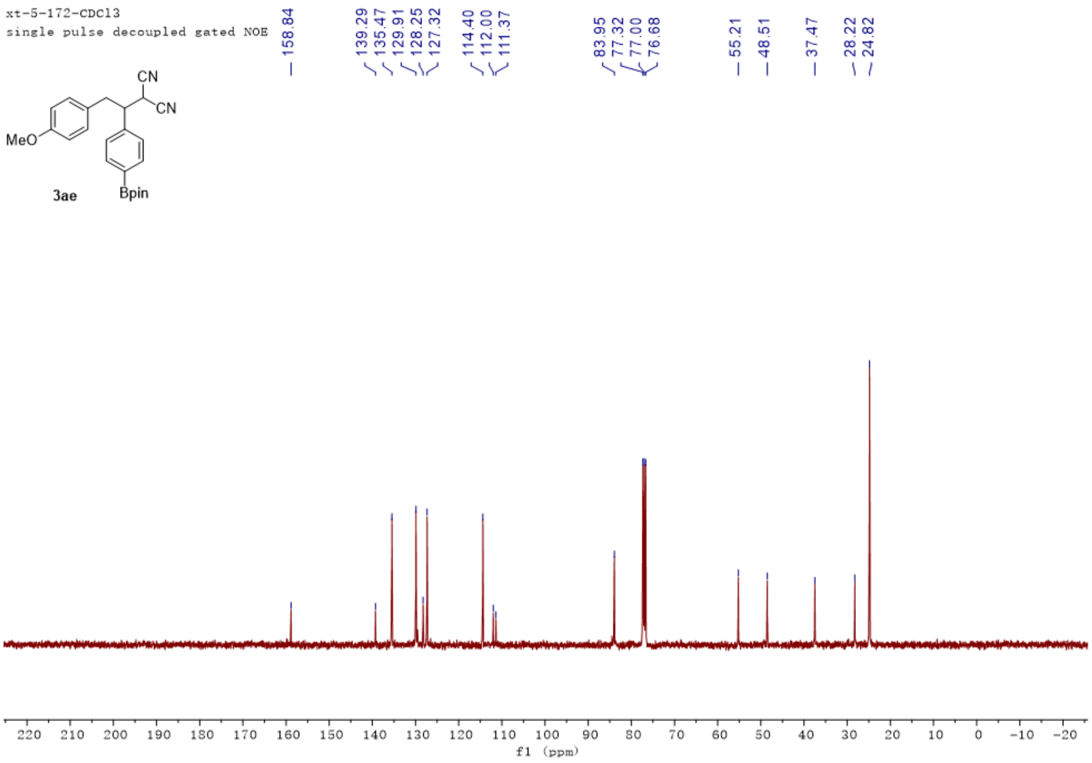

^{13}C NMR (101 MHz, CDCl$_3$) of **3ae**

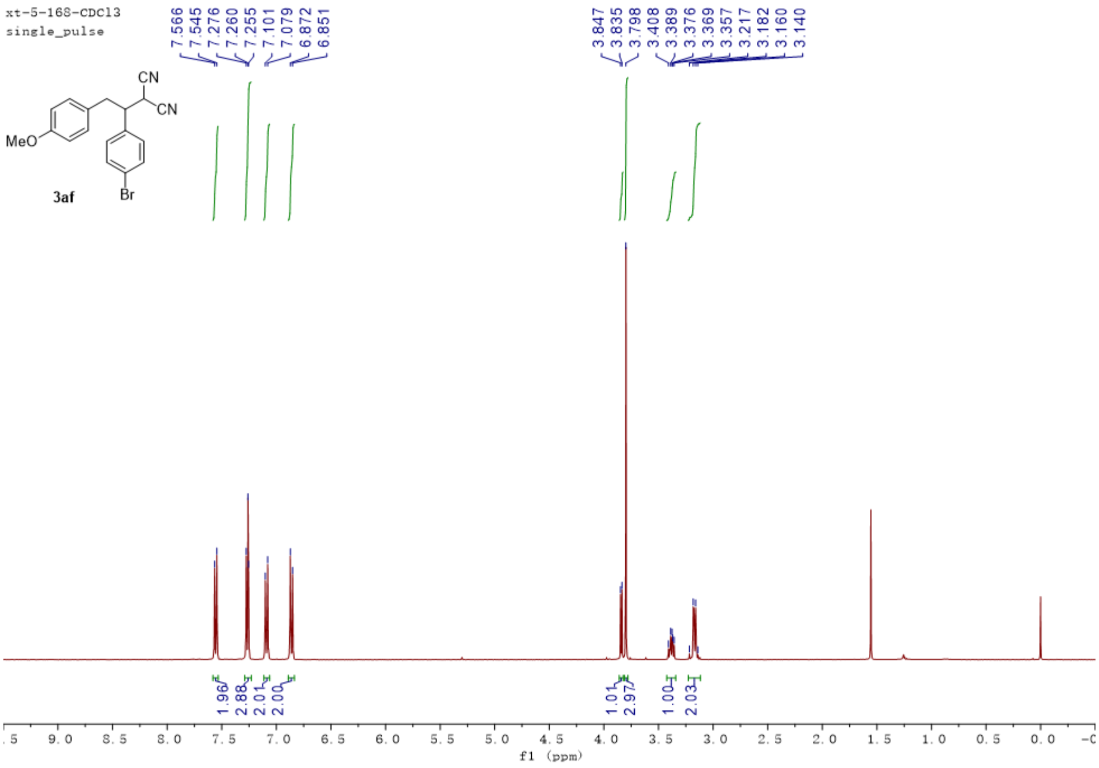

^{1}H NMR (400 MHz, CDCl$_3$) of **3af**

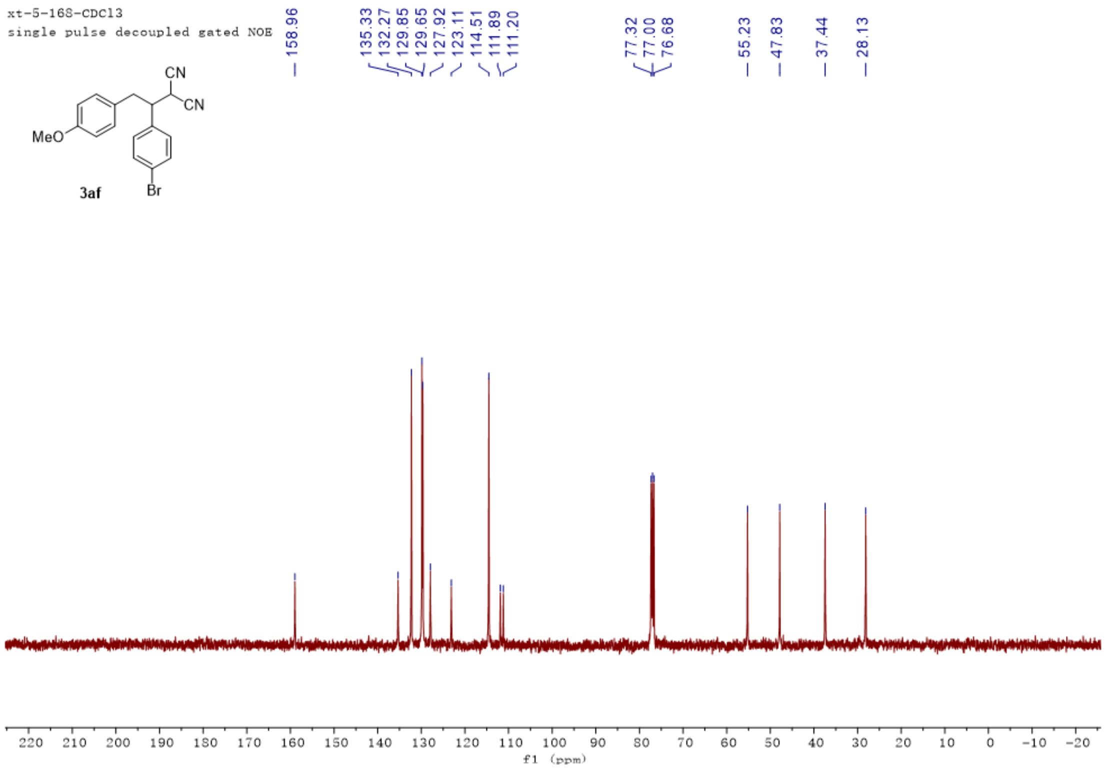

^{13}C NMR (101 MHz, $CDCl_3$) of **3af**

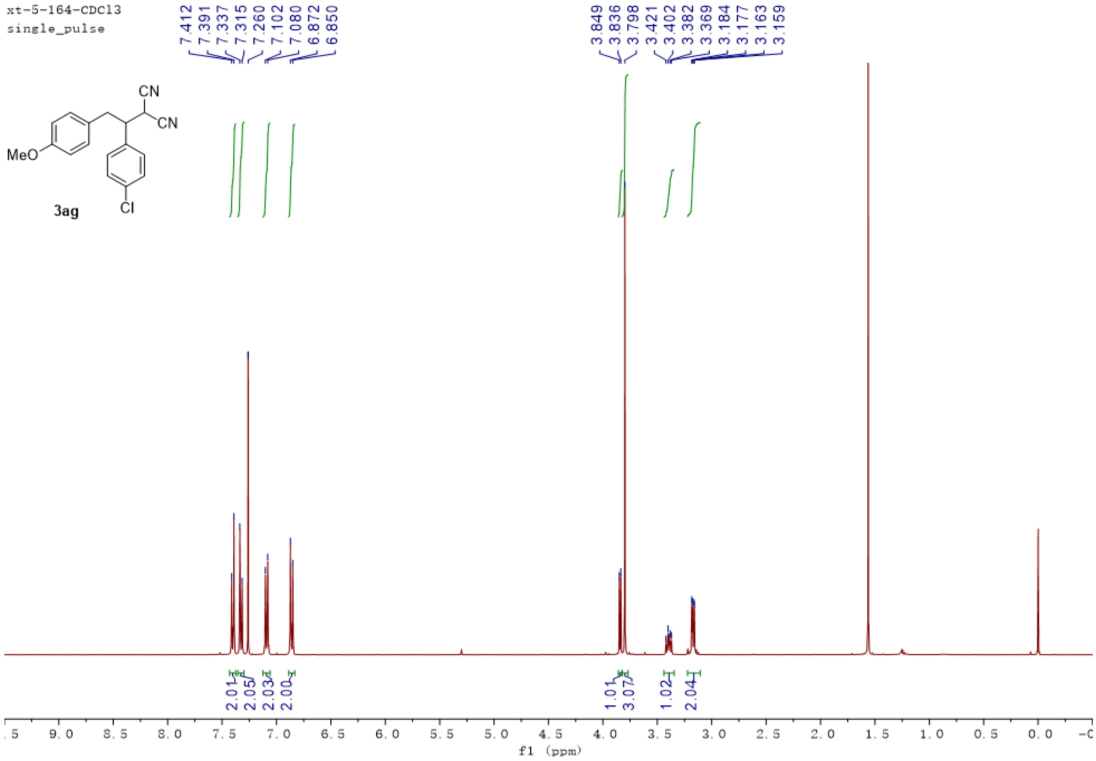

^{1}H NMR (400 MHz, $CDCl_3$) of **3ag**

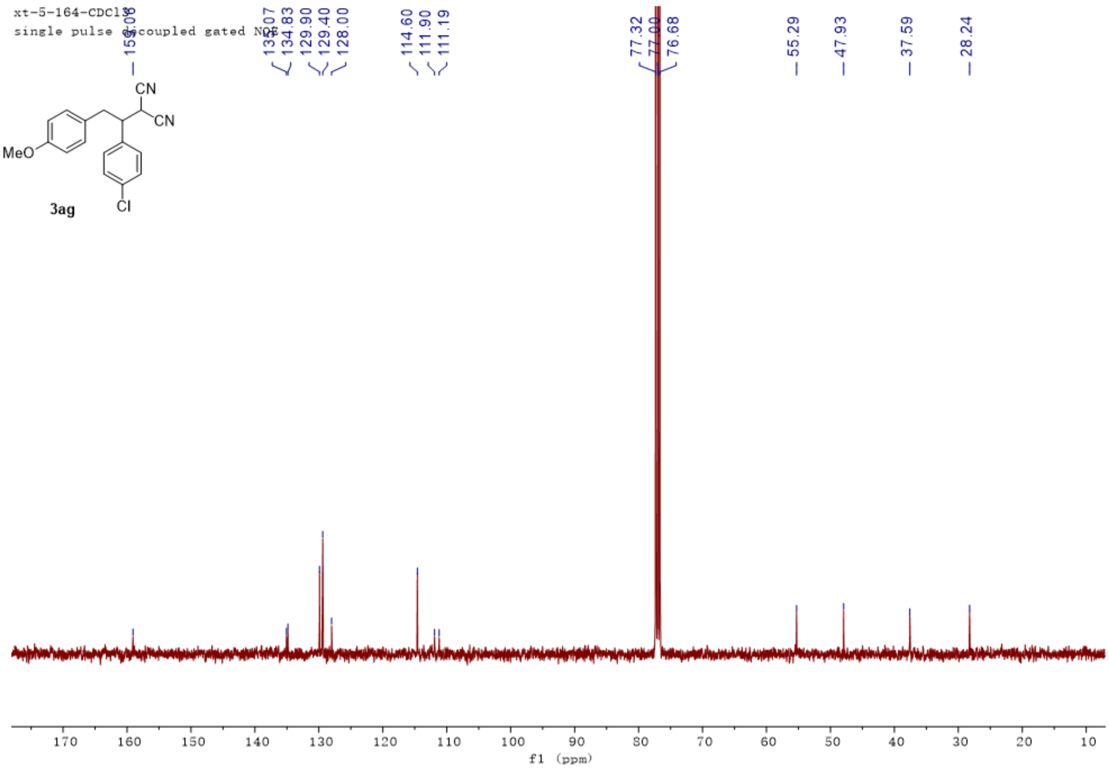

^{13}C NMR (101 MHz, $CDCl_3$) of **3ag**

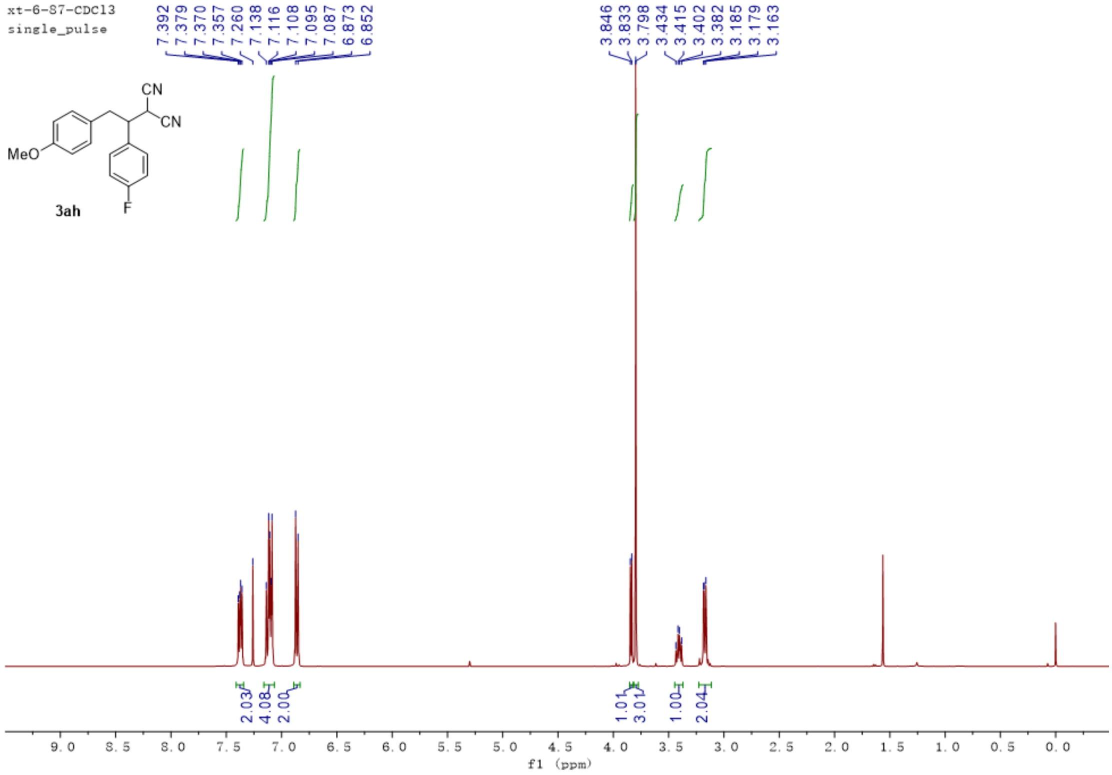

^{1}H NMR (400 MHz, $CDCl_3$) of **3ah**

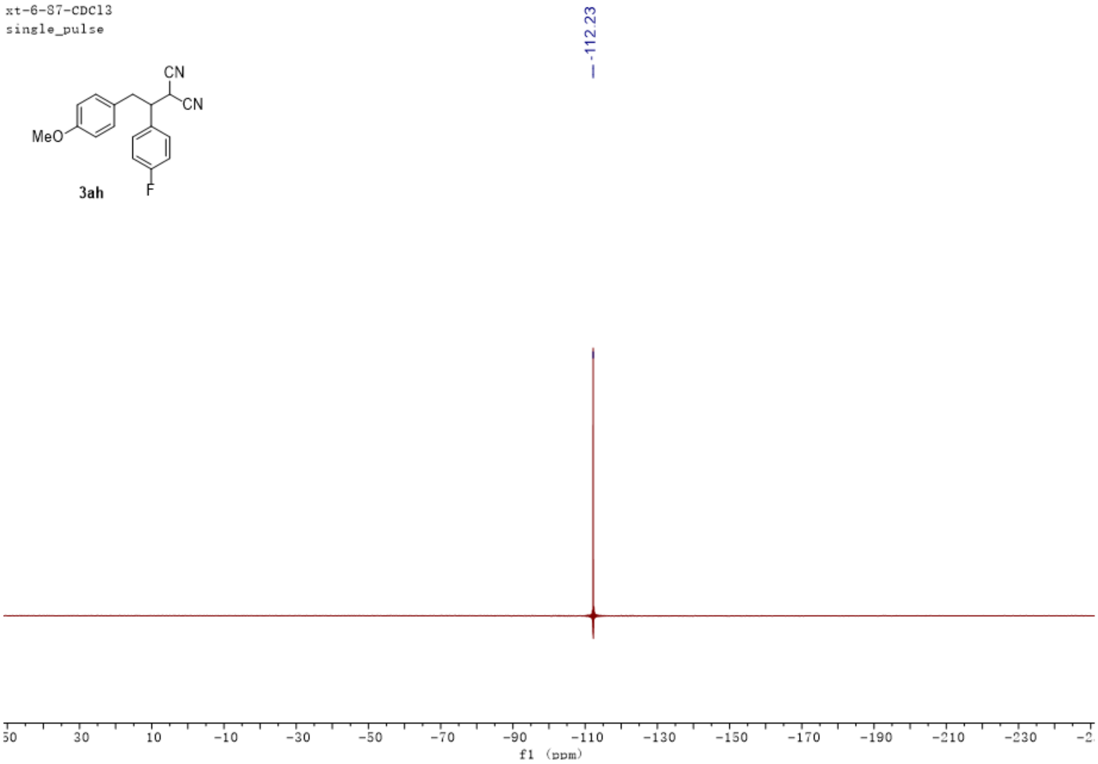

^{19}F NMR (376 MHz, $CDCl_3$) of **3ah**

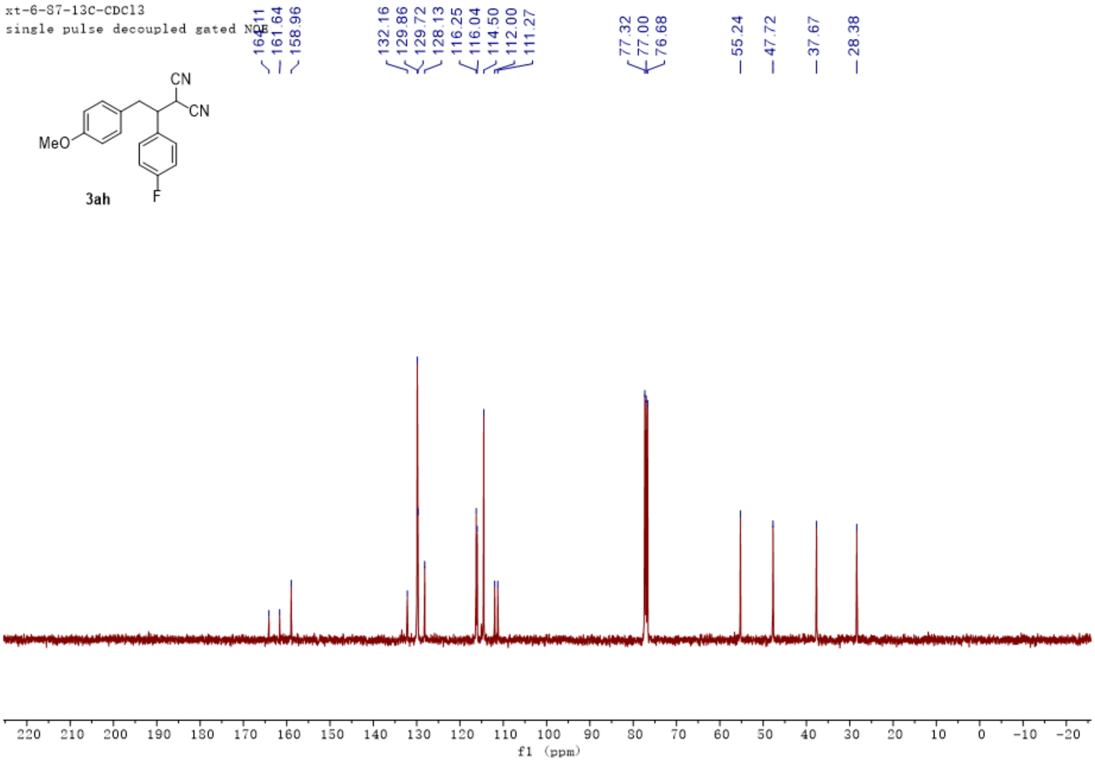

^{13}C NMR (101 MHz, $CDCl_3$) of **3ah**

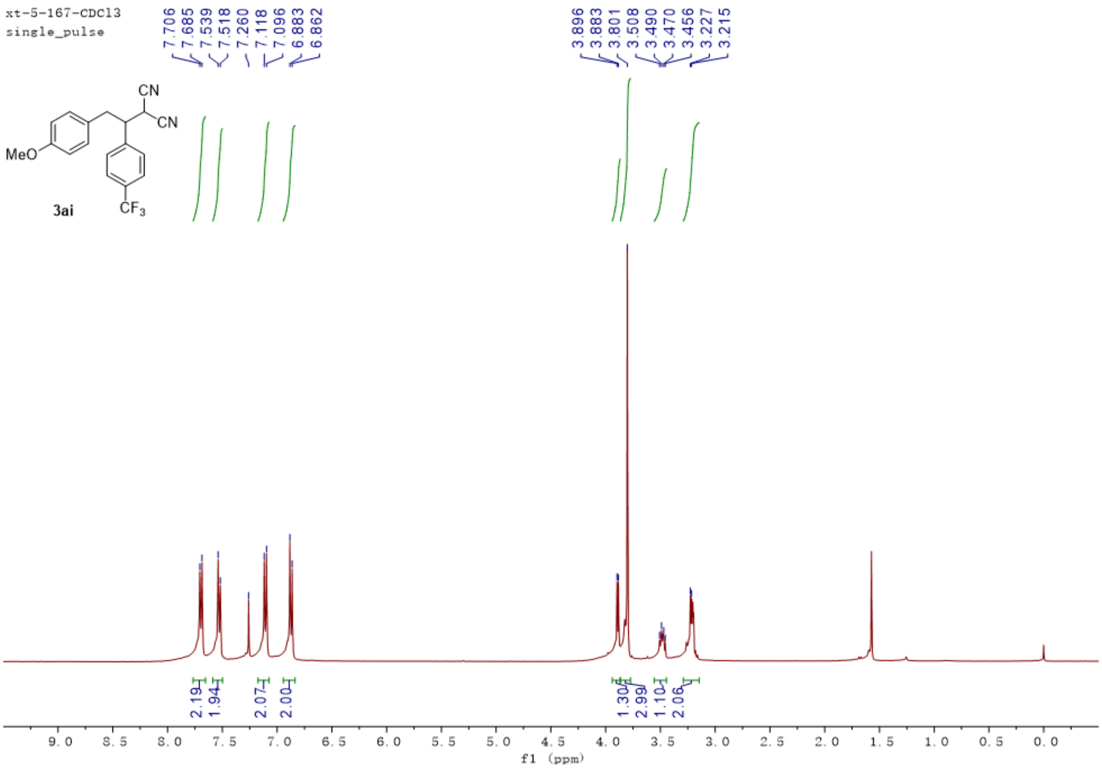

^{1}H NMR (400 MHz, $CDCl_3$) of **3ai**

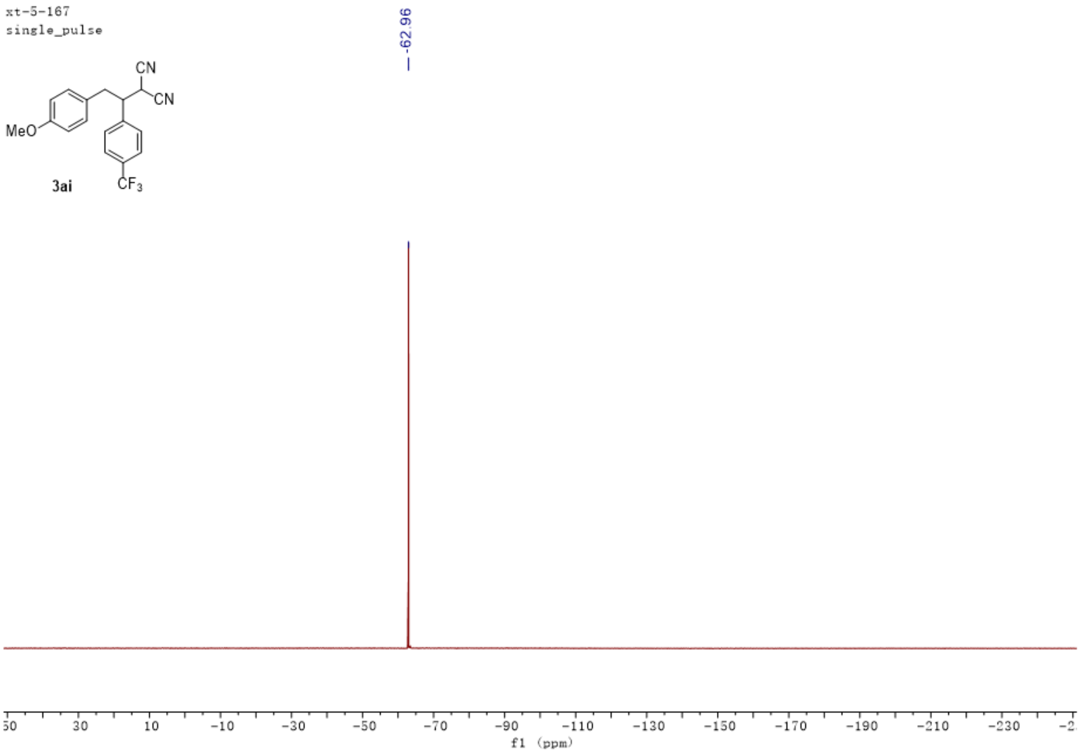

^{19}F NMR (376 MHz, $CDCl_3$) of **3ai**

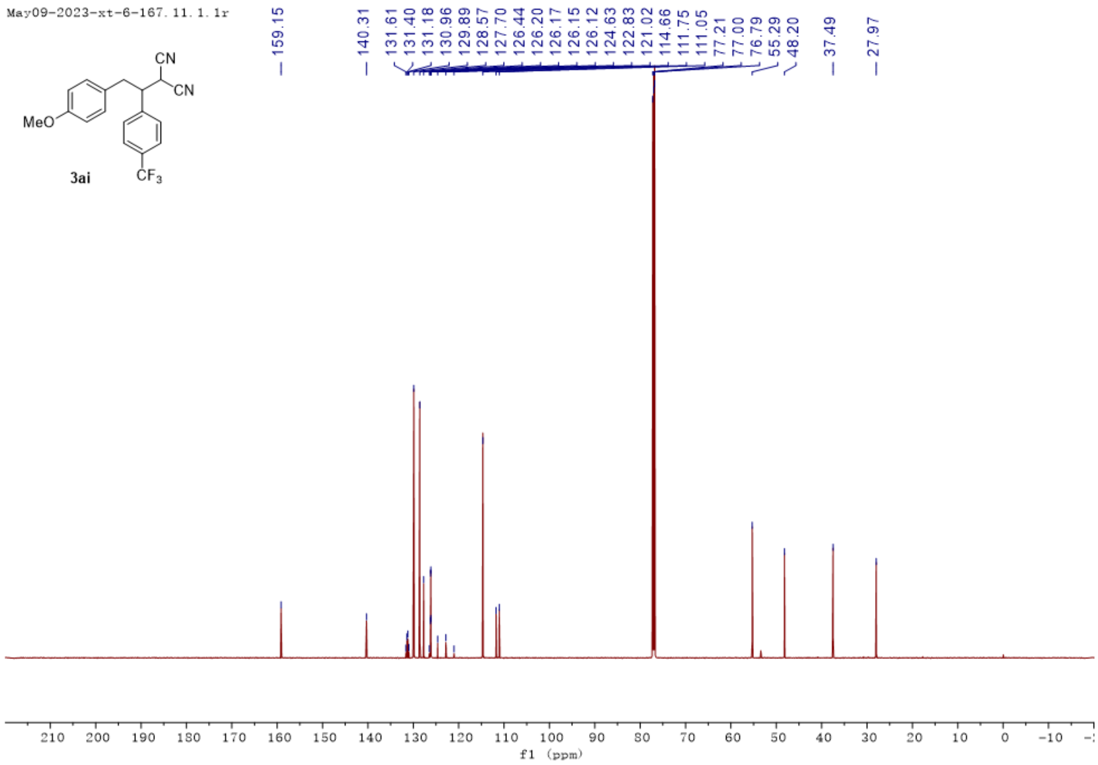

^{13}C NMR (151 MHz, $CDCl_3$) of **3ai**

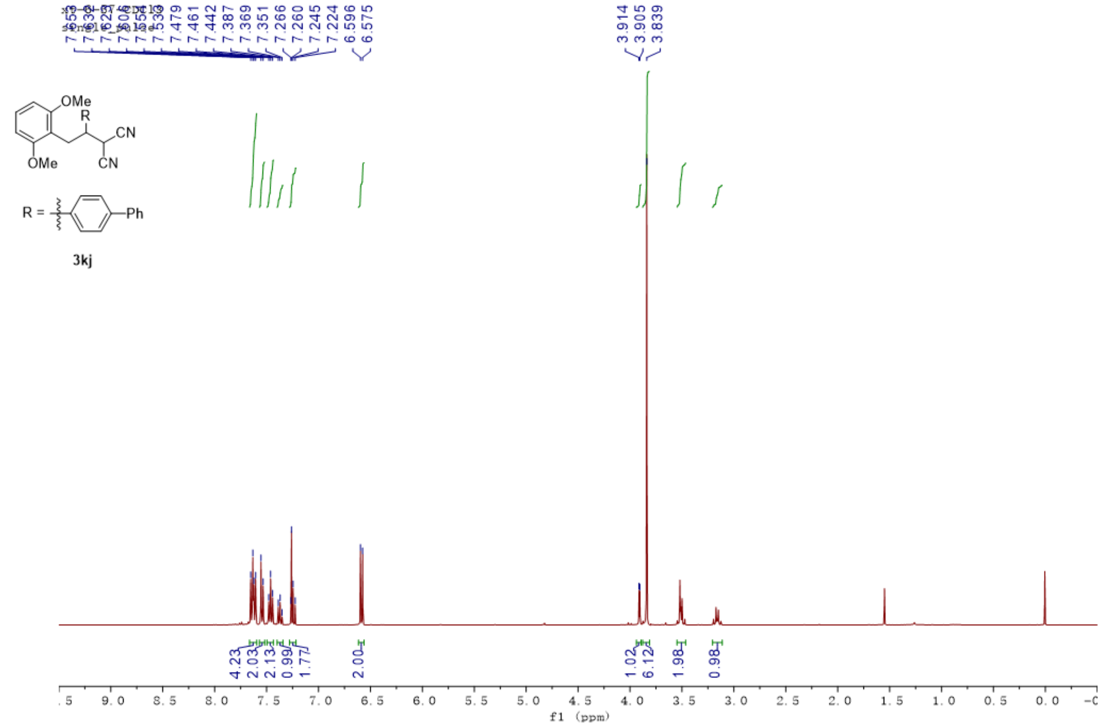

^{1}H NMR (400 MHz, $CDCl_3$) of **3kj**

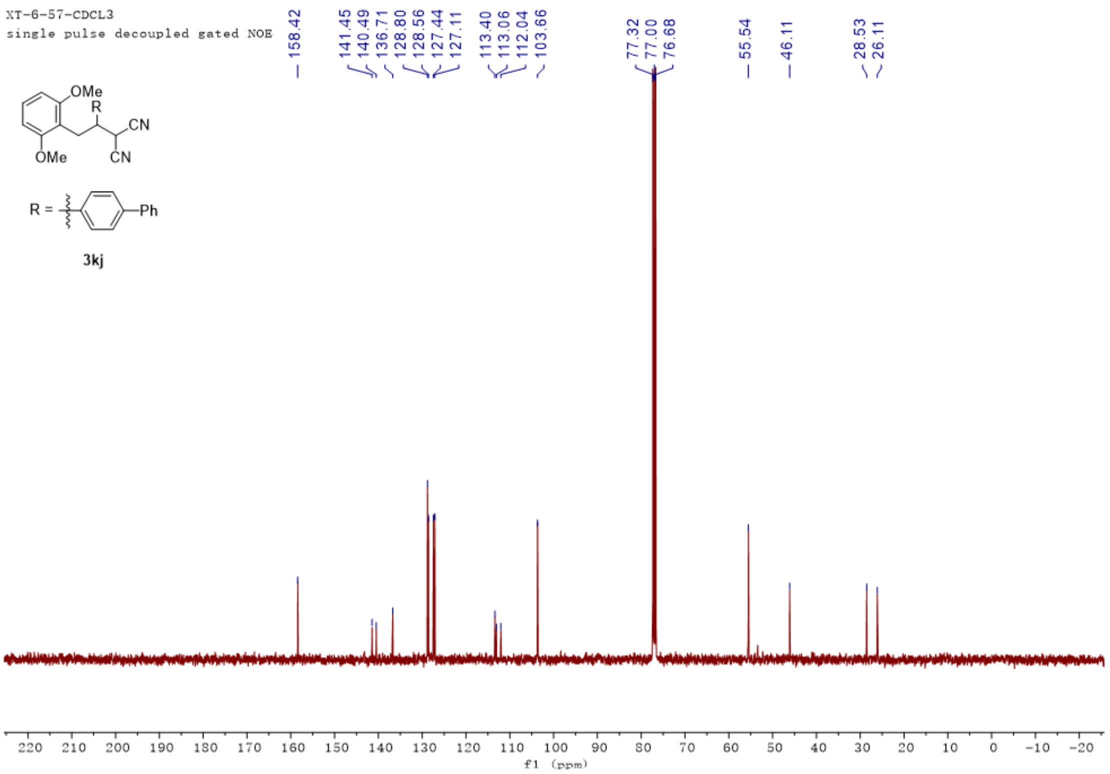

^{13}C NMR (101 MHz, $CDCl_3$) of **3kj**

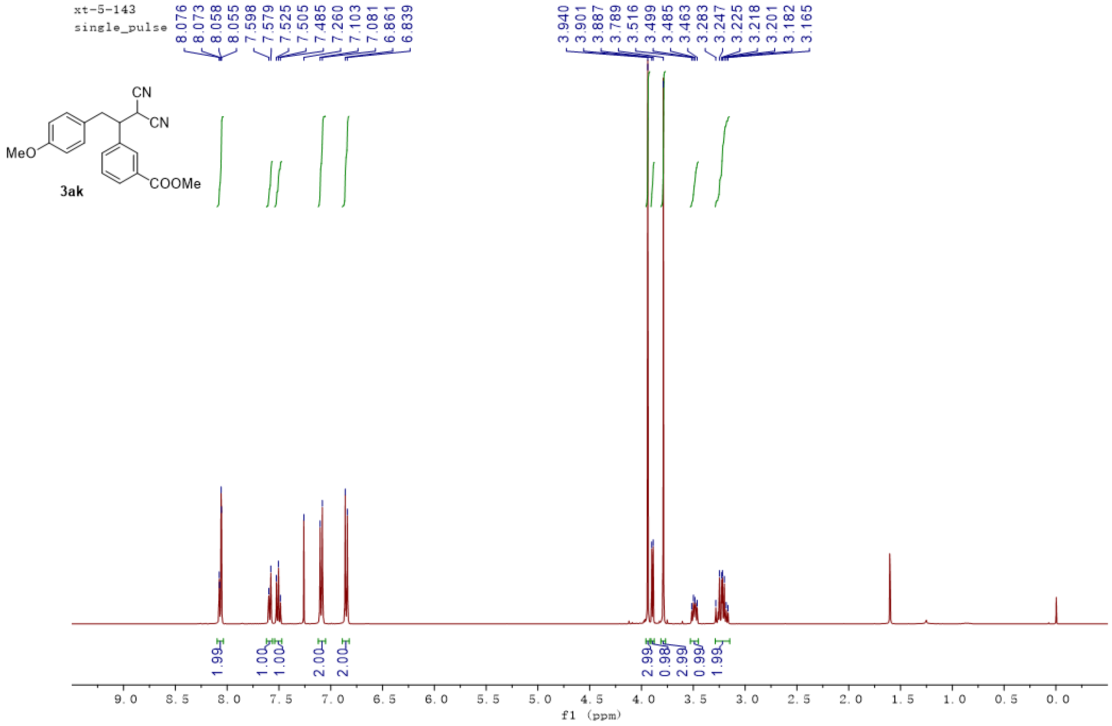

^{1}H NMR (400 MHz, $CDCl_3$) of **3ak**

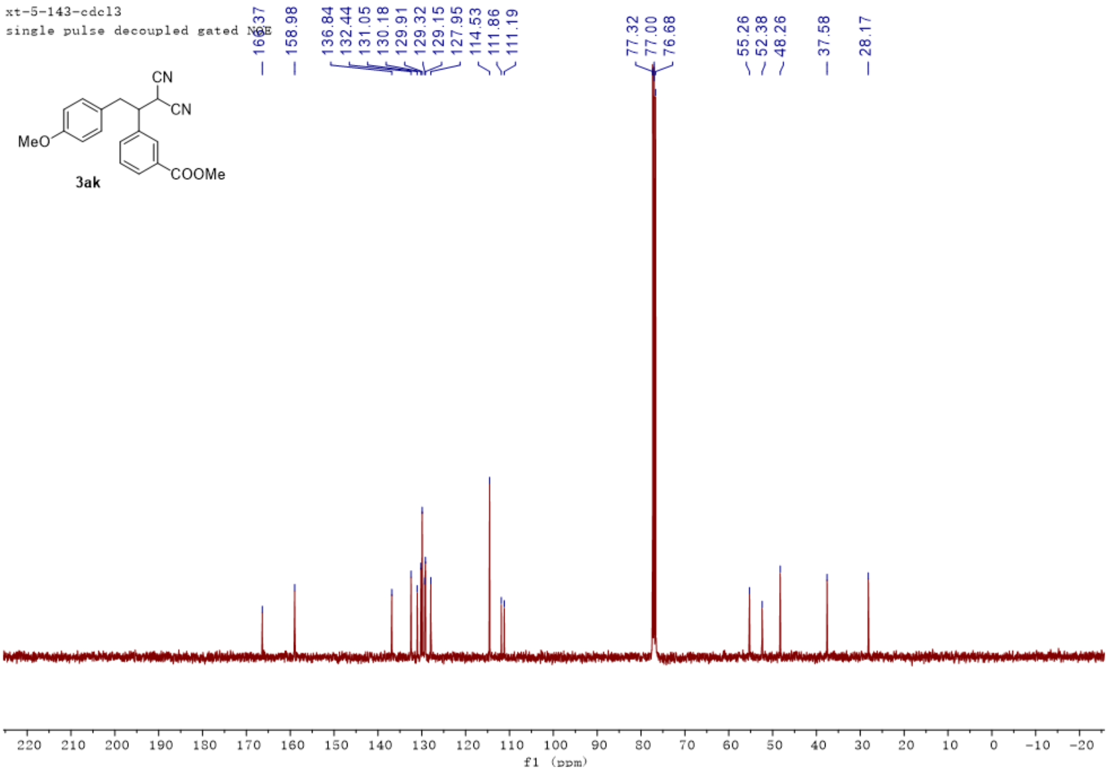

^{13}C NMR (101 MHz, $CDCl_3$) of **3ak**

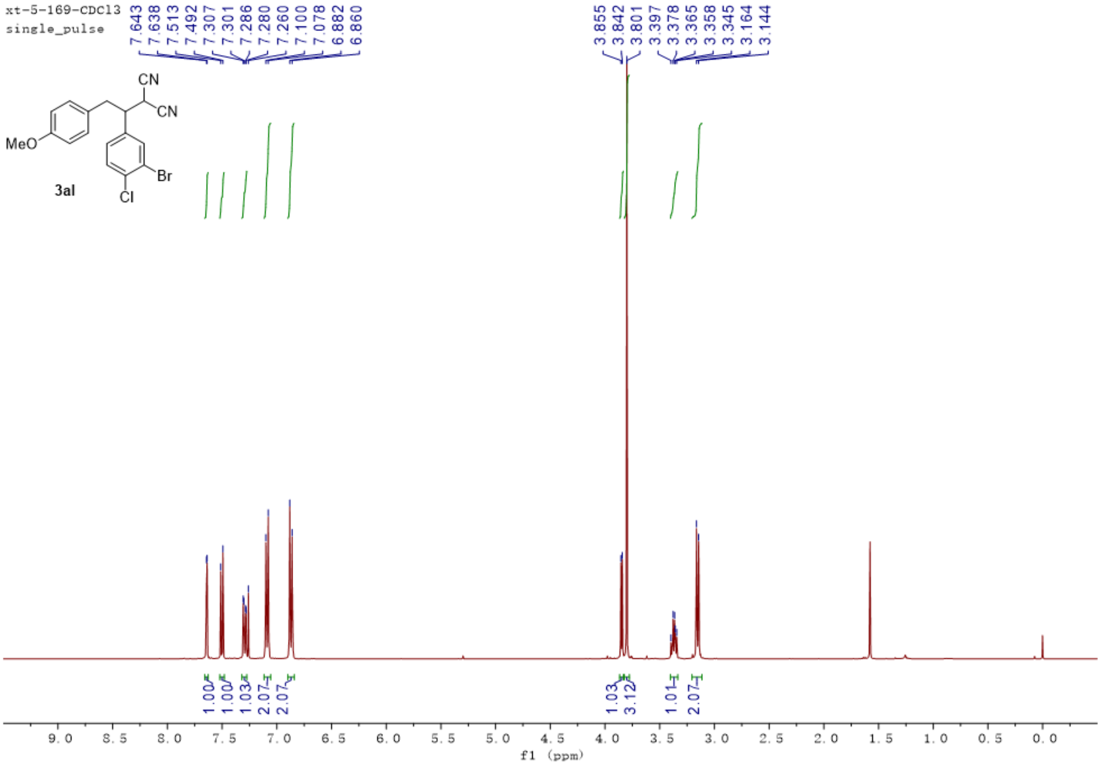

^{1}H NMR (400 MHz, $CDCl_3$) of **3al**

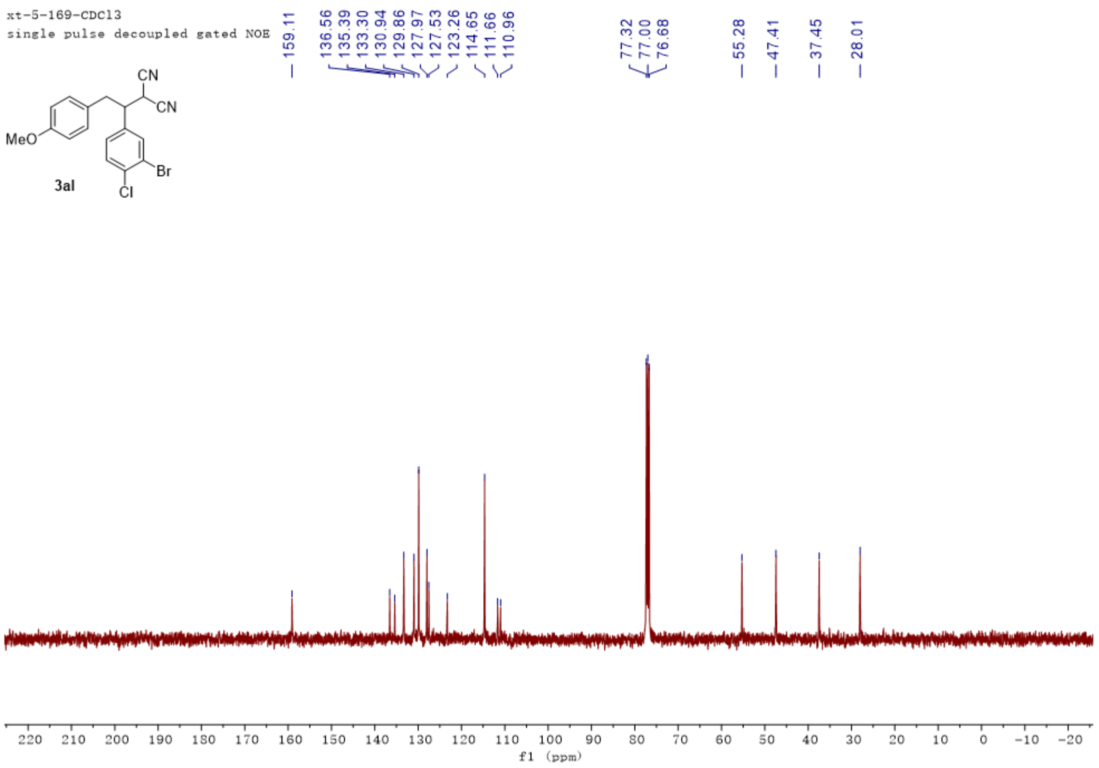

^{13}C NMR (101 MHz, CDCl$_3$) of **3al**

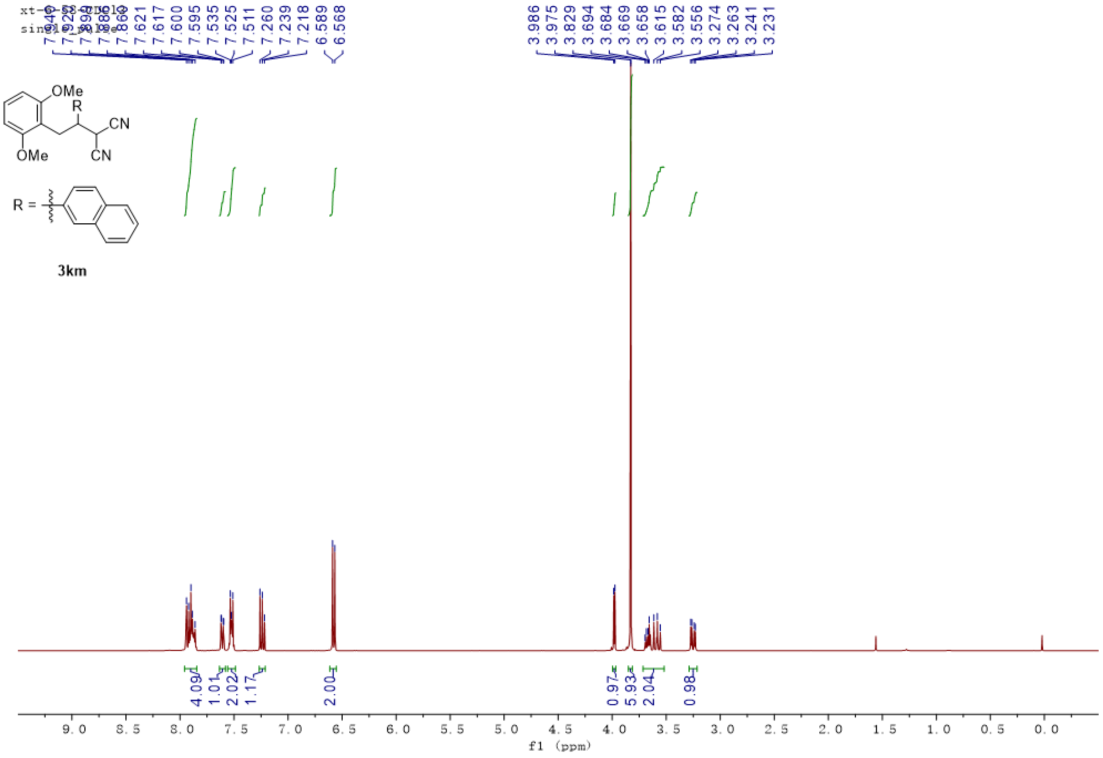

^{1}H NMR (400 MHz, CDCl$_3$) of **3km**

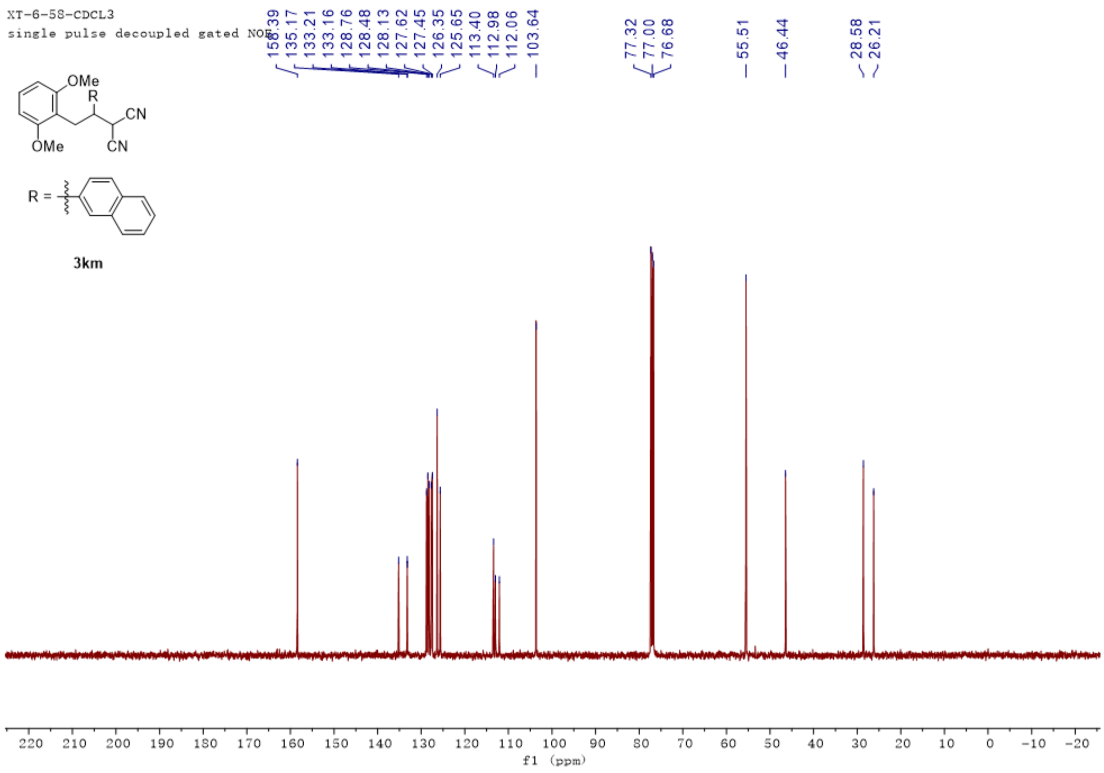

^{13}C NMR (101 MHz, $CDCl_3$) of **3km**

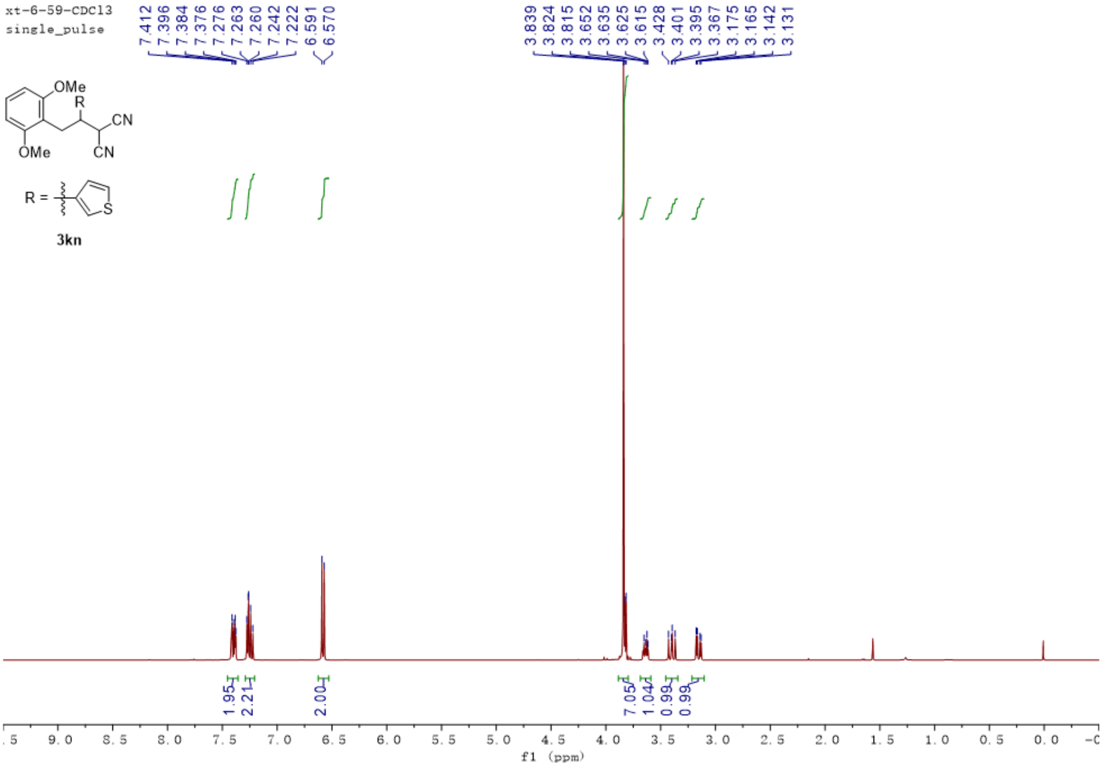

^{1}H NMR (400 MHz, $CDCl_3$) of **3kn**

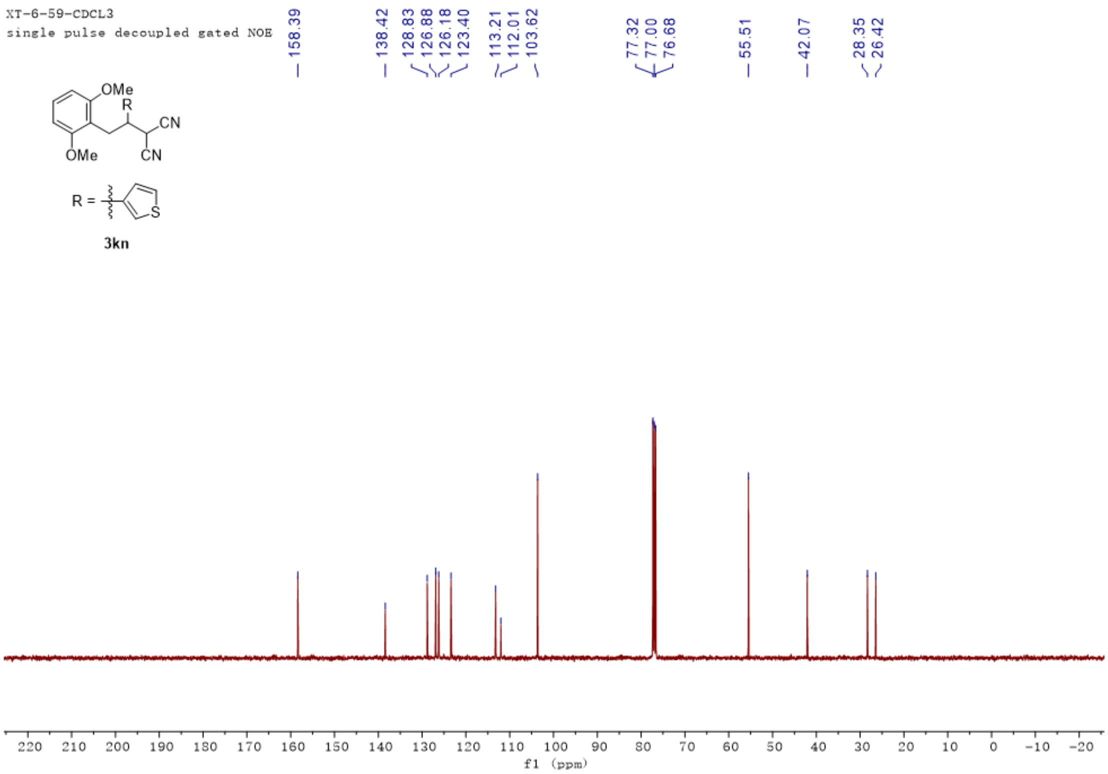

^{13}C NMR (101 MHz, CDCl$_3$) of **3kn**

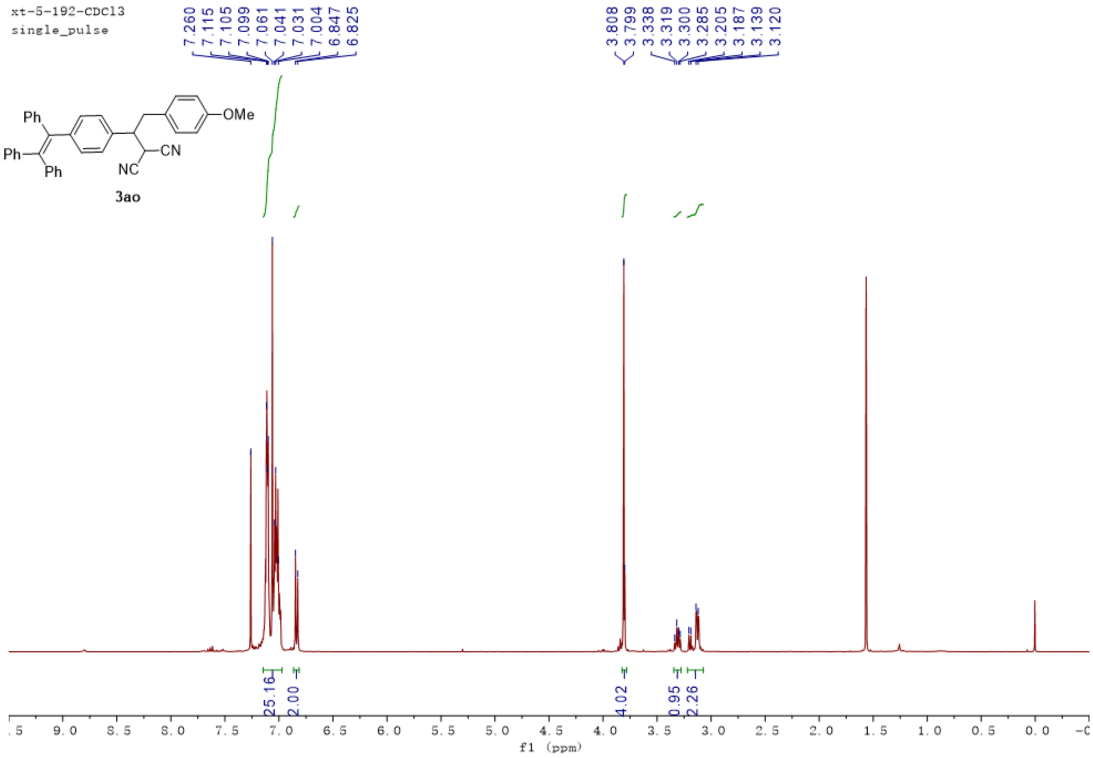

^{1}H NMR (400 MHz, CDCl$_3$) of **3ao**

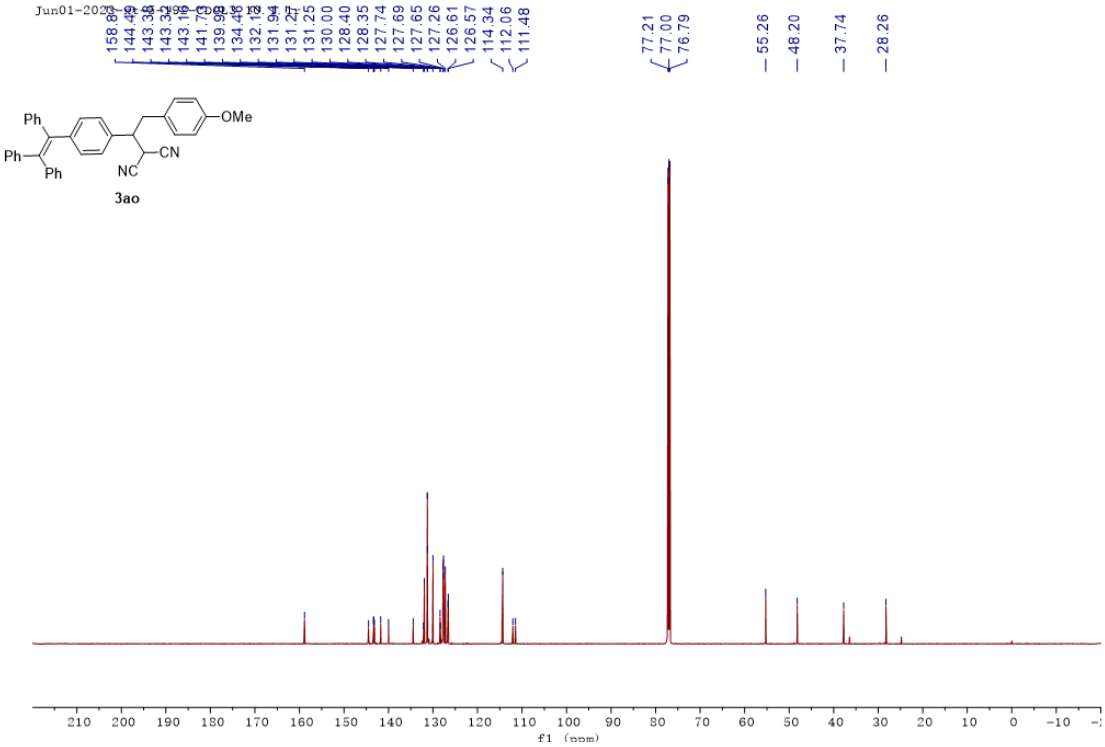

^{13}C NMR (151 MHz, $CDCl_3$) of **3ao**

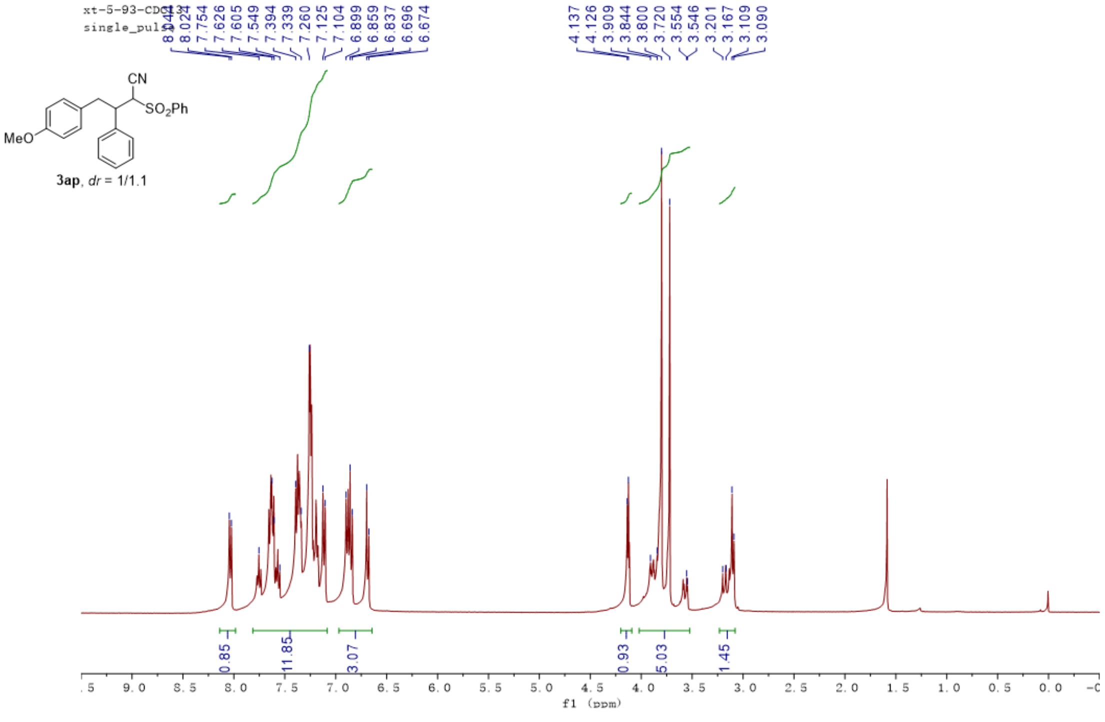

^{1}H NMR (400 MHz, $CDCl_3$) of **3ap**

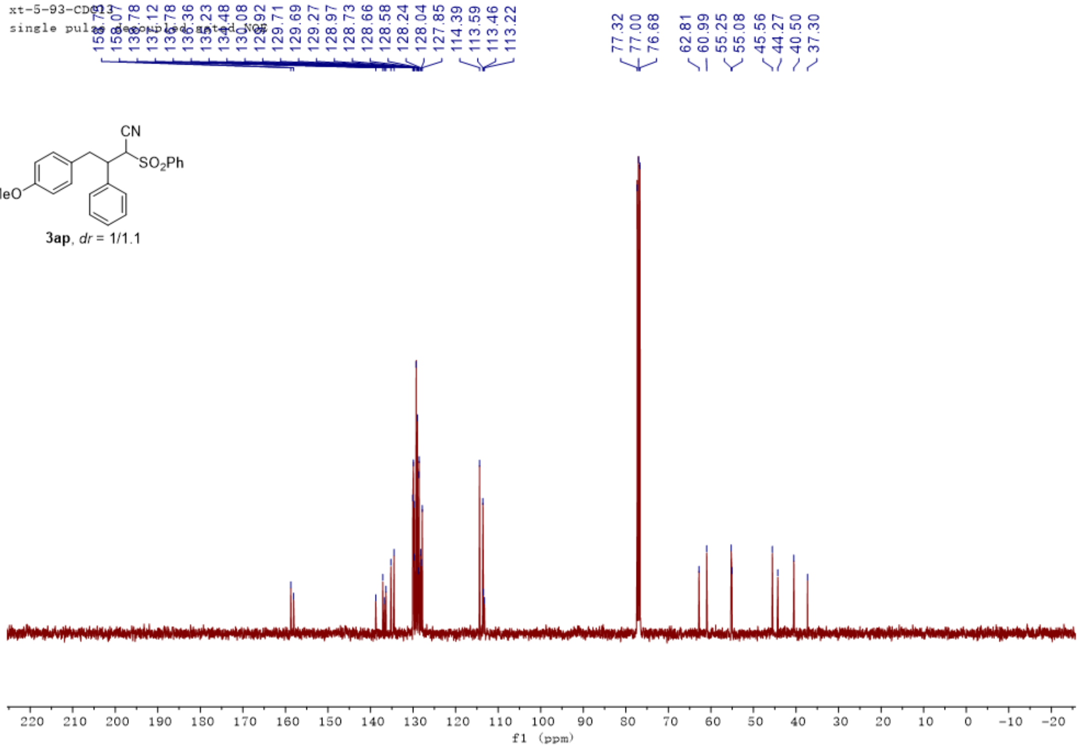

^{13}C NMR (101 MHz, CDCl$_3$) of **3ap**

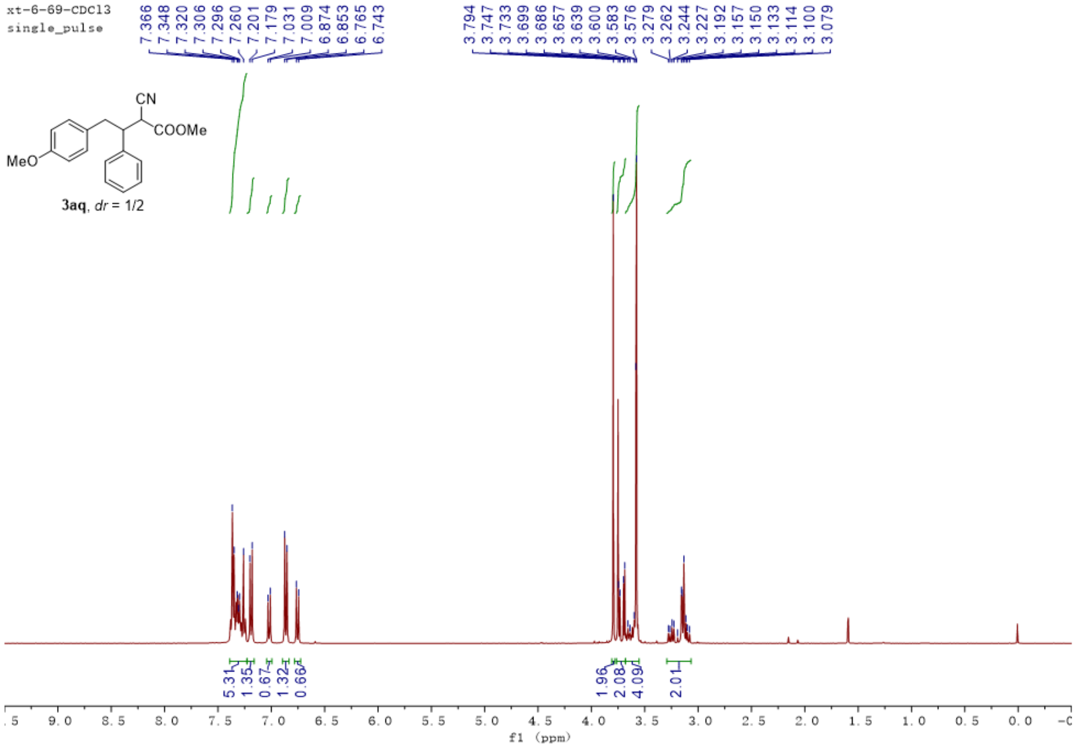

^{1}H NMR (400 MHz, CDCl$_3$) of **3aq**

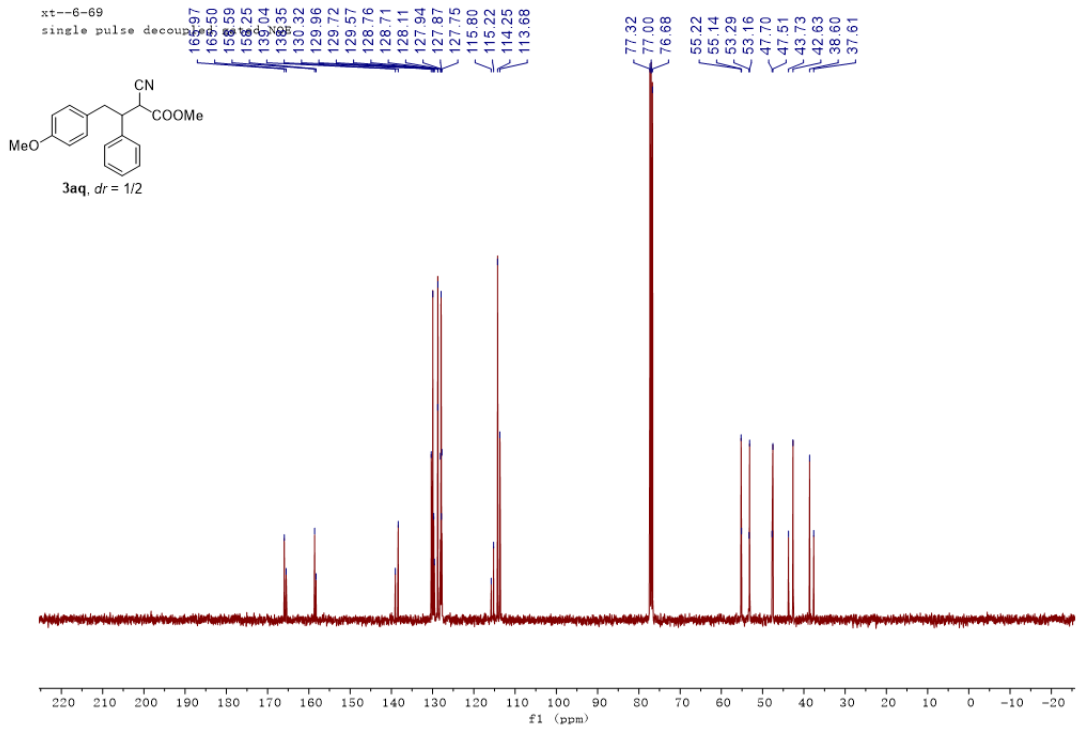

^{13}C NMR (101 MHz, $CDCl_3$) of **3aq**

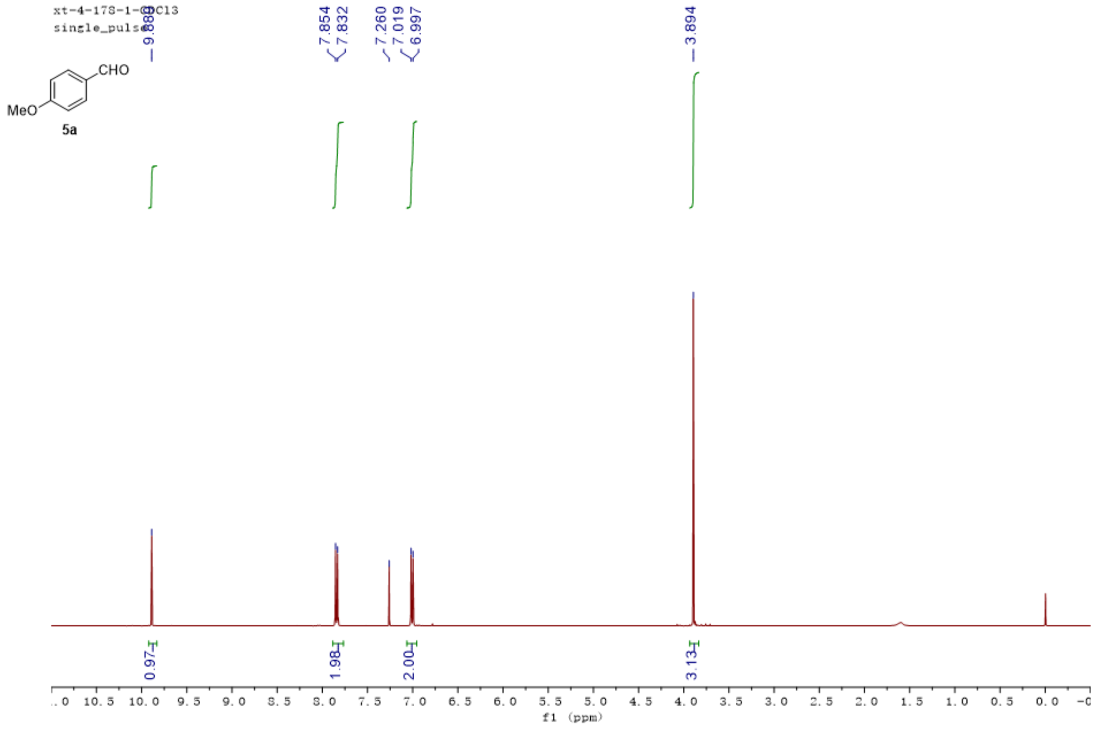

^{1}H NMR (400 MHz, $CDCl_3$) of **5a**

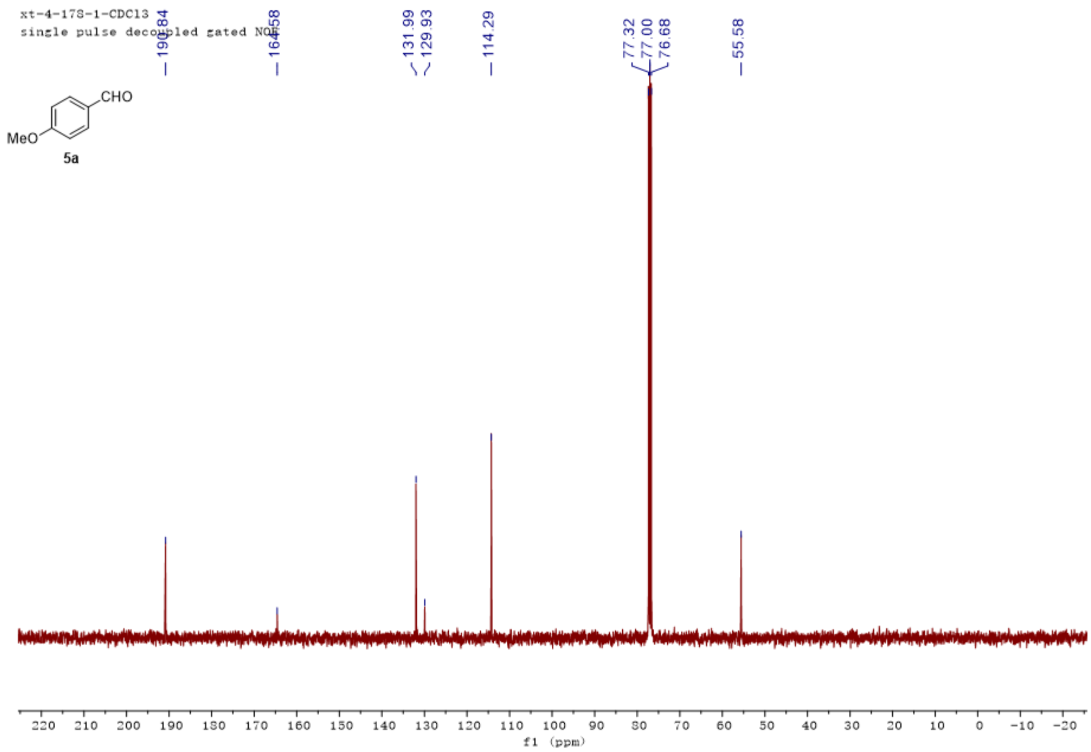

^{13}C NMR (101 MHz, $CDCl_3$) of **5a**

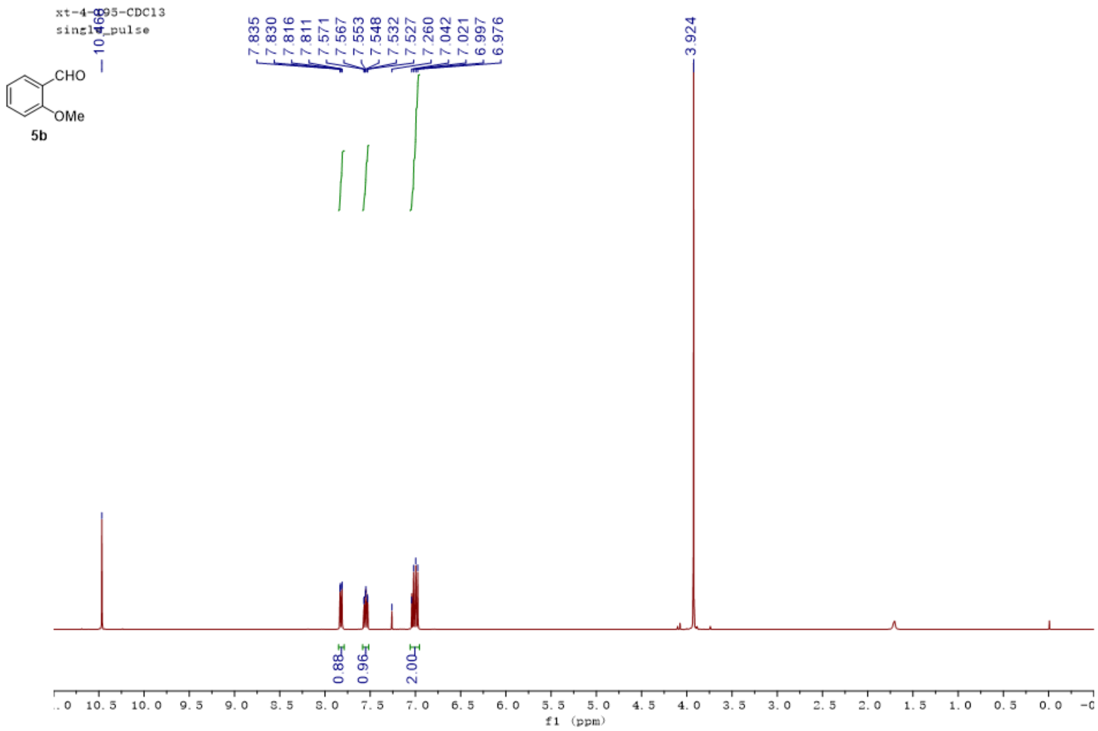

^{1}H NMR (400 MHz, $CDCl_3$) of **5b**

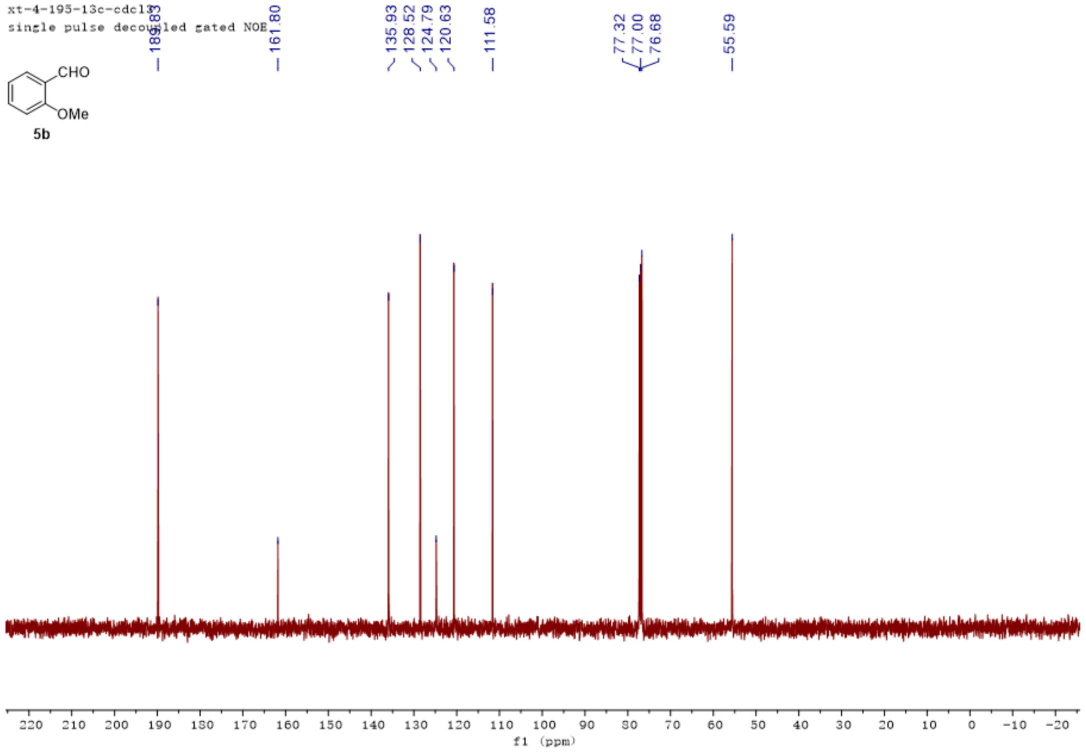

^{13}C NMR (101 MHz, $CDCl_3$) of **5b**

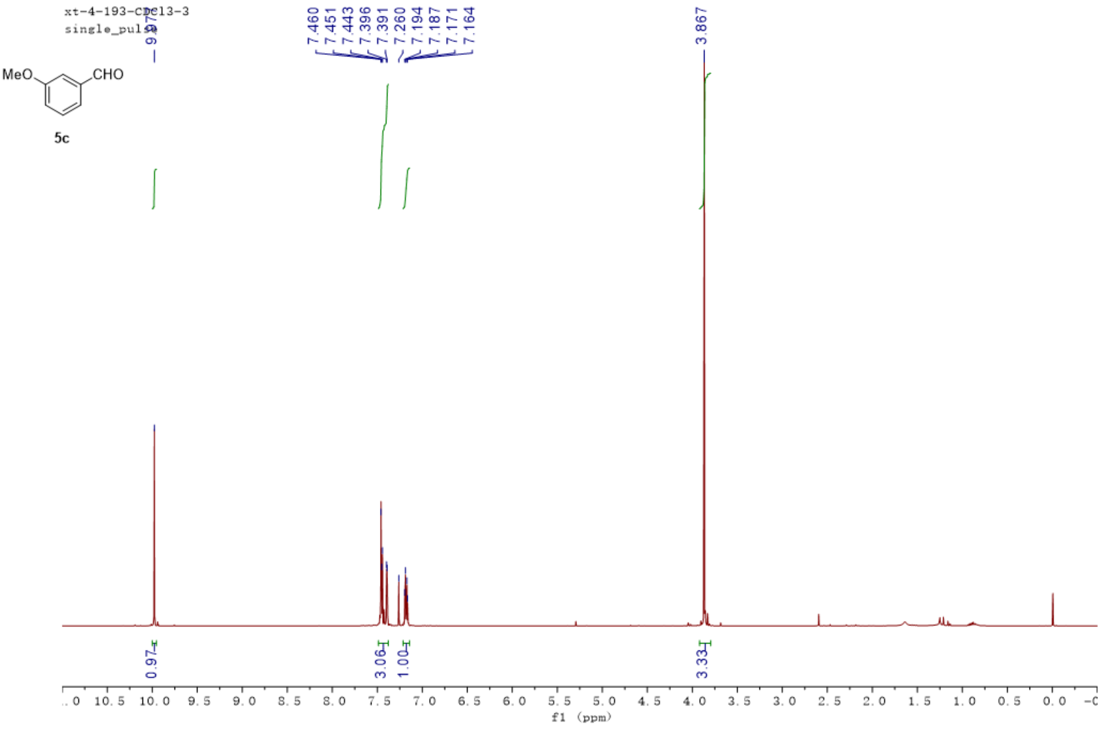

^{1}H NMR (400 MHz, $CDCl_3$) of **5c**

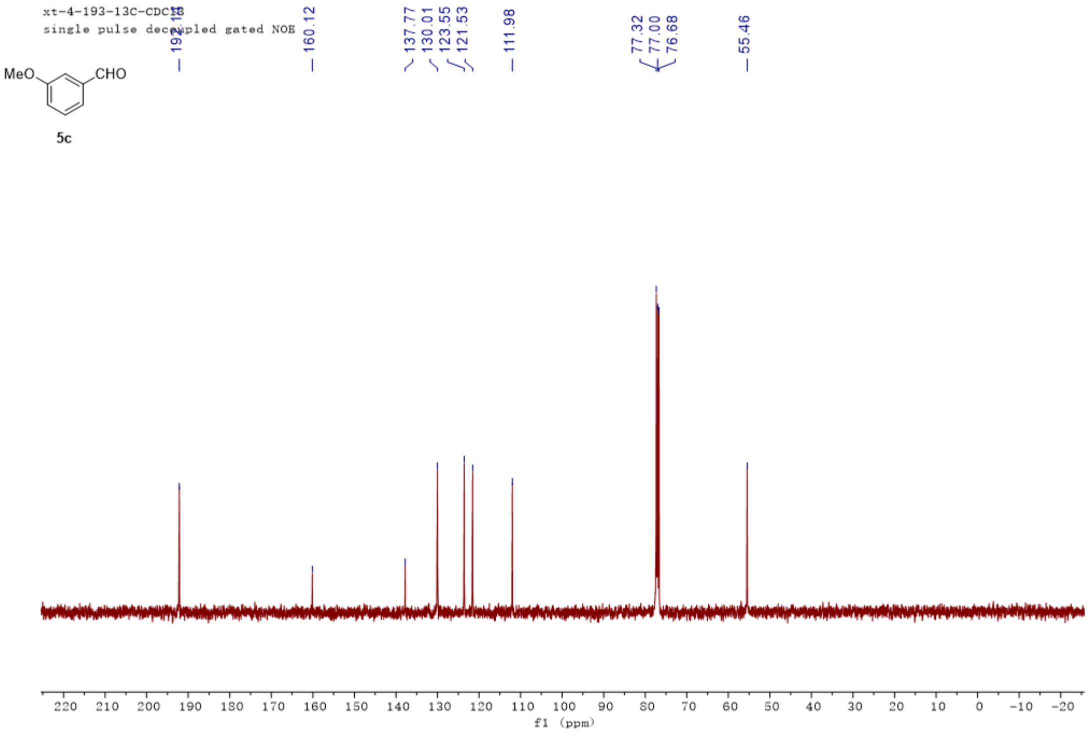

^{13}C NMR (101 MHz, $CDCl_3$) of **5c**

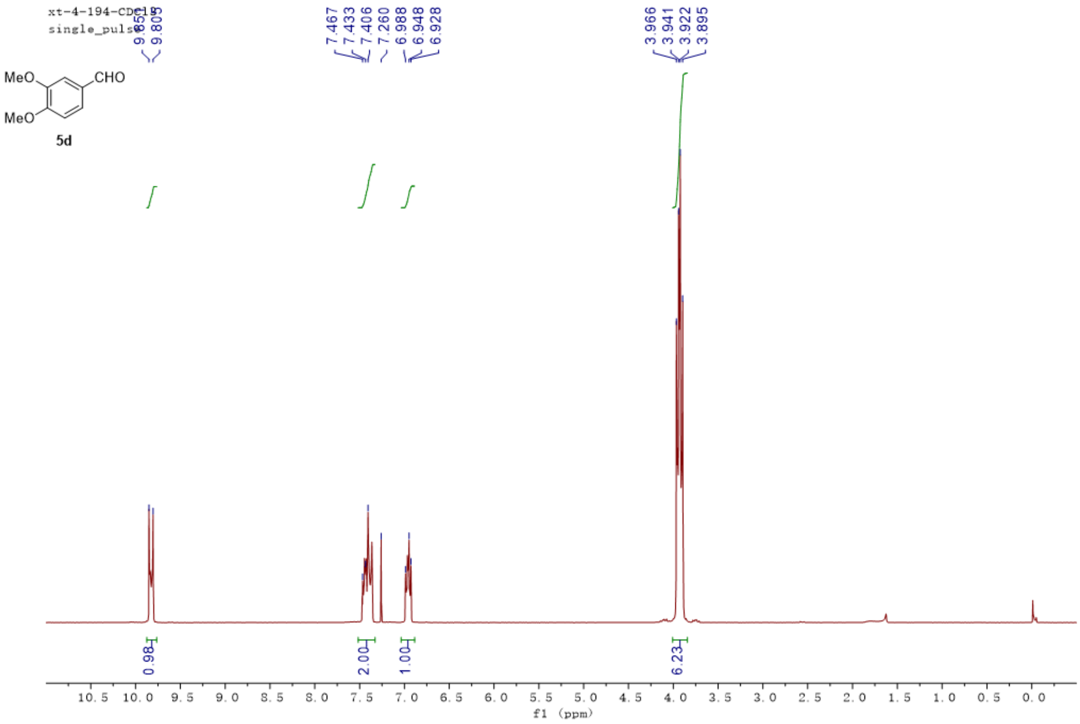

^{1}H NMR (400 MHz, $CDCl_3$) of **5d**

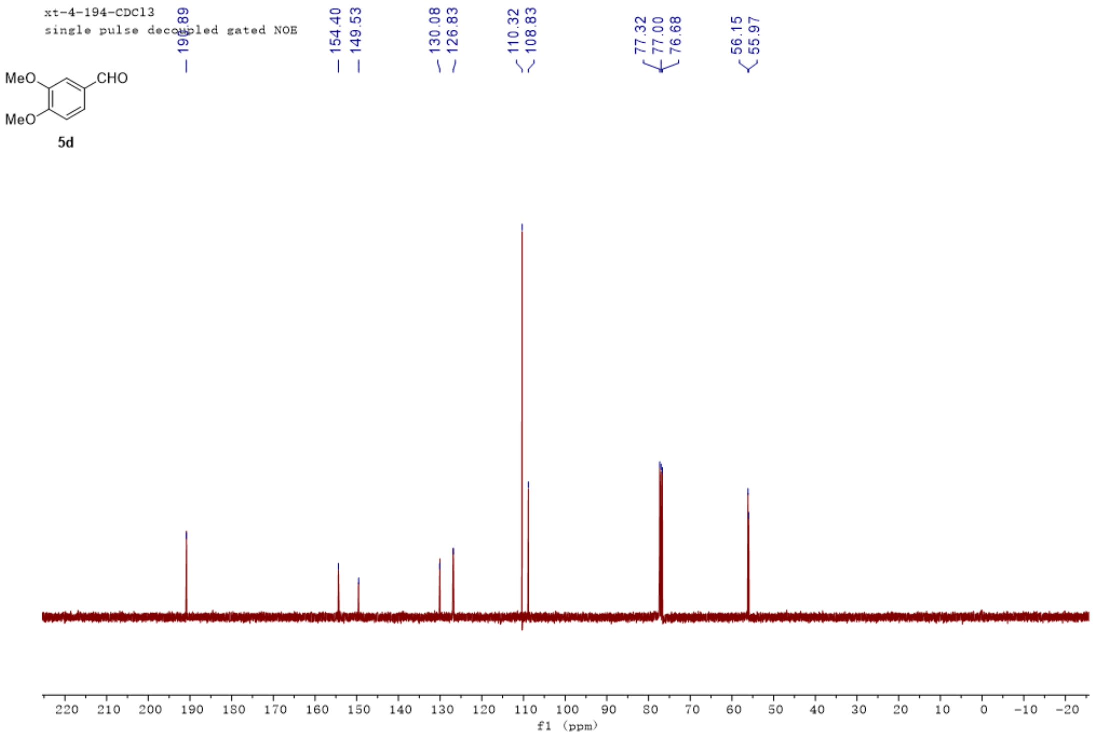

^{13}C NMR (101 MHz, CDCl$_3$) of **5d**

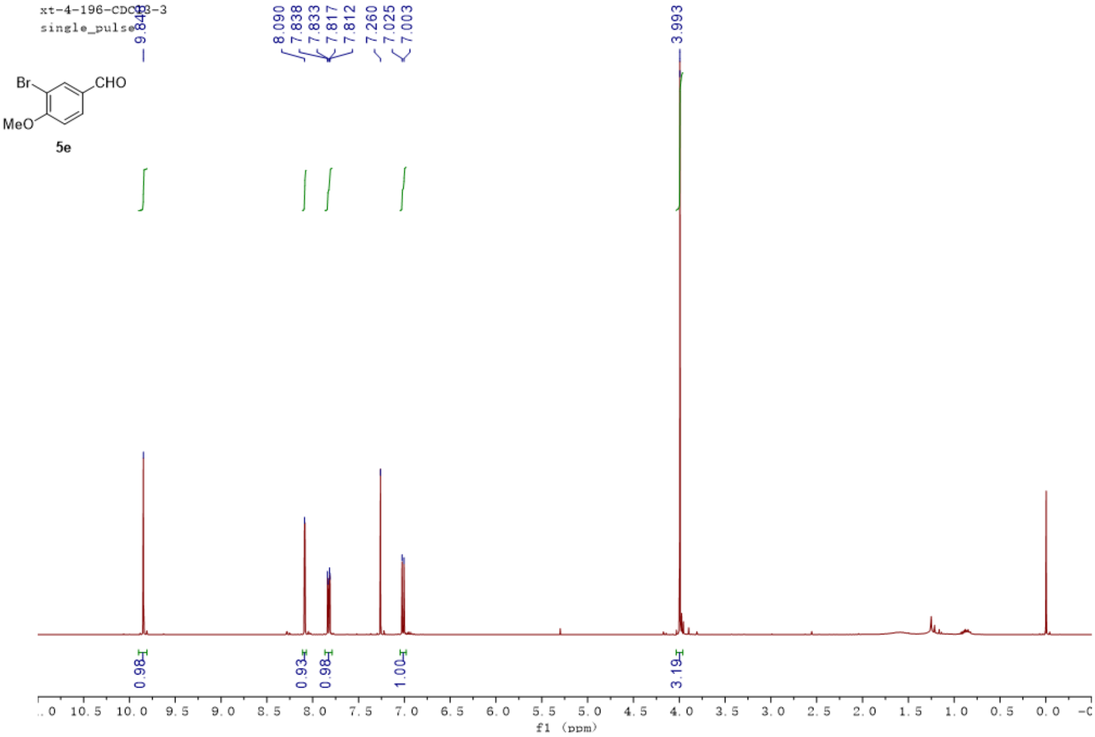

^{1}H NMR (400 MHz, CDCl$_3$) of **5e**

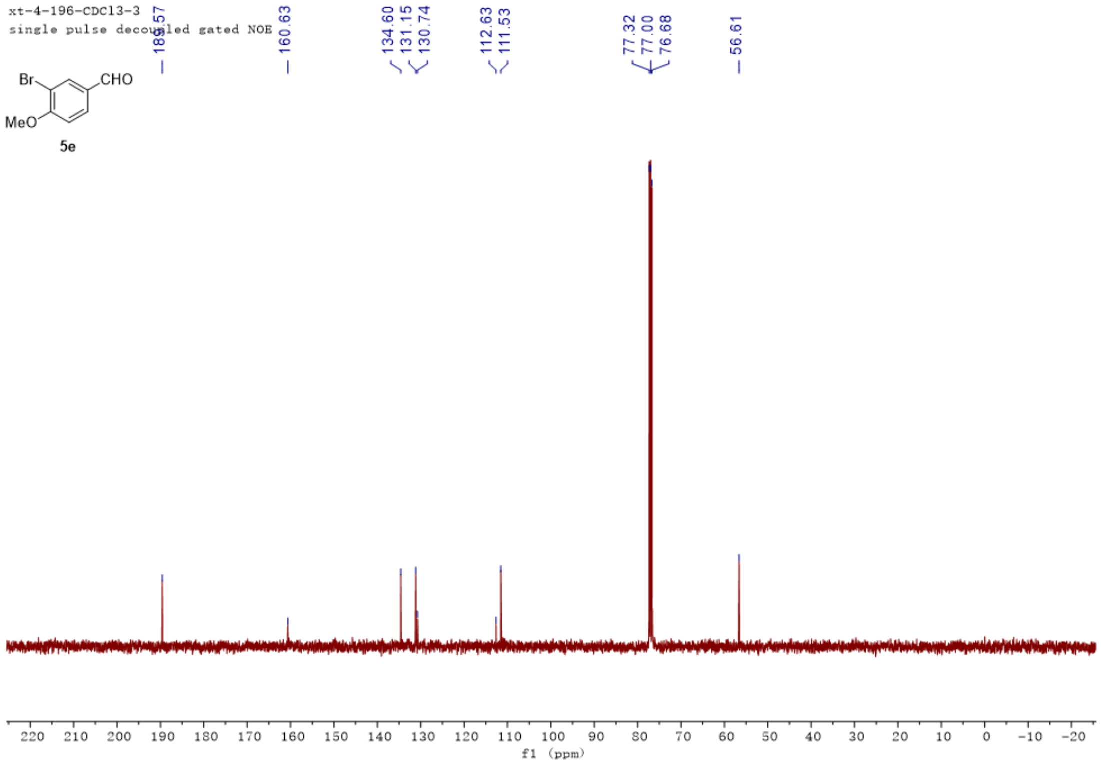

^{13}C NMR (101 MHz, $CDCl_3$) of **5e**

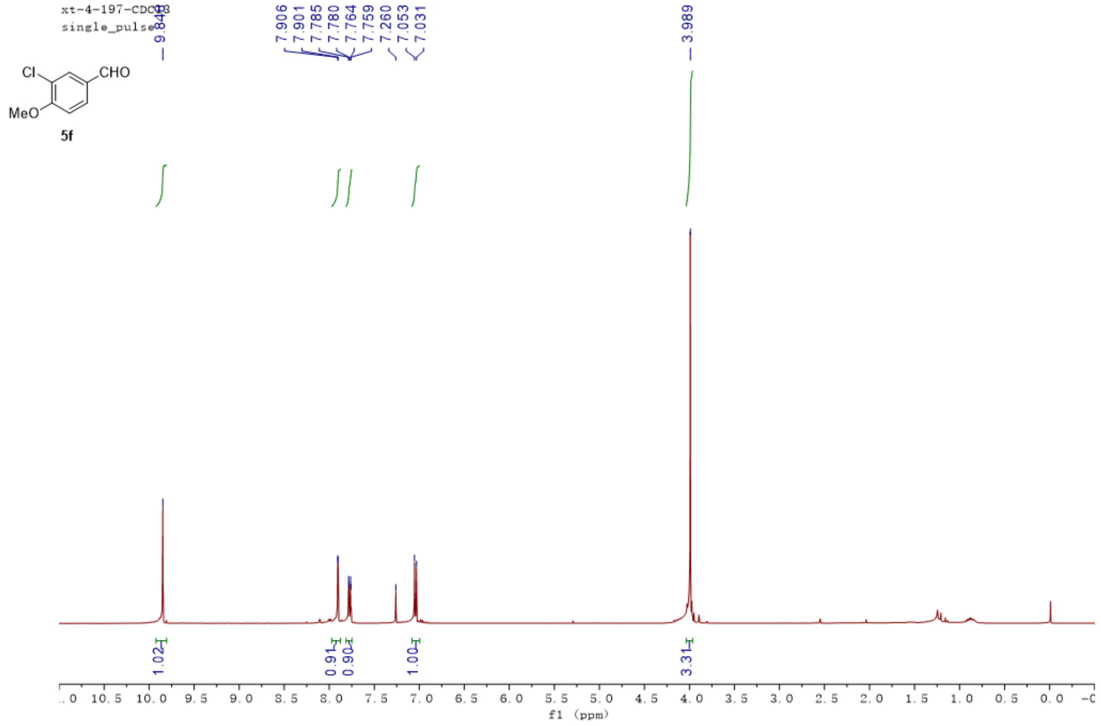

^{1}H NMR (400 MHz, $CDCl_3$) of **5f**

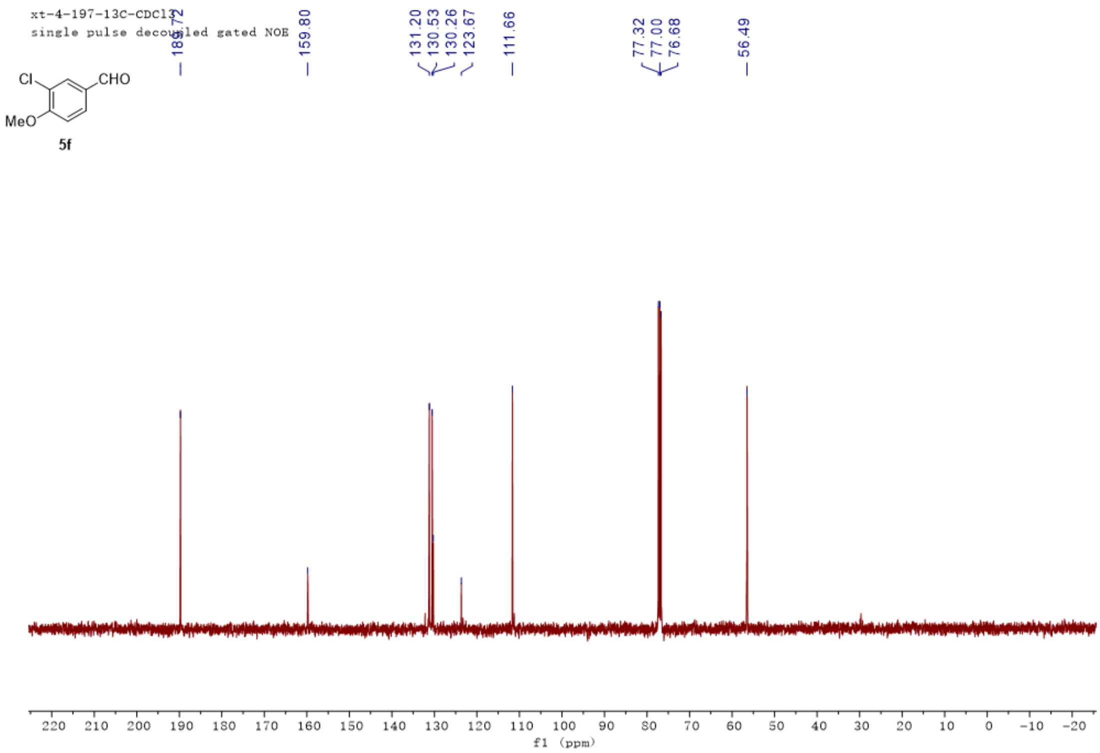

^{13}C NMR (101 MHz, $CDCl_3$) of **5f**

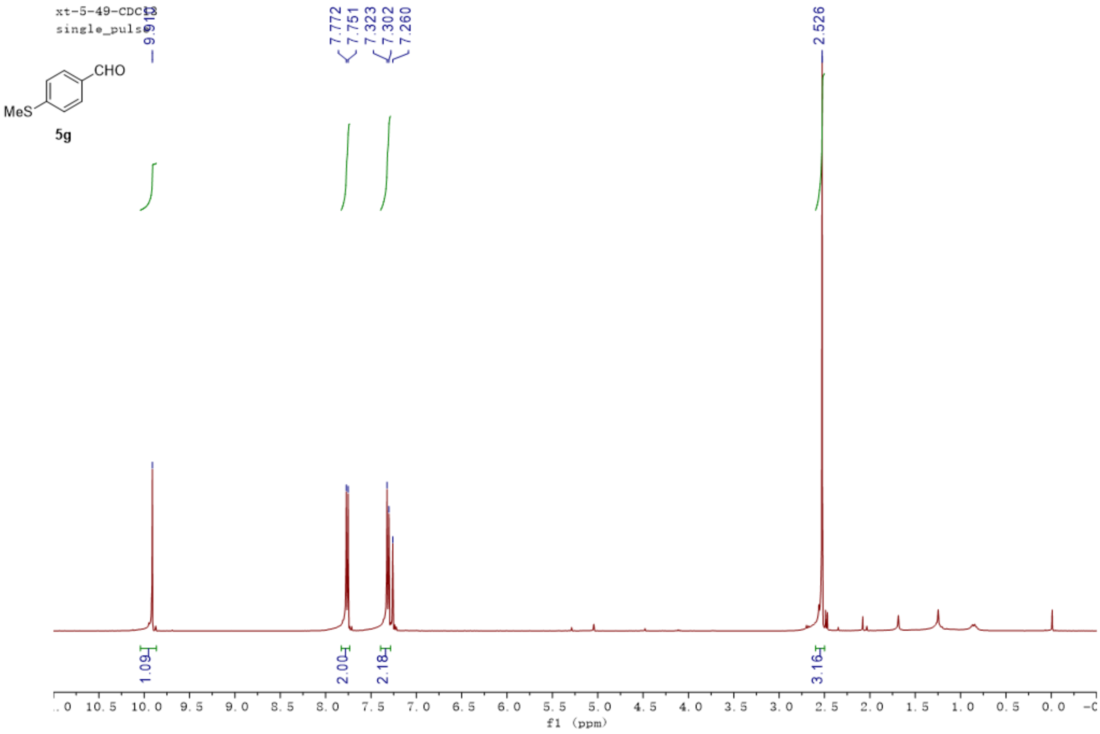

^{1}H NMR (400 MHz, $CDCl_3$) of **5g**

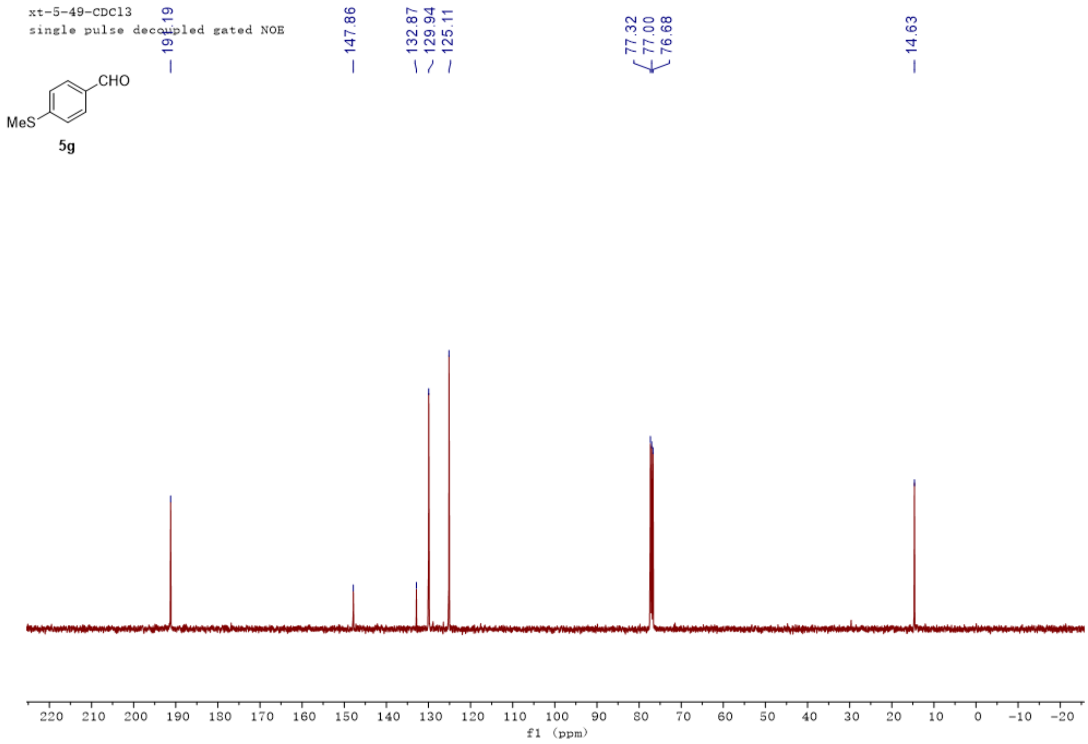

^{13}C NMR (101 MHz, $CDCl_3$) of **5g**

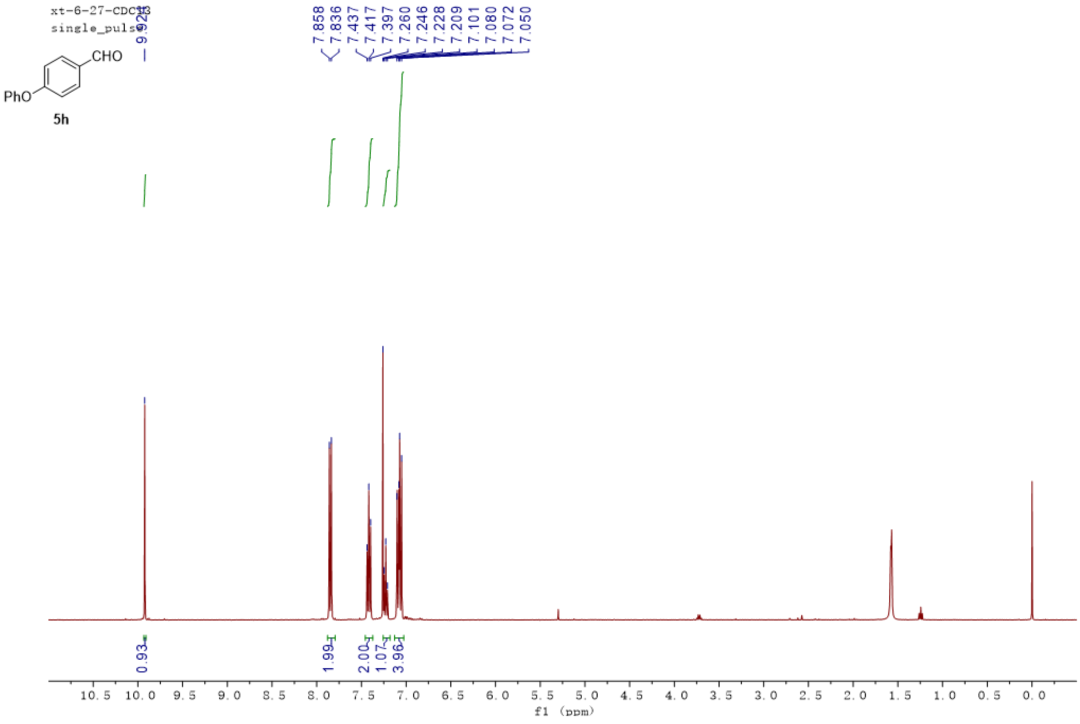

^{1}H NMR (400 MHz, $CDCl_3$) of **5h**

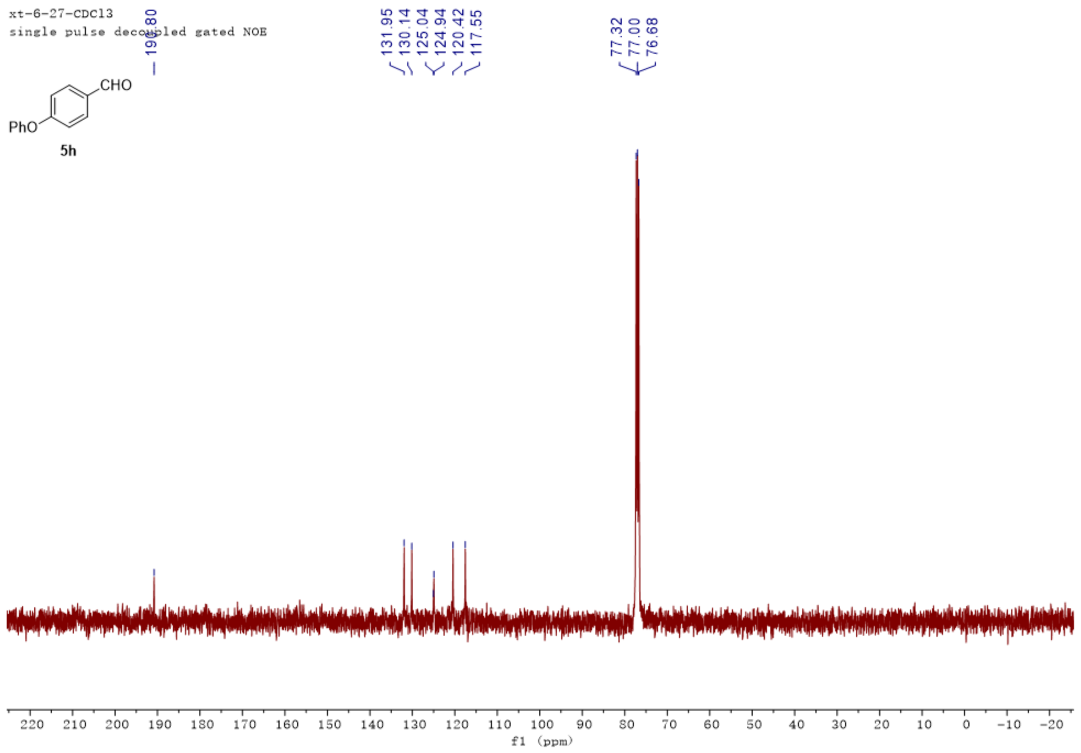

^{13}C NMR (101 MHz, $CDCl_3$) of **5h**

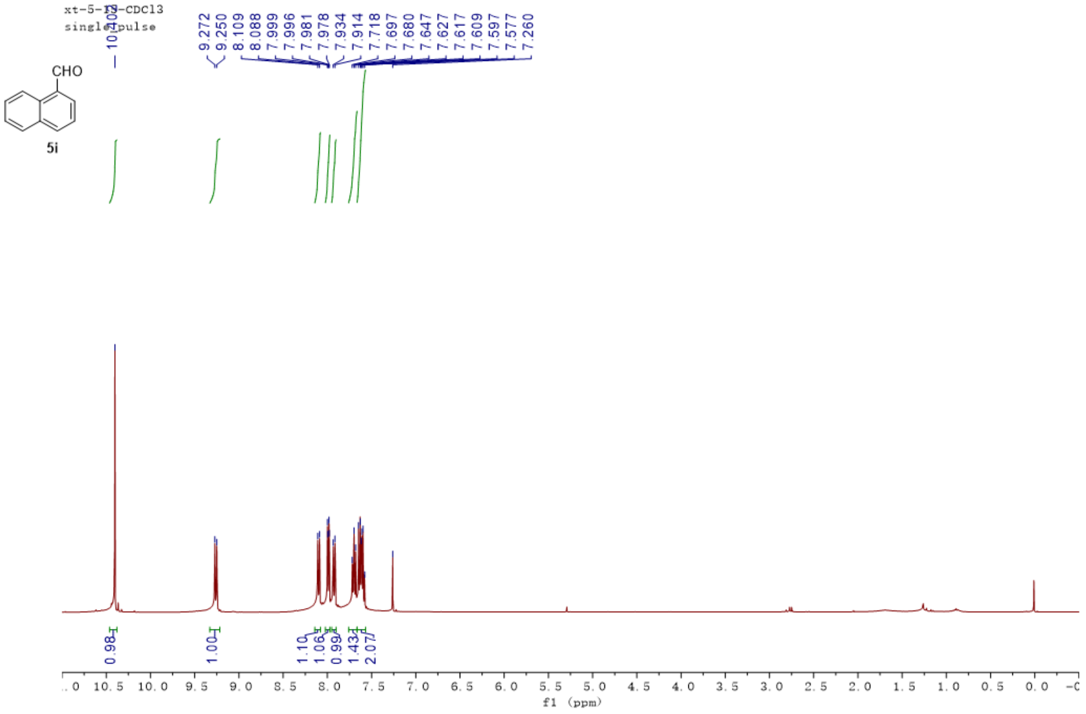

^{1}H NMR (400 MHz, $CDCl_3$) of **5i**

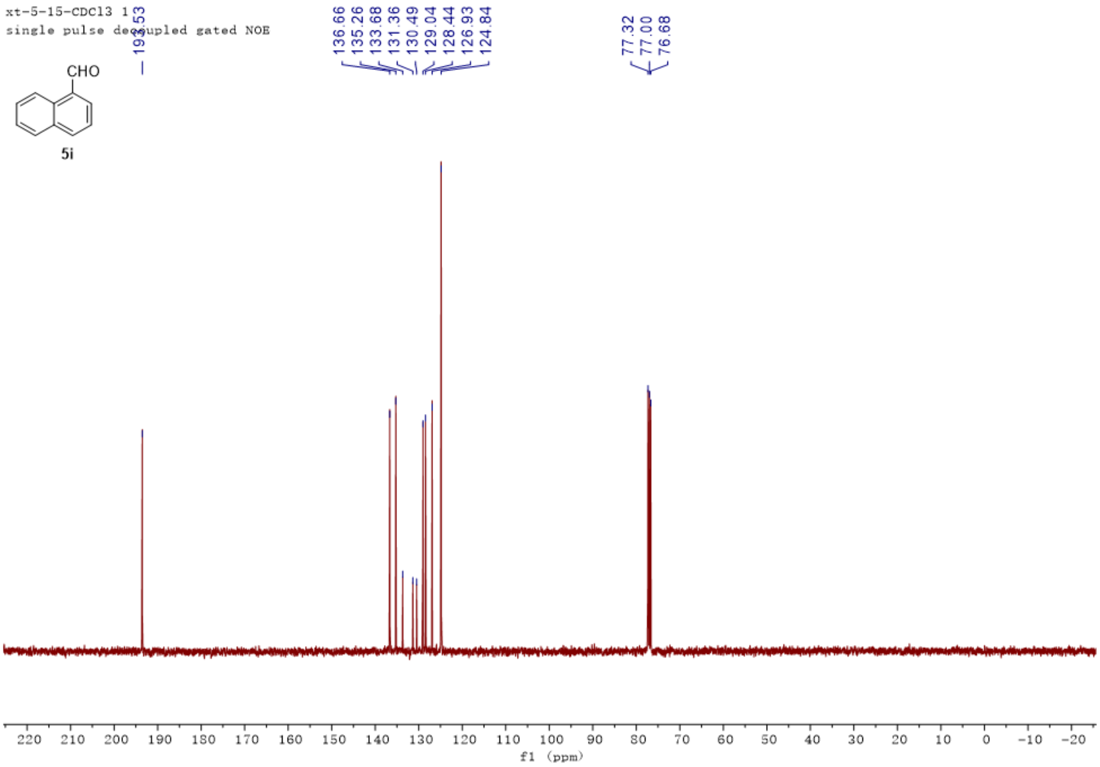

^{13}C NMR (101 MHz, $CDCl_3$) of **5i**

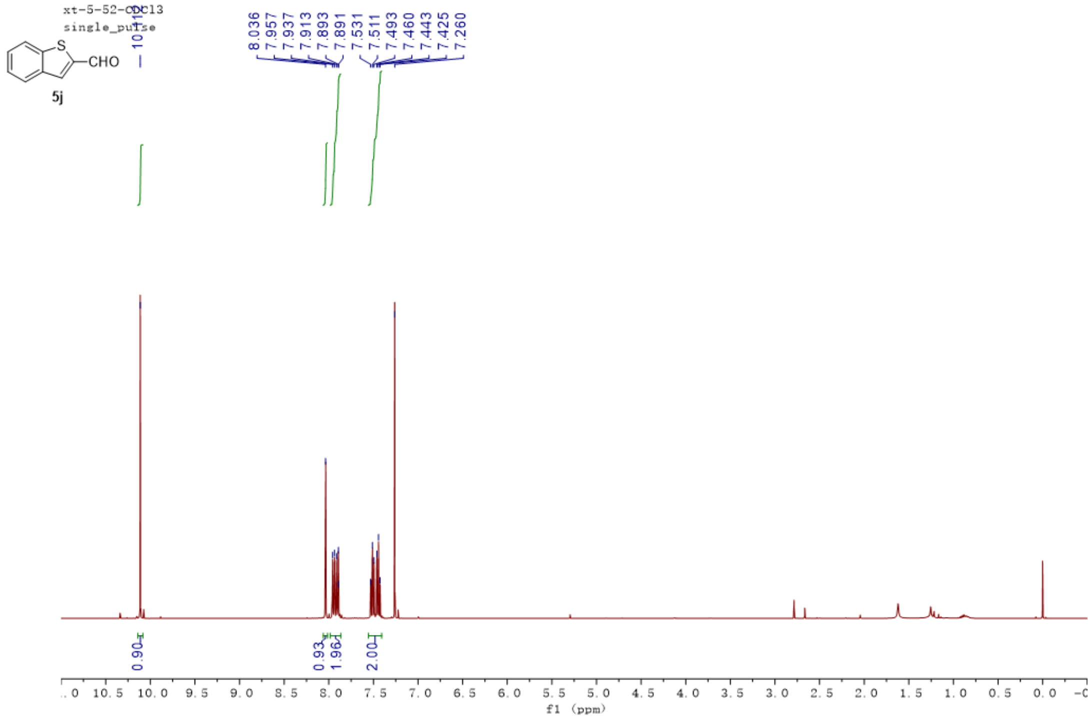

^{1}H NMR (400 MHz, $CDCl_3$) of **5j**

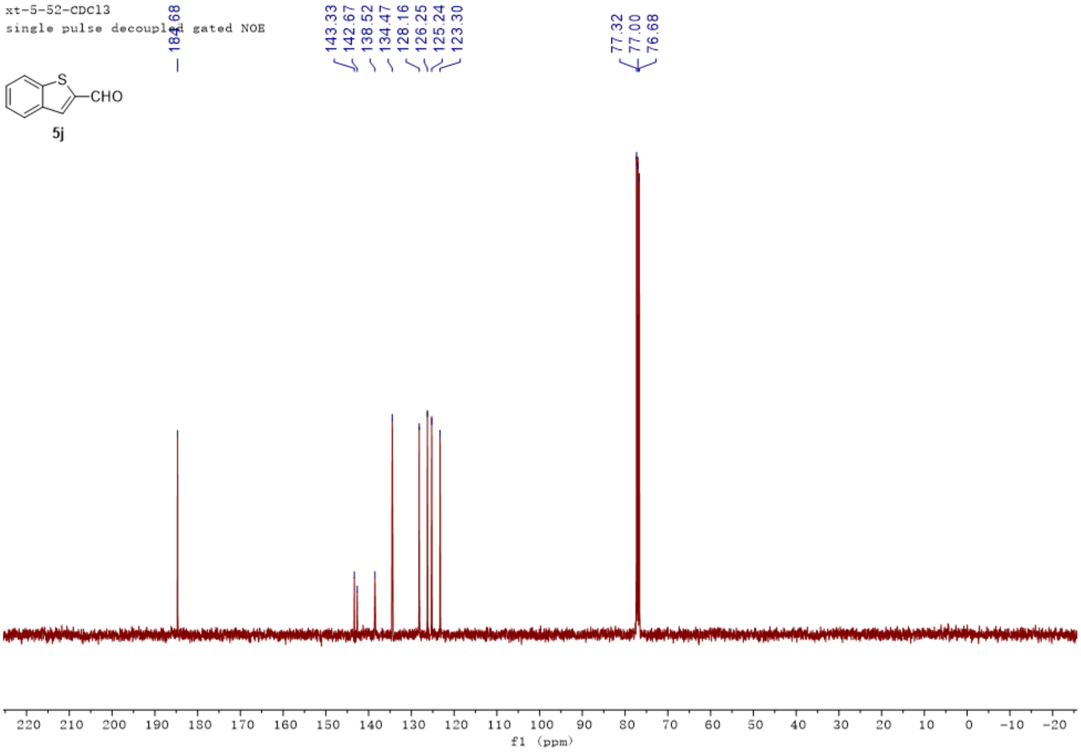

^{13}C NMR (101 MHz, $CDCl_3$) of **5j**

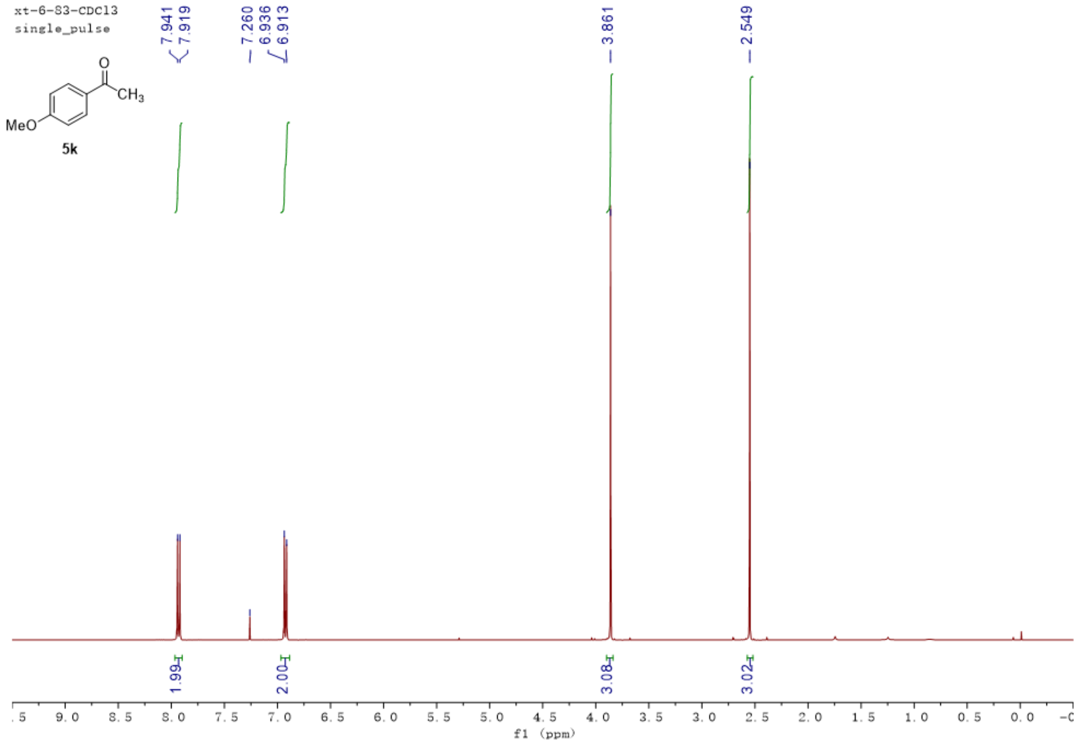

^{1}H NMR (400 MHz, $CDCl_3$) of **5k**

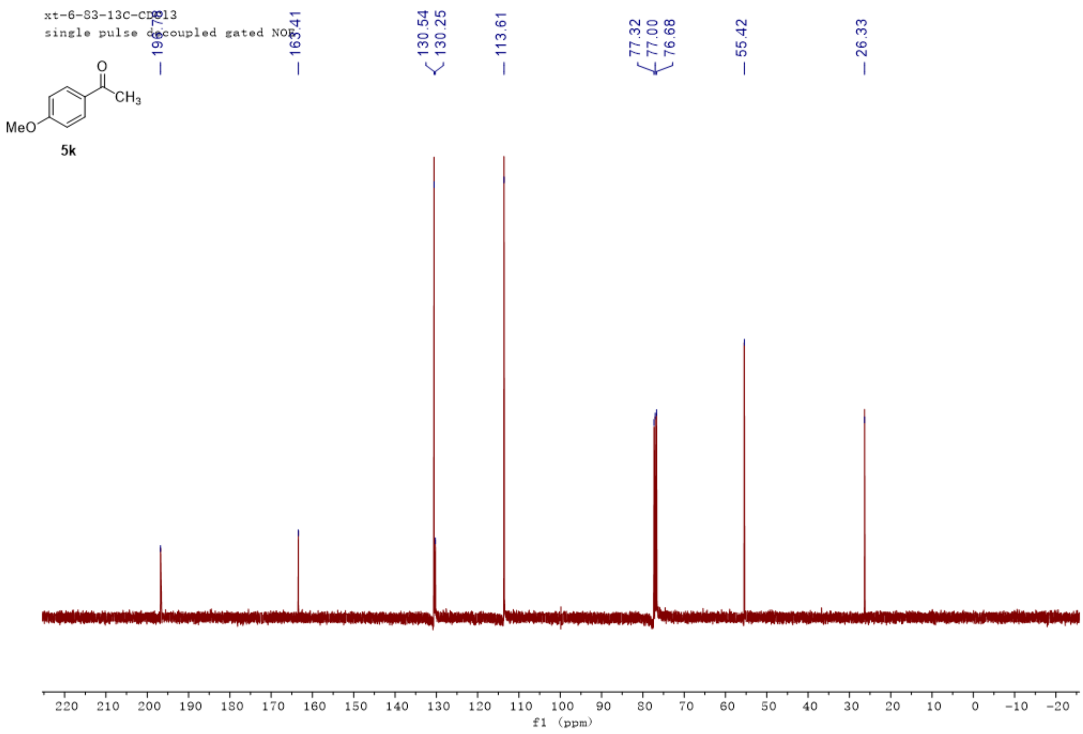

^{13}C NMR (101 MHz, $CDCl_3$) of **5k**

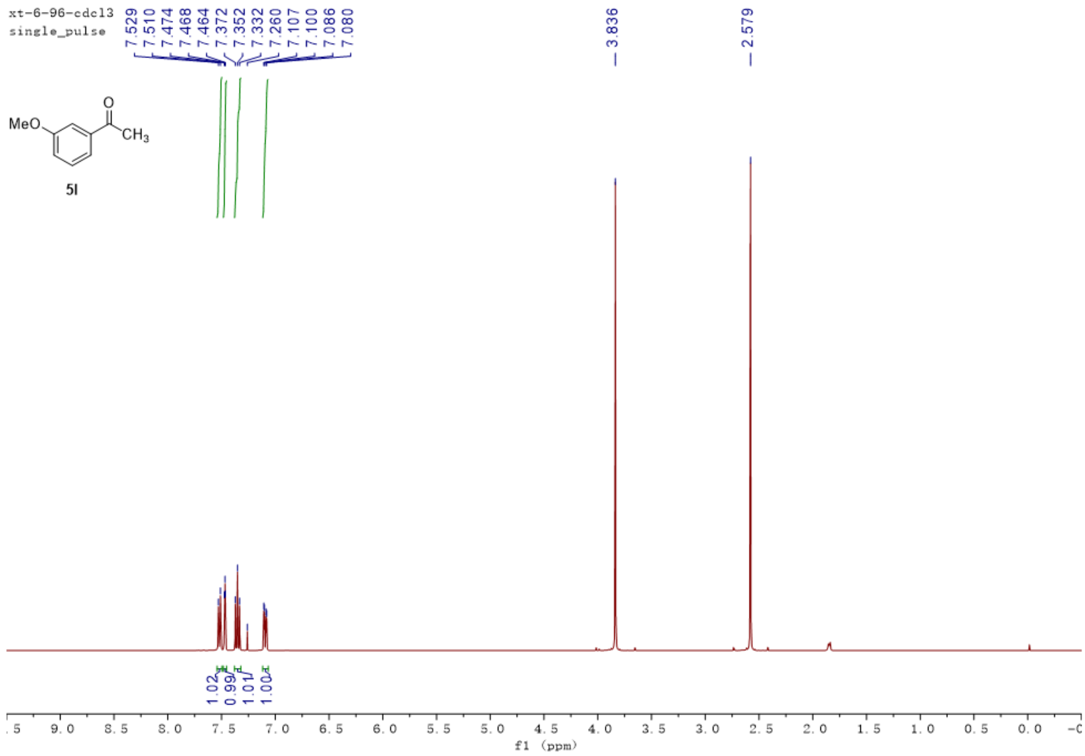

^{1}H NMR (400 MHz, $CDCl_3$) of **5l**

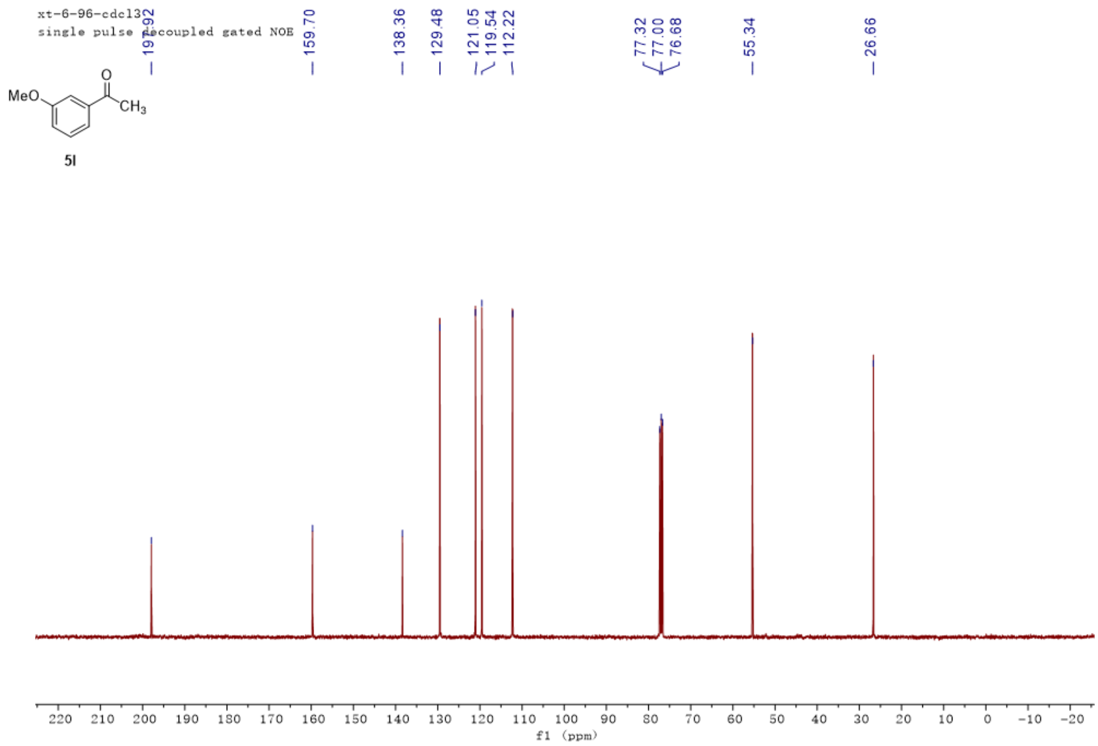

^{13}C NMR (101 MHz, $CDCl_3$) of **5l**

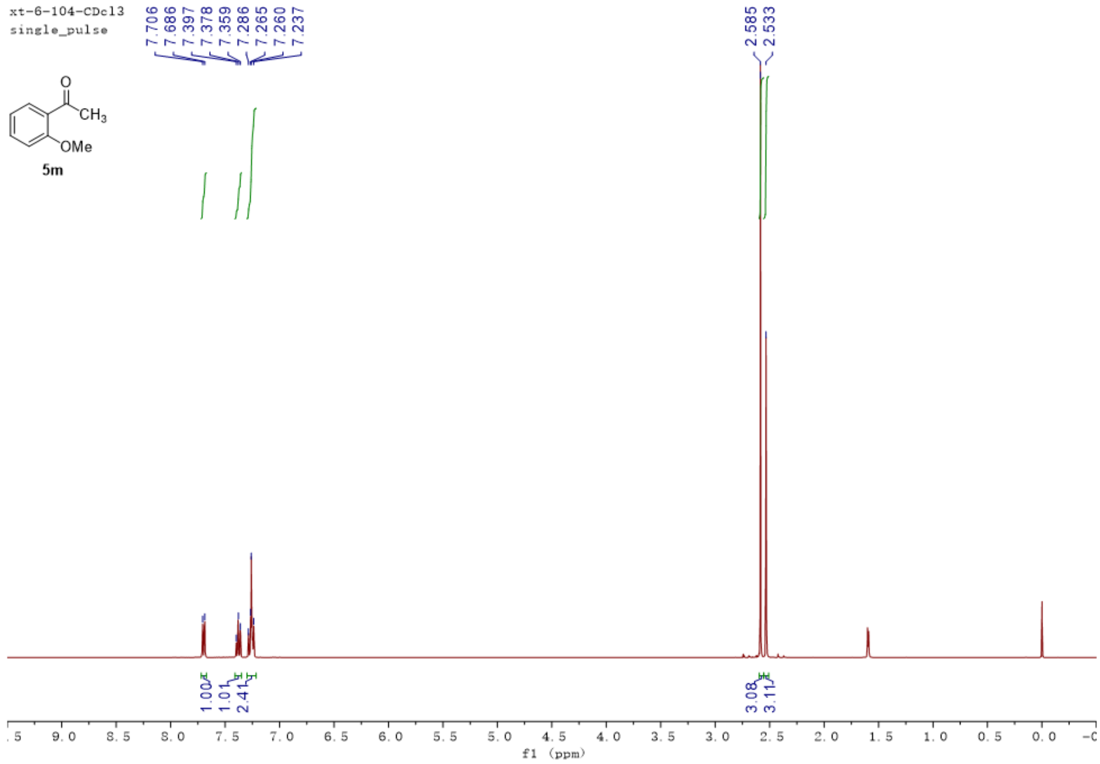

^{1}H NMR (400 MHz, $CDCl_3$) of **5m**

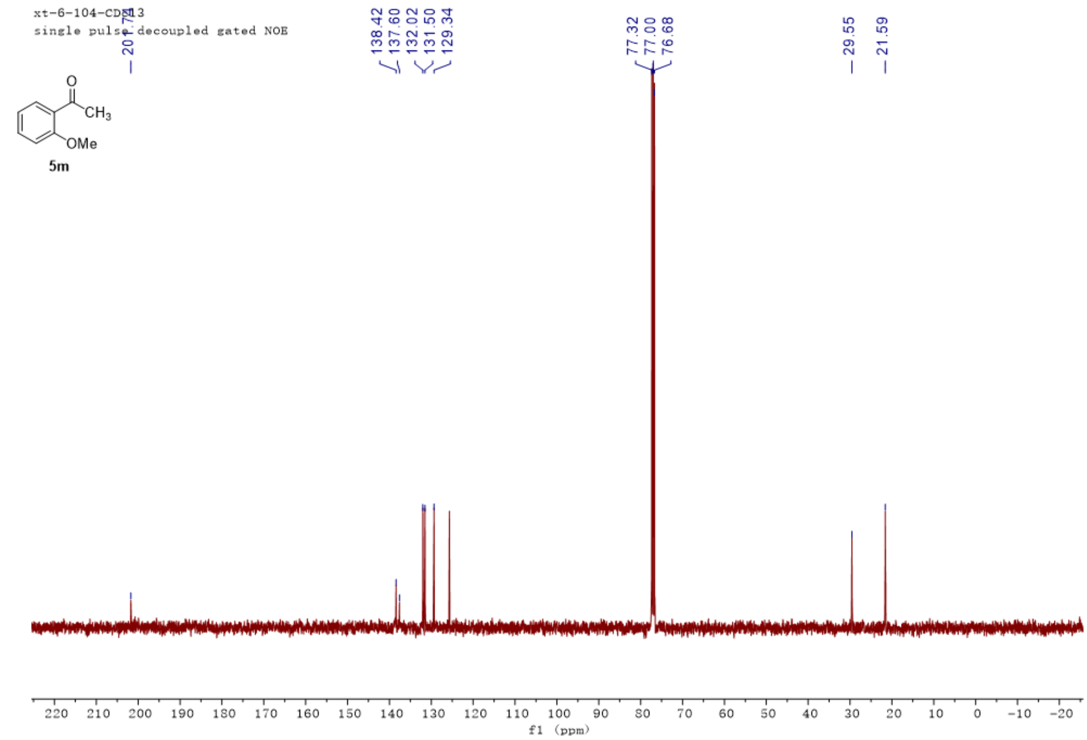

^{13}C NMR (101 MHz, $CDCl_3$) of **5m**

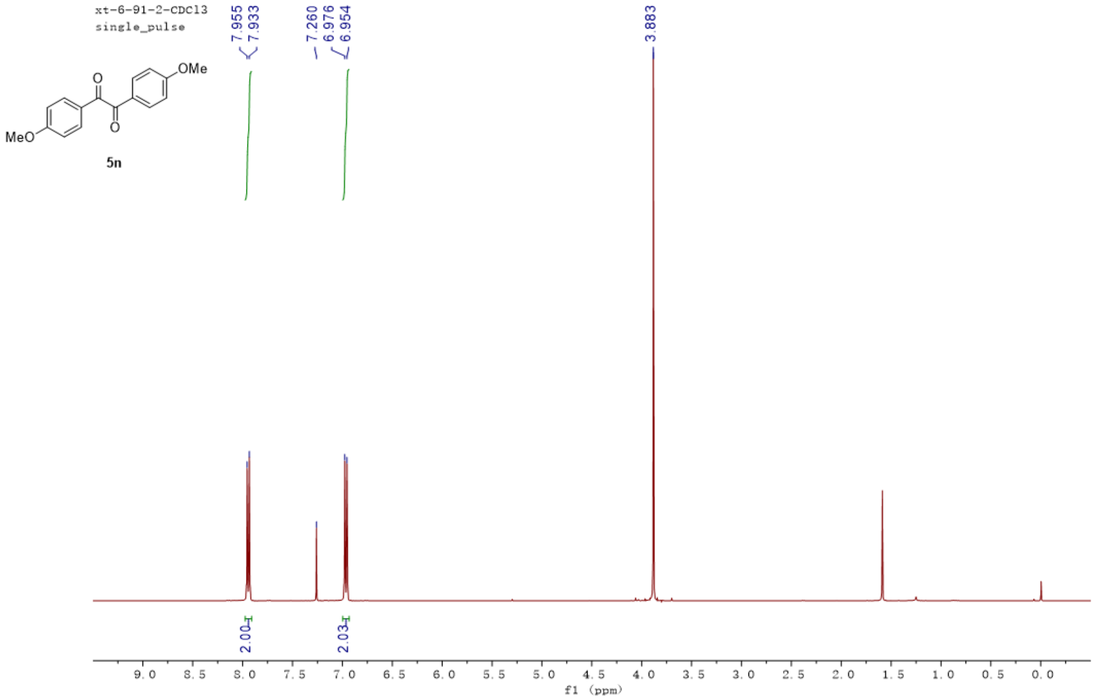

^{1}H NMR (400 MHz, $CDCl_3$) of **5n**

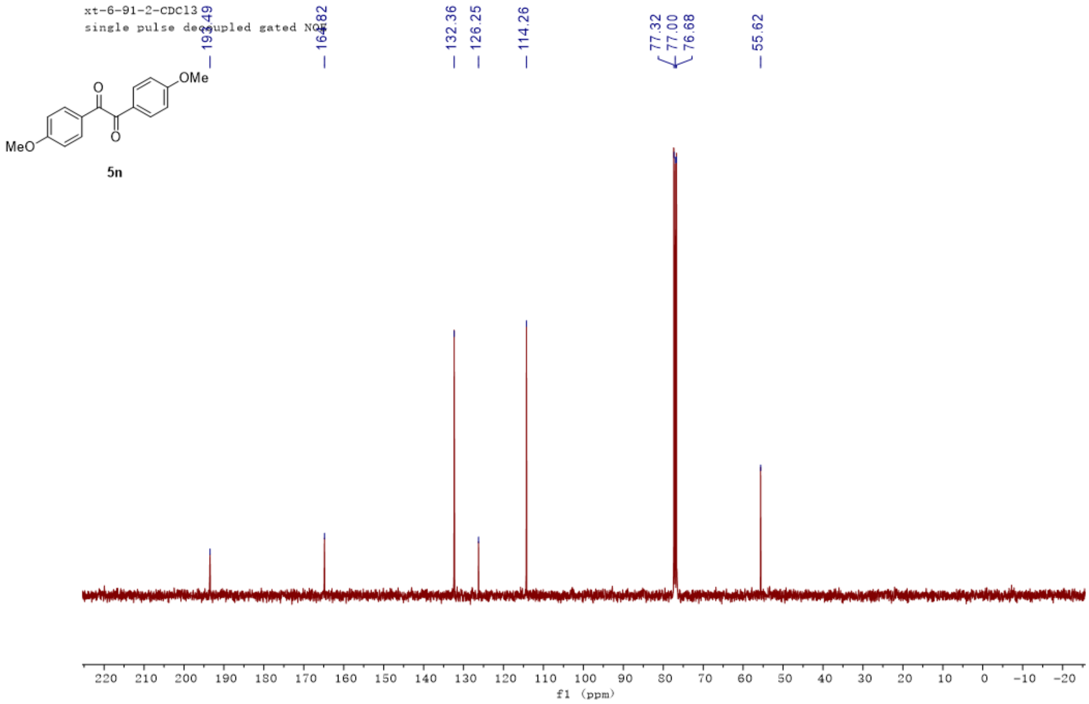

^{13}C NMR (101 MHz, $CDCl_3$) of **5n**

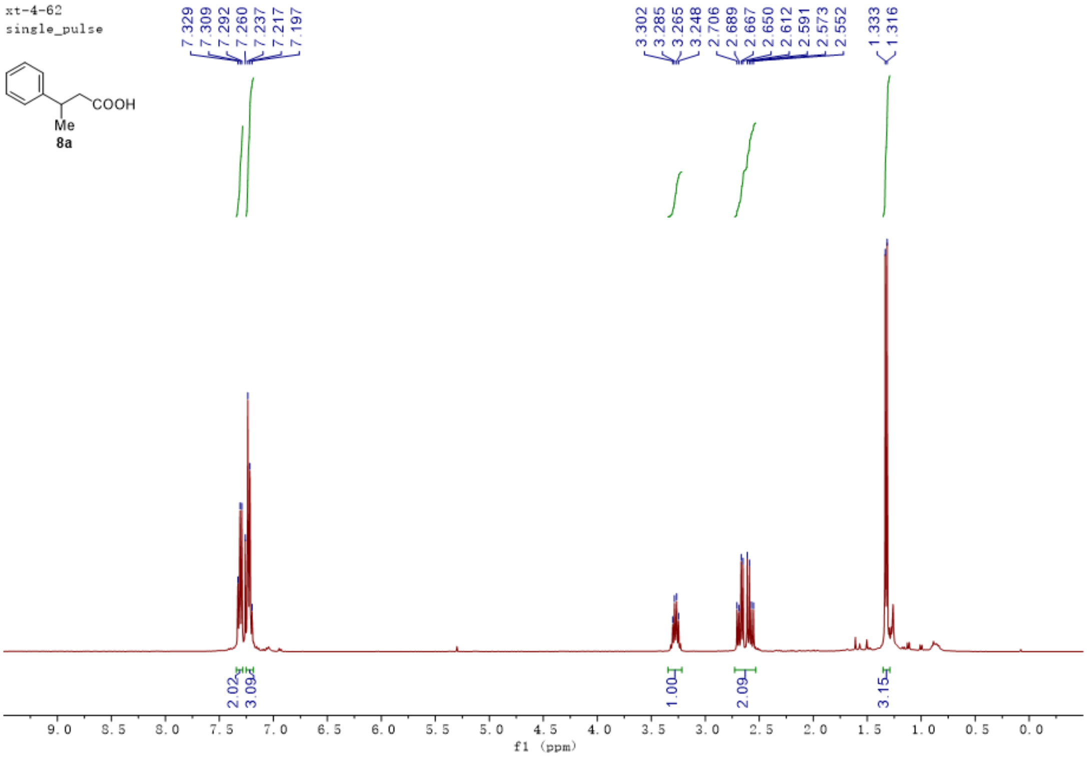

^{1}H NMR (400 MHz, $CDCl_3$) of **8a**

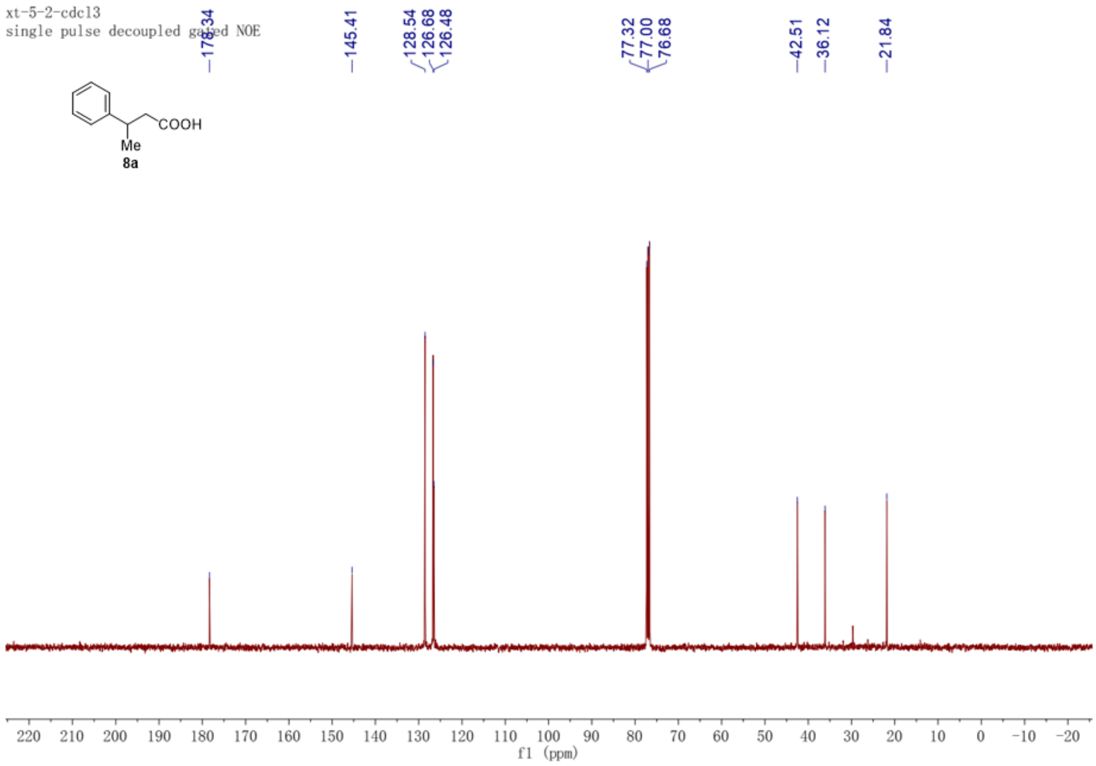

^{13}C NMR (101 MHz, $CDCl_3$) of **8a**

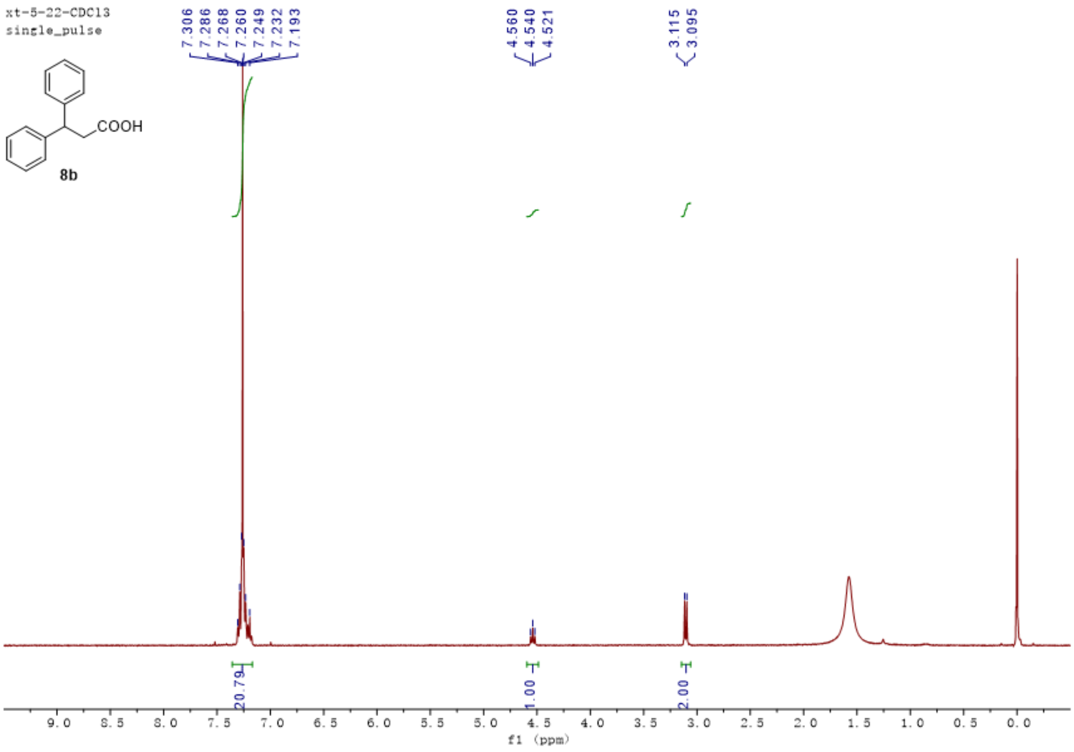

^{1}H NMR (400 MHz, $CDCl_3$) of **8b**

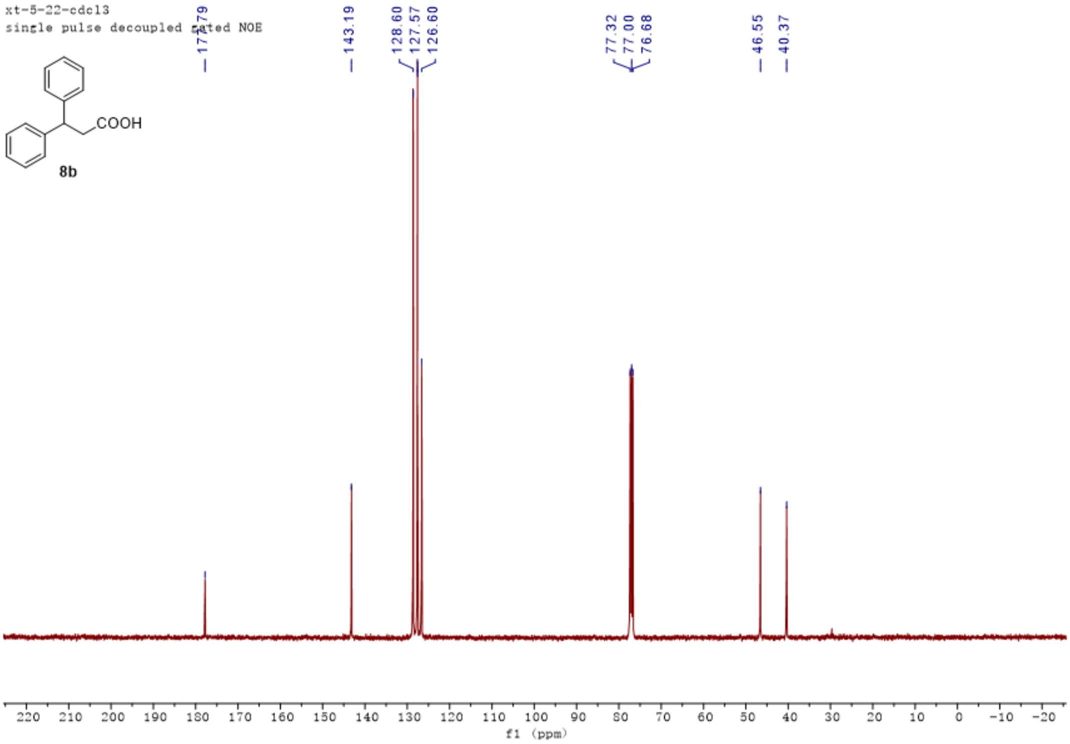

^{13}C NMR (101 MHz, $CDCl_3$) of **8b**

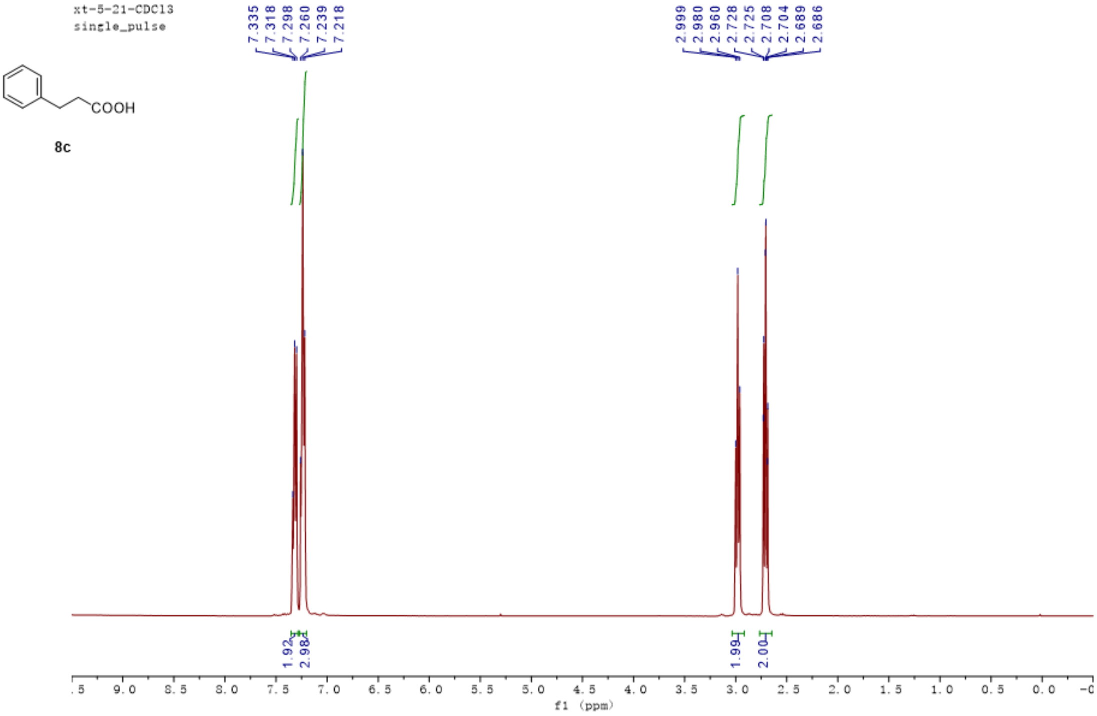

^{1}H NMR (400 MHz, $CDCl_3$) of **8c**

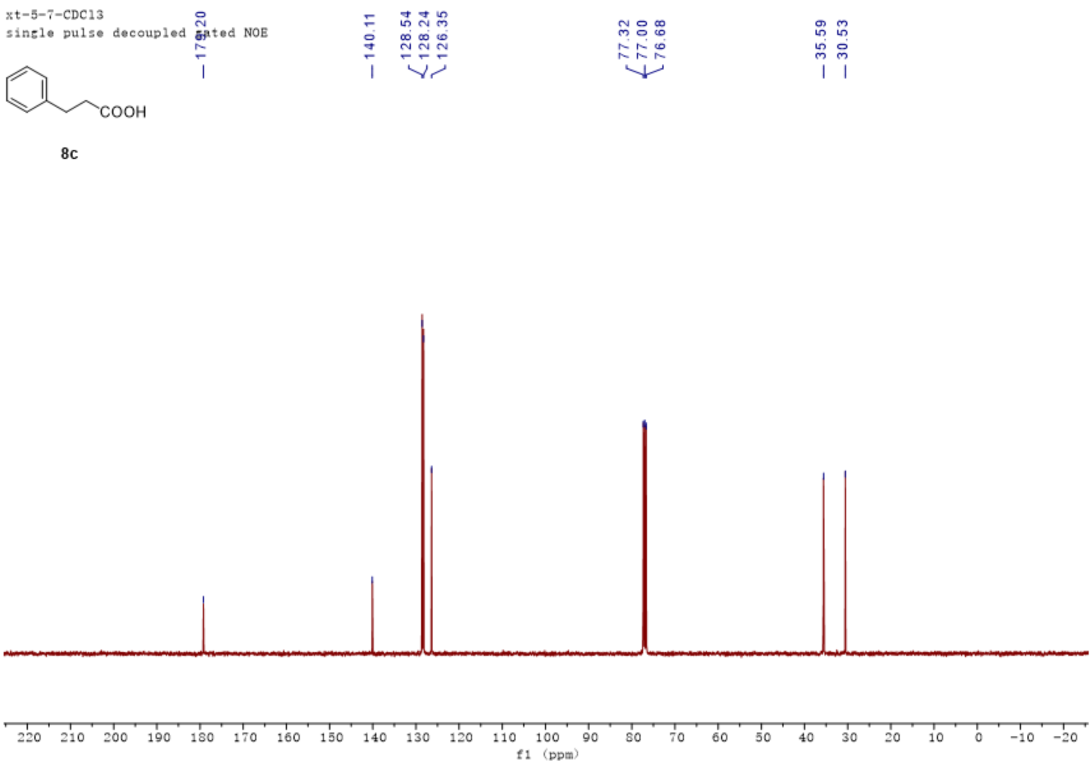

^{13}C NMR (101 MHz, $CDCl_3$) of **8c**

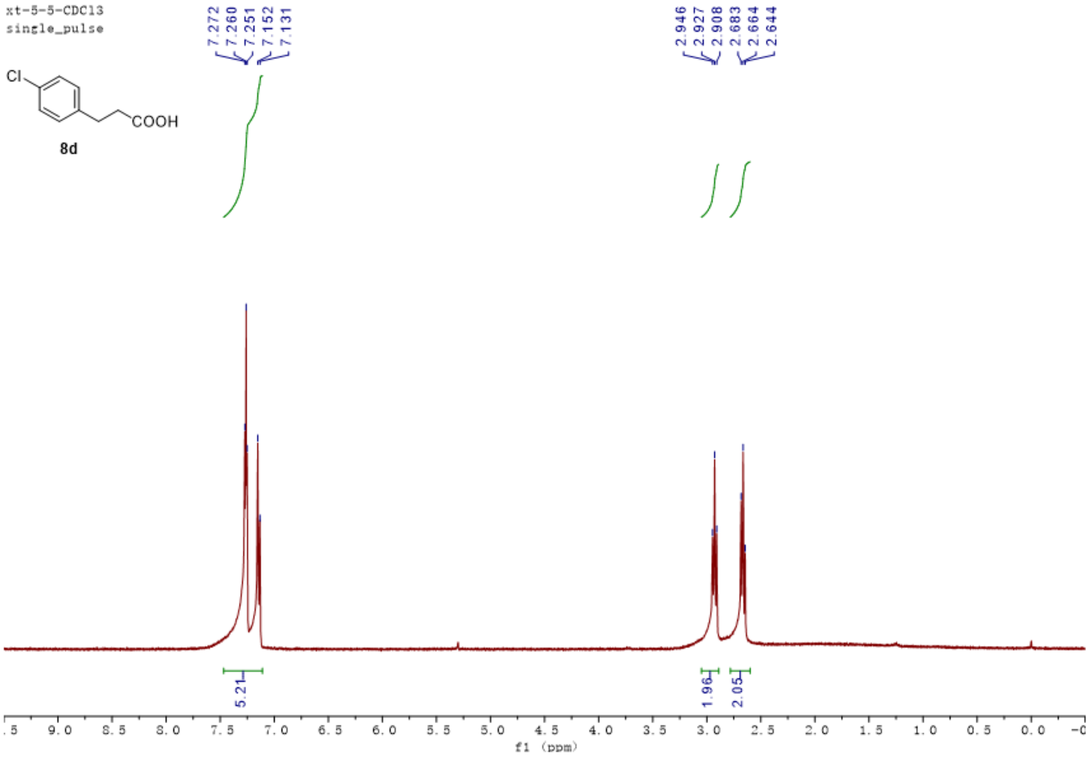

^{1}H NMR (400 MHz, $CDCl_3$) of **8d**

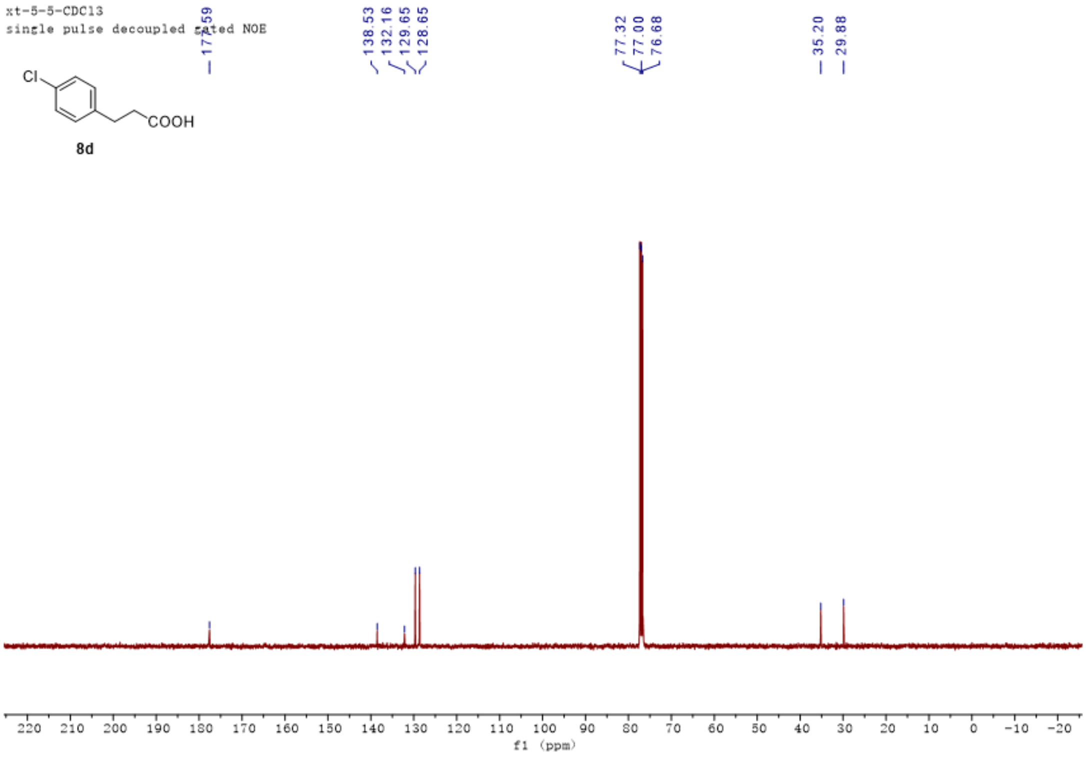

^{13}C NMR (101 MHz, $CDCl_3$) of **8d**

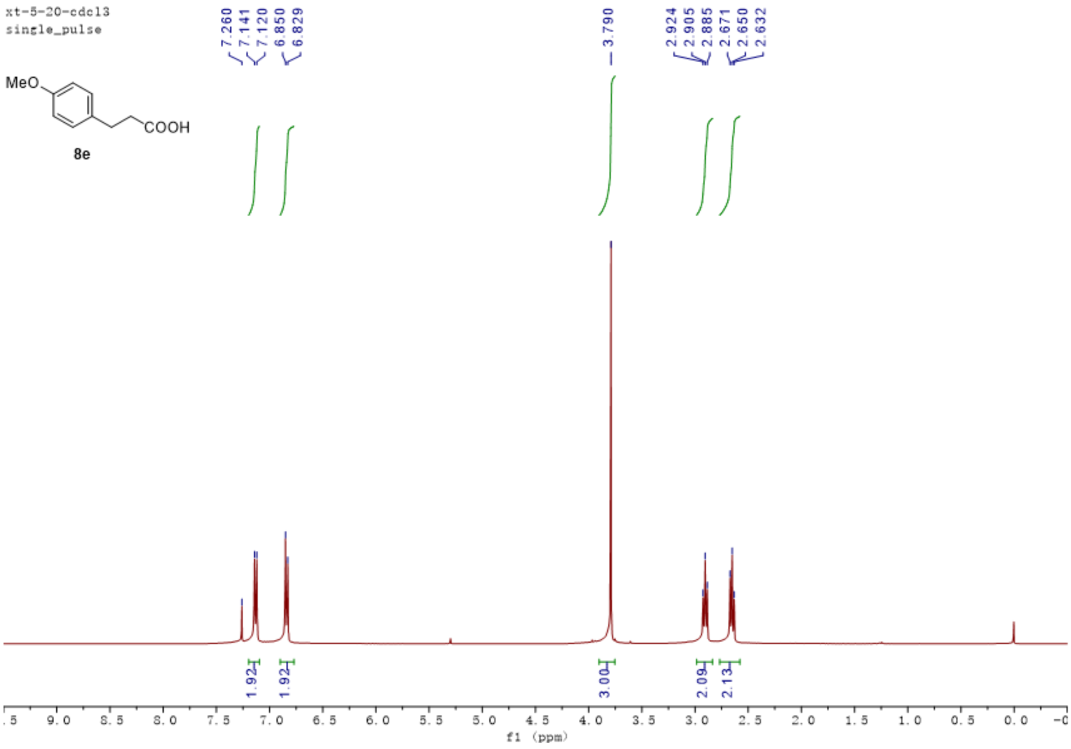

^{1}H NMR (400 MHz, $CDCl_3$) of **8e**

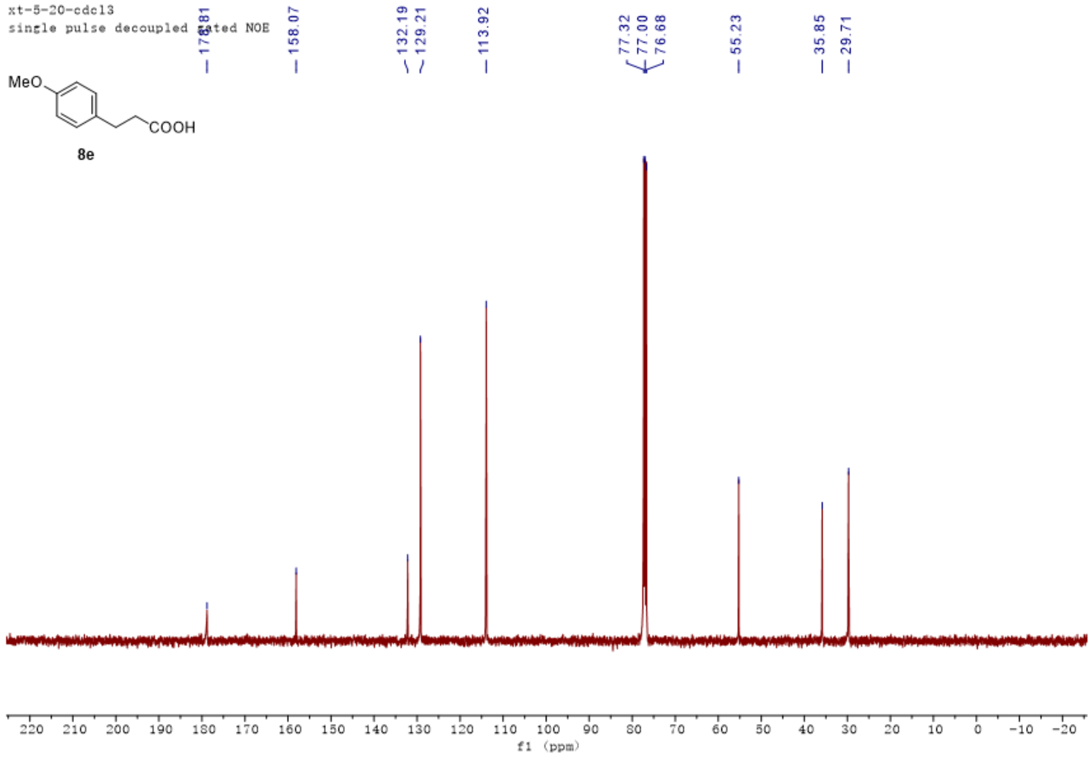

^{13}C NMR (101 MHz, $CDCl_3$) of **8e**

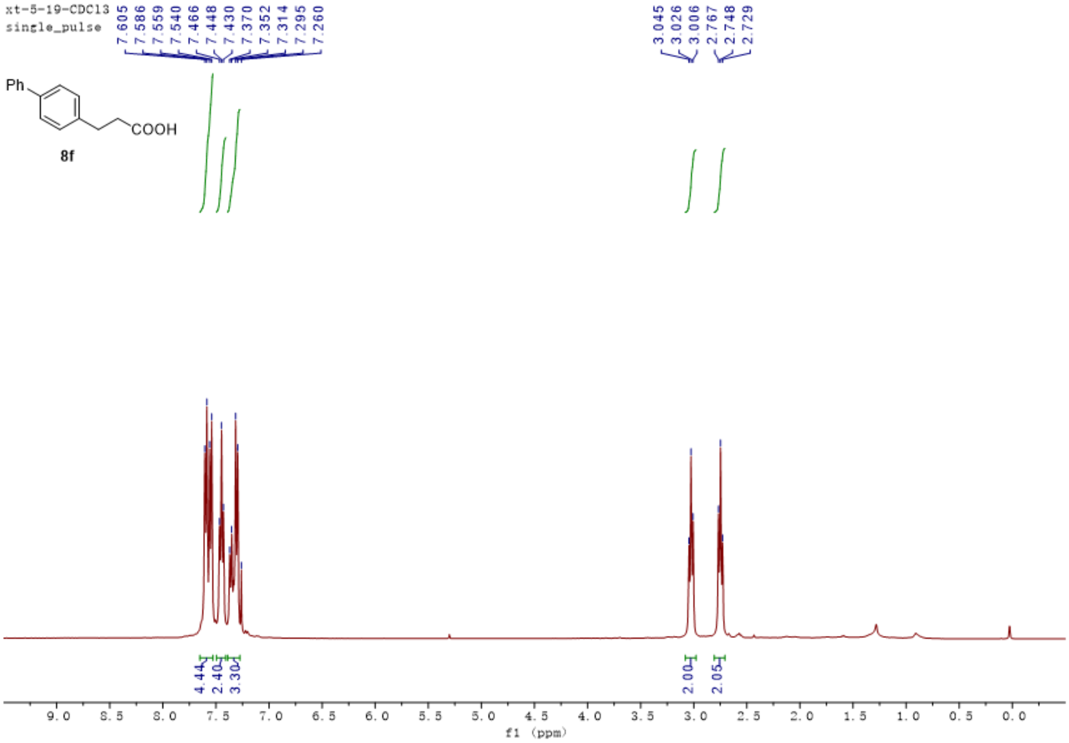

^{1}H NMR (400 MHz, $CDCl_3$) of **8f**

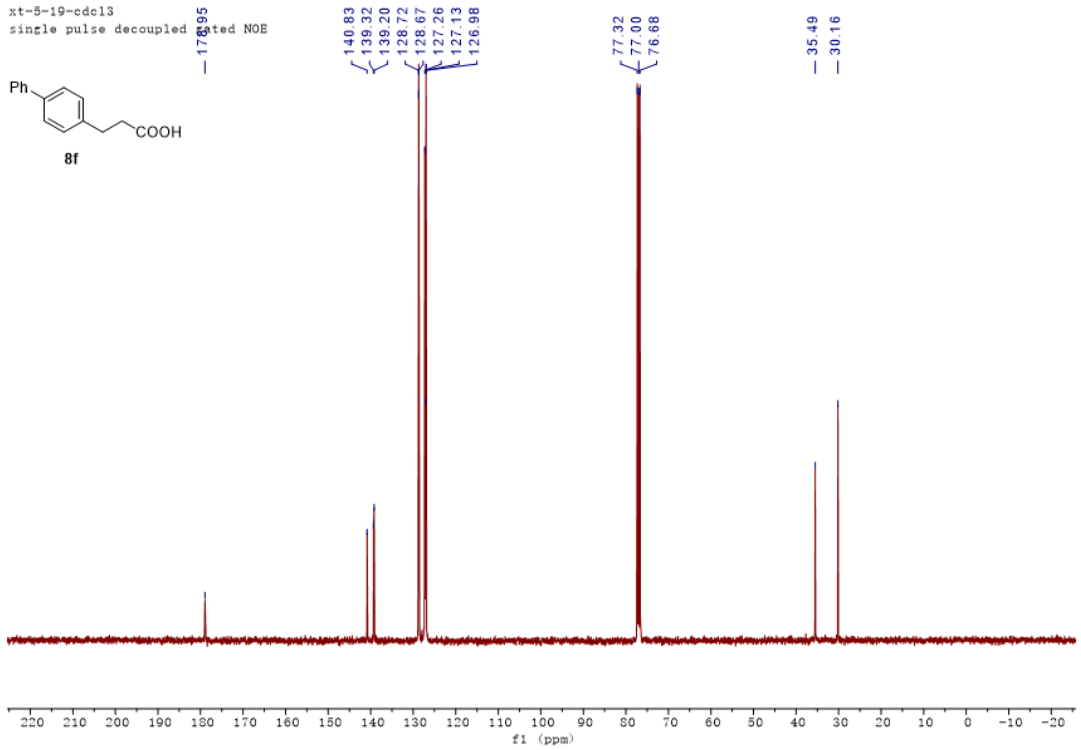

^{13}C NMR (101 MHz, $CDCl_3$) of **8f**

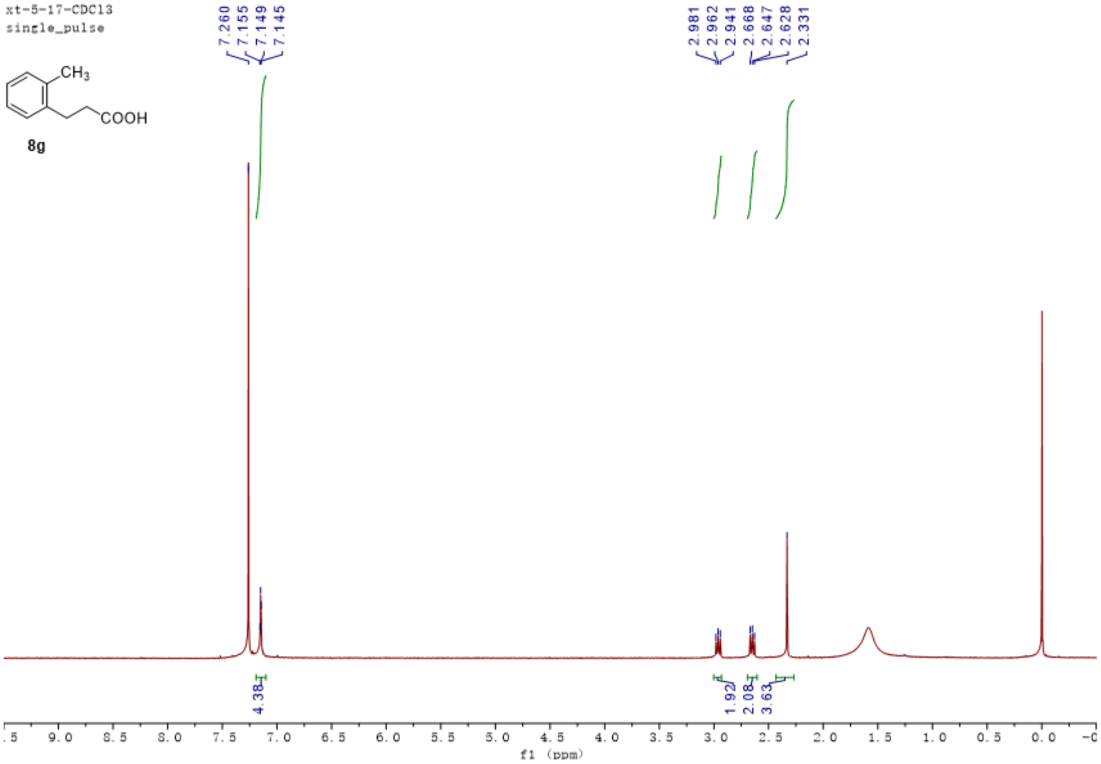

^{1}H NMR (400 MHz, $CDCl_3$) of **8g**

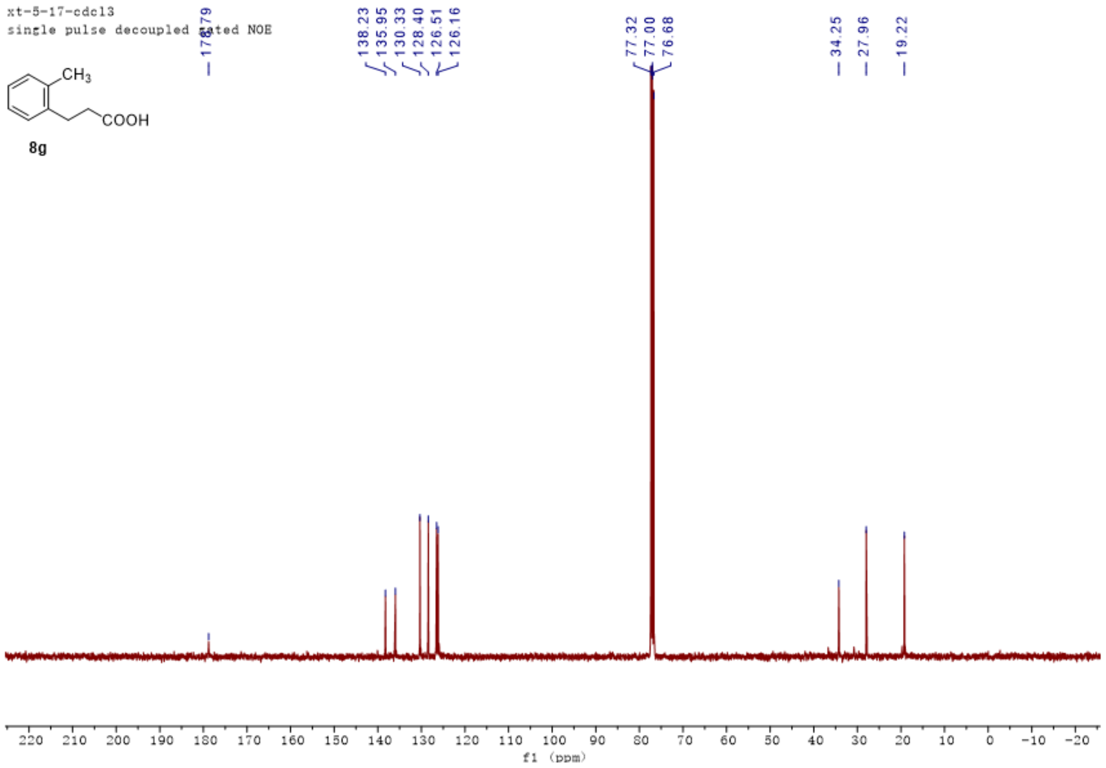

^{13}C NMR (101 MHz, $CDCl_3$) of **8g**

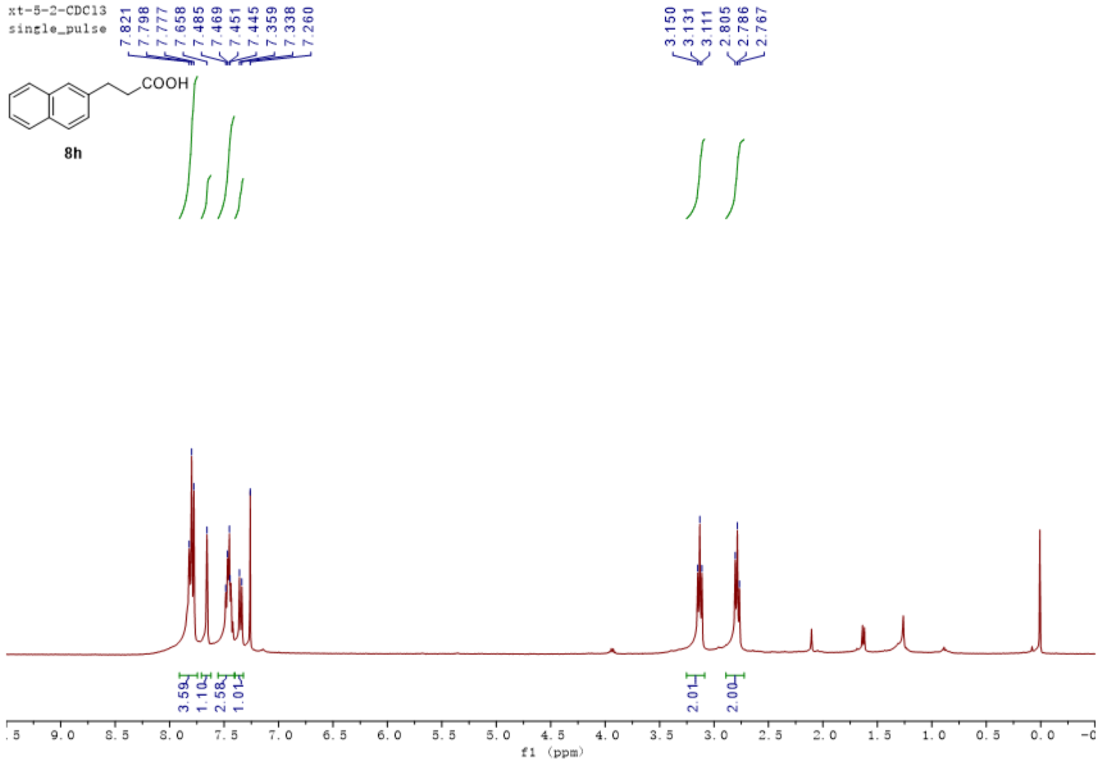

^{1}H NMR (400 MHz, $CDCl_3$) of **8h**

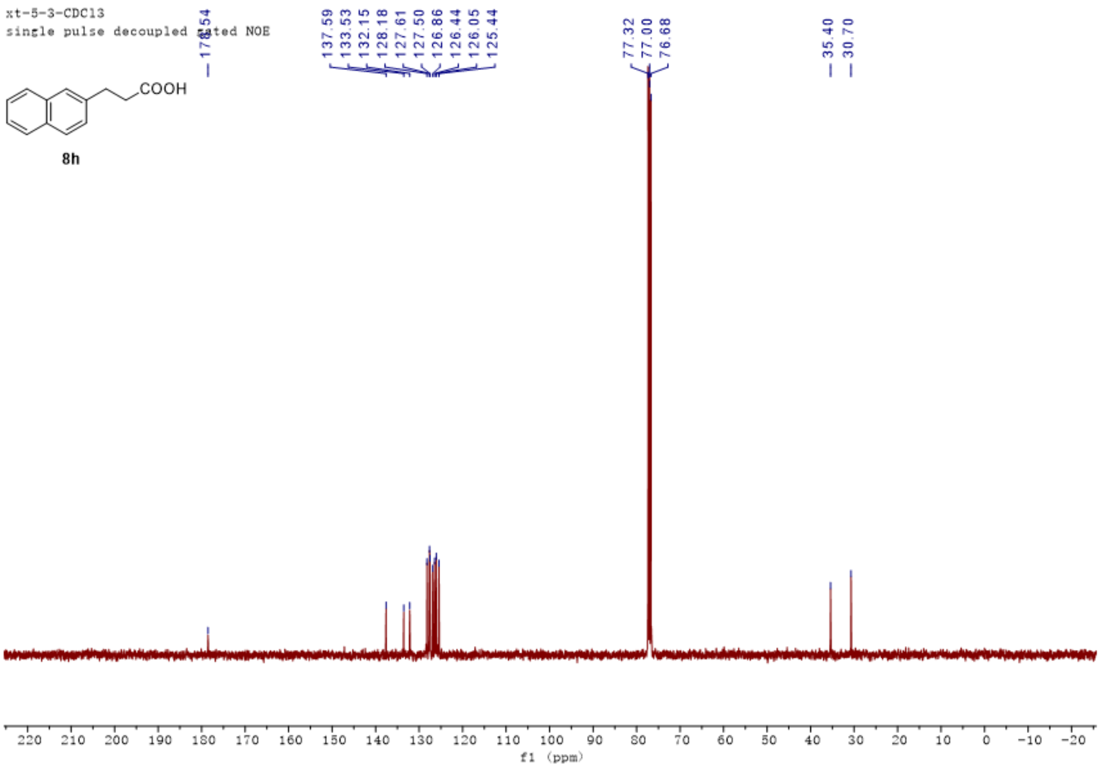

^{13}C NMR (101 MHz, $CDCl_3$) of **8h**

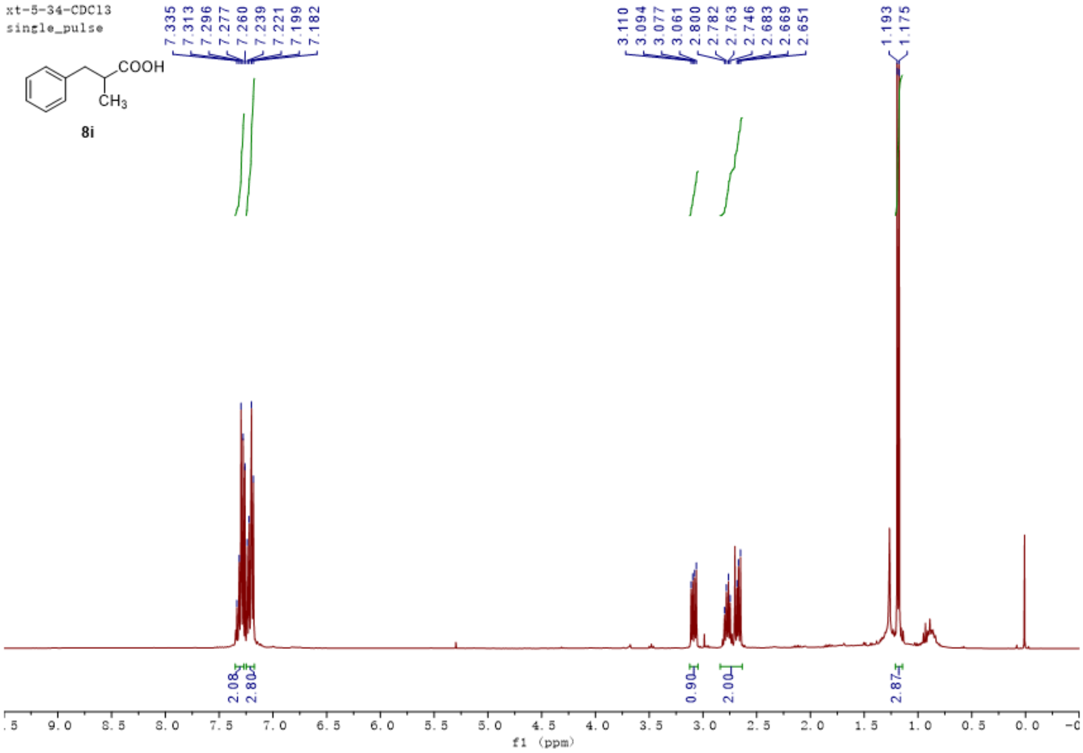

^{1}H NMR (400 MHz, $CDCl_3$) of **8i**

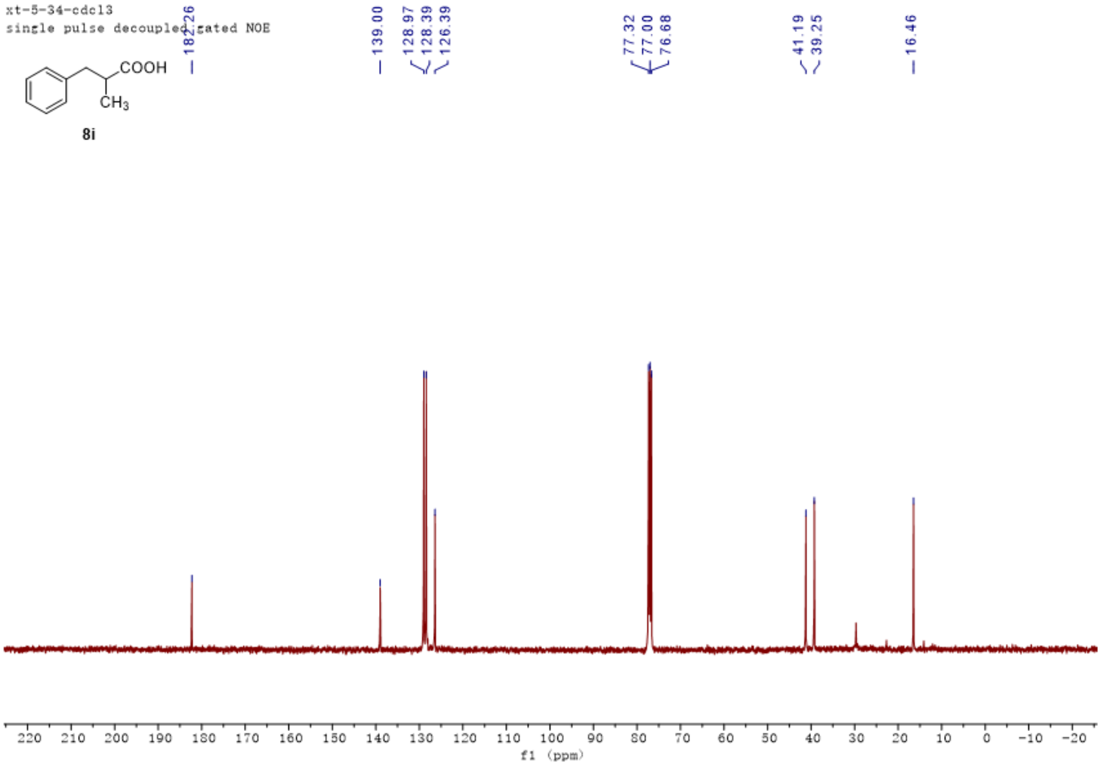

^{13}C NMR (101 MHz, $CDCl_3$) of **8i**

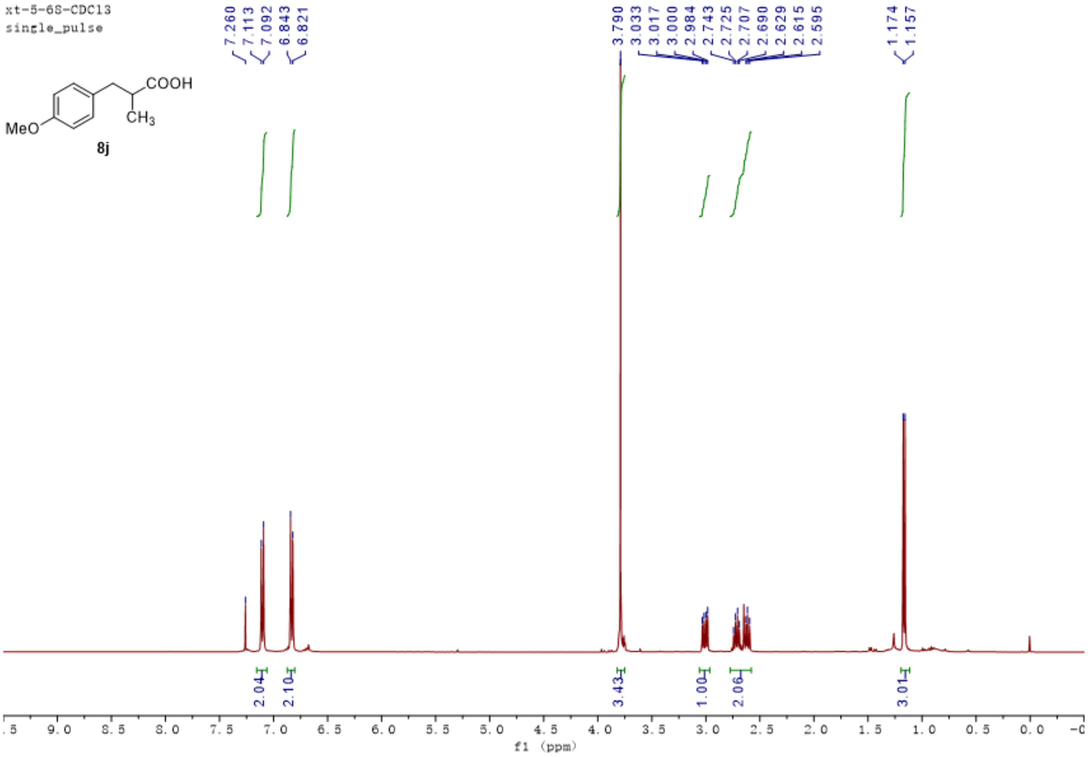

^{1}H NMR (400 MHz, $CDCl_3$) of **8j**

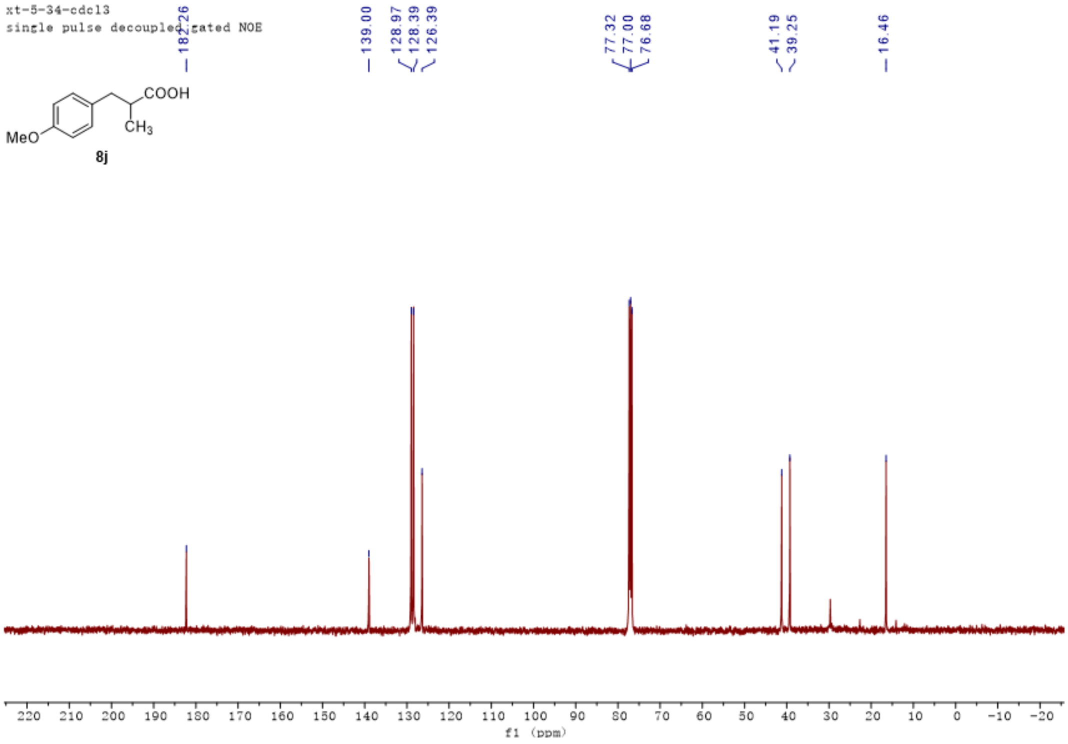

^{13}C NMR (101 MHz, $CDCl_3$) of **8j**

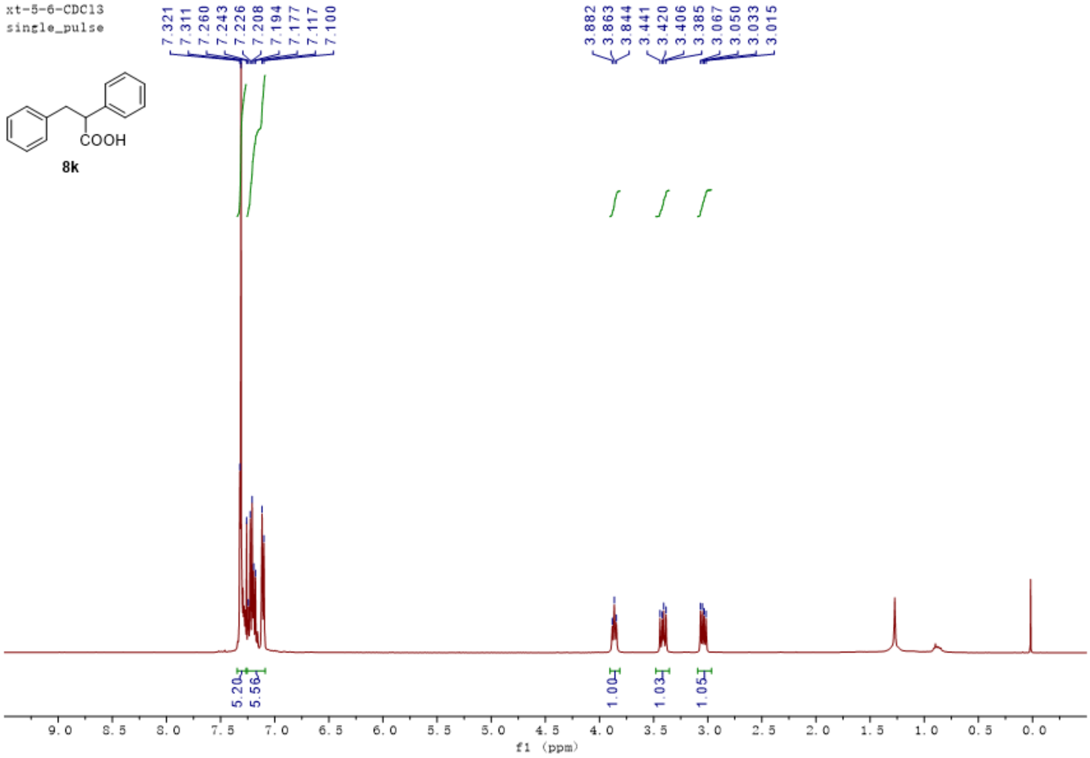

^{1}H NMR (400 MHz, $CDCl_3$) of **8k**

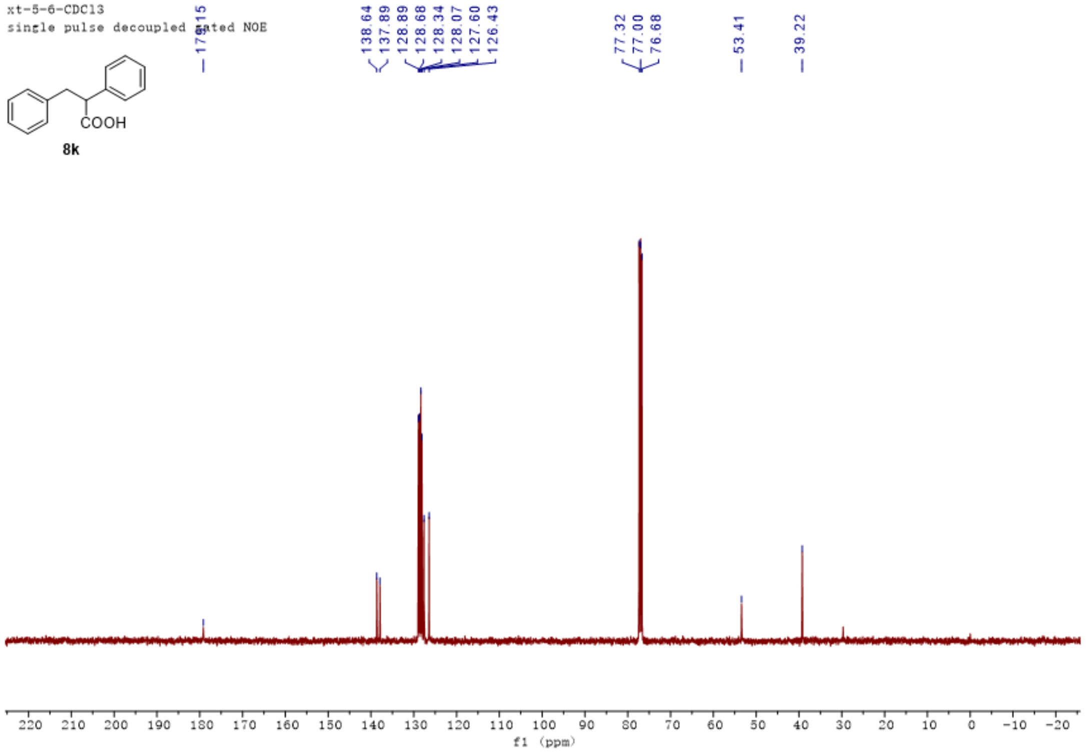

^{13}C NMR (101 MHz, $CDCl_3$) of **8k**

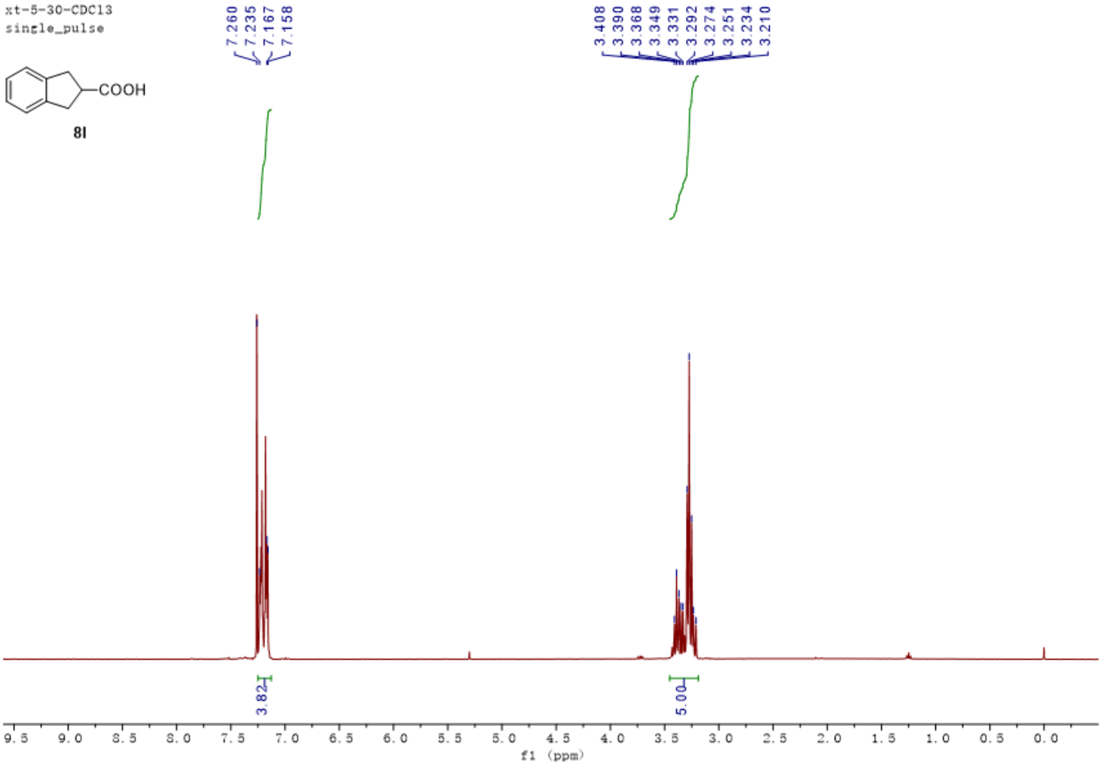

^{1}H NMR (400 MHz, $CDCl_3$) of **8l**

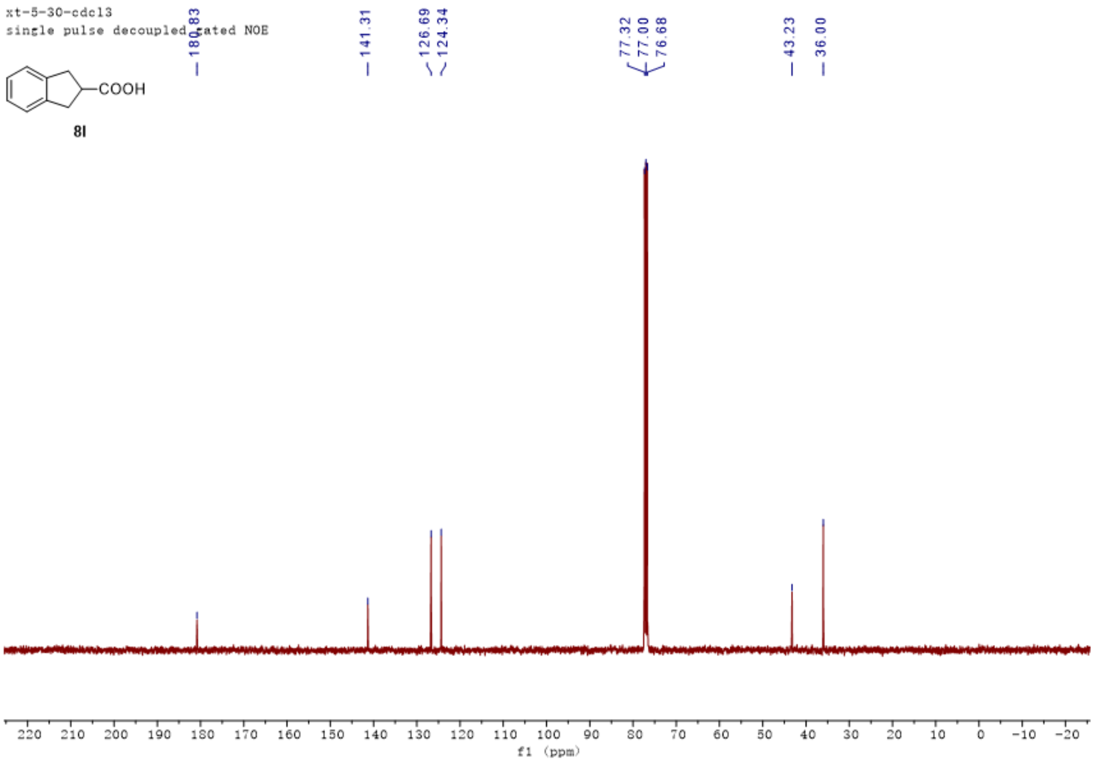

^{13}C NMR (101 MHz, $CDCl_3$) of **8l**

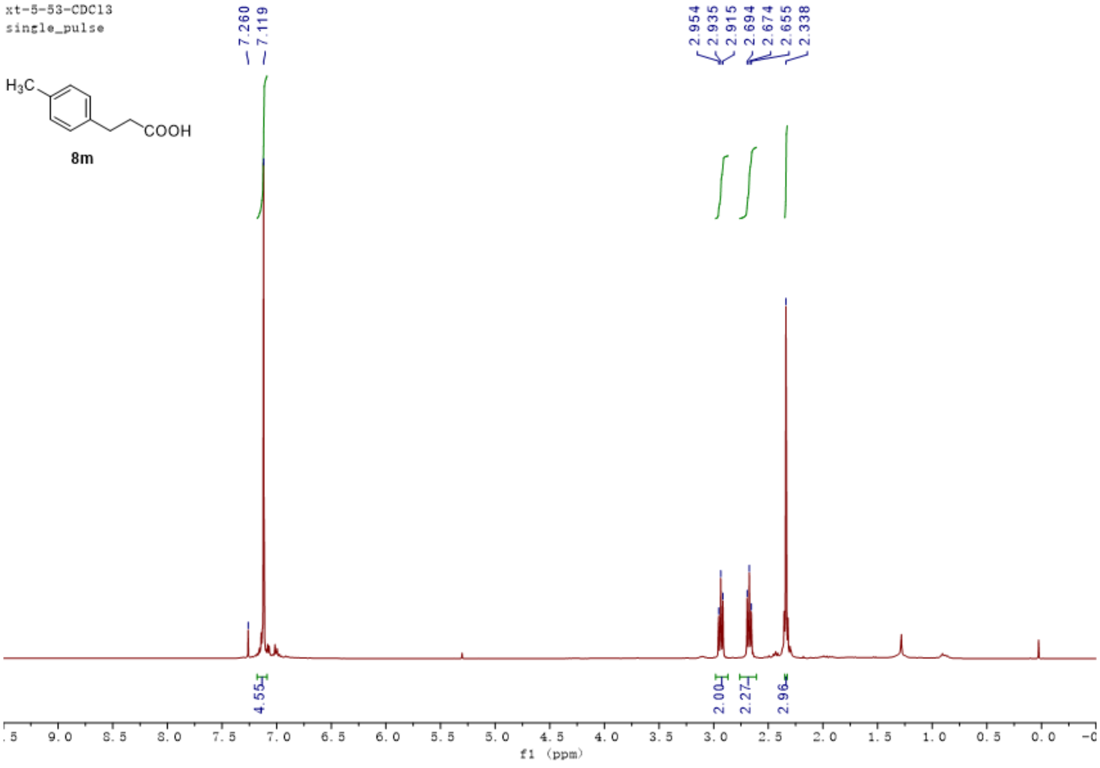

^{1}H NMR (400 MHz, $CDCl_3$) of **8m**

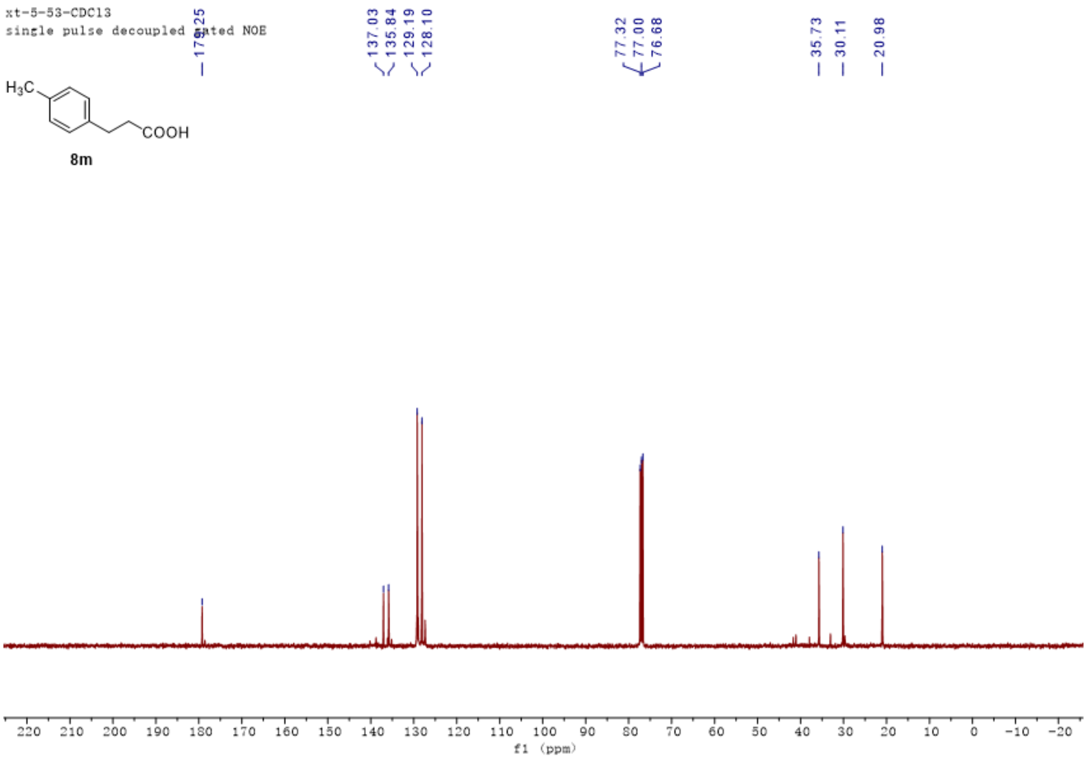

^{13}C NMR (101 MHz, $CDCl_3$) of **8m**

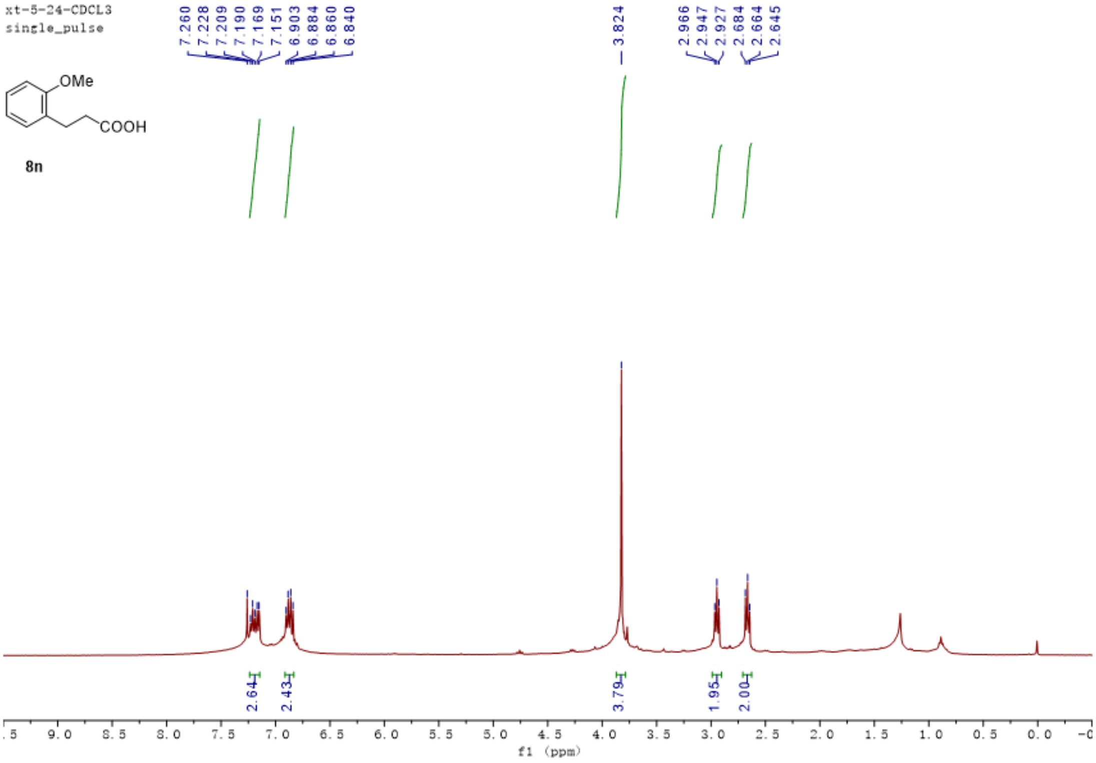

^{1}H NMR (400 MHz, $CDCl_3$) of **8n**

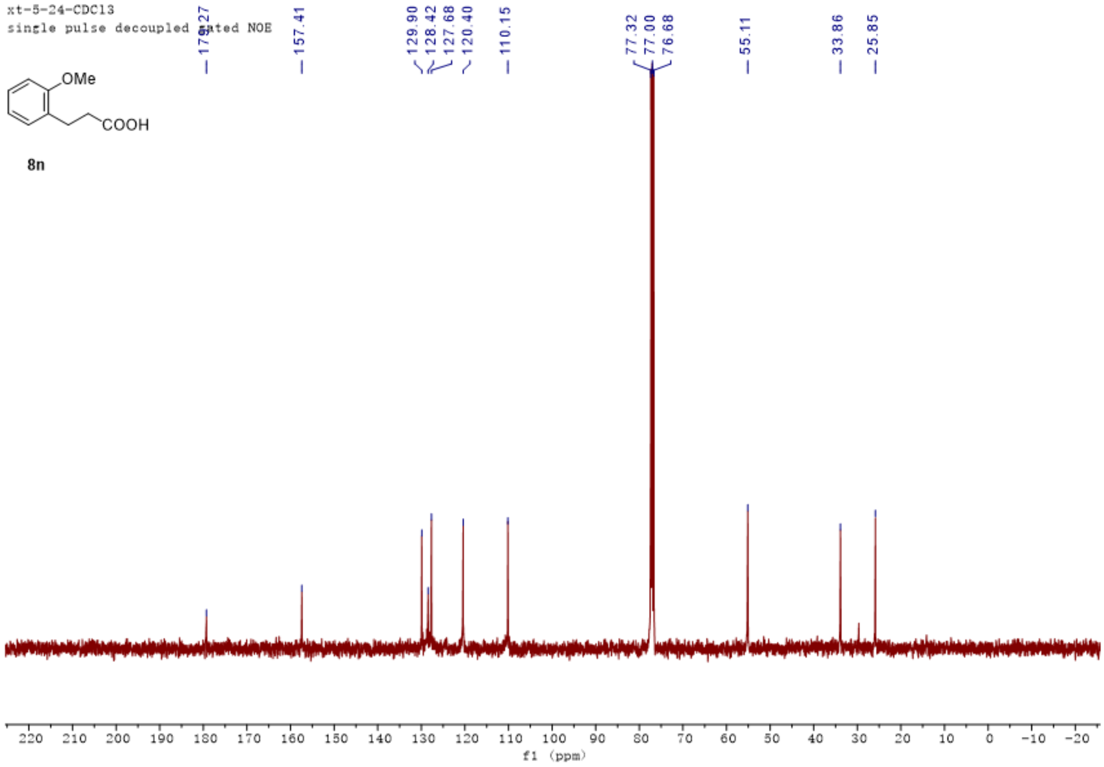

^{13}C NMR (101 MHz, $CDCl_3$) of **8n**

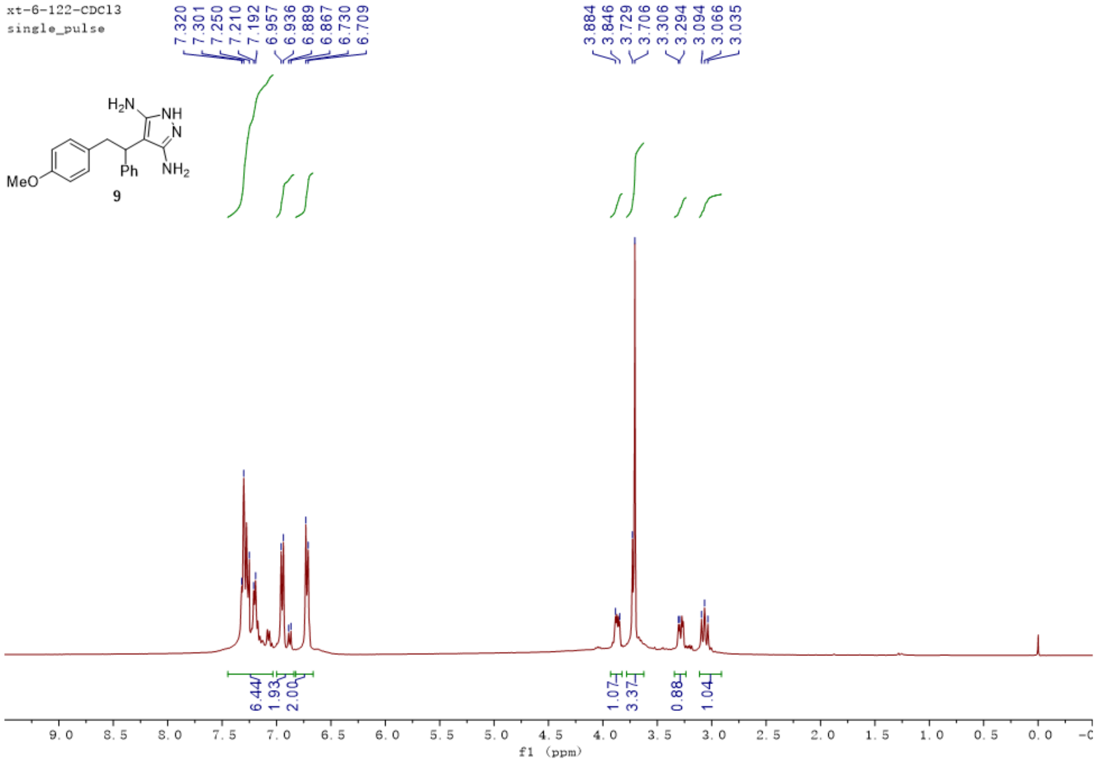

^{1}H NMR (400 MHz, $CDCl_3$) of **9**

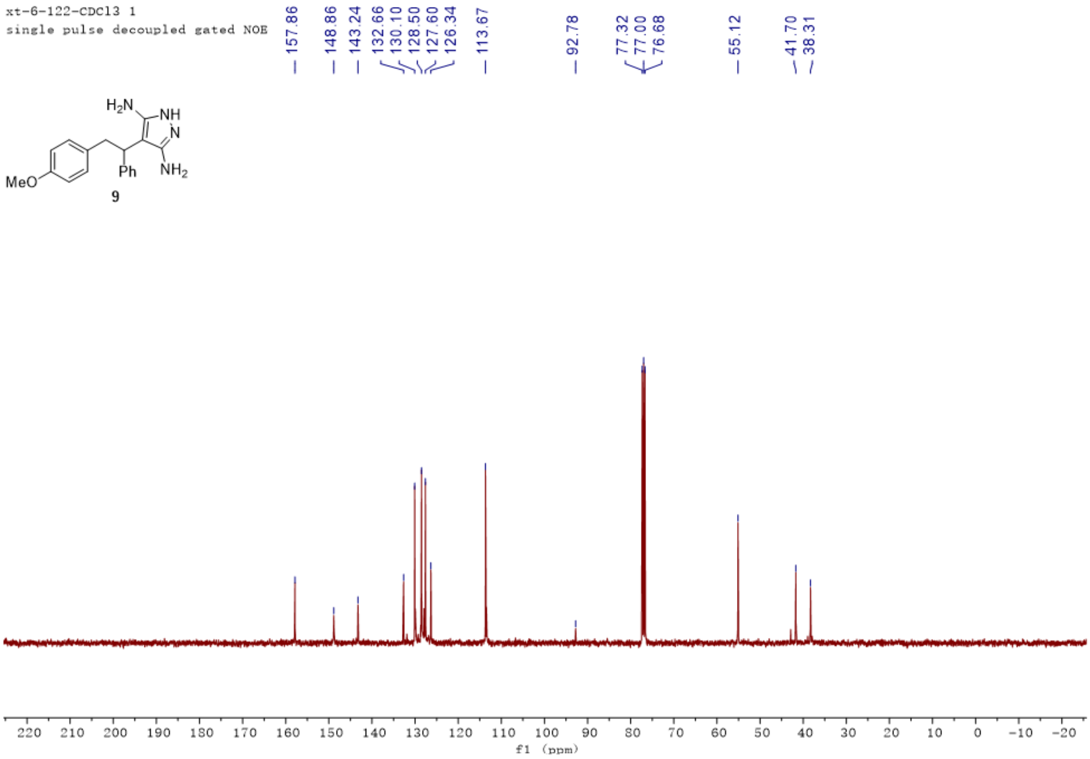

^{13}C NMR (101 MHz, $CDCl_3$) of **9**

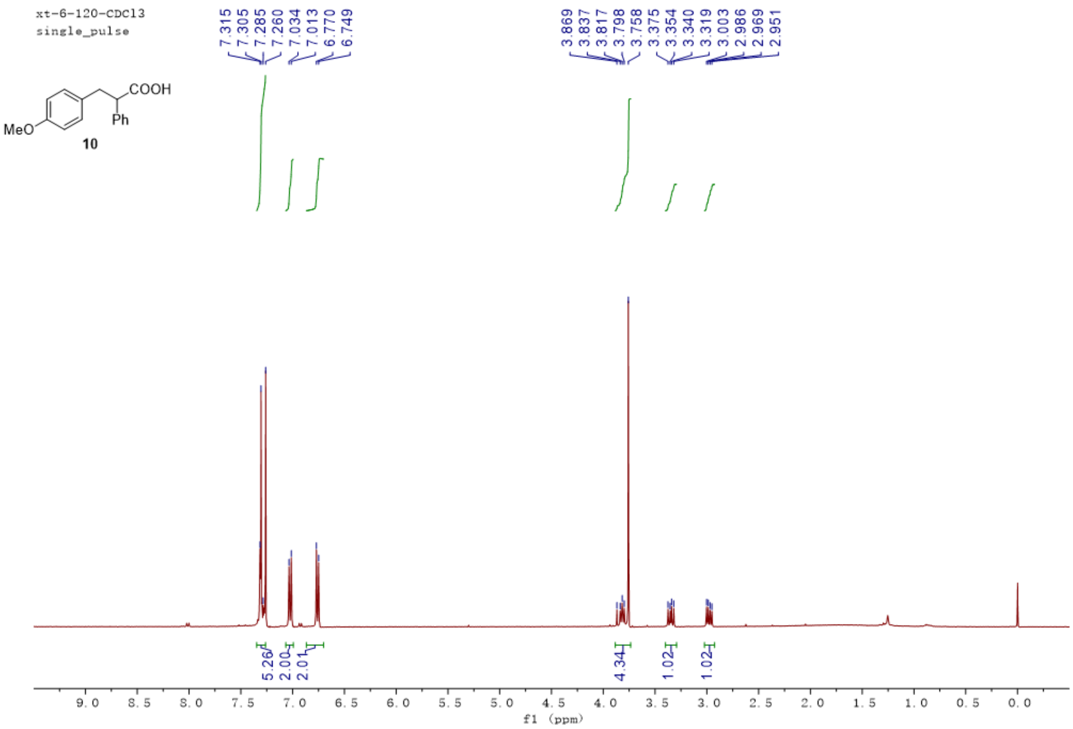

^{1}H NMR (400 MHz, $CDCl_3$) of **10**

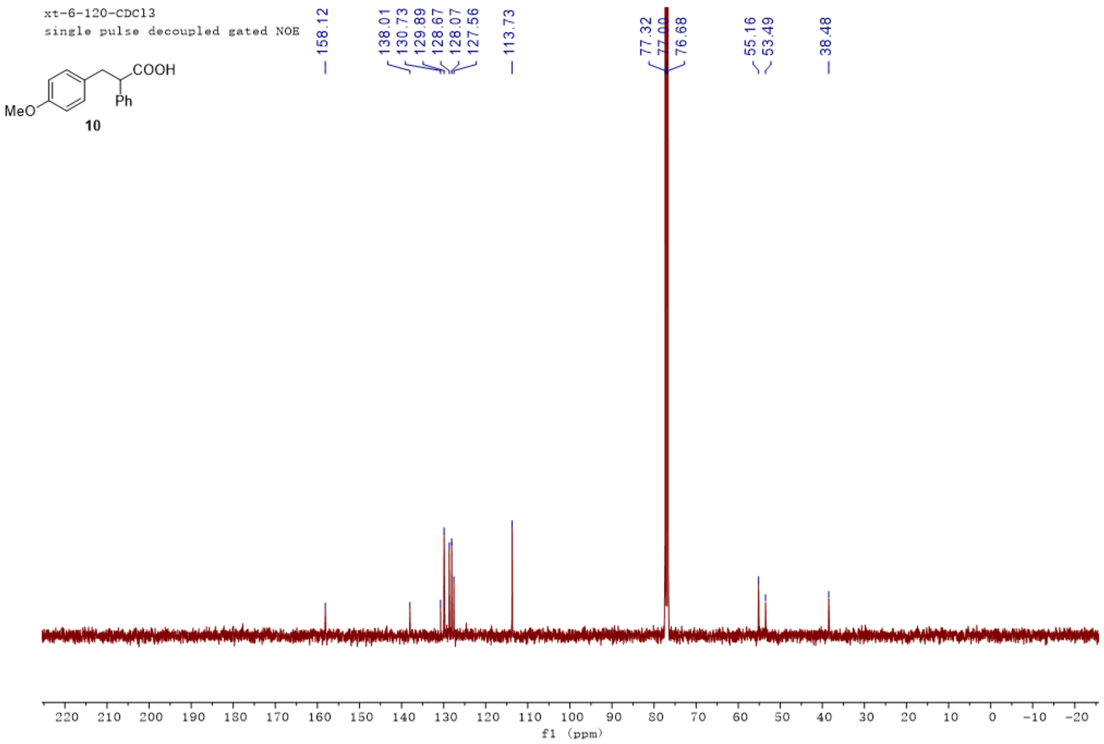

^{13}C NMR (101 MHz, $CDCl_3$) of **10**

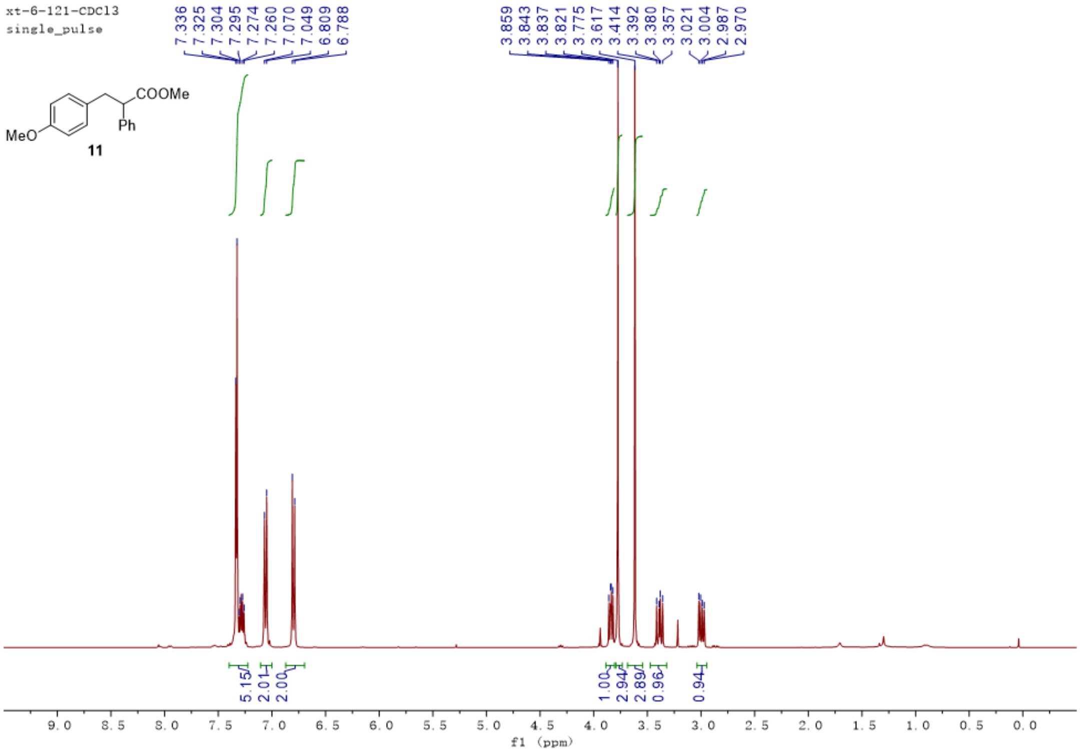

^{1}H NMR (400 MHz, $CDCl_3$) of **11**

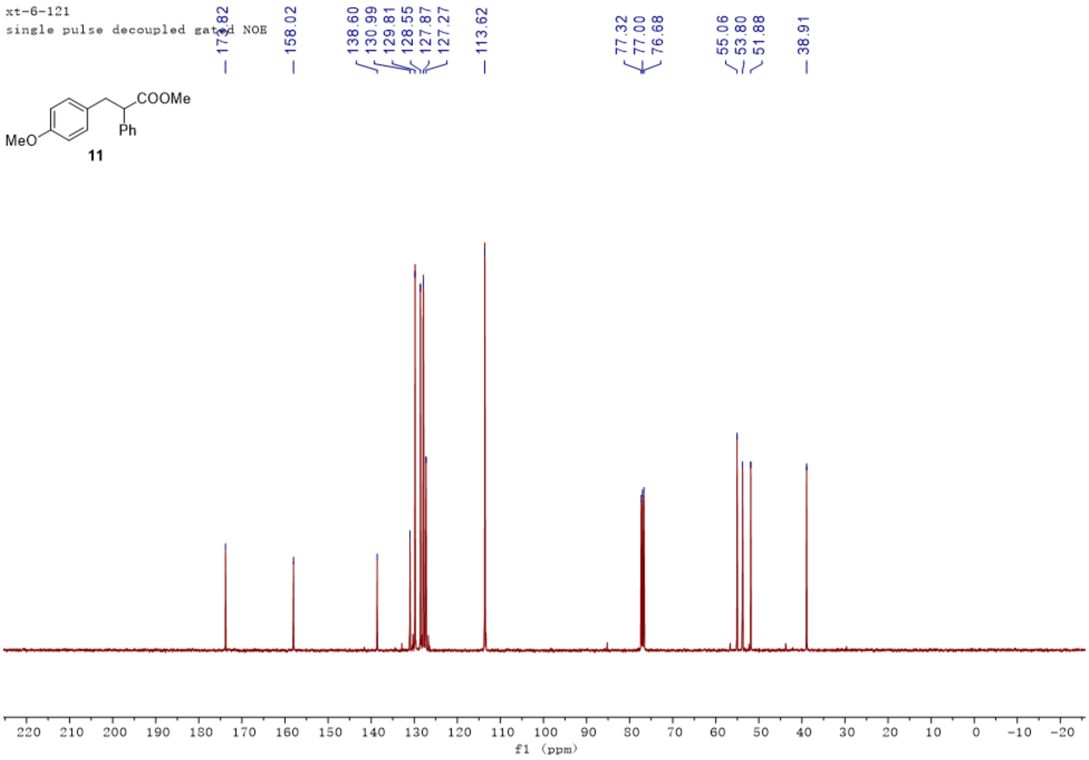

^{13}C NMR (101 MHz, $CDCl_3$) of **11**

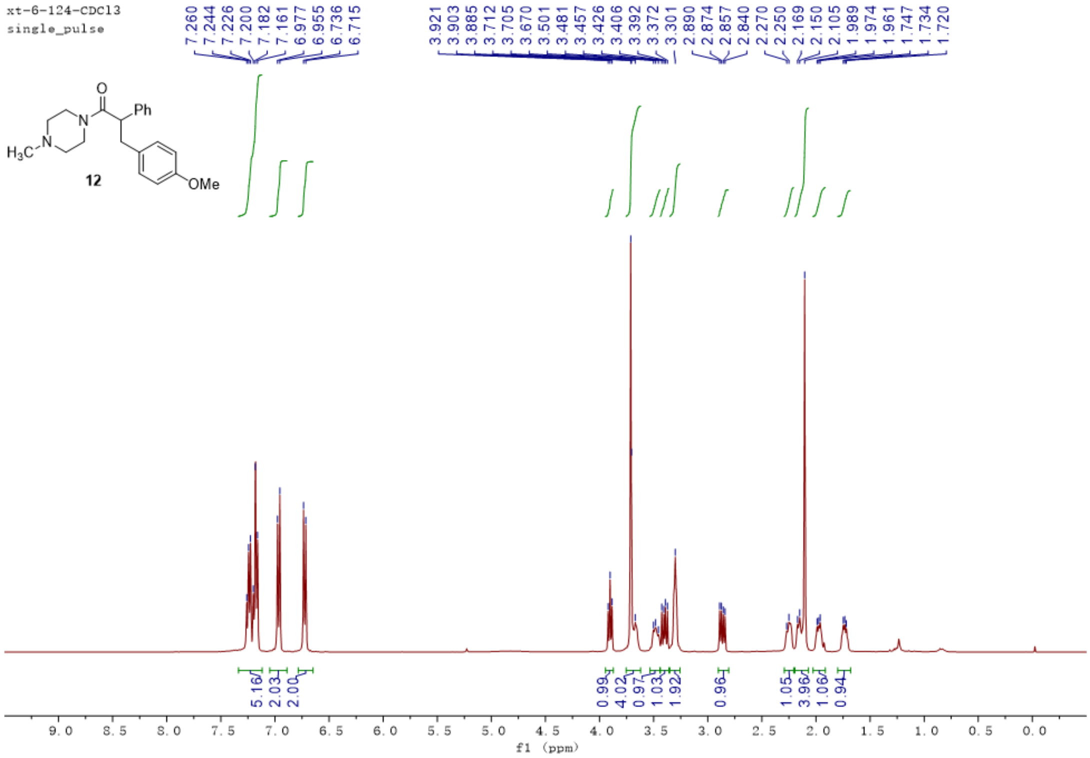

^{1}H NMR (400 MHz, $CDCl_3$) of **12**

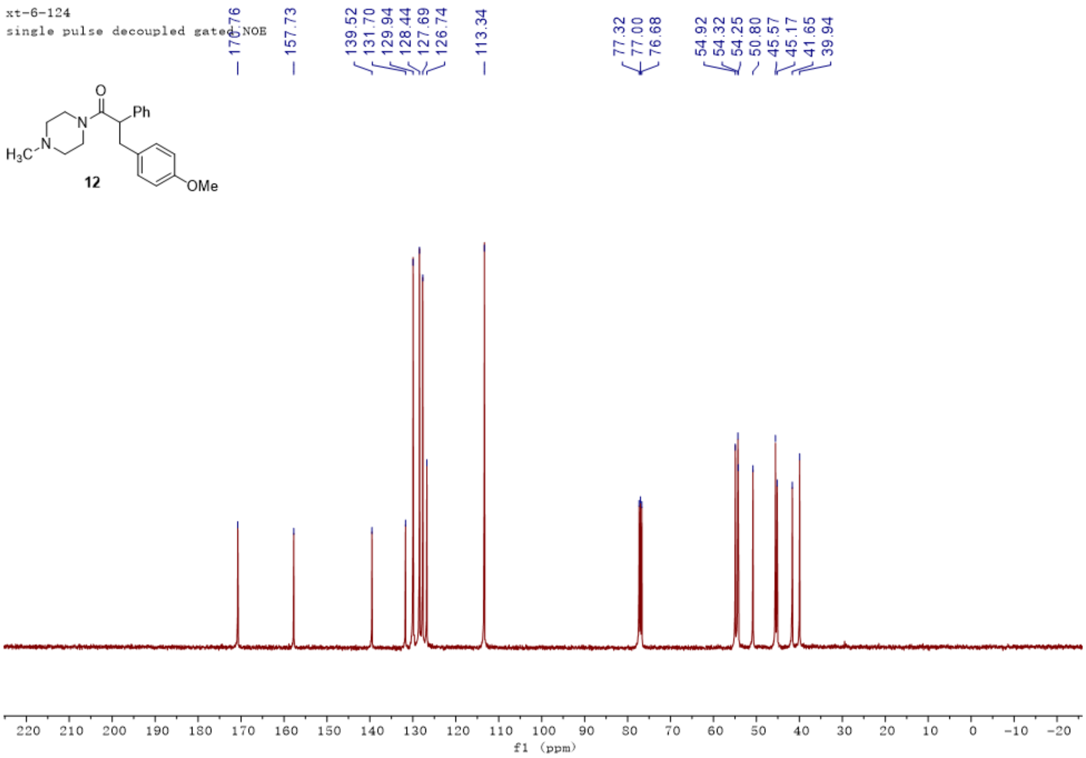

^{13}C NMR (101 MHz, $CDCl_3$) of **12**

References

(1) Meng, Q. Y., Lezius, L. & Studer, A. Benzylic C-H acylation by cooperative NHC and photoredox catalysis. *Nat. Commun.* **12**, 2068 (2021).

(2) Zuo, W. et al. Amorphous zirconium metal-organic frameworks assembled from mixed porphyrins as solvent-free catalysts for Knoevenagel condensation. *Dalton. Trans.* **51**, 6631-6637 (2022).

(3) Cheng, H. et al. Nickel−alkyne−functionalized metal−organic frameworks: An efficient and reusable catalyst. *Appl. Catal A-Gen.* **623**. 118216 (2021).

(4) Lv, J., He, Z., Zhang, J., Guo, Y., Han, Z. & Bao, X. One-pot synthesis of [1,2,4]Triazolo[1,5-a]pyridines from azines and benzylidenemalononitriles via copper-catalyzed tandem cyclization. *Tetrahedron* **74**, 3996-4004 (2018).

(5) Yuan, X., Wang, J., Wan, Z., Zhang, Q. & Luo, J. One-pot Suzuki coupling-Knoevenagel condensation tandem reaction catalyzed by a recyclable magnetic bifunctional catalyst. *ChemistrySelect* **6**, 1238-1243 (2021).

(6) Xu, Z.-H. et al. Facile Access to saccharin-fused 1,4-dihydropyridines through [3+3] annulation reactions. *Eur. J. Org. Chem.* **7**, e202101423 (2022).

(7) Zhu, T., Zhang, X.-J., Zhou, Z., Xu, Z., Ma, M. & Zhao, B. Synthesis of functionalized malononitriles *via* Fe-catalysed hydrogen atom transfers of alkenes. *Org. Biomol. Chem.* **20**, 1480-1487 (2022).

(8) Gu, X. et al. New electron-donor/acceptor-substituted tetraphenylethylenes: aggregation-induced emission with tunable emission color and optical-waveguide behavior. *Chem. Asian. J.* **8**, 2362-2369 (2013).

(9) Jadhav, S. N., Patil, S. P., Sahoo, D. P., Rath, D., Parida, K. & Rode, C. V. Organocatalytic cascade Knoevenagel–Michael addition reactions: direct synthesis of polysubstituted 2-amino-4*H*-chromene derivatives. *Catal. Lett.* **150**, 2331-2351 (2020).

(10) Almáši, M., Zeleňák, V., Opanasenko, M. V. & Čejka, J. Efficient and reusable Pb(II) metal–organic framework for Knoevenagel condensation. *Catal. Lett.* **148**, 2263-2273 (2018).

(11) Huang, J., Hu, G., An, S., Chen, D., Li, M. & Li, P. Synthesis of N-alkylpyridin-4-ones and thiazolo[3,2-*a*]pyridin-5-ones through pummerer-type reactions. *J. Org. Chem.* **84**, 9758-9769 (2019).

(12) Gaussian 16, Revision A.03, Frisch, M. J. G., Trucks, W., Schlegel, H. B., Scuseria, G. E., Robb, M. A., Cheeseman, J. R., Scalmani, G., Barone, V., Petersson, G. A., Nakatsuji, H., Li, X., Caricato, M., Marenich, A. V., Bloino, J., Janesko, B. G., Gomperts, R., Mennucci, B., Hratchian, H. P., Ortiz, J. V., Izmaylov, A. F., Sonnenberg, J. L., Williams-Young, D., Ding, F., Lipparini, F., Egidi, F., Goings, J., Peng, B., Petrone, A., Henderson, T., Ranasinghe, D., Zakrzewski, V. G., Gao, J., Rega, N., Zheng, G., Liang, W., Hada, M., Ehara, M., Toyota, K., Fukuda, R., Hasegawa, J., Ishida, M., Nakajima, T., Honda, Y., Kitao, O., Nakai, H., Vreven, T., Throssell, K., Montgomery, J. A. Jr., Peralta, J. E., Ogliaro, F., Bearpark, M. J., Heyd, J. J., Brothers, E. N., Kudin, K. N., Staroverov, V. N., Keith, T. A., Kobayashi, R., Normand, J., Raghavachari, K., Rendell, A. P., Burant, J. C., Iyengar, S. S., Tomasi, J., Cossi, M., Millam, J. M., Klene, M., Adamo, C., Cammi, R., Ochterski, J. W., Martin, R. L., Morokuma, K., Farkas, O., Foresman, J. B. & Fox, D. J. Gaussian, Inc., Wallingford CT (2016).

(13) Yanai, T., Tew, D. P. & Handy, N. C. A new hybrid exchange–correlation functional using the Coulomb-attenuating method (CAM-B3LYP). *Chem. Phys. Lett.* **393**, 51-57 (2004).

(14) Lu, T. & Chen, F. Multiwfn: a multifunctional wavefunction analyzer. *J. Comput. Chem.* **33**, 580-592 (2012).

(15) Liu, Z., Lu, T. & Chen, Q. An sp-hybridized all-carboatomic ring, cyclo[18]carbon: Electronic structure, electronic spectrum, and optical nonlinearity. *Carbon* **165**, 461-467 (2020).

(16) Capaldo, L., Buzzetti, L., Merli, D., Fagnoni, M. & Ravelli, D. Smooth photocatalyzed benzylation of electrophilic olefins via decarboxylation of arylacetic acids. *J. Org. Chem.* **81**, 7102-7109 (2016).

(17) Feng, G., Wang, X. & Jin, J. Decarboxylative C-C and C-N bond formation by ligand-accelerated iron photocatalysis. *Eur. J. Org. Chem.* **39**, 6728-6732 (2019).

(18) Huang, Y., Hou, J., Zhan, L.-W., Zhang, Q., Tang, W.-Y. & Li, B.-D., Photoredox activation of formate dalts: hydrocarboxylation of alkenes via carboxyl group transfer. *ACS Catal.* **11**, 15004-15012 (2021).

(19) Banks, T. M., Clay, S. F., Glover, S. A. & Schumacher, R. R., Mutagenicity of N-acyloxy-N-alkoxyamides as an indicator of DNA intercalation part 1: evidence for naphthalene as a DNA intercalator. *Org. Biomol. Chem.* **14**, 3699-3714 (2016).

(20) Wang, Q. et al. Iridium catalysts with modular axial-unfixed biphenyl phosphine–oxazoline ligands: asymmetric hydrogenation of *α,β*-unsaturated carboxylic acids. *Org. Chem. Front.* **4**, 627-630 (2017).

(21) Seo, H., Liu, A. & Jamison, T. F. Direct *β*-Selective hydrocarboxylation of styrenes with CO_2 enabled by continuous flow photoredox catalysis. *J. Am. Chem. Soc.* **139**, 13969-13972 (2017).

(22) Lim, M. et al. Pd nanoparticles dispersed on solid supports: synthesis, characterization and catalytic activity on selective hydrogenation of olefins in aqueous media. *Appl. Organometal. Chem.* **25**, 1-8 (2011).

(23) Yang, S. et al. Enantioselective Iridium-catalyzed hydrogenation of *α*-arylcinnamic acids and synthesis of (S)-equol. *Tetrahedron* **68**, 5172-5178 (2012).

(24) Davies, H. M. L. & Jin, Q. Intermolecular C–H activation at benzylic positions: synthesis of (+)-imperanene and (−)-*α*-conidendrin. *Tetrahedron: Asymmetry* **14**, 941-949 (2003).
